# Two genomes of highly polyphagous lepidopteran pests (*Spodoptera frugiperda*, Noctuidae) with different host-plant ranges

**DOI:** 10.1038/s41598-017-10461-4

**Published:** 2017-09-25

**Authors:** Anaïs Gouin, Anthony Bretaudeau, Kiwoong Nam, Sylvie Gimenez, Jean-Marc Aury, Bernard Duvic, Frédérique Hilliou, Nicolas Durand, Nicolas Montagné, Isabelle Darboux, Suyog Kuwar, Thomas Chertemps, David Siaussat, Anne Bretschneider, Yves Moné, Seung-Joon Ahn, Sabine Hänniger, Anne-Sophie Gosselin Grenet, David Neunemann, Florian Maumus, Isabelle Luyten, Karine Labadie, Wei Xu, Fotini Koutroumpa, Jean-Michel Escoubas, Angel Llopis, Martine Maïbèche-Coisne, Fanny Salasc, Archana Tomar, Alisha R. Anderson, Sher Afzal Khan, Pascaline Dumas, Marion Orsucci, Julie Guy, Caroline Belser, Adriana Alberti, Benjamin Noel, Arnaud Couloux, Jonathan Mercier, Sabine Nidelet, Emeric Dubois, Nai-Yong Liu, Isabelle Boulogne, Olivier Mirabeau, Gaelle Le Goff, Karl Gordon, John Oakeshott, Fernando L. Consoli, Anne-Nathalie Volkoff, Howard W. Fescemyer, James H. Marden, Dawn S. Luthe, Salvador Herrero, David G. Heckel, Patrick Wincker, Gael J. Kergoat, Joelle Amselem, Hadi Quesneville, Astrid T. Groot, Emmanuelle Jacquin-Joly, Nicolas Nègre, Claire Lemaitre, Fabrice Legeai, Emmanuelle d’Alençon, Philippe Fournier

**Affiliations:** 1 INRIA, IRISA, GenScale, Campus de Beaulieu, Rennes, 35042 France; 2 INRA, UMR Institut de Génétique, Environnement et Protection des Plantes (IGEPP), BioInformatics Platform for Agroecosystems Arthropods (BIPAA), Campus Beaulieu, Rennes, 35042 France; 3 INRIA, IRISA, GenOuest Core Facility, Campus de Beaulieu, Rennes, 35042 France; 4 DGIMI, INRA, Univ. Montpellier, 34095 Montpellier, France; 5 CEA, Genoscope, 2 rue Gaston Crémieux, 91000 Evry, France; 6 Université Côte d’Azur, INRA, CNRS, Institut Sophia Agrobiotech, 06903 Sophia-Antipolis, France; 7 Sorbonne Universités, UPMC University Paris 06, Institute of Ecology and Environmental Sciences of Paris, 75005 Paris, France; 8 Department of Entomology, Max Planck Institute for Chemical Ecology, D-07745 Jena, Germany; 9 URGI, INRA, Université Paris-Saclay, 78026 Versailles, France; 10 CSIRO Ecosystem Sciences, Black Mountain, Canberra, ACT 2600 Australia; 11 School of Veterinary and Life Sciences, Murdoch University, Murdoch, 6150 Australia; 12 INRA, Institute of Ecology and Environmental Sciences, 78000 Versailles, France; 13 Department of Genetics, Universitat de València, 46100 Burjassot, Valencia Spain; 14 Estructura de Recerca Interdisciplinar en Biotecnologia i Biomedicina (ERI-BIOTECMED), Universitat de València, 46100 Burjassot, Valencia Spain; 15 EPHE, PSL Research University, UMR1333 - DGIMI, Pathologie comparée des Invertébrés CC101, F-34095 Montpellier cedex 5, France; 16 Laboratory of Mammalian Genetics, Center for DNA Fingerprinting and Diagnostics (CDFD), Lab block: Tuljaguda (Opp. MJ Market), Nampally, Hyderabad, 500 001 India; 17 Institute for Biodiversity and Ecosystem Dynamics (IBED), University of Amsterdam, Science Park 904, 1090 GE Amsterdam, The Netherlands; 18 Plateforme MGX, C/o institut de Génomique Fonctionnelle, 141, rue de la Cardonille, 34094 Montpellier cedex 05, France; 19 Key Laboratory of Forest Disaster Warning and Control of Yunnan Province, Southwest Forestry University, Kunming, 650224 China; 20 CSIRO, Clunies Ross St, (GPO Box 1700), Acton, ACT 2601 Australia; 21 Departamento de Entomologia e Acarologia, Escola Superior de Agricultura Luiz de Queiroz, Universidade de São Paulo, Av. Pádua Dias 11, 13418-900 Piracicaba, Brazil; 22 Department of Biology, 208 Mueller Laboratory, The Pennsylvania State University, University Park, 16802 Pennsylvania USA; 23 Department of Plant Science, 102 Tyson Building, The Pennsylvania State University, University Park, 16802 Pennsylvania USA; 24 CNRS UMR 8030, 2 rue Gaston Crémieux, 91000 Evry, France; 25 Université d’Evry Val D’Essonne, 91000 Evry, France; 26 INRA, UMR1062 CBGP, IRD, CIRAD, Montpellier SupAgro, 755 Avenue du campus Agropolis, 34988 Montferrier/Lez, France

## Abstract

Emergence of polyphagous herbivorous insects entails significant adaptation to recognize, detoxify and digest a variety of host-plants. Despite of its biological and practical importance - since insects eat 20% of crops - no exhaustive analysis of gene repertoires required for adaptations in generalist insect herbivores has previously been performed. The noctuid moth *Spodoptera frugiperda* ranks as one of the world’s worst agricultural pests. This insect is polyphagous while the majority of other lepidopteran herbivores are specialist. It consists of two morphologically indistinguishable strains (“C” and “R”) that have different host plant ranges. To describe the evolutionary mechanisms that both enable the emergence of polyphagous herbivory and lead to the shift in the host preference, we analyzed whole genome sequences from laboratory and natural populations of both strains. We observed huge expansions of genes associated with chemosensation and detoxification compared with specialist Lepidoptera. These expansions are largely due to tandem duplication, a possible adaptation mechanism enabling polyphagy. Individuals from natural C and R populations show significant genomic differentiation. We found signatures of positive selection in genes involved in chemoreception, detoxification and digestion, and copy number variation in the two latter gene families, suggesting an adaptive role for structural variation.

## Introduction

In phytophagous insects, adaptation to host-plants is thought to play an important role in speciation because host-plants provide a site for mating and oviposition and a food resource for progeny^1^. Comparative genomics of recently diverged phytophagous insect taxa that differ in diet range should reveal responses to selection imposed by changes in host-plant as well as reproductive isolation, and possible genetic links between the two^2^.

*Spodoptera frugiperda* (fall armyworm) belongs to the superfamily Noctuoidea that comprises more than one third of all Lepidoptera including a large number of agriculture and forest pest species. Noctuoidea diverged *ca*. 94 million years ago (Ma) from the Bombycoidea superfamily^3^ to which the lepidopteran model, *Bombyx mori*, belongs. While *B. mori* is monophagous, *S. frugiperda* is polyphagous and a major agricultural pest in the North and South American continent and Caribbean, which makes its economic importance. Also called the Fall armyworm (FAW), it can reach pest status on several of cultivated species of Poaceae^1^ (e.g. rice, wheat, sorghum and corn). Despite the preference for plants of the family Poaceae, it is increasingly becoming a pest of important broadleaf crops such as cotton and soybean in the brazilian Cerrado, especially where they are cultivated after corn^1^. FAO estimates that Brazil alone spends US$600 million each year on controlling infestations. Since January 2016, it has become invasive in Africa where it reached 12 countries^2, 3^.

It consists of two sympatric host-plant strains (Fig. S1), the “corn strain” (C strain) feeding mostly on maize, cotton and sorghum and the “rice strain” (R strain) mostly associated with rice and various pasture grasses^4^. These two strains are morphologically indistinguishable but differ by their fitness on different host-plants^5, 6^. They have diverged for *ca*. 2 Ma^7^ and show partial pre- and post-zygotic reproductive isolation^8^, however the extent of their genomic differentiation is unknown since only few genetic markers have been characterized^9–14^.

A comparison between polyphagous *S. frugiperda* and other monophagous lepidopterans (e.g., *B. mori, Manduca sexta, Danaus plexippus, Heliconius melpomene*) will shed light on the genetic basis of adaptation to host-plant changes, as a polyphagous insect should detoxify a wider variety of plant defensive chemicals. In addition, polyphagous insects need to have chemosensory genes that enable the identification of a wider range of plants for food and oviposition. Finally, they have to utilize diverse food that may differ in levels of nutrients and factors affecting digestion. In this study, we perform a comprehensive analysis of genes associated with these functions via analysis of whole genome sequence data. In addition, we analyzed the level of genomic differentiation between the two strains by re-sequencing field samples and mapping on the whole genomes of lab populations. We also investigated the existence of strain-genomic variation related to adaptation to different host-plant ranges. Our data complete the previously published genome sequence of *Sf*21 cell line^15, 16^ generated from *S. frugiperda* ovary since they offer a unique resource to infer adaptive evolution.

## Results

### A reference genome assembly for *S. frugiperda*

In order to decrease the level of heterozygosity for sequencing, we minimized the number of insects (N = 2 for C strain, N = 1 for R strain) used for sequencing. Since the assemblies obtained were fragmented (N50 of scaffold size 52.7 kb for the C strain, 28.5 kb for the R strain, N50 contigs size of 21.6 kb and 25.4 kb, respectively, Supplementary Notes S2 and S3), we took advantage of the colinearity between the strain genomes to order and orient scaffolds by aligning their genomes through a reference guided assembly procedure (Supplementary Note S9). This approach allowed us to group and order 29,949 scaffolds of the C strain reference genome, leading to 4,222 joined scaffolds (312 Mb) and 11,628 singletons (126 Mb) with a final N50 of 144 kb. The *S. frugiperda* C strain has a genome size of 396+/−3 Mb measured via flow cytometry (J. Spencer Johnson, pers. comm.), while the final assemblies encompassed 438 Mb for the C strain and 371 Mb for the R strain.

The C and R strain genomes contain 21,700 and 26,329 predicted protein coding genes, of which 21,357 and 23,055 were supported by RNA-Seq, respectively. Concerning orthology with other insects, the number of proteins in different classes of orthologous groups was similar to those of *B. mori* (Table S11 and Fig. S3).

Based on conserved synteny between Lepidoptera^17^, a set of 6,995 one-to-one orthologous genes between the C strain and the lepidopteran model *B. mori* were identified and used to physically anchor 10,531 C strain scaffolds on *B. mori* chromosomes (Fig. S7). Anchoring was based on the identification of synteny blocks containing at least two markers in the same order and orientation in both C strain and *B. mori*. Anchored scaffolds represented 43% of the C strain genome (188 Mb) and 34% (155 Mb) of the *B. mori* chromosome size.

The GC content of each strain genome was 36%. Proportion of repetitive elements in the C strain (29.16%) was similar to the R strain (29.10%) but lower than in *B. mori* (44.1%). The two strains share the same TE families, with a predominance of Non-LTR retrotransposons and SINES, like in *B. mori* ^18^ (Table S9 and Fig. S2).

Gene annotation of the corn and rice genomes and alignments against a set of anonymous transcriptomic data in various experimental conditions are available through the LepidoDB Information system at the Bioinformatics Platform for Agroecosystem Arthropods (BIPAA) Portal (Additional Information).

### Analysis of genes likely involved in polyphagy

We carefully annotated gene families known to be involved in interaction with the host-plant according to ref. 19 and compared with that of four monophagous or oligophagous lepidopteran species, such as *B. mori*, *M. sexta*, *D. plexippus* and *H. melpomene* to highlight possible molecular adaptations that could be linked to polyphagy (Supplementary Notes S11 to S23, Table S5).

**Figure 1:**
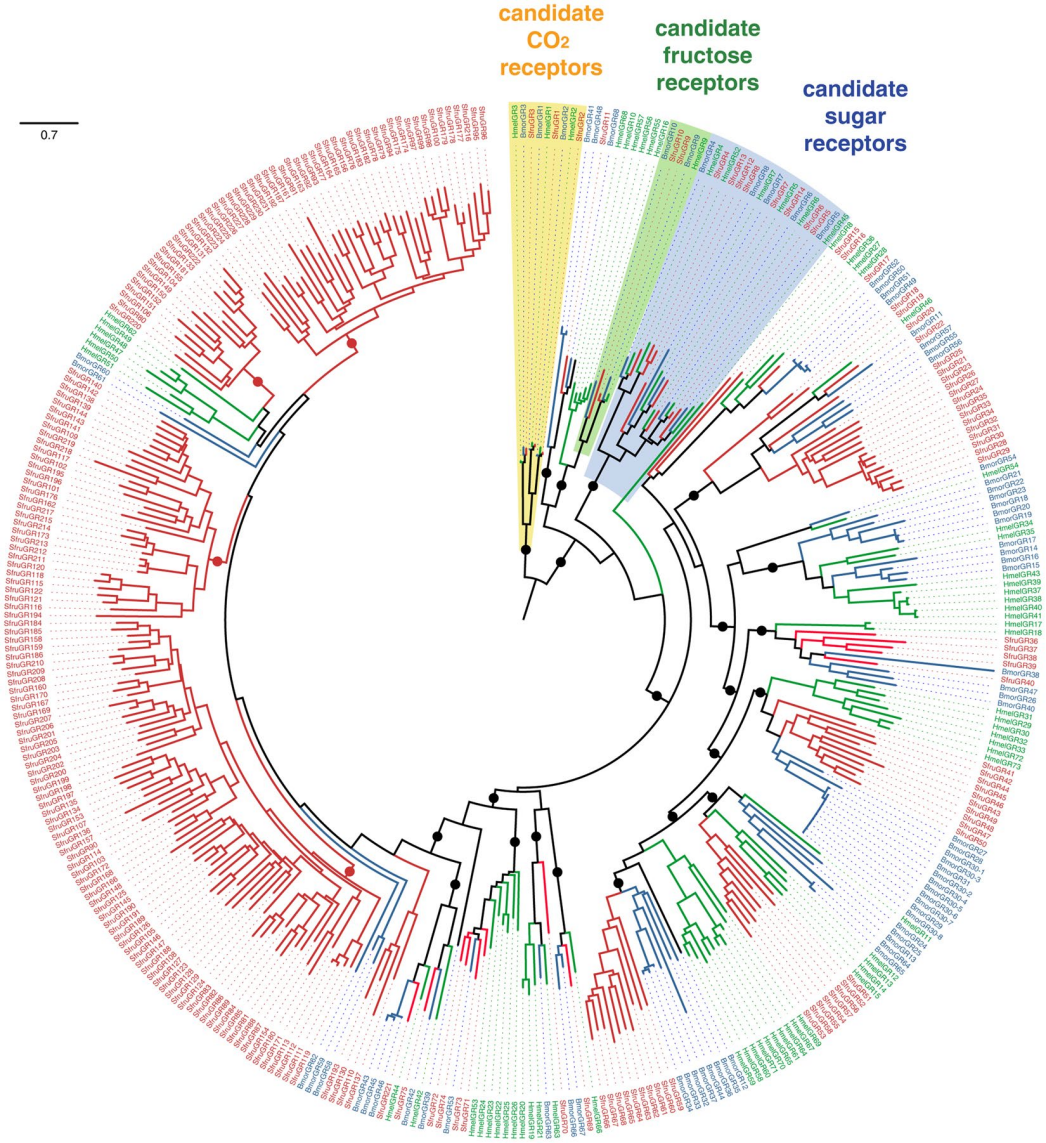
Unrooted maximum-likelihood phylogeny of the lepidopteran GRs. The amino-acid dataset included GR repertoires *from S. frugiperda* (Noctuoidea, red), *B. mori* (Bombycoidea, blue) and *H. melpomene* (Papilionoidea, green). Circles indicate basal nodes supported by the approximate likelihood ratio-test (aLRT > 0.9).

**Figure 2:**
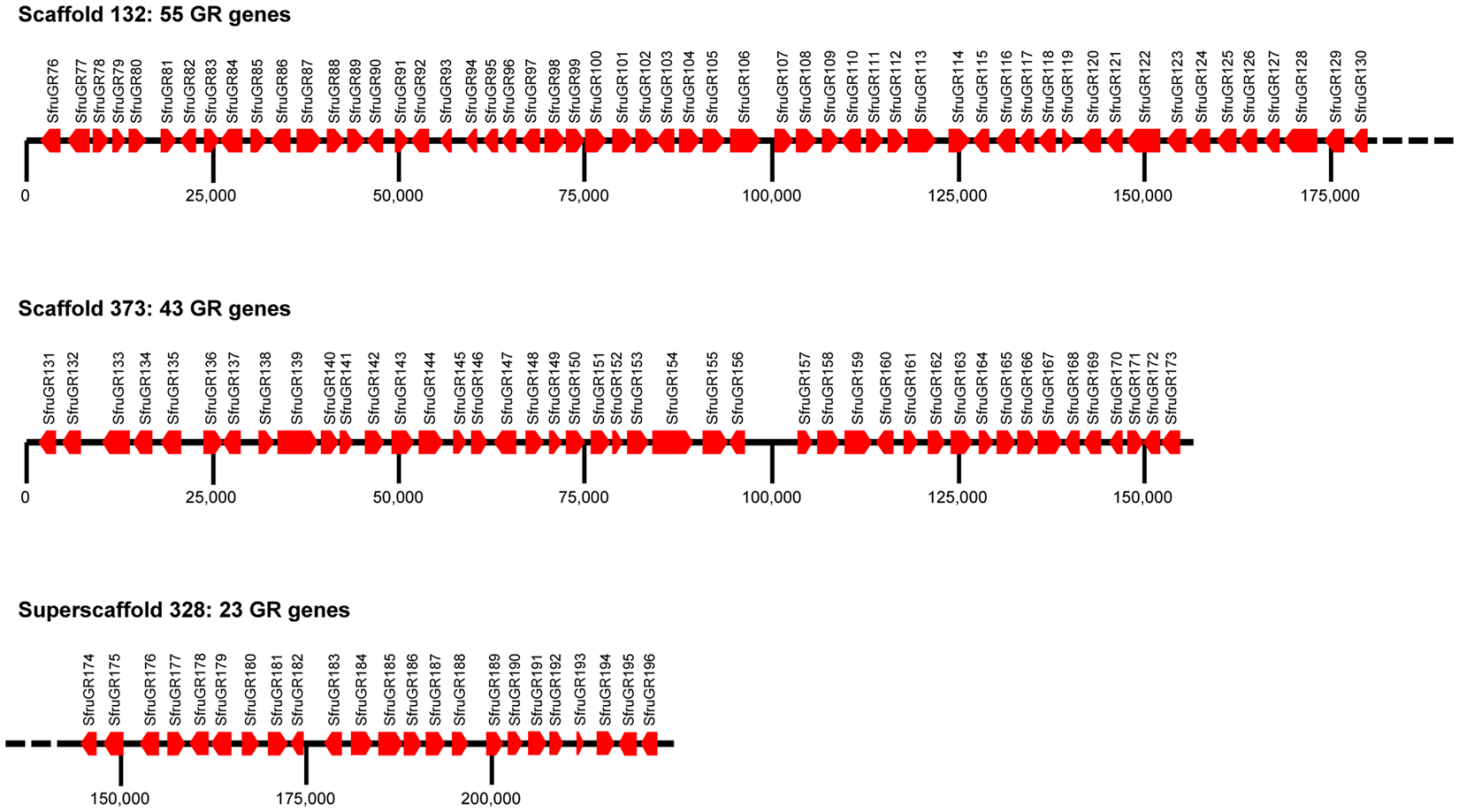
Large clusters of GR genes annotated in the *S. frugiperda* genome. Position and orientation (arrows) of genes within the scaffolds are indicated. Successive genes have been assigned successive numbers.

Chemosensory genes are involved in many recognition processes in insects, among which host-plant detection and sexual communication^19, 20^. Gustatory receptors (GRs) are expressed in taste sensilla on tarsi, ovipositors and mouthparts where they probably detect non-volatile molecules (*e.g*. sugars and bitter compounds) found on food sources and oviposition substrates^21^. We observed an incredible high number (N = 231 genes in the C strain) of candidate GR genes in *S. frugiperda* compared with non-polyphagous lepidopteran species (N = 45 to 74 genes) (Table 1 and Supplementary Note S11). Expansion mainly results from recurrent tandem duplications within four lineages of putative “bitter” receptors (see red branches in Fig. 1) as demonstrated by the presence of three large clusters of GR genes in the genome, notably one (on scaffold 132) containing 55 genes that span a 175 kb region (Fig. 2). Next we investigated the chemosensory gene families that are involved in detecting volatile molecules, namely odorant-binding proteins (OBPs), chemosensory proteins (CSP), olfactory receptors (OR) and ionotropic receptors (IR), with the latter being involved in both olfaction and taste. OBPs and CSPs are proposed to facilitate the transport of odorants to the membrane receptors. Among OBPs (50 genes in the C strain), we found expansion of 10 genes compared to *B. mori* (Fig. S9) resulting from tandem duplications within a single region of the genome (Fig. S10). The CSP repertoire (22 genes) is much more conserved when compared with *B. mori* (Table 1, Fig. S11) and we confirm the occurrence of a large number of CSP genes in phytophagous insects. The number of OR genes, (69), is very close to that in other lepidopteran species (Table 1) with no remarkable gene gains or losses (Fig. S12). For IRs, (42 genes in the C strain) we found a strong conservation of candidate antennal IRs putatively involved in olfaction^22^ but we also annotated a large number of divergent IRs likely to be involved in taste (Fig. S13). These latter genes have not been annotated in detail in other lepidopteran genomes, thus precluding further comparison.

**Table 1:** Number of genes in chemosensory, detoxification, digestion gene families found in different insect genomes. With brackets, automatic prediction, without, curated genes, *K. Mita, pers. comm., **http://supfam.cs.bris.ac.uk/SUPERFAMILY/cgi-bin/gen_list.cgi?genome = Hm

	Species	*S. frugiperda*		*B. mori*	*M. sexta*	*H. melpomene*	*D. plexippus*
	Gene family	**C strain**	**R strain**				
chemosensory	CSP	**22**	**22**	21*	19^60^	33^61^^,^	34^62^
	OBP	**50**	**51**	43*	49^63^	51^61, 63^	32^62^
	IR	**42**	**43**	25^22, 64^^,^*	21^60^	31^22^	27^22^^,^^62^
	OR	**69**	**69**	70*	71^60^	66^61^	64^62^
	GR	**231**	**230**	74*	45^60^	73^37^	47^62^
detoxification	CYP2	**8**	**8**	7*	8^60^	9^65^	[8]
	CYP3	**59**	**61**	32*	45^60^	43^65^	[36]
	CYP4	**39**	**55**	32*	34^60^	39^65^	[30]
	Mitochondrial CYP	**11**	**11**	10*	16^60^	9^65^	[12]
	GST	**46**	**45**	23	31^60^	[1]	[24]
	Esterase	**93**	**90**	73^66^	96^60^	[52]^67^	[56]^67^
	UGT	**47**	**47**	45^68^	44^60^	52^61^	46***
digestion	Protease	**86**	**112**	[143]^69^	68^29^	[180]**	?

***Manual annotation by S. Ahn, pers. comm.

These results suggest that host-plant diversification may have involved expansion of chemosensory gene families used for detecting non volatile and, to a lesser extent, volatile molecules.

Polyphagous insects must cope with toxic secondary metabolites produced by the host as well as environmental xenobiotics, which are generally detoxified by cytochrome P450s (CYPs), glutathione-S-transferases (GSTs), esterases (CCEs), and UDP-glycosyltransferases (UGTs). A total of 117 *CYP* genes (Table S15, Supplementary Note S12.1) were annotated in the C strain genome. Among four clans of CYP, strong gene expansion is observed from clan 3 (59 for *S. frugiperda* and 32 for *B. mori* or 45 in *M. sexta*), which is the most numerous type of P450s in insects and whose role in insecticide resistance is most obvious among CYP clans. From clan 3, *CYP6*, *CYP9*, *CYP321* and *CYP324* families showed an expansion in *S. frugiperda* genome compared to non-polyphagous species (Table 1 and Table S15). There are 15 members of the *CYP9* family in *S. frugiperda* versus only 4 members in the monophagous *B. mori* and none were found in the cruciferous specialist *Plutella xylostella* (diamondback moth). Interestingly several *S. frugiperda CYP9A*s are induced by 2-tridecanone or by the insecticide methoxyfenozide^23^. In *S. littoralis* and *S. exigua* members of *CYP9A* subfamily are also induced by plant compounds (quercetin, cinnamid acid, tannin) as well as insecticides (deltamethrine, methoxyfenozide)^24^. In *H armigera CYP9A12* and *CYP9A14* are induced by gossypol from cotton plant as well as by an insecticide, moreover knock out of *CYP9A12* in *H. armigera* larvae increased their susceptibility towards this insecticide^25^. Gene expansion is also observed in the *CYP4* family, clan4, which is involved in odorant and pheromone metabolism and inducible metabolizers of xenobiotics.

GSTs are another group of detoxifying enzymes that function either exogenously or endogenously, thereby increasing solubility of hydrophobic compounds and facilitating their excretion. There are 46 *GST* genes in the *S. frugiperda* genome, which outnumbers those found in the monophagous *B. mori* and *M. sexta* (Table 1), but is similar to the omnivorous beetle, *Tribolium castaneum*. Phylogenetic analysis clustered *S. frugiperda* GST with other lepidopteran GSTs in the six insect GST classes, showing recent divergence of the delta and epsilon cytosolic classes with a remarkable expansion of the epsilon class (Fig. S15, Supplementary Note S12.2).

A third group of detoxifying enzymes are esterases which form a multifunctional family that is widely distributed in animals, plants and microorganisms. Esterases are involved in xenobiotic detoxification, developmental regulation, pheromone and hormone degradation and neurogenesis. The *S. frugiperda* genome contained 96 carboxyl/cholinesterases (CCEs), 24 more than in *B. mori* but similar to *M. sexta*, with notable expansions of two clades (Fig. S16, Supplementary Note S12.3). This result is in agreement with the transcriptomic analysis of another polyphagous noctuid species, *H. armigera* ^26^. All homologues of *S. littoralis* antennal esterases were identified except two members of clade 001: *CXE7* and *CXE29* and clade 009. Most (N = 71) of the *S. frugiperda CCE*s are organized in tandem or clusters (Fig. S17).

A fourth group of detoxifying enzymes is UGTs, which catalyze the conjugation of a range of diverse small hydrophobic compounds with sugars to produce water-soluble glycosides, thereby playing an important role in the detoxification of xenobiotics and in the regulation of endobiotics^27^. We found patterns of interspecific conservation in gene number and lineage-specific expansion, mainly of the *UGT33* and *UGT40* families (Fig. S18 and Supplementary Note S12.4). The *UGT33* family of *S*. *frugiperda* showed a lineage-specific gene diversification possibly from *UGT34*, as this is also composed of four exons. Microsynteny analysis of these two families supported expansion through tandem duplications (Fig. S19).

Phytophagous insects are exposed to reactive oxygen species from pro-oxidant allelochemicals produced by the host-plant in response to herbivory in addition to those generated from endogenous sources. The antioxidant defense system is conserved in *S*. *frugiperda* compared to other insects (Table S23).

Digestive proteases are the most abundant and essential protease enzymes necessary for metabolism in herbivorous insects. In Lepidoptera, serine proteases (SP) carry out about 95% of protein digestion^28^. We found 86 digestive SP genes in the C strain genome. For comparison, in the specialist *Manduca sexta*, 68 digestive SP have been annotated, and 125 other SP genes or SP homolog genes have been identified^29^. The genome of *B. mori* contains a total of 143 automatically predicted proteases genes, 17 of which are involved in immunity, suggesting the remaining 126 are digestive (Supplementary Notes S13 and S14). All of these digestive serine proteases in *S. frugiperda* belong to the S1 family as in *B. mori*. Phylogenetic relationships inferred using the neighbor-joining method contain eleven sub-groups (Trypsin; Chymotrypsin 1, 2, 3, 4; Chymotrypsin like proteases; Diverged serine proteases 1, 2, 3, 4; and Azurocidine) in this gene family (Fig. S22). The number of proteases has increased rapidly by gene duplication, as evidenced by clusters found for instance on scaffold 448 which carries 9 chymotrypsin type 1 genes.

Although we primarily considered in this study the host-plant as an ecological niche for food and oviposition, survival on different host-plants might involve changes in an insect’s defense system against pathogens or parasites, especially when performance is stressed by feeding on a subpar host plant.

Annotation of genes involved in immunity showed that the number of genes involved in recognition (N = 45) and signaling (N = 44) in *S. frugiperda* is comparable to other insects whereas effectors (N = 50) that code for short peptides involved in antibacterial response, are slightly more numerous compared to other insects (Supplementary Note S14, Table S19).

Annotation of all *S. frugiperda* homeodomain (HD) proteins (N = 107), mostly transcription factors involved in developmental processes, showed a strong conservation of the HD gene complement compared to *B. mori* (N = 109) and the common fruit fly, *Drosophila melanogaster* (N = 107). We report a previously identified Lepidoptera-specific class of HD proteins: the Special Homeobox (Shx) class^30^, but with a unique cluster organization compared to other Lepidoptera (Supplementary Note S16, Fig. S29).

In summary, we observed remarkable and specific expansion of chemosensory and detoxification genes in the lineage of *S*. *frugiperda* and these expansion might be involved in the emergence of polyphagy in Lepidoptera.

### Comparative analysis between the corn and rice strain

To investigate whether the C and R strains correspond to different genetic entities, we compared DNA sequences between them. The probability of observing different alleles per site from a randomly chosen pair of chromosomes within all sequenced individuals, which are diploid, is far greater than that observed within either the C or the R strain (Watterson’s θ = 0.89%, 0.12% and 0.044% for total, the corn and the R strain, respectively; Supplementary Note S7).

**Figure 3:**
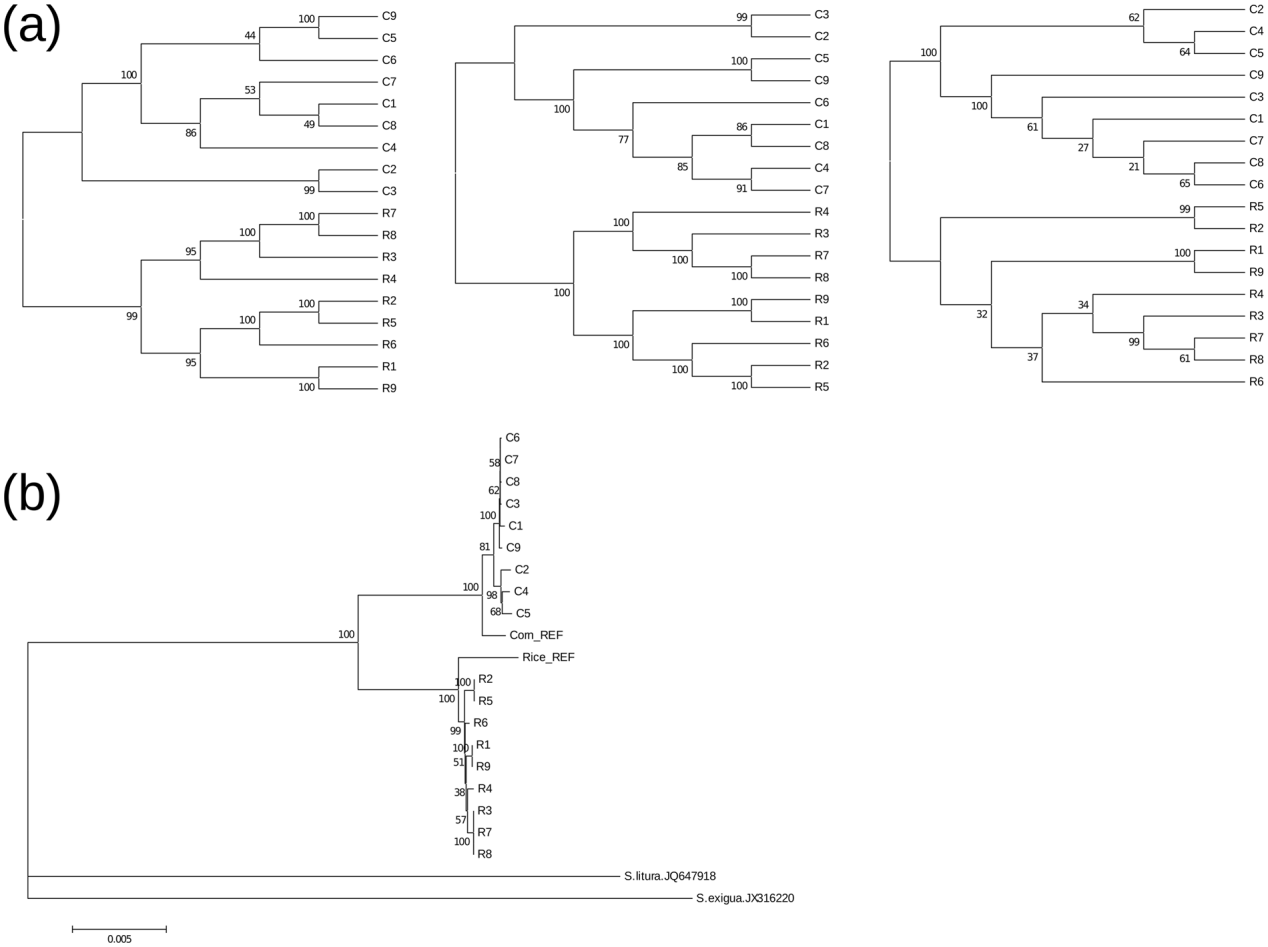
Phylogenetic relationship among individuals. (**a**) Neighbour joining phylogenetic trees of the mapping of resequencing data from natural samples of corn strains (from MS_C1 to MS_C8) and rice strain (from MS_R1 to MS_R8) against the reference genomes of the C strain (left), the R strain (middle) and mitochondrial DNA (right). The average genetic distance between pairs of individuals was estimated by the comparison of the genotype (see supplementary information for mode detail) and the distance matrix was generated from these distances. The neighbour joining tree was reconstructed using neighbour program in the phylip package with 1,000 bootstrapping, and the consensus tree was generated using the consense program in the same package. (**b**) Neighbour joining phylogenetic tree of mitochondrial genomes from natural populations of the corn (C1-C9) and the rice (R1-R9), reference sequences of the corn (Corn REF) and the rice strains (Rice REF) and outgroup species (*Spodoptera litura* and *S. exigua*). The DNA sequences of *Spodoptera frugiperda* were inferred from the VCF and those of outgroup species were downloaded from the NCBI homepage. Then, multiple sequence alignment was generated using the muscle software. The neighbour joining tree was reconstructed using MEGA software with 1,000 times of bootstrapping.

However, this divergence itself does not necessarily reveal significant genetic differentiation between C and R strains from natural populations, because genetic drift acting on lab population may reduce genetic variations severely whereas the very large effective population size of lepidopteran species may have substantial genetic variation in natural population. To determine if natural populations from which the lab strains originated are genetically differentiated from each other, we performed re-sequencing of nine individuals each from C and R populations sampled from Mississippi, USA (Supplementary Note S10). The phylogenetic tree based on whole mtDNA shows that sequence differences observed in lab strains indeed reflects true genetic differentiation. The phylogenetic tree based on the nuclear DNA of whole genomes also indicated a clear split between the R and C strains (Fig. 3, panels a and b). The Fst of mtDNA is 0.938, while that of nuclear DNA is only 0.019, which is small but significantly higher than the expectation based on randomization with 200 replicates (p < 0.0005). This result indicates that both nuclear and mitochondrial sequences have differentiated between the strains, albeit to different extents. Smaller effective population size of mtDNA due to linked selection is perhaps the primary reason of the increased Fst, but sex biased demographic history might also increase the Fst of mtDNA. The distribution of Fst along 1 kb windows of genomic sequence shows global differentiation at the whole genome scale (Fig. S8), with different extent among loci. We conclude from phylogenetic and Fst analyses that there is significant genomic differentiation between the two strains genomes. We then investigated whether there was adaptive evolution according to the host-plant ranges.

Almost no difference in number of chemosensory genes were found between the two strains (Table 1).

Concerning detoxification, variation in the composition of the CYPome in the C and R strains has been suggested for many years^31^ and is associated with a difference in susceptibility to insecticides^32^. Both strains had the same composition of *CYP* genes in clade 2 and the mitochondrial clade, consistent with these clades being ancient, conserved sequences^33^ (Table S15). Clade 3 and 4 of the CYPome show major strain differences. The majority of the 56 clades have a 1:1 ortholog relationship but three C genes (*CYP6*, *CYP9*) and five R genes (*CYP6 and CYP9*) do not share strain orthology. Clade 4 has 34 genes in a 1:1 ortholog relationship with 5 C genes (*CYP4*, *CYP340L*) and 21 R genes (*CYP340* and *CYP341* Lepidoptera-specific families) not sharing strain orthology. By PCR amplification with specific primers, we could confirm that 2 out of 3 genes tested, *CYP6AE86* and *CYP340L10*, are specific of the R strain (Supplementary Note S12.6 and Fig. S21). These differences may orchestrate adaptation to host plant allelochemicals and xenobiotics. An expansion of *CYP340L* genes occurred in R strain leading to 15 members whereas C strain contained only 9. Moreover R and C strains share only 5 orthologs in this subfamily *CYP340L*, four and ten *CY340L* are specific to C and R strain, respectively. CYP340 is a Lepidoptera-specific family that was shown to have midgut-specific expression and abundant transposable elements per gene in *P. xylostella* and where family members are organized in cluster^34^. Chromosomal rearrangements of *CYP340* cluster might have participated to the loss of nearly half of R variant members in the C strain and could explain the high plasticity observed between strains for this CYP340 family.

All C strain *GST* genes were conserved in the R strain genome except *GST8*. A comparison of their protein sequences highlighted conservation of the glutathione binding site (G site), but high variability in the substrate binding site (H site) for instance in delta GST3, epsilon GST10 and GST14, which may reflect adaptation to particular ecological niches (Supplementary Note S12.2.4).

Six CCEs identified in the C strain genome, CXE012a, CXE25 (clade 013), CXE16 and CXE24 (clade 024), and CXE025a, were absent in R variant genome (Supplementary Note S12.3). One CCE was only present in the R variant genome assembly: CCE001q, located between CXE28 and CCE001m (Fig. S17).

Amino-acid substitutions were identified in most clades, as well as insertions and deletions. This variation is particularly extensive in the very large clade 001 (Supplementary Excel Table 4).

The UGT33 and UGT34 families showed a slightly variable number of paralogs between the two strains (Fig. S18 panel B). We confirmed by PCR amplification that *UGT33-17* is specific of R strain and that *UGT40-06* is specific of C strain (Fig. S21, Supplementary Note S12.6). The amino-acid substitution rate of the UGT protein set between the strains ranged from 0 to 8%, with the highest rates occurring in the most expanded families (Fig. S20). The UGT gene families showed strain differences in their expression patterns on the same diet, either pinto bean or corn leaves (Fig. S23).

All the subfamilies of digestive serine proteases have true orthologs in both strains, with a variable number of paralogs (86 genes in C strain and 113 in R strain) (Fig. S22). Differences in the transcriptional level of serine proteases genes were also found between strains fed on the same diet (Fig. S23).

A subset of immunity genes was compared between strains without showing variation in number (Supplementary Note S14.2).

In summary we found significant gene number variation between the strains in detoxification and digestion genes, which is consistent with differential adaptation to different host-plant ranges.

The above mentioned gene number variations can result from duplications, insertions or deletions that have occurred between the strains. This possibility led us to compare the genome structure of both strains to identify structural variation. This comparison was performed by whole genome alignment of both assemblies, followed by validation using the mapping of reads on the assemblies to remove those resulting from miss-assembly of some parts of the genomes. For instance, if a deletion occurred in the C strain, it was validated when no or only few reads (<10X) of the C strain were mapped to the corresponding region in the R strain genome assembly (Supplementary Excel Table 1). Duplications were validated if the read depth over all copies in each strain was similar to the rest of the genome, using the mapping of reads of a given strain to its corresponding assembly (Supplementary Note S8).

One thousand one hundred and eight regions of the C strain covering 1.1 Mb in total, appeared to be absent from the R strain, either due to insertions of novel sequences in the C strain or to deletions in the R strain. When taking the R strain genome as reference, the same analysis generated a similar estimation of 0.9 Mb of R strain specific sequences. Eight hundred ninety two regions with different copy number between the strains were identified, approximately 80% of them corresponding to 1:2 or 2:1 duplications. Concerning balanced chromosomal rearrangements, we identified 49 inversions (59 kb) and 271 transpositions (346 kb) with an average length of 1.2 kb as events embedded in longer alignments between the reference genomes (Table 2).

**Table 2:** Rearrangements between C and R strain genomes taking the C genome as reference, *i.e*. insertions are corn-specific sequences and deletions are rice-specific sequences. Copy number gains (resp. loss) refer to duplications where the copy number is higher (resp. lower) in the C strain than in the R strain, the values refer to the number of duplication groups (not taking into account the number of copies).

	Insertion	Deletion	Copy number gain	Copy number loss	Inversion	Transposition
Number	1,108	1,009	475	417	49	271
Coverage	1.1 Mb	0.9 Mb	5.2 Mb	1.0 Mb	59 kb	345 kb

**Figure 4:**
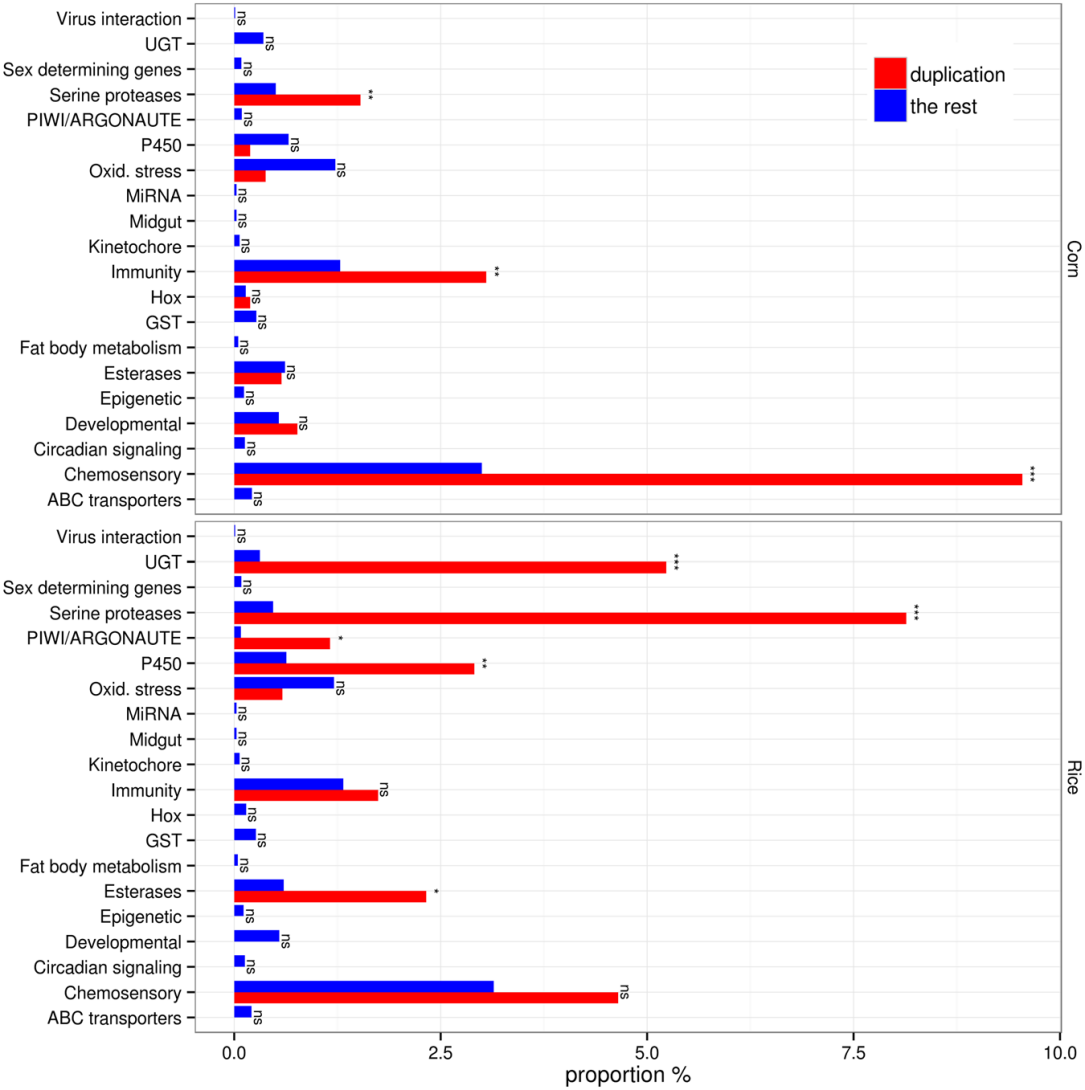
Gene content of loci with structural variation. The proportion of genes with specific functional categories in structural variation (insertion or duplication) and in the rest of genomes. ***And ns indicate FDR-corrected p-values with < 0.001 and ≥ 0.05, respectively.

Interestingly, 131 predicted genes were embedded in the C strain specific sequences (Supplementary Excel Table 1) including a UGT and a GR gene. Reciprocally, one P450 gene (*CYP9A91*) was specific to the R strain. Compared to the rest of the genome, genes associated with chemosensation, digestion and immunity were overrepresented in the regions that exhibited a higher copy number in the C strain (Fig. 4, top panel). In the R strain, it is the genes involved in detoxification (P450, UGT and esterases) and digestion (serine proteases) that were overrepresented in the regions that show higher copy number (Fig. 4, bottom panel). This suggests that the evolutionary forces inducing copy number variation are different between C and R strains and adaption to host-plant is a plausible reason for the shift in the host-ranges, analogous to the observation from the comparison between polyphagous *S. frugiperda* and three monophagous lepidopteran species.

### Analysis of genes showing signature of selection between the strains

In addition to gain-loss of genes, we also analyzed if positive selection on chemosensory, detoxification, and digestion genes has been acting by modifying pre-existing coding sequences. From 10,684 1:1 ortholog pairs (Supplementary Notes S5 and S6) between the two strains, we identified 780 genes where the proportion of codons with dN/dS greater than one is significantly higher than zero (Supplementary Note S6, Supplementary Excel Table 2). Among the 200 most differentiated genes, two are known to play a role in feeding behavior (long neuropeptide F and insulin-like peptide), seven others are candidates for host-plant chemodetection or detoxification (GR135, GR141, GR171, CSP9,*CYP6AE74*, *CYP340L16* and a GST), five are playing a role in digestion or metabolism (pancreatic lipase 2 and 3, Cathepsin B like cysteine protease, alanine aminotransferase, phosphomannomutase) or involved in the gut peritrophic membrane (mucin 2 and 4, chitin binding protein). The signatures of positive selection in these genes may reflect divergent selection on digestive and physiological traits related to recognition or processing of different plant chemicals that might have been imposed by the use of different host-plants by the two populations. However, genes that are related with chemoreception, detoxification, and digestion are not overrepresented in the list of positively selected genes (not shown).

## Discussion

Comparative genomics between *S. frugiperda* and non-polyphagous lepidopteran models, such as *B*. *mori*, *D*. *plexippus*, *M. sexta* and *H*. *melpomene* highlighted remarkable and specific expansions of chemosensory and detoxification genes.

*S*. *frugiperda* is able to extend its geographic range through annual long distance migration^35^ along which it encounters a variety of host-plants. Its host-range is reported to consist of 98 species of plants belonging to 27 families of monocots as well as dicots^7, 36^. The genetic adaptations uncovered may enable this species to feed and reproduce on a large variety of host-plants across its geographic range. Notably, duplications among the ‘bitter’ GRs have been previously observed in *B. mori* and *H. melpomene* ^37, 38^, albeit to a much lesser extent than in *S. frugiperda*. Moreover, the link between the GR expansions and polyphagy is supported by the recent discovery of 197 GRs, most from the candidate “bitter” receptor family, in another polyphagous lepidopteran pest, *H. armigera* ^39^. Great gene expansions of GRs and ORs have been found also in the omnivorous beetle *Tribolium castaneum* ^40, 41^, which suggests that they reflect ability to feed on a large variety of food.

Interestingly, we found only a few intronless GRs whereas in *H. armigera*, most of the bitter GRs are intronless, suggesting that the mechanism of gene duplication differs between *S. frugiperda* and *H*. *armigera*. In *S*. *frugiperda*, tandem duplications of DNA sequences appears to be a main mechanism whereas in *H*. *armigera* retroposition from processed mRNA may be a dominant mechanism (thus we cannot exclude the possibility that a significant proportion of GRs in *H. armigera* are pseudogenes). Tandem duplication, as evidenced by the presence of large clusters of genes in all expanded families, may be favored by the scattering of repeated elements along holocentric chromosomes of Lepidoptera (Supplementary Note S17). Our phylogenetic analysis shows that the most recent common ancestor (MCRA) of the *Spodoptera* genus was polyphagous (Supplementary Fig. S33). Polyphagy thus evolved over a long time in this genus, consistent with the observed accumulation of genetic variation in the genes linked to it. Since the transition to polyphagy is associated with the adaptive evolution of detoxification genes to neutralize diverse natural toxic chemicals in *S. frugiperda*, this species may have been pre-adapted to chemical and pesticides.

*S. frugiperda* exists as two strains living in sympatry in the whole distribution area, however their level of genetic differentiation was unknown. Our population genomics analysis supports that natural populations show significant genetic differentiation between C and R strains; both at the nuclear and mitochondrial DNA level, and homogeneously at a whole genome scale. Our expert reannotation of gene families in the two strains found strain variation in sequences and copy-number of genes involved in detoxification and digestion of plant compounds. In addition, signatures of positive selection in a set of genes having a function in chemosensation, detoxification, and digestion were identified. Copy-number variation may be under strong selection, even though we have no direct evidence of it. Signatures of positive selection in coding sequences shows that divergent selection by the host-plant was at play during strain differentiation, either initially on ancestors of their current host-plants like teosinte or grasses, or more recently to reinforce prezygotic reproductive isolation.

For ecological speciation to occur between populations with substantial gene flow, as expected from the C and R strains of *S*. *frugiperda*, a source of divergent selection by the ecological environment has to arise, in addition to evolution of prezygotic reproductive isolation^2^. In *S. frugiperda*, adaptation to a different range of host-plants can generate prezygotic reproductive isolation. At the adult stage, both C and R strains showed weak evidence of preference for their principal host-plant, corn or rice, in choice and non-choice laboratory experiments (Orsucci *et al*., in preparation). The comparative analysis of whole genomes between C and R strains suggests that copy number variation is a plausible mechanism underlying this phenotypic divergence. Whereas the total number of genes involved in detoxification and digestion is not greatly different between C and R strains, we observed that strain-specific gene expansion or shrinkage has often happened. This result is in line with the notion that shifts in host-plant range is associated with changes in number of specific detoxification and digestion genes. We also found signature of positive selection in four chemosensory genes, three GRs and one CSP, all of which might be related to divergent selection by the host-plants. The weak preference for host-plant by adults suggests that fidelity to their main host-plants is not the only prezygotic reproductive barrier between the strains. Another consistent prezygotic reproductive barrier between the two strains is their different timing of sexual activity at night^8^, which might be linked to the host-plant phenology. Therefore, we scrutinized the circadian clock genes found in the genomes of both strains (Supplementary Note S18). All of the critical clock genes – clock (*clk*), cycle (*cyc*), period (*per*), timeless (*tim*) cryptochrome-type1 (*cry1*) and type 2 (*cry2*)– were found, as well as Double-time, vrille and PAR domain protein 1 (PDP1).The *Clk*, *cyc*, and *per* coding sequences differ between strains by only two, two and one non-synonymous substitutions, respectively, whose putative role in gene expression regulation cannot be ruled out.

If speciation between two populations is led by multi-gene families, such as chemosensory or detoxification genes, it might not be possible to find the causative genes of speciation using Fst-outlier approach with resequencing data. A single read can be mapped against multiple genomic positions that carry multi-gene families with comparable confidences. Thus, variants identified from multi-gene families may not be reliable. This ambiguous mapping essentially lowers mapping score, thus resulting in likely elimination of possible variants by filtering during variant calling, but a method bypassing these mapping issues is not available.

To conclude, we provide the first exhaustive analysis of gene repertoires underlying interaction with the host-plant of a polyphagous lepidopteran pest of crops. The variation in copy number and sequences of detoxification and digestion genes between the strains suggests that they contribute to adaptation to different ranges of host-plants and thus to their genetic differentiation, either by initiating their divergence or by reinforcing of reproductive barriers.

The genomic resources generated provide the basis of a better understanding of pest physiology, that could lead in the near future to the design of new environment friendly plant protection strategies.

## Materials and Methods

Detailed methods can be found in Supplementary Notes.

### Sequencing and assembly of the nuclear and mitochondrial genomes

Whole genome sequencing was performed with Illumina HiSeq. 2000 from DNA extracted from two male larvae for the C strain and one larva of the R strain. Sequences from paired-end and mate-pair reads of multiple libraries for the C strain were assembled using the ALLPathsLG software^42^ and an in-house procedure was used to identify and correct mis-assemblies due to high levels of heterozygosity in the sequencing data. Sequences from paired-end reads of 150–170 bp DNA fragment libraries from the R strain were assembled using the Platanus software^43^ that was specifically designed to assemble sequencing data with high level of heterozygosity. The SPAdes software^44^ was used to assemble rDNA and mitochondrial DNA.

### Genes prediction, TE annotation, validation of assemblies and gene predictions

Gene models of the C strain were automatically built using GAZE^45^, based on alignment of various proteins and RNA-Seq ressources and the SNAP *ab initio* gene prediction software^46^. Gene models of the R strain were built using MAKER2^47^, and various *ab initio* gene predictors trained against a R strain reference transcriptome assembled using Trinity. A WebApollo server^48^ was made available in the SfruDB Information system to members of the consortium for manual annotation of specific gene families Assemblies and gene predictions were validated by the mapping of the Benchmarking Sets of Universal Single-copy orthologs (BUSCO, 2,675 for arthropod species)^49^ and/or BAC end sequences. Repetitive elements were annotated with the REPET package^50^.

### Orthology analysis

Orthology between insect species was inferred using OrthoMCL^51^ and orthology between the two strains was assessed with OrthoMCL and the Inparanoid softwares^52^. After aligning protein coding sequences from each 1:1 orthologous genes pair using the Prank software^53^, the signature of positive selection was tested based on the site model using the codeml software in the PAML 4.8 package^54^.

### Reference guided assembly procedure and analysis of synteny with *Bombyx* chromosome

The pairwise whole genome alignment of both *S. frugiperda* strains was conducted following the UCSC Lastz+chainnet pipeline^55^ after masking repetitive elements. This approach led to two nets, one for each strain as reference, which allowed the detection of structural variation between the strain genomes. A novel scaffolding of the C strain assembly was built using the whole genome alignment with the other strain. Only alignment chains larger than 800 bp and from the top level of the reciprocal best net (one-to-one alignments) were used at this step. If two scaffolds of the C strain aligned to a single scaffold of the R strain, then these two were merged to a single pseudo scaffold. To anchor such scaffolds on the *B. mori* chromosomes, the Cassis software^56^ was used to build synteny blocks, which contain at least two 1:1 orthologous genes in the same order and orientation between the genomes of *S. frugiperda* and *B. mori*.

### Population genomics analysis

To investigate the genetic relationship among individuals from the lab strains and sympatric natural populations, we performed 125 bp paired-end whole genome re-sequencing (HiSeq. 2500) of nine individuals from C population and nine individuals from R populations using a HiSeq. 2500. Calling of SNPs was performed using the Samtools mpileup, followed by vigorous filtering. The average genetic distance between each pair of individuals was estimated and followed by reconstructing phylogenetic trees using the neighbor program in the phylip package^57^. Weighted Fst using the vcftools^58^ estimated the level of genetic differentiation between the C and the R populations.

### Evolution of host-range in the genus *Spodoptera*

The evolution of host-range in the genus *Spodoptera* was inferred under maximum likelihood using the phytools R package^59^, which allows to reconstruct ancestral states for a continuous trait (fastAnc function). To do so, we used host-range information and the dated phylogeny from the study of ref. 7.

### Data availability

The SfruDB Information system is available through the web portal: http://bipaa.genouest.org/is/lepidodb/spodoptera_frugiperda/. The WGS reads, the two corn and rice reference genome assemblies and their gene annotation have been submitted to the EBI under the number PRJEB13110 and PRJEB13834.

## Electronic supplementary material

Supplementary Information

Dataset 1

Dataset 2

Dataset 3

Dataset 4

Dataset 5
