# Supplementary material for: Two genomes of highly polyphagous lepidopteran pests (Spodoptera frugiperda, Noctuidae) with different host-plant ranges
Source: Sci Rep. 2017 Sep 25;7:11816. doi: 10.1038/s41598-017-10461-4 (PMC5613006; doi:10.1038/s41598-017-10461-4)
Supplement: Supplementary file 1 — Supplementary Information [file 41598_2017_10461_MOESM1_ESM.pdf]

SUPPLEMENTARY INFORMATION
(NOTES, TABLES, FIGURES)

# Two genomes of highly polyphagous lepidopteran pests (Spodoptera frugiperda, Noctuidae) with different host-plant ranges.

Anaïs Gouin, Anthony Bretaudeau, Kiwoong Nam, Sylvie Gimenez, Jean-Marc Aury, Bernard Duvic, Frédérique Hilliou, Nicolas Durand, Nicolas Montagné, Isabelle Darboux, Suyog Kuwar, Thomas Chertemps, David Siaussat, Anne Bretschneider, Yves Moné, Seung-Joon Ahn, Sabine Hänniger, Anne-Sophie Gosselin Grenet, David Neunemann, Florian Maumus, Isabelle Luyten, Karine Labadie, Wei Xu, Fotini Koutroumpa, Jean-Michel Escoubas, Angel Llopis, Martine Maïbèche-Coisne, Fanny Salasc, Archana Tomar, Alisha R. Anderson, Sher Afzal Khan, Pascaline Dumas, Marion Orsucci, Julie Guy, Caroline Belser, Adriana Alberti, Benjamin Noel, Arnaud Couloux, Jonathan Mercier, Sabine Nidelet, Emeric Dubois, Nai-Yong Liu, Isabelle Boulogne, Olivier Mirabeau, Gaelle Le Goff, Karl Gordon, John Oakeshott, Fernando L. Consoli, Anne-Nathalie Volkoff, Howard W. Fescemyer, James H. Marden, Dawn S. Luthe, Salvador Herrero, David G. Heckel, Patrick Wincker, Gael J. Kergoat, Joelle Amselem, Hadi Quesneville, Astrid T. Groot, Emmanuelle Jacquin-Joly, Nicolas Nègre, Claire Lemaitre, Fabrice Legeai, Emmanuelle d'Alençon, Philippe Fournier

# Supplementary Information

SI Notes ........ 6

S1. Distribution area of *Spodoptera frugiperda*, the Fall armyworm ........ 6

S2. Nuclear and mitochondrial genomes sequencing and assembly ........ 6

S2.1 Starting material ........ 6

S2.2 Library preparation and DNA sequencing ........ 6

S2.3 Genome assembly of *Spodoptera frugiperda* (corn strain) ........ 7

S2.4 Genome assembly of *Spodoptera frugiperda* (rice strain) ........ 8

S2.5 Mitochondrial genomes and rDNA assemblies ........ 8

S3. Genome annotation and quality verification ........ 8

S3.1 Gene prediction of *Spodoptera frugiperda* genome assembly (Corn strain) ........ 8

S3.2 Gene prediction of *Spodoptera frugiperda* genome assembly (Rice strain) ........ 9

S3.3 Automatic functional annotation ........ 9

S3.4 Expert-re-annotation system ........ 10

S3.5 Quality of the assemblies and of annotation ........ 10

S3.6 Annotation of transposable elements ........ 10

S3.7 SfruDB Information system ........ 10

S4. Orthology with other insects species ........ 11

S5. Orthology between the two strains ........ 11

S6. Identification of genes under selection ........ 11

S7. Heterozygosity measurement ........ 12

S8. Rearrangement analysis ........ 12

S9. Synteny analysis ........ 13

S10. Population genomics study ........ 14

S10.1 Sampling and sequencing ........ 14

S10.2 Methods ........ 14

S10.3 Phylogenetic analysis ........ 15

S10.4 Genetic differentiation between corn and rice populations ........ 15

S11. Chemosensory genes ........ 15

S11.1 Methods ........ 16

S11.2 Soluble proteins involved in olfaction ........ 16

S11.3 Chemosensory receptors ........ 17

S12. Detoxification genes ........ 19

S12.1 Cytochromes P450 ........ 19

S12.2 Glutathione S-transferases ........ 22

S12.3 Carboxylesterases ........ 24
S12.4 UDP-glycosyltransferases ........ 25
S12.5 ATP-binding cassette transporters genes ........ 26
S12.6 Experimental validation of some interstrain differences in detoxification genes repertoire ........ 27
S13. Digestion genes ........ 27
S13.1 Methods ........ 28
S13.2 Number, phylogeny, between strain comparison ........ 28
S14. Immunity genes ........ 28
S14.1 Methods ........ 29
S14.2 Results ........ 29
S15. RNA interference genes ........ 29
S15.1 Methods ........ 30
S15.2 Results ........ 30
S16. Homeodomain (HD) genes ........ 30
S16.1 Methods ........ 31
S16.2 Results ........ 32
S17. Centromere protein genes ........ 33
S17.1 Methods ........ 34
S17.2 Results ........ 34
S18. Circadian rhythm genes ........ 34
S18.1 Methods ........ 35
S18.2 Results ........ 35
S19. Autophagy-related genes ........ 35
S19.1 *Atg1* and their regulators ........ 36
S19.2 the *Vps34* complex ........ 36
S19.3 The Atg9-dependent vesicular complex and the ubiquitin-like proteins Atg12 and Atg8 and their conjugation systems ........ 36
S20. Apoptosis-related genes ........ 38
S20.1 Extrinsic pathway ........ 38
S20.2 Intrinsic pathway ........ 39
S21. Heat Shock proteins ........ 39
S21.1 *Hsp* gene content of the corn strain ........ 39
S21.2 Comparison with rice strain ........ 40
S22. Oxydative stress related genes ........ 40
S22.1 Methods ........ 40

S22.2 Results ........ 41
S23. Neuropeptides ........ 42
S23.1 Methods ........ 43
S23.2 Results ........ 43
SI Tables ........ 44
Table S1 Statistics for different sequencing technologies performed for *Spodoptera frugiperda* genomes. ........ 44
Table S2 Statistics for *Spodoptera frugiperda* genome assemblies ........ 45
Table S3 Complete statistics of mitochondrial and ribosomal DNA sequence assembly ........ 46
Table S4 Statistics for *Spodoptera frugiperda* genomes predictions. ........ 47
Table S5 List of manually curated genes families, number of curated genes models in corn and rice strain ........ 48
Table S6 Assessment of quality of genome assemblies by mapping of BAC ends sequences. ........ 49
Table S7 BUSCO assessment of the completeness of genome assemblies ........ 50
Table S8 BUSCO assessment of the quality and completeness of automatic gene annotation ........ 51
Table S9 Genome coverage of different classes of transposable elements in the two strains ........ 52
Table S10 Protein datasets used for orthology assessment ........ 53
Table S11 Number of proteins in different classes of orthologous groups. ........ 54
Table S12 The distance between two individuals with homozygous non-variants (00), heterozygous variants (01), and homozygous variants (00) ........ 55
Table S13 Number of chemosensory genes annotated in lepidopteran genomes ........ 56
Table S14 CYP genes clan composition in various arthropods ........ 57
Table S15 Detailed list of P450 genes by clan in Corn and Rice strain of *Spodoptera frugiperda* ........ 58
Table S16 Comparison of GST gene number ........ 62
Table S17 Comparison of the CCE repertoires of *B.mori* and *S. frugiperda*. ........ 63
Table S18 Number of genes involved in immunity ........ 64
Table S19 Immunity genes ........ 65
Table S20 HD genes with paralogs in Drosophila not in Lepidoptera ........ 67
Table S21 HD genes with paralogs in Lepidoptera but not in Drosophila ........ 68
Table S22 Sequence homology of Atg8, PI3K, Akt and TOR proteins from different organisms ........ 69
Table S23 Major components of enzymatic antioxidant system of *Spodoptera frugiperda, Drosophila melanogaster, Anopheles gambiae* and *Bombyx mori*. ........ 71

Table S24 Neuropeptides genes in the Corn strain genome and their expression level ...... 72
SI Figures ........ 76
Figure S1 Distribution area of *Spodoptera frugiperda* and picture of a caterpillar on corn .... 76
Figure S2 Comparison of TE content of the two *Spodoptera frugiperda* corn and rice strains77
Figure S3 Number of proteins in different classes of orthologous groups. ........ 78
Figure S4 Number of genes with one or more orthologs for each strain........ 79
Figure S5 Number of genes having no or more paralogs in each strain. ........ 80
Figure S6 GO enrichment of genes spanning rearrangements. ........ 81
Figure S7 Synteny with *Bombyx mori* chromosomes........ 82
Figure S8 The genomic differentiation between strains........ 83
Figure S9 Unrooted maximum-likelihood phylogeny of lepidopteran OBPs. ........ 84
Figure S10 Comparison of synteny among clusters of OBP genes in *S. frugiperda* and *B. mori*. ........ 85
Figure S11 Unrooted maximum-likelihood phylogeny of lepidopteran CSPs. ........ 86
Figure S12 Maximum-likelihood phylogeny of the lepidopteran ORs........ 87
Figure S13 Maximum-likelihood phylogeny of insect IRs. ........ 88
Figure S14 Phylogenetic analysis of CYP. ........ 89
Figure S15 Neighbour-joining tree of GSTs. ........ 90
Figure S16 Phylogeny of lepidopteran esterases. ........ 91
Figure S17 Comparison between the two *S. frugiperda* strains of the genomic organization of CCE genes from the clade 001 cluster ........ 92
Figure S18 A consensus Maximum-likelihood tree of the deduced amino acid sequences of UGTs from Spodoptera frugiperda and Bombyx mori. ........ 93
Figure S19 Genomic position and orientation of the Spodoptera frugiperda and Bombyx mori UGTs. ........ 94
Figure S20 Comparison of UGT amino acid sequences between the rice and corn strains. 95
Figure S21 Experimental validation of variation in detoxification gene repertoire between C and R strain........ 96
Figure S22 Molecular phylogenetic analysis of lepidopteran Serine Proteases........ 97
Figure S23 Expression of UGT and serine proteases genes in the two strains reared on different diets ........ 99
Figure S24 Phenoloxydase genes manual annotation in corn and rice strains genomes ... 100
Figure S25 Phylogenetic tree of AGO1, AGO2, AGO3, AUB, DCR1 and DCR2 ........ 101
Figure S26 Neighbour-joining tree of HoxL family homeodomains (HD) in sequenced Lepidoptera. ........ 102
Figure S27 Phylogenetic tree of Cers family ........ 103
Figure S28 Phylogenetic tree of the Irx family and Tgif family HD proteins. ........ 104

Figure S29 The *S. frugiperda Hox3* genes cluster. ..... 105
Figure S30 Multiple alignments of Atg8 protein sequences in insects. ..... 106
Figure S31 Maximum likelihood tree of insect superoxide dismutase (SOD), based on the LG+G+I model. ..... 107
Figure S32 Amino acid sequences alignment of insect catalases. ..... 109
Figure S33 Evolution of host-plant range in the genus *Spodoptera*. ..... 110
SI References ..... 111

# SI Notes

## S1. Distribution area of *Spodoptera frugiperda*, the Fall armyworm

The two corn and rice strains leave in sympatry on the American continent. Damages on corn (Fig. S1, top right) and rice (Fig. S1, bottom right).

## S2. Nuclear and mitochondrial genomes sequencing and assembly

### S2.1 Starting material

The *S. frugiperda* laboratory strains have been seeded with 30 to 50 pupae in 2000 and 2010 for the corn and rice strain, respectively. Since then, they were reared in laboratory conditions (on an artificial diet [1], at 24°C with a 16:8 photoperiod and hygrometry of 40 %). The individuals that seeded the corn strain came from Guadeloupe whereas those that seeded the rice strain came from Florida (Gift of Dr Meagher). They have been genotyped using the *FR* repeat marker [2].

Corn strain:

Whole genomic DNA was extracted from fourth instar male larvae belonging to the same brotherhood issued from one couple of adults. One larva "4D" was used for construction of one paired ends (PE) library (177 x) and one 3 kb mate pairs (MP) library (50 x). An unrelated larva from the same population was used for construction of 5-6-7kb libraries. The bacterial artificial chromosomes (BACs) library (10 x genome) was made of DNA from thousands of eggs of the corn strain [3].

Rice strain:

Genomic DNA was extracted from one male larva issued from one couple of adults to construct the PE library.

### S2.2 Library preparation and DNA sequencing

*Rice strain*

Genomic DNA (2 µg) was sheared to a 150-700 bp range using the Covaris E210 sonication (Covaris, Inc., USA). Sheared DNA was used for Illumina library preparation by a semi automatized protocol. Briefly, end repair, tailing and Illumina compatible adaptors (BiooScientific) ligation was performed using the SPRIWorks Library Preparation System and SPRI TE instrument (Beckmann Coulter), according to the manufacturer protocol. A 300-600 bp size selection was applied in order to recover most of the fragments. DNA fragments were amplified by 10 cycles of PCR using Platinum Pfx Taq Polymerase Kit (Life Technologies) and Illumina adapter-specific primers. Libraries were purified with 0.8x AMPure XP beads (Beckmann Coulter). After library profile analysis by Agilent 2100 Bioanalyzer (Agilent Technologies, USA) and qPCR quantification, the libraries were sequenced using 100 base-length read v3 chemistry in PE flow cell on the Illumina HiSeq2000 (Illumina, USA).

*Corn strain*

An overlapping PE library and four MP libraries (about 3Kb, 6Kb, 7Kb and 8Kb) were prepared and sequenced on the Illumina HiSeq2000 (Illumina, USA). Moreover, 27155 BACs were end-sequenced using dye terminator chemistry on ABI 3730 sequencers (Applied Biosystems, France).

In order to prepare the overlapping PE library, 30ng of genomic DNA was sonicated to a 100- to 800-bp size range using the E210 Covaris instrument (Covaris, Inc., USA). Fragments were end-repaired, then 3'-adenylated and Illumina adapters were added by using NEBNext Sample Reagent Set (New England Biolabs). Ligation products were purified by Ampure XP (Beckmann Coulter) and DNA fragments (>200 pb) were PCR-amplified using Illumina adapter-specific primers and Platinum Pfx DNA polymerase (Invitrogen). Amplified library fragments were size selected on 3% agarose gel around 300 bp. After library profile analysis by Agilent 2100 Bioanalyzer (Agilent Technologies, USA) and qPCR quantification (MxPro, Agilent Technologies, USA), the library was sequenced using 101 base-length read chemistry (v1) in a PE flow cell on the Illumina sequencer (Illumina, USA) in order to obtain overlapping reads and generate longer reads of 180 bp. A 165X coverage was obtained for the overlapping PE library.
For the 4 MP libraries, 10µg of genomic DNA were sonicated separately to a 3-8 Kb size range using the E210 covaris instrument (Covaris, Inc., USA). Libraries were prepared following Illumina's protocol (Illumina Mate Pair library kit). Briefly, fragments were end-repaired and biotin labeled. A size selection of fragments with length of interest (3, 6, 7 and 8Kb) was performed. DNA was then circularized and linear, non-circularized DNA was digested. Circularized DNA was fragmented to 300-700-bp size range using covaris E210. Biotinylated DNA was purified, end-repaired, then 3'-adenylated, and Illumina adapters were added. DNA fragments were PCR-amplified using Illumina adapter-specific primers. Finally, the PCR amplified libraries (350-650 bp) were size-selected. Libraries were then quantified using a Qubit Fluorometer (Life technologies) and libraries profiles were evaluated using an Agilent 2100 bioanalyzer (Agilent Technologies, USA). Each library was sequenced using 51 or 101 base-length read chemistry (v2) in a paired-end flow cell on the Illumina HiSeq2000 (Illumina, USA). We performed 37.5X, 60.8X, 63.1X, 65X coverage for the 3Kb, 6Kb, 7 Kb and 8 Kb MP libraries, respectively (Table S1).

## S2.3 Genome assembly of *Spodoptera frugiperda* (corn strain)

*Sequence processing*
Illumina PE reads were cleaned in a three-step procedure: i) sequencing adapters and low-quality nucleotides (phred quality value < 20) were removed, ii) sequences between the second unknown nucleotide (N) and the end of the read were removed, iii) reads shorter than 30 nucleotides after trimming were discarded, together with reads and their mates mapping onto run quality control sequences (PhiX genome).

*Genome assembly*
Overlapping 100bp PE and MP libraries (3Kb, 6Kb, 7Kb and 8Kb) were assembled using AllPathsLG release 43241 [4] with default parameters. The *S. frugiperda* genome assembly v3.0 was composed of 48,272 scaffolds (N50 = 39.6 kb) totaling 526 Mb (Table S2).

*Reduction of heterozygosity*
A strategy based on self-alignment and on a read-depth analysis allowed to identify and correct mis-assemblies due to heterozygous positions. Plast [5] was used to carry out a self whole genome alignment and to get rapidly a selection of interesting pairs of scaffolds that could contain regions corresponding actually to alleles of a same locus. Only hits longer than 1 kb (or larger than 80% of the smallest scaffold), with an e-value lower than 1e-30 and with a percentage of identity equal or higher to 80% were considered, All these pre-selected pairs were then re-aligned using Lastz [6] and chained using axtChain [7]. Hypothesizing that the contigs in scaffolds were in correct order and orientation, two kinds of assembly problems due

to heterozygosity were identified and corrected: 1) a complete scaffold representing an allele of a region already assembled in the genome, 2) two alleles of a same locus located at the extremity of two distinct scaffolds. If the chain length was longer than 1 kb (or larger than 80% of the smallest scaffold) and that the added read depth of the two alleles was close to the expected read depth, these regions were merged by keeping only the allele located on the longest scaffolds involved in the chain. This correction allowed to decrease the number of scaffolds and to increase the N50 statistics.

### S2.4 Genome assembly of *Spodoptera frugiperda* (rice strain)

Reads from the 3 libraries were trimmed on 3' with PrinSeq [8] to remove low quality bases (quality < 20). An error correction step was then performed using Soapdenovo2 error correction module [9]. Assembly, scaffolding and gap closing were then performed using the corresponding modules of Platanus genome assembler [10] (kmer size = 91, mapping seed length = 50, minimum overlap length = 50). Short scaffolds (<500bp) were eliminated. The statistics for *S. frugiperda* genome assemblies can be found on Table S2.

### S2.5 Mitochondrial genomes and rDNA assemblies

We used a kmer-based approach to reconstruct mitochondrial (mt) genomes and rDNAs for both strains (Corn and Rice). We retrieved illumina reads from each WGS dataset which contain a common kmer with a given target sequence. We choose the *Helicoverpa armigera* mt genome and the *Papilio Xuthus* rDNA as closely related target sequences.

First, we filtered WGS reads using the kfir software (http://www.genoscope.cns.fr/kfir) and a 21-mer for mt genomes and 25-mer for rDNA sequences. In each case, we obtained a high coverage of the mt and rDNA sequences. We randomly sampled the illumina read subsets, to obtain a coverage around 200-400X (Table S3). Finally, we assembled each short-read dataset using spades assembler [11] with default parameters. We obtained a 15,411 bp contig and a 15,431 bp contig for respectively the corn and the rice variants that correspond to each mt genome. In the same way, we obtained a 7,817 bp contig that correspond to the rDNA of *S. frugiperda*. The reconstruction of the rDNA was not achieved during this first step; we launched a second iteration of the pipeline using the contigs obtained with Spades during the first step to complete the assembly (Table S3).

## S3. Genome annotation and quality verification

### S3.1 Gene prediction of *Spodoptera frugiperda* genome assembly (Corn strain)

Gene models were automatically built using GAZE [12] with the four resources described below, each affected with a different weight to reflect its reliability and accuracy. When applying this procedure we predicted a total of 24,447 protein-coding gene models (Table S4). Finally, genes located on merged or removed region, have been remapped using the reannotation process described below. This process in parallel to the manual curation by the FAW-International Public Consortium members generated the final protein gene coding annotation (Table S4).

*Protein sequence alignments*

A total of 152,984 *Lepidoptera* protein sequences, extracted from a selected subset of UniProt [13] was used to detect conserved genes between *S. frugiperda* and other species. The protein

sequences were first aligned against each genome assembly using Blat [14] with standard parameters, and then against the remaining sequences with no match using relaxed parameters. Each match was first refined using Genewise [15] in order to identify exon/intron boundaries and, finally, Genewise alignments were clustered, based on their genomic locations, and for each cluster, were retained the best alignment (based on score) and all alignments with scores above 90% of the best score.

*Mapping of S. frugiperda unigene set*
A collection of 54,976 transcripts from *S. frugiperda* [16] was aligned against the genome assemblies using Blat [14], with default parameters. For each transcript model, we retained the best match (based on score) and all matches with scores above 90% of the score of the best score, fixing a minimal sequence identity at 50%. Est2Genome [17] was finally used to refine Blat alignments and identify exon/intron structures. Following this procedure, 95.8% of the transcripts from the initial unigene set, were mapped onto the genome assembly.

*Ab initio gene predictions*
SNAP ab inito gene prediction software [18] was trained for each genome specificity on open reading frames derived from the Gmorse transcript models and then launched on the whole genome assembly. It predicted a total of 76,640 gene models.

*Automatic reannotation of the gene predictions located in merged or removed regions due to the heterozygosity*
We reannotated genes located on the merged allelic scaffolds. The relocation and merging of supernumerary gene annotations were performed using *Exonerate* [19] and *Augustus* [20]. The former allows the identification of location of the deleted genes onto the remaining allele. If they were not previously annotated on these target regions or if they overlap one or several annotated genes, the latter was used to predict either new genes or consensus ones. The final automatic set was merged with the manual curation annotation in the current OGS2.2 version (Table S4).

### S3.2 Gene prediction of *Spodoptera frugiperda* genome assembly (Rice strain)

First, RNA-Seq Illumina reads from 10 libraries of the rice strain were trimmed using Trimmomatic [21] and assembled using Trinity v2.0.6 [22] with the option --jaccard_clip, to build a “complete” reference transcriptome set of 81432 genes and 113710 transcripts. In order to extract the most accurate sequence, we removed the lowly expressed transcripts using RSEM [23] with the options --fpkm_cutoff 1.0 and –isopct_cutoff=15.0. The final “filtered reference transcriptome” includes 94618 sequences.

The annotation of the rice strain was performed using MAKER2 [24]. Augustus [20], SNAP [18] and GeneMark [25] were trained against the filtered reference transcriptome and used as ab initio gene predictors. Furthermore proteins from related organisms (*Drosophila melanogaster*, *Danaus Plexippus*, *Manduca sexta*, *Heliconius melpomene*, *Bombyx mori*, see Table S10 for references) were also used to guide the Maker annotation. See Table S4 for statistics on genes predictions.

### S3.3 Automatic functional annotation

The proteins sets from corn and rice strains have been compared to NR database (version 05/2015) by blastp (blasp+ v2.2.30) [26], keeping a maximum of 20 results with an e-value lower than 1e-08. The proteins functional domains have been recognized with the help of Interproscan v5.13-52.0 [27]. Peptide signals have been identified with SignalP v4.1 [28] and

transmembrane domains with Tmhhm v2.0c [29]. The Gene Ontology classification has been obtained with the help of the Blast2Go software v2.5.0 [30], using the NR and Interproscan hits that are already annotated in the database b2g_sep15 to replicate their classification on the query proteins. Each valued output from that analysis has been stored into the SfruDB information system (Described in S3.7) and are queryable and browsable on the web interface.

### S3.4 Expert-re-annotation system

A WebApollo server [31] was made available to manually curate the annotation of specific gene families (list of genes on Table S5). A total of 1933 manually curated gene models were merged with the corn variant automatic annotation to create successive Official Gene Sets. Gene curation consisted in i) naming of the gene ii) when different copies of the gene of interest were found, checking whether they corresponded to alleles of the same gene or to different genes, or to different parts of the same gene iii) correction of the structure of the gene when mapping of transcripts or RNA-Seq data allowed it. The complete method for curation is available on the wiki of SfruDB (S3.7)

### S3.5 Quality of the assemblies and of annotation

The completeness of the genome assemblies were assessed by mapping on them both ends of the 32166 BACs of the corn strain with the bwa-sw algorithm [32] and checking the number of correctly oriented alignments on a single scaffold distant by a size of 50 to 200kb. We report on Table S6 the number of BACs end pairs in the right orientation, correctly mapped on a single scaffold and distant by 50 to 200 kb.

There were  was also assessed by the mapping of the arthropods BUSCO (Benchmarking Sets of Universal Single-Copy Orthologs) v1.0 including 2675 core proteins with the supplied script (BUSCO_v1.0.py) with the options --mode all –lineage a [33] (Table S7).

The completeness of the annotation of both strains were assessed using the arthropods BUSCO v1.0 set including 2675 proteins with the supplied script (BUSCO_v1.0.py) with the options --mode OGS –lineage a [33] (Table S8).

### S3.6 Annotation of transposable elements

Repetitive elements have been annotated with the REPET package (v2.2). The TEdenovo pipeline [34] from REPET was used to build libraries of consensus sequences representative of each type of repetitive elements. For each assembly, only the contigs of length over 2 kb were used as input for TEdenovo. Consensus sequences were built only if at least five similar copies were detected. The libraries from each assembly were used for genome annotation of the respective assembly with the TEannot pipeline [35] from REPET to select the consensus sequences that are present for at least one full length copy. The selected consensus were pooled and redundancy was removed with parameters of length >= 98% and identity >= 95%. The non-redundant library was finally used to perform genome annotation of each strain with TEannot (BLASTER sensitivity = 3). The results are shown on Table S9. A comparison of the TE content of the two strains can be found on Fig. S2.

### S3.7 SfruDB Information system

An information system named SfruDB was set up to provide a bioinformatics environment dedicated to the *Spodoptera frugiperda* genome. Through a web portal
`http://bipaa.genouest.org/is/lepidodb/spodoptera_frugiperda/`
it gives access to i) a genome browser (JBrowse [31]) for each variant, ii) a WebApollo [31] server for the manual curation of the annotation of each variant, iii) a Blast [26] server, iv) a

Galaxy server [36-38] v) a synteny browser based on Circos [39], see below vi) transcript and protein reports presenting the functional annotation of the Official Gene Sets vii) an efficient search tool for retrieving gene with id, names or annotation and viii) a Gene-Ontology browser allowing to extract all *Spodoptera frugiperda* genes that belong to a Gene-Ontology category ix) a community wiki.
The access was originally restricted to members of the consortium, until the publication of the genome.

## S4. Orthology with other insects species

We inferred the relationships of orthologs between the two *Spodoptera frugiperda* strains and 4 lepidopteran species (*Bombyx mori*, *Danaus plexippus, Heliconius melpomene* and *Manduca Sexta*), plus *Drosophila melanogaster* using OrthoMCL [40] with default parameters (See Table S10 for proteome versions and references). 19,471 orthologous groups genes were identified. The number of proteins in different classes of orthologous groups can be found on Fig. S3.
On Table S11 are reported values used to obtain Fig. S3.

## S5. Orthology between the two strains

In order to identify homologs groups between the corn and rice strains, we used OrthoMCL with default parameters [40]. We also used the inparanoid v4.1 software [41] to infer homologous groups with the default parameters
Then we merged these two results. A final list of 31087 orthologous groups between the two strains was identified. Among them, 12841 were identified by both algorithms, 16318 by OrthoMCL only, and 1928 by in-paranoid only. The number of genes with one or more orthologs in each strain in shown on Fig. S4. The number of genes having no or more paralogs is shown on Fig. S5.

## S6. Identification of genes under selection

Among the orthologous groups identified by the inparanoid software [42] between the rice and the corn strains, we used 10,732 1:1 orthologous groups that show 100% of bootstrapping support to identify positively selected genes. Among them, 10,683 groups have intact protein coding sequences without internal stop codons and they are used for further analysis. For each orthologous group a codon-based pairwise alignment was generated using the prank software [43]. Poorly aligned sequences in which the codon-orthology is unclear were eliminated using a house-perl script based on the Head-Or-Tail algorithm [44] or the gblocks software [45].

For each alignment, the signature of positive selection was tested based on the site model [46] using the codeml software in the PAML package [47]. With this model, first we calculated likelihood of the alternative model in which the proportion of codons with nonsynonymous to synonymous substitution higher than 1 is allowed to be higher than zero. Second, we calculated likelihood of the null model in which this proportion is forced to be zero. To reduce the number of estimated parameters, the DNA substitution ratio of transition to transversion is forced to be 2. The significance level (p value) was calculated by fitting two times of the difference in the likelihoods between the alternative model and the null model to the chi-square distribution with degree of freedom equals to 1. Multiple testing correction was performed using FDR in the R package.

In total, 780 and 1,010 orthologous pairs show significant signatures of positive selection, based on the gblocks and the Head-Or-Tail, respectively. The genes are listed in Supplementary Excel Table2.

## S7. Heterozygosity measurement

In order to estimate the heterozygosity level in each strain genomes, we mapped the Illumina reads of each strains onto their respective scaffolds using bowtie2 [48]. Putative PCR duplicates were removed using MarkDuplicates program in Picard tools (http://broadinstitute.github.io/picard). Then we binned all the called positions into 1kb windows and calculated the average read depth of each bin using samtools [49] and bedtools [50]. If a bin has a very high or low coverage (average coverage of entire genome +/- 2 standard deviation), this bin is discarded to avoid a potential bias caused by differential coverage. The numbers of survived bins are 206,176 and 185,624 for corn and rice strains, respectively.

Bins with indels were realigned with RealignerTargetCreator and IndelRealigner programs GATK [51]. Then, SNP were called using mpileup samtools and bcftools [52]. Variants with Minor Allele Frequency (MAF) below 0.25 were filtered out.

The SNP density of corn strain is 0.12% and that of rice strain is 0.08. As the assembly of the corn variant was obtained from four chromosomes (that corresponds to two diploid individuals) whereas that of the rice variant was obtained from two chromosomes, we cannot directly compare these SNP densities. Thus, we calculated Watterson's θ with which the effect of unequal number of samples can be controlled. The Watterson's θ of corn and rice is 0.12% and 0.044%, respectively.

Then, we calculate the level of average heterozygosity level of total two populations. Both reads from the corn and the rice variant populations are mapped against the corn reference genome. And we calculated the SNP density with the same way with estimating strain-specific heterozygosity. From 206,176 1kb genes, the SNP density is 2%. As these density is calculated from six chromosomes (four from corn and two from rice), the Watterson's θ is 0.89%.

## S8. Rearrangement analysis

The pairwise whole genome alignment of both *Spodoptera frugiperda* strains was generated by UCSC Lastz+chainnet pipeline [7]. First, repetitive elements are masked. Then all-vs-all local alignments between both genome assemblies are obtained using Lastz [6]. UCSC utilities, axtChain and chainNet [7], were then used to select and chain relevant local alignments and to combine the resulted chains into nets. A net is obtained for each variant as reference. These two nets differ essentially in duplicated regions since each position of the reference genome is covered by at most one chain while this may not be the case for the query genome. To obtain a unique one-to-one whole genome mapping, a reciprocal best net was also built following UCSC guidelines. Genomic rearrangements, such as insertions/deletions, inversions, transpositions and duplications, were then identified in such data structures. Insertions of novel sequence correspond to reference regions in the net that are not covered by other chain. Deletions for one strain are obtained by looking for insertions in the other strain. In both cases, if the chain-free region is located inside a gap of another chain, the insertion site can be localized in the other strain genome. Inversions and transpositions are detected as chains nested in gaps of longer chains, and are selected according to their annotated type, qDup and qFar attributes (see the net format). For both inversions and transpositions the qDup attribute must be below 20% (meaning that the region is not duplicated in the reference genome). Inversions are such chains of type “inv” and qFar=0 bp (distance from the expected location on query). Transpositions are chains with qFar>0 bp or with a different query scaffold from the parent chain, and the gap they fill in the reference must be twice longer than the associated gap region on the query side. Specific copy number variations were detected as chains with

qDup attribute larger than 80% of the chain size, this gives regions that have at least one more copy in the reference genome than in the query genome. This selection was performed on both nets with both variants as reference. To obtain all the copies in both genomes for each putative duplication event, the candidate regions were mapped against the reference and query genomes with the tool LiftOver [UCSC]. To avoid redundancy, distinct duplication events were merged using the single linkage criterium, if significant overlap between some of their copy coordinates were found.

For all types of rearrangements, only regions larger than 500 bp, with less than 80% of transposable elements and less than 50% of N bases are kept (for duplications, this filtering step was carried out only for the first step of selection of candidate regions). For balanced rearrangements (inversions and transpositions together), to be sure the regions are not duplicated in either genome, only regions not covered (less than 80%) by detected duplication regions were kept.

For unbalanced rearrangements (insertion/deletions and copy number variations), additional filters based on read mapping were applied to filter out artefactual rearrangements that could be due to assembly issues in one or both strain genomes. For strain-specific regions (insertions and deletions), the absence of the sequence in the other strain assembly must be confirmed at the read level. For instance, a strain A- specific region (ie. an insertion in strain A or a deletion in strain B) is kept only if the read depth, when mapping the reads of strain B on the concerned region in strain A, is near zero (<10X). For each copy number variant, two read depth values were computed: one averaging the read depth of the corn strain reads over all the detected copies in the corn variant assembly, the other using the reads of the rice strain over all copies of the rice strain. The variant is kept only if both coverage values follow the read depth distributions over the whole genomes, that is they are within the following intervals: [120 – 220X] for the corn strain (ie. 170X +/- 30%) and [200 – 300X] for the rice strain (ie. 280X +/- 30%).

The number, type of rearrangements and their size are reported on Table 2 (main text).

The list of predicted genes associated to rearrangements, their annotation and Gene Ontology group is available in Supplementary Excel Table1. Gene Ontology terms enrichment within genes spanning rearrangements was tested (Figure S6)

## S9. Synteny analysis

Synteny analysis between *Spodoptera frugiperda* and its closest arthropod relative with a finished genome, *Bombyx mori*, was based on one-to-one orthologous gene assignations between the corn variant gene set and *Bombyx mori* gene set from the silkworm genome research program (http://sgp.dna.affrc.go.jp/ComprehensiveGeneSet/,[53]), using OrthoMCL [40] as described above, amounting to 6695 markers. To be able to anchor a *Spodoptera* scaffold on a *Bombyx mori* chromosome, it must contain at least 2 one-to-one orthologous genes. To increase the amount of *Spodoptera* sequences being putatively anchored, a novel scaffolding of the corn variant assembly was built using the whole genome alignment with the other variant. Only alignment chains larger than 800 bp and from the top level of the reciprocal best net (one-to-one alignments, see supplementary information note 8) were used at this step. Two corn variant scaffolds are combined in a pseudo scaffold if there exist two alignment chains at each joinable scaffold extremity, which are consecutive on a rice variant scaffold. This novel assembly can be seen as a representative assembly of both variants, rather than the strict arrangement of scaffolds in one or the other variant. Its N50 is of 144 kbp, including 4222 newly joined scaffolds (312Mb) and 11628 singletons (126Mb) and its number of "anchorable" scaffolds (including at least 2 orthologous genes) increased to 1123 (206 Mb or 47% of the genome).

Synteny blocks containing at least two markers in the same order and orientation were built using Cassis [54]. This resulted in 1150 blocks, containing 4885 markers located on 1065 *Spodoptera* scaffolds. 1440 markers were removed, the majority of them (73%) were isolated on a scaffold (scaffold with only one marker), others, either disrupted a longer synteny block (likely an orthology assignation error) or overlapped on at least one genome.
A scaffold of *Spodoptera* is considered anchored on a chromosome of *Bombyx* if all its synteny blocks map to the same *Bombyx* chromosome and the corresponding chromosomal region on *Bombyx* does not contain other blocks orthologous to another *Spodoptera* scaffold / is not disrupted by any other blocks with another *Spodoptera* scaffold. 1038 *Spodoptera* scaffolds, corresponding to 10531 corn variant scaffolds could be anchored to *Bombyx* chromosomes. This represents 188 Mbp or 43 % of the corn variant genome (Fig. S7).

## S10. Population genomics study

### S10.1 Sampling and sequencing

FAW larvae were collected from a single sweet corn field in Stoneville, MS (U.S.A.) in October, 2009. They were raised in the lab on artificial diet until adulthood. Adult females were frozen, genotyped by using mitochondrial markers [55] on DNA extracted from thorax. Nine Corn strain individuals and nine Rice strain individuals were selected after genomic DNA extraction on abdomens to be sequenced. The genotype of Corn and Rice individuals was confirmed post-sequencing by mapping of reads on the FR repeat, which is more abundant in the Rice strain[56].
Sequencing has been performed at the MGX platform in Montpellier following a paired-end 125bp design on a HiSeq 2500. The 18 individuals were sequenced on 3 lanes of the flow cell, 6 out of 9 individuals of the C strain were loaded on one lane, 6 out of 9 individuals of the R strain, and only three individuals of each strain were sequenced on the same lane, so the majority of the samples were treated separately, which minimizes the risk of signals cross contamination. Each individual sample generated between 20 and up to 70 million reads for a total of more than 260 million reads per lane.

### S10.2 Methods

Adapter sequences of fastq files were removed using the skewer-0.1.127 software [57] and all the bases that have a lower sequence quality (phred score <20) were filtered using the sickle 1.33 software [58]. If a filtered read is shorter than 15 bp, both paired-ends of the read were removed as well.
The filtered reads were mapped against the nuclear sequences of the corn and the rice strains and the mitochondrial sequences from the NCBI (accession ID: KM362176) using the bowtie2-2.1.0 software [48]. The '--very-sensitive' preset was used for the mapping, in order to maximize the sensitivity and the accuracy. Then, potential PCR or optical duplicates were filtered using the picard-tools-1.140 (http://broadinstitute.github.io/picard/). The read depth of each resequenced individuals was estimated using the samtools 0.1.1910.
SNP calling was performed using the samtools mpileup [49], followed by vigorous filtering. A variant position is discarded if the phred mapping quality score is lower than 30, or SNP quality score is lower than 20, or read depth is higher than 700, or the number of reads with alternative allele is not higher than one. In addition, if the p value of strand bias, or base quality bias, end distance bias, or mapping quality bias is lower than 0.0001, we excluded corresponding variants. And if a window size of adjacent gaps is not larger than three, we excluded this position. Finally, unless every individual has at least 1X coverage, we excluded the variant position.

To reconstruct phylogenetic tree, the distance between each pair of individuals was calculated from the genotypes in the vcf [52] (Table S12). Non-parametric sampling for each polymorphic position was performed to generate 1,000 bootstrapping distance matrices. Then, for the each distance matrix, a phylogenetic tree was reconstructed using the neighbour program in the phylip package [59] and a consensus tree was inferred using the program in the phylip software.

To reconstruct the mitochondrial phylogenetic tree, we identified complete sequences of mitochondrial genomes of each resequenced individual from the vcf and the mitochondrial reference genome (KM362176). As outgroup species, we used *Spodoptera exigua* (JX316220) and *S.litura* (JQ647918). Then, a multiple sequence alignment was generated using the muscle software [60]. The neighbour-joining phylogenetic tree was reconstructed using MEGA software [61].

Genetic differentiation between the corn and the rice populations was from the weighted Fst [62] using the vcftools v0.1.1013 [52].

### S10.3 Phylogenetic analysis

We identified 258, 14,642,556, 17,809,858 polymorphic sites from the mapping against references of the mitochondrial genome, the corn strain, and the rice strain, respectively. The neighbor-joining trees show that the grouping of rice and corn populations is strongly supported from all the mappings (Fig.3 A). To test if the reference sequences of the corn and rice strains belong to the natural population of corn and rice, respectively, we reconstructed the phylogenetic tree with the mitochondrial genome sequences including sequences from the corn and the rice strains that are used to generate reference nuclear sequences (Fig. 3B). The tree shows that the corn strain is included in the clade of the natural corn population and that the rice strain is included in the natural rice population. This result strongly supports that the difference in the sequences between the corn and rice strains reveals genetic differentiations between natural corn and rice populations.

### S10.4 Genetic differentiation between corn and rice populations

In order to estimate the level of genetic differentiation between the corn and natural populations, we calculated Fst [62]. In the nuclear genome, average Fst across the genome is ~0.0196. To test if this level of genetic differentiation can be generated by chance, we re-calculated Fst based on the randomized grouping with 2050 replicates. We found that no randomized replicate has higher Fst than the Fst based on the grouping of corn and rice populations. This result supports that corn and rice populations have been genetically diverged ($p < 0.0005$).

Fst from the mitochondrial genome is 0.938, which is much higher than the nuclear genome. This level of divergence cannot be explained by chance ($p < 0.0005$; the same test with the nuclear genome). This result indicates that mitochondrial genomes have been diverged much more than nuclear genomes and thus that mitochondrial sequences can be reliably used as a marker to identify a population that an individual of *S.frugiperda* belongs to. The distribution of Fst was calculated from 1kb windows using either corn or rice reference genomes. The horizontal red line indicates when Fst equals to zero, an expectation that there is no genetic differentiation between corn and rice strains (Fig. S8).

## S11. Chemosensory genes

The insect environment is full of chemicals that these animals use efficiently to serve different needs during their life cycle *i.e.* food and oviposition substrate choice, mating partner identification and danger avoidance. The evolution of chemosensory capacities thus plays an

important role in the adaptation of insects to a diversity of ecological niches. Several chemosensory gene families are known in insects, with distinct roles in taste and olfaction, and molecular analyses have documented the links between the evolution of these genes and insect adaptation and speciation [63-69]. Here we report the annotation of full repertoires of the different chemosensory gene families in the *S. frugiperda* genome.

## S11.1 Methods

We used described sets of Lepidoptera chemosensory gene families, especially those described in *S. littoralis* [70-72], to search the *S. frugiperda* genome by TBLASTN using Galaxy [38]. For GRs (Gustatory Receptors), we additionally used a combination of HMMER [73] and Genewise [15]. Once the scaffolds have been identified as containing candidate chemosensory genes, we used Scipio [74] and Exonerate [19] to align protein sequences on the genome and define intron/exon boundaries. Signal peptides were searched for secreted proteins such as OBPs (odorant-binding proteins) and CSPs (chemosensory proteins) using SignalP [28]. All gene models generated have been manually validated or corrected in WebApollo based on homology with other lepidopteran sequences, and on alignment with *S. frugiperda* transcripts (TR2012b) and RNAseq data, when available. The classification of deduced proteins and their integrity were verified using BlastP against the non-redundant (nr) GenBank database. When genes were suspected to be split on different scaffolds, protein sequences were merged for further analyses.

Maximum-likelihood phylogenies were created for the different gene families using amino-acid sequences. For OBPs, CSPs, GRs and ORs, datasets contained sequences annotated from the genomes of the silkworm *Bombyx mori* (super-family Bombycoidea) and the butterfly *Heliconius melpomene* (super-family Papilionoidea), together with sequences from *S. frugiperda* (super-family Noctuoidea). For IRs, the dataset contained sequences from *S. frugiperda*, *B. mori* and *Danaus plexippus* (Super-family Papilionoidea), but also from species belonging to other insect orders, namely *Drosophila melanogaster*, *Apis mellifera* and *Tribolium castaneum*. Sequences were aligned using MAFFT v7 [75], and trees were built using using PhyML 3.0 [76]. The best-fit model of protein evolution and the estimated values for the different parameters were determined using ProtTest 2.4 [77]. Node support was assessed by carrying out a hierarchical likelihood-ratio test [78].

## S11.2 Soluble proteins involved in olfaction

Odorant-binding proteins (OBPs) and chemosensory proteins (CSPs) are small globular secreted proteins, with members found in abundance in the olfactory organs [79,80]. They are characterized by conserved patterns of six and four cysteines, respectively. They are involved in disulfide bridges [81] that confer a specific domain which allows binding of different hydrophobic ligands. OBPs are supposed to bind odorant molecules and transport them through the aqueous sensillar lymph to the olfactory receptors expressed in the dendritic membranes of olfactory sensory neurons. OBPs that are specialized in binding pheromone components in Lepidoptera are called pheromone-binding proteins (PBPs) [82]. Together with the so-called general odorant-binding proteins (GOBPs), they form a Lepidoptera specific monophyletic clade within the overall OBP [82].

CSPs function is still unclear although some exhibit binding activity towards odorants and pheromones [83,84]. Whereas OBP expression is usually restricted to olfactory organs, CSPs are widely expressed in all body parts [79].

### S11.2.1 Odorant-binding proteins (OBPs)

The genome of *S. frugiperda* corn strain contains 50 OBP genes, 43 of which encoding a full-length sequence, exhibiting the signal peptide expected for these secreted proteins. The overall number of OBP genes is similar to what has been described in *Manduca sexta* and *H. melpomene* genomes, but is slightly higher than the number of OBP genes described in *B. mori* and *D. plexippus* (Table S13). For comparison, 43 OBP genes were found in *B. mori*, 51 in *H. melpomene* and 32 in *D. plexippus* [82,85-87]. We notably identified six members of the conserved PBP/GOBP sub-family [82] one of them (SfruPBP4) presenting no ortholog in the two other species included in the phylogeny (Fig. S9). PBPs/GOBPs usually reside in a single gene cluster with the noted exception of GOBP1 [82]. In the *S. frugiperda* genome, this could be confirmed at least for PBP2,3,4 and GOBP2. We also identified 6 members of the more divergent Plus-C sub-family (OBPs with more than 6 cysteines), and 5 members of the Minus-C sub-family (OBPs with less than 6 cysteines). In this latter sub-family, the number of *S. frugiperda* representatives is much reduced compared with *B. mori* and *H. melpomene*, where numerous gene duplications have been evidenced [88]. Considering the whole OBP family, 21 SfruOBPs present one single ortholog in both *B. mori* and *H. melpomene*, within highly supported clades. They may represent conserved OBPs that share a similar function among every lepidopteran. In contrast, one clade contains 19 SfruOBPs, versus 6 in *H. melpomene* and 8 in *B. mori*. This discrepancy is mainly due to a large expansion found among SfruOBP genes (Fig. S9). Interestingly, these 19 OBP genes are arranged in a cluster, located on two overlapping scaffolds (scaffold_109 and superscaffold_1125). As *B. mori* orthologs are also arranged in a cluster [86], we compared the organization of both clusters and found a conserved synteny between the two species (Fig. S10). The large expansion observed in *S. frugiperda* is due to repeated tandem duplications of one OBP ancestor gene, corresponding to BmorOBP11 (in red in Fig. S10) but duplications of two other genes also occurred (in blue and green in Fig. S10). This may correspond to a noctuidae-specific expansion of OBP genes, although additional lepidopteran species are to be included in such an analysis for further confirmation.

### S11.2.2 Chemosensory proteins (CSPs)

We annotated 22 CSP genes all encoding a full-length protein with a signal peptide. This number of genes is close to the 21 CSPs annotated in *B. mori* [87,89], but less than in *H. melpomene* and *D. plexippus* (Table S13), where butterfly-specific expansions occurred [85]. No such expansion has been observed in *S. frugiperda* (Fig. S11). Moreover, we could identify one single *B. mori* ortholog for 16 of the 22 SfruCSPs, mirroring the high conservation level between CSP repertoires of these two species. Our data confirm the occurrence of a large number of CSPs in herbivorous insects (e.g. Lepidoptera, Orthoptera) compared to the limited number identified in genomes of insects exhibiting a different lifestyle (e.g., *Drosophila* spp., 3-4; *Anopheles gambiae*, 8; *Apis mellifera*, 6; *Pediculus humanis*, 7) [87].

## S11.3 Chemosensory receptors

Among the chemosensory membrane receptors, the olfactory receptors (ORs) and the ionotropic receptors (IRs) are involved in the recognition of different volatile families as demonstrated in *D. melanogaster* [90,91]. Co-receptors highly conserved among species are required for the proper functioning of these receptors: ORco [92-94] is required to form heterodimers with ORs while IR25a and IR8a are proposed to complex with IRs [95]. Among lepidopteran ORs, sex pheromone receptors (PRs) form a clearly distinct sub-family, detecting volatiles meant for intraspecific communication, such as sexual communication [96,97]. Another chemosensory receptor family is the highly divergent gustatory receptors (GRs)

family. The GRs are found in taste organs and are believed to detect non-volatile molecules such as sugars and bitter compounds found on food sources and oviposition sites [98]. In addition, some members of the GR family are involved in carbon dioxide detection [99].

### S11.3.1 Olfactory receptors(ORs)

We annotated 69 OR genes in the genome of *S. frugiperda*, including the co-receptor Orco gene. The number of OR genes (Table S13) is close to what has been described in other Lepidoptera (e.g. 64 in *D. plexippus*, [85]; 73 in *M. sexta* [100]) and to what we found during our own reannotations of available genomes (70 in *B. mori* and 66 in *H. melpomene*). ORs from all three species fell into 15 highly supported clades (Fig. S12). In none we observed remarkable *S. frugiperda* OR gene losses or expansions. This mirrors the overall conservation of the OR repertoire between different lepidopteran species. Six ORs (SfruOR6, 11, 13, 16, 56 and 60) clustered in the sub-family of candidate sex pheromone receptors. Orthologues of SfruOR6, 13 and 16 have been functionally characterized in other *Spodoptera* species: SlitOR6 and SexiOR13 bind (*Z,E*)-9,12-tetradecadienyl acetate [101,102], SexiOR13 also binds (Z)9-tetradecenyl acetate [102] (the major component of the *S. frugiperda* sex pheromone blend) and SexiOR16 binds (*Z*)9-tetradecenol [102]

Apart from candidate pheromone receptors, we identified one ortholog (SfruOR51) of the cis-jasmone specific larval receptor of *B. mori* [103], one ortholog (SfruOR28) of a cis 3-hexenyl acetate receptor of *S. litura* [104],one ortholog (SfruOR3) of the citral receptor of *Epiphyas postvittana* [105] and of the trans-farnesene receptor of *S. exigua* [106]. It is not known if these chemical are behaviorally active in *S. frugiperda*, and if any can activate any SfruORs. Further functional studies would be useful to evidence (or not) any functional conservation.

### S11.3.2 Ionotropic receptors (IRs)

We annotated 42 IR genes in the *S. frugiperda* genome, 28 of which encoding a full-length sequence. In addition to the highly conserved co-receptors IR8a and IR25a, we identified 17 candidate antennal IRs putatively involved in odorant detection (Fig. S13). The repertoire of candidate antennal IRs of *S. frugiperda* is globally similar to that of other Lepidoptera. SfruIR1, 2, 3 and 31a did not have any orthologue outside Lepidoptera, and may belong to Lepidoptera-specific IR lineages [107]. We also identified 23 divergent IRs, related to the candidate taste receptors identified in *Drosophila* [108]. The number of divergent IRs is much higher in *S. frugiperda* than in *B. mori* and *D. plexippus*; the reason may be that the entire set of divergent IRs has not been yet annotated in other lepidopteran species. Most divergent SfruIR genes (SfruIR7d.1 to 3 and SfruIR100a to r) were intronless, as those from *Drosophila* [109], and some of them were arranged in tandem.

### S11.3.3 Gustarory receptors (GRs)

We annotated 231 GR genes in the genome of *S. frugiperda*, a number far higher than what has been annotated in other lepidopteran genomes (60-70 GR genes) (Table S13). The phylogenetic analysis (Fig. 1, main text) revealed the presence of conserved clades classically observed in most insect species [110,111] including 3 putative CO2 receptors (SfruGR1, 2 and 3), 8 putative sugar receptors (SfruGR4, 5, 6, 7, 8, 12, 13 and 14) and 2 candidate fructose receptors (SfruGR9 and 10). All the other GR genes we identified belonged to the so-called bitter receptor clade, which groups the vast majority of lepidopteran GRs in lineage-specific clades that expanded through an extensive number of gene duplications. Interestingly, while expansions observed in *B. mori* and *H. melpomene* [112,113] are rather limited, we evidenced in *S. frugiperda* several lineage-specific GR clades containing a very large number of genes,

sometimes more than 50 (Fig. 1). These incredible expansions result from repeated tandem duplications, as demonstrated by the presence within the genome of large clusters of GR genes, notably one (on scaffold 132) containing 55 GR genes spanning a 200 kb region (Fig. 2). To our knowledge, such large genomic clusters of GR genes have never been described in any insect genome, except in a recent study conducted on another noctuid moth *Helicoverpa armigera* [114]. Notably, GR genes in other lepidopterans have been observed to be mostly distributed as singletons and small gene clusters [113,115]. The expansions we observed may reflect an adaptation to the polyphagous diet of the *S. frugiperda* larvae, which need to perceive via GRs a large variety of secondary plant chemicals. By contrast, the larvae of the other lepidopteran species considered here (*B. mori* and *H. melpomene*) feed exclusively on, mulberry and passiflora leaves, respectively, which may require a smaller number of GRs.

## S12. Detoxification genes

### S12.1 Cytochromes P450

Cytochrome P450 or CYP genes constitute one of the largest gene families with representatives in nearly all living organisms from viruses, Archaea, bacteria, fungi, plant vertebrates and insects [116]. CYP are heme-containing monooxygenases that generally catalyze the insertion of one oxygen atom in a substrate after activation of molecular oxygen. Many CYP are involved in the metabolism of key endogenous substrates such as steroid hormones and lipids but CYP are also associated to the metabolism or detoxification of xenobiotics such as plant natural product and pesticides. CYPs, by enabling insect to overcome plant chemical defenses are key component of successful adaptation to their host plant [117].

Identity between two CYP proteins can be as low as 25 percent but conserved motifs spread along the sequence allow clear identification of CYP sequences. Conserved CYP protein structure is formed of a four-helix bundle (D, E, I and L), helices J and K, two sets of β sheets and a coil called the “meander”. Conserved motifs include WXXXR in the C helix, the conserved Thr of helix I, EXXR of helix K and the PERF motif followed by the heme binding region FXXGXXXCXG around the axial Cys ligand [116]. Based on their sequence identity, insect CYPs are classified into 4 clades: the CYP2 clade, the CYP3 clade, the CYP4 clade and the mitochondrial clade.

#### *S12.1.1 Methods*

P450 genes of *Spodoptera frugiperda* maize genome were first searched by TBLASTN against the whole genome assembly using CYP protein sequences from *Bombyx mori* [118] as well as 42 *Spodoptera frugiperda* CYP sequences previously identified [119]. All the scaffolds containing candidate CYPs were manually annotated to identify intron/exon boundaries and reported in WebApollo. Protein CYP sequences were sent to D. Nelson (http://drnelson.uthsc.edu/CytochromeP450.html) for name attribution following the CYP nomenclature [120]. Protein sequences from *B. mori*[118] and *S. frugiperda* corn and rice strain above 300 amino-acids were kept to performed alignments using MAFFT v7program using E-INS-i option [75]. Alignment were manually checked and edited and only conserved region of CYP sequences were kept for further analysis. Finally, 390 sequences with sequence length of 282 amino-acids were used in tree inference using Bayesian method. An unrooted phylogenetic tree was constructed with Bayesian analysis implemented in MrBayes 3.2 program [121]. For Bayesian phylogenetic inference, firstly we used ProtTest 3.4 [122] to determine the best fitting model of amino acid substitution for the data under the maximum likelihood assumption. A LG model turned out to be the best model and was utilized in Bayesian analysis subsequently. 100,000 generations were run and congruence was reached

with the average standard deviation of split frequencies being inferior to 0.05. Consensus tree and statistics were obtained after "burning" 25% of generated trees. Posterior probability support values are reported for each node. The final unrooted tree diagram was generated using MEGA Tree Explorer[123] (Fig. S14).
Few *S. frugiperda* CYPs were not included in the phylogenetic analysis because their sequences contained less than 300 amino-acids: SFMCYP333A12, SFMCYP305B1, SFMCYP6B38, SFMCYP9A32, SFMCYP9A60, SFMCYP9A76, SFMCYP4M17, SFMCYP4S8, SFMCYP4S9, SFMCYP340L11, SFMCYP340L15, SFMCYP340L18, SFMCYP340L21 and SFRCYP366A1, SFRCYP354A14, SFRCYP6AE69, SFRCYP6AE73, SFRCYP9A32, SFRCYP9A60, SFRCYP9BS1, CYP340L13, SFRCYP340AH and SFRCYP4CG18.

### *S12.1.2 Gene number*

We annotated 203 *CYP* gene models of which 148 were informative enough to be sent to D Nelson for naming (Naming requires that the genes or fragment of genes are long enough to be distinguished from the ones already contained in Dr Nelson's database). Among these 148, 58 included a full-length ORF and some of the gene fragments could be merged, leading to a final set of 117 *CYP* genes in our CYPome annotation. *CYP* were detected on 129 scaffolds in *S frugiperda* corn variant. Some genes were split on different scaffolds. The majority of these scaffolds (117) contain only one *CYP* and 18 scaffolds contain at least 2 CYPs. Scaffold_1102 had the largest *CYP* cluster with 5 *CYP*s all belonging to the *CYP341* family. The number of *SfruCYP*s is slightly higher than the number of *CYP* genes described in other Lepidoptera genomes except for *Manduca sexta*. For comparison, 81 *CYP* genes were found in *B. mori*, 85 in *Plutella xylostella* [124], 100 in *Heliconius melpomeme* [125] and 117 in *Manduca sexta* [100]. A description of clan composition is given in table (Table S14)

Clan 2 and mitochondrial clan which are evolutionary conserved contain 8 and 11 members, respectively, 59 *CYP*s were assigned to clan 3, and 39 to clan 4 (Table S14). *CYP9A28*, was missing in the corn genome assembly, however this gene was sequenced from a BAC obtained from the same corn strain [119]. A new family, *CYP3097*, was discovered in clan 3, containing only one member. Few families showed an expansion in the *S frugiperda* genome compared to *B mori*, *CYP6*, *CYP9*, *CYP321*, and *CYP324* from clan3 and *CYP4* from clan 4.

### ***S12.1.3 Strains comparison***

In rice strain we annotated 170 *CYP* gene models, all of them were informative enough to be sent to D. Nelson for naming. Our rice strain CYPome annotation corresponds to 136 *CYP* genes. Of the 136 rice strain *SfruCYP* genes, 88 include a full-length ORF. *CYP* were detected on 123 scaffolds in *S frugiperda* rice variant. The majority of these scaffolds (92) contain only one *CYP* and 31 scaffolds contain at least 2 *CYP*s. Scaffold_005057 had the largest *CYP* cluster with 6 *CYP*s all belonging to the *CYP9* family. A description of clan composition is given below in Table S15. Clan 2 and mitochondrial clan which are evolutionary conserved contain 8 and 11 members, respectively, 61 *CYP*s were assigned to clan 3, and 55 to clan 4. A phylogeny of corn and rice proteins sequences as well as *B mori* sequences was performed for each of the 4 CYP clans (Fig. S14) to identify orthologs.
No difference between rice and corn strain were found in the clan2 and the mitochondrial clan which are evolutionary conserved clan. However clan 3 and 4 present major differences between the two strains.
Some clan 3 members are involved in adaptation to plant allelochemicals as well as to resistance to insecticides. In Lepidoptera members of CYP6B family have been implicated in detoxifying a variety of allelochemicals such as furanocoumarins in *Papilionidae* [126] and

xanthotoxin in *Helicoverpa* [127] and nicotine in *Manduca sexta* [128]. Only one CYP6B is found in *B. mori* and no member of this sub-family was found in *P xyllostela* whereas 7 and 6 members were respectively found in corn and rice strain of *S frugiperda*. The additional gene found in corn genome is a pseudogene *CYP6B65P*. In *S frugiperda CYP6B39* is induced by xanthotoxin, indole and indole-3-carbinol [119]. In *H armigera CYP6AE14* and *CYP6AE11* are induced by gossipol from cotton plants [129], and *B mori CYP6AE22* is induced by an organophosphorous pesticide [130]. *CYP6AE* subfamily presents similar number of members in *B mori* and corn and rice strain of *S frugiperda* with respectively 10, 11 and 12 gene copies but *P. xylostella* contains only 2 members. Three genes from this subfamily, the rice specific *CYP6AE86* and *CYP6AE87* and the corn specific *CYP6AE49*, derived from recent duplications events involving *CYP6AE72*, *CYP6AE73* and *CYP6AE75*, respectively and could be involved in adaptation to plant host. Additionally corn *CYP6AE74* has been shown to be under positive selection. Several *SfruCYP9A*s are induced by 2-tridecanone and another one is induced by methoxyfenozide, an insecticide [119]. In *S litoralis* and *S exigua* members of CYP9A subfamily are also induced by plant compounds (quercetin, cinnamid acid, tannin) as well as insecticides (deltamethine , metoxyfenozide [131]). Clan 3 subfamily, *CYP9A*, presents different composition with 14 and 15 members in corn and rice strains, whereas only 3 genes of *CYP9A* family are present in *B mori* genome and none were found in *P. xyllostella*. *CYP9A76* is corn-specific. *CYP9A91* and the pseudogene *CYP9A28P* are rice specific. Three members of *S. frugiperda* and one from *Helicoverpa zea CYP321* subfamily are induced by plant compounds [119]. Eight members of this subfamily were annotated in both rice and corn strain whereas no copy was found in *B mori* and only one in *P xylostella*. No information for the role of *CYP324* subfamily members is available but 3 copies were found in both corn and rice genome whereas only one member is found in *B mori* and none in *P. xylostella*.

Clan 4 is a highly diversified group of enzymes in insects with roles in pesticide metabolism, development and chemical communication. *CYP340* and *CYP367* are Lepidoptera-specific families. CYP4 family members are involved in odorant metabolism [132] as well as in cuticular hydrocarbon biosynthesis [133]. There are two times more members in this family in corn and rice strain of *S. frugiperda* (18, 17, respectively) compared to *B mori* and *P. xylostella* (9 and 8 members, respectively). In addition, two CYP families from clan4, *CYP340* and *CYP341*, present striking differences between corn and rice variant. Single-member subfamilies *CYP340G*, *CYP340Q*, *CYP340AB, CYP340AH*, *CYP340AX* were only found in the rice variant, one additional member of *CYP340AD* subfamily was found in rice strain, and the *CYP340AA* subfamily, found only in rice, contained 3 members including 2 pseudogenes. A blooming of *CYP340L* occurred in rice variant leading to 15 members whereas corn variants contained only 9. Moreover rice and corn variants only share 5 orthologs in this subfamily *CYP340L*, four and ten *CY340L* are corn and rice -variant-specific, respectively. *CYP340L16* is under positive selection in the corn variant. CYP340 is a Lepidoptera-specific family that was shown to have midgut-specific expression and abundant transposable elements per gene in *P. xylostella* [124] and where family members are organized in cluster [124]. Chromosomal rearrangements of *CYP340* cluster might have contribute to the loss of nearly half of rice variant members in the corn variant and could explain the high plasticity observed between rice and corn variants for this *CYP340* family. In swallowtail butterfly, *Papilio Xuthus*, *CYP341A2* is preferentially expressed in the chemosensory organs and is related to the chemosensory reception for host plant recognition [134]. *CYP341B14* from lepidopteran *Hypantria cunea* is involved in the biosynthesis of sex pheromone from dietary linolenic acid [135]. In *S frugiperda CYP341* family shared 4 members between corn and rice strains with an additional three members specific to rice variant that might mirror the highest plant host range of rice variant compared to corn one.

### S12.2 Glutathione S-transferases

Glutathione S-transferases (EC 2.5.1.18, GSTs) belong to a gene superfamily present in most species, from prokaryotes to eukaryotes. GSTs can be divided into several classes based on their cellular localizations (cytosolic or microsomal), substrate specificities and phylogenetic relationships: the cytosolic class contains seven subclasses (Delta, Epsilon, Omega, Sigma, Theta, Mu and Zeta). GSTs catalyze the conjugation of reduced glutathione (GSH) to hydrophobic compounds, either exogenous or endogenous, that increases their solubility, thus facilitating their excretion. The cytosolic GSTs are dimeric proteins (homo- or heterodimers) with the active site composed of two binding sites: the conserved G-site, which binds reduced GSH, and the highly variable H-site that binds their substrate, allowing GSTs to detoxify a variety of hydrophobic substrates.

In insects, six cytosolic subclasses are found (lacking members in the Mu subclass). Insect GSTs are particularly involved in the detoxification of xenobiotics and the expression level is often correlated with enhanced insecticide resistances. Particularly, members of the delta and epsilon subclasses are specific to insects and have been implicated in resistance to various pesticides, while the omega, theta, zeta and microsomal sub-groups appear to be involved in other cellular processes, including protection against oxidative stress.

This highly diverse gene family is known to be rapidly evolving in insects in response to selection pressures (like exposure to new insecticides or changes in environmental conditions). Multiple gene duplications and polymorphism had been shown to drive this diversity. Here, based on data mining from *S. frugiperda*, we report the repertoire of GST gene families, and their evolution in rice and corn strains.

#### *S12.2.1 Methods*

We used described sets of Lepidoptera GST proteins, (especially the families described in *S. litura* [136-138], to search the *S. frugiperda* genome by TBLASTN using Galaxy [139]. Once the scaffolds have been identified as containing candidate GST genes, we used Scipio [74] and Exonerate to align protein sequences on the genome and define intron/exon boundaries. All gene models generated have been manually validated or corrected in WebApollo based on homology with other lepidopteran sequences, and on alignment with *S. frugiperda* transcripts (TR2012b) and RNAseq data, when available. Alternatively, a direct query search was used using keyword search at the LepidodB website (http://www6.inra.fr/lepidodb). The classification of deduced proteins and their integrity were verified using BlastP against the non-redundant (nr) GenBank database. When a gene is found from multiple scaffolds, protein sequences were merged.

Neighbour-joining tree was constructed with different gene families from the genomes of *Bombyx mori*, *Spodoptera litura*, *Drosophila melanogaster*, *Anopheles gambiae*, *Apis mellifera*, *Nasonia vitripennis*, *Locusta migratoria* and *Tribolium castaneum*. Sequences were aligned using MAFFT v7 [75], and tree was built using the BioNJ algorithm, as implemented in SeaView v4. Node support was assessed by carrying out a bootstrap analysis with 1000 replicates.

GSH and substrate binding sites were analyzed using the NCBI CD-search program.

#### *S12.2.2 Gene number*

We annotated more than 60 GST sequences, corresponding to 46 GST genes (partial and complete sequences). This number is comparable with other overall GST number found in other insect genomes (Table S16) including *T. castaneum* or *A. gambiae*, but so far higher than any other insect species (even more than other *Spodoptera* species, like *S. litura* with 37 known genes). This great GST number in a given species could be related to the

environmental complexity where the insects live. As generalist pests, *Spodoptera* moths may need a diversified repertoire of detoxification enzymes to cope with various toxicants found in different host plants.

Of particular interest is the repartition of identified genes according to GST subclasses with an over representation (more than 50% of cytosolic genes) of epsilon subclass. This trend indicates greater duplication events than in the other four subclasses, possibly linked to environmental adaptation or insecticide resistance.

### S12.2.3 Phylogenetic analysis

Based on sequence comparison (corn variant) we constructed a Neighbour-joining tree with different insect species (Fig. S15).This analysis illustrated the seven subclasses of GSTs, and allowed the clustering of *S. frugiperda* GSTs into their relevant phylogenetic branches. In all the subclasses, the *S. frugiperda* GSTs were all clustered into the Lepidoptera specific branches.

This tree analysis revealed that insect-specific epsilon and delta GSTs seem to have diverged recently from the other subclasses. Moreover, the epsilon subclass is characterized by a remarkable expansion and is clustering in a lineage-specific clade of Lepidoptera that might have same or similar functions, (Fig. S15)

To further analyze *S. frugiperda* GSTs, we compared protein sequences with corresponding members of the same classes from other insect species (Not shown). This analysis revealed the presence of a common motif, E/QSxAIxxYL/I in all the identified cytosolic GSTs. In the microsomal GSTs, a motif, DPxVERVRRAHxNDxENILPx was identified.

Amino acid residues that interact with GSH were conserved in every subclass of GSTs (G-site), whereas the substrate binding pocket (H-site) appears more variable among the different subclasses and even in each given member of a specific subclass. Thus, the conserved G-sites seem to indicate their importance in enzyme function while the variable H-sites are more related to their evolutionary adaptation towards new substrates.

Altered G-sites were found for sigma GST7 and for omega GST3, suggesting either these enzymes to be non-functional or involved in alternative functions as intracellular transporters.

### S12.2.4 Strain comparison

We compared both the number of genes and their relative amino acid sequences for each strain. For every subclass of GST in the corn strain, we were able to identify the corresponding gene in the rice strain, at least partially (Supplementary Dataset S5, GST Tab). GSTs show a high sequence identity between strains. This is particularly true for the G-sites that appear conserved in all subclasses. Most of the observed differences were located at the C terminal part of the sequences, associated with the H-site. This highly variable region associated with the substrate specificity is marked by numerous polymorphisms, ranging from very few amino acid substitutions to totally alternative alleles. A striking example is the comparison of delta and epsilon GSTs: delta GST3, epsilon GST10 and epsilon GST14 for instance share a common G-site region in the two strains, whereas the H-sites are drastically different, leading to almost independent proteins (Not shown). This observed diversity could involve those GSTs in the adaptation to the strains particular ecological niches and are perhaps particularly important in the detoxification of environmental xenobiotics.

### S12.2.5 Conclusion

*Spodopoptera frugiperda* GSTs form a complex, multigenic family of enzymes that fulfill diverse important protective roles. Their potential roles in insecticide resistance and protection against oxidative stress are probably crucial in this species, and our analysis highlights the rapid expansion of this important enzyme class. Further investigations, particularly using biochemistry and molecular biology experiments would drive a better understanding of their importance in Lepidoptera adaptation to their relative environment.

### S12.3 Carboxylesterases

Esterases, (CCEs), form a multifunctional family of enzymes widely distributed in animals, plants and microorganisms involved in xenobiotic detoxification, development regulation, pheromone and hormone degradation and neurogenesis [140]. In insects, these enzymes are divided into three phylogenetic classes, subdivided further into 33 clades [141]. Class 1 includes intracellular xenobiotic-metabolizing CCEs, class 2 contains extracellular xenobiotic, hormone and pheromone degrading enzymes and class 3 comprises non-catalytic CCEs involved in cell adhesion and neuron development. The two first classes expanded after the separation of the different insect orders. In contrast, the class 3 CCEs are generally well conserved across insect species.

#### *S12.3.1 Methods*

The identification of *Spodoptera frugiperda* CCEs was performed using two different methods. The first one concerned non-catalytic esterases and antennal esterases, the second one concerned all the remaining esterases. For the class 3 non-catalytic esterases (clades 27 to 32), protein sequences previously identified in the *Bombyx mori* and *Apis mellifera* genomes served as queries to search the *S. frugiperda* genome using tBLASTn (directly on the SfruDB website or using Galaxy). The same approach was then used to identify the orthologous of the 30 sequences identified in *Spodoptera littoralis* antennae [142]. *S. littoralis* amino-acid sequences were used as queries. To identify all the other esterases in the *S. frugiperda* genome, we developed a complementary method using a dataset composed of the 39 sequences identified in the *Helicoverpa armigera* transcriptome as a query in the Galaxy workflow developed and shared by the Olfaction/Chemosensory annotation group. Then all the scaffolds that were in the BLAST result list were manually observed and inserted/corrected in the user-created annotations section of Apollo.

To classify the CCE of *S. frugiperda*, we performed phylogenetic analysis together with sequences from *B. mori*, *H. armigera* and *S. littoralis*. Sequences were aligned using ClustalW [143] and a Neighbor-joining tree was then constructed with MEGA v.6 [123]. Names were given according to two previous studies [141,144].

#### *S12.3.2 Number, phylogeny*

A total of 96 CCE genes were annotated in the genome of *S. frugiperda* corn and rice variants (Table S17). This number is higher than that in the *B. mori* genome by 24 [145]. Exceptional recent duplications were observed in the clades 001 and 016. This result is in agreement with the transcriptomic analysis done with another noctuid species, *H. armigera* [141].

All homologs of *S. littoralis* antennal esterases were identified from a phylogenetic analysis (Fig. S16), except two members of clade 001: CXE7, which in *S. littoralis* is able to degrade the pheromone *in vitro* [146] and CXE29. Only clade 009 is not represented in *S. frugiperda*. Our study also revealed gene alternative transcription. CXE4 and CXE14 are two transcripts of the same gene and CXE8 could also produces two alternative transcripts. The organization of *S. frugiperda* CCEs is very specific. 71 of *S. frugiperda* CCEs are organized in tandem or clusters.

#### *S12.3.3 Comparison with rice strain*

Six CCEs identified in the corn variant genome, CXE012a, CXE25 (clade 013), CXE16 and CXE24 (clade 024), and CXE025a, were absent in rice variant genome. Two CCEs were only present in the rice variant genome: CCE001q, located between CXE28 and CCE001m (Fig. S17), and CXE15 (clade 020). The latter could be detected by PCR amplification with specific primers from genomic DNA of both strain, and its specificity for R strain could not be confirmed (Supplementary Note S12.6 and Fig. S21). Amino-acid substitutions were

identified in most clades, as well as insertions and deletions. This is especially the case in the very large clade 001. This is illustrated in Supplementary Excel Table4.

### S12.4 UDP-glycosyltransferases

UDP-glycosyltransferases (UGTs) catalyze the conjugation of a range of diverse small hydrophobic compounds with sugars to produce water-soluble glycosides, playing an important role in the detoxification of xenobiotics and in the regulation of endobiotics [147]. Insect UGT enzyme activity has been investigated in the housefly *Musca domestica* [148], the fruitfly *Drosophila melanogaster* [149], the tobacco hornworm *Manduca sexta* [150], the silkworm *Bombyx mori* [151], and other insects [152], revealing that the insect UGTs play an important role in the detoxification and sequestration of a variety of plant allelochemicals and insecticides [153-157]. Enzyme activities of the insect UGTs are detected mostly in the fat body, midgut and other tissues [152], but also expressed in the antenna of *D. melanogaster* [158,159] and *Spodoptera littoralis* [160]. In addition, many endogenous compounds, like ecdysteroid hormones [161] and cuticle tanning precursors [162,163] are glycosylated by UGT enzymes. Furthermore, dietary flavonoids have been shown to be sequestered as glucose conjugates to impart color to the wings in a lycaenid butterfly [164] or in *B. mori* to be glycosylated to produce a green color in the cocoon with UV-shielding properties [154]. A UGT enzyme was recently shown to catalyze the final step in synthesis of cyanogenic glucosides by the Burnet moth *Zygaena filipendulae* [165]. These findings suggest multiple roles of the insect UGT enzymes in detoxification, olfaction, endobiotic modulation, and sequestration.

*Spodoptera frugiperda*, in particular, is known to glucosylate MBOA (6-methoxy-2-benzoxazolinone) and excrete into the frass as an N-glycoside. When fed on MBOA-containing artificial diet, *S. frugiperda* excreted a high amount of MBOA-N-glucoside. In vitro assays showed that MBOA-N-Glc is formed enzymatically in the insect gut using MBOA as a substrate [166]. A recent study revealed that a benzoxazinoid, (2R)-DIMBOA-Glc, which is hydrolyzed into the toxic DIMBOA by plant glucosidase upon herbivory, is reglucosylated by the insect to produce an epimeric glucoside, (2S)-DIMBOA-Glc, which is no longer active towards plant glucosidases, suggesting that such a stereoselective reglucosylation might contribute the successful pest status of the *Spodoptera* species on benzoxazinoid-containing crops [167].

#### *S12.4.1 Methods*

The UGT genes of *Spodoptera frugiperda* were identified and classified according to method described [168]

#### *S12.4.2 Number*

*Spodoptera frugiperda genome* contains a total of 48 putative UGT genes. This is the similar number found in other lepidopteran insects, *Bombyx mori* (45 genes), Manduca sexta (44 genes), *Heliconius melpomene* (52 genes), and in a beetle, *Tribolium castaneum* (43 genes), but it is a relatively large number compared to dipteran and hymenopteran insects (Table 1, main text)

#### *S12.4.3 Phylogeny*

A consensus Maximum-likelihood tree constructed with deduced amino acid sequences from the *S. frugiperda* rice strain and *B. mori* UGTs revealed patterns of inter-specific conservation and lineage-specific expansion of the gene family (Fig. S18).

Among others, UGT33 and UGT40 families comprise the largest two ones with 17 genes and 16 genes, respectively, accounting for 69% in total. The UGT33 family of *S. frugiperda* shows a lineage-specific gene diversification which might happen very recently from a possible ancestor family, UGT34, also composed of 4 exons as UGT33 genes. The UGT40 is also a highly diverged gene family clustered with UGT48 and UGT41. The former consists of

8 exons, the same exon number of the mother family UGT40, whereas the latter contains 9 exons, suggesting an additional intron gain at the last exon of its ancestral gene. The rest of UGT families might have been diversified at earlier time and then have remained without duplication for a long time, resulting in mostly single- or two-gene families. Their microsynteny shows the highly conserved genomic position and orientation, suggesting they might play fundamental roles at least in Lepidoptera (Fig. S19A). On the other hand, the most diverged two families (UGT33 and UGT40) do not seem to be as conserved as the others, although these genomic locations are nearby between two species (Fig. S19B), suggesting the lineage-specific gene duplications in tandem in these loci might occur independently after Bombycidae and Noctuidae had been diverged.

#### *S12.4.4 Comparison between corn and rice strain*

UGTs of the corn and rice strains were different in terms of amino acid sequence identity (Fig. S20).

The difference in protein sequence ranges from 0 – 8%; Sfru-UGT33-3, Sfru-UGT33-08, Sfru-UGT40-14, and Sfru-UGT40-03 shows differences higher than 5%. It is noteworthy that the members of UGT33 and UGT40 families show relatively higher sequence discrepancy between two strains, whereas the rest of UGT families, which are to be conserved across species, show higher similarity between strains. Another difference between two strains is the loss or gain of UGT gene in a certain strain. Sfru-UGT40-06 of the corn strain was not identified in the rice strain, whereas Sfru-UGT33-17 of the rice strain was not found in the corn strain (Fig. S19B). In addition, Sfru-UGT33-04 in the rice strain seems to be multiplied by domain (exon1) duplication, which translates substrate binding domain, probably resulting in an increased range of substrates.

### S12.5 ATP-binding cassette transporters genes

ABC (ATP-binding cassette) transporters constitute one of the most abundant protein families in all organisms. These transmembrane proteins hydrolyze ATP in order to conduct transport and other cellular processes [169]. A functional transporter consists of four core domains: two nucleotide-binding domains with seven conserved motifs alternating with two transmembrane domains. Although a total of eight subclasses (A-H) with different functions have been identified in insects, only two of those, the ABC-B and ABC-C, are known to be involved in multidrug resistance mechanisms [170].

#### *S12.5.1 Method*

As query for the ABC transporter genes we used the genome information from *Bombyx mori* and *Manduca sexta*. The SfruDB was searched by using tblastn (default parameters) and the corresponding transcripts were annotated in WebApollo. The exon-intron structure was corrected based on homology. The transmembrane topology of all genes was verified using Phobius (http://phobius.sbc.su.se/). The nomenclature for each subfamily was used according to the orthologs in *Bombyx mori* and *Manduca sexta*.

#### *S12.5.2 Results*

We have identified and annotated 8 genes encoding ABC transporters of subfamily B and 10 genes for subfamily C. Each B and C subfamily gene has an ortholog in *Bombyx mori* [171], [172] and *Manduca sexta*, but *Bombyx* has an additional B gene not present in *Spodoptera*. One gene (ABC-C5) has an alternate splice form which was annotated as a separate transcript (ABC-C5.2). ABC-C2 and ABC-C7 could not be distinguished from each other. Except for three genes of the B subfamily, all were fragmented among different scaffolds. Three genes had one or more exons missing from the scaffold (ABC-B8, ABC-B5 and ABC-C10). The

transmembrane topology of all genes was verified using Phobius (http://phobius.sbc.su.se/). The nomenclature for each subfamily was used according to the the orthologs in *Bombyx mori* and *Manduca sexta*.

### S12.6 Experimental validation of some interstrain differences in detoxification genes repertoire

Since some missing genes in one strain could result from differences in the genome assemblies, we performed PCR validations for some of them, when we could design primers specific to the missing paralogs.

#### *S12.6.1 Method*

We amplified by PCR the following genes *SfCYP340L10*, *SfCYP6AE86*, *SfCYP6AE87*, *SfCXE15, SfUGT33-17*, expected to be specific of R strain, *Sf UGT40-06*, expected to be specific of C strain and *SfGST8,* which was found in both strains as positive control. We used as template genomic DNA extracted from one male of the C or the R strains and the following primer pairs.

| | |
|---|---|
| SfCYP340L10F | 5′-GAAGTACGCCATGATGACCTTG-3′ |
| SfCYP340L10R | 5′-CCATCAAACATACTCGATCTG-3′ |
| SfCYP6AE86F | 5′-GTCTTTAATAGTTAACGTTTGAC-3′ |
| SfCYP6AE86R | 5′-CACCATGGTTATATTACTTCTGGTG-3′ |
| SfCYP6AE87F | 5′-GACGAGAATCAGTAGCGTTATTG-3′ |
| SfCYP6AE87R | 5′-CGTTAACTATTAAAGACTCTTAC-3′ |
| SfCXE15F | 5′-TTCGCTGAACACTCCCAAGATACC-3′ |
| SfCXE15F | 5′-TTCCCTCGACCTTGCTCTATGAGT-3′ |
| SfGST8F | 5′-TTGAAGGCATGTGGGGCTC-3′ |
| SfGST8R | 5′-TCGAGAAAGTGGAAATGTCAATTT-3′ |
| SfUGT33-17F | 5′-GTTCGTTTGGAGCTGTGTTCG |
| SfUGT33-17R | 5′ -TGGACTGAAACCCTAAGTCTTGT |
| SfUGT40-06F1 | 5′ - GGCCATGCCTCGATTTTTCG |
| SfUGT40-06F2 | 5′ -AAGCATGGCAGTCATACCAA |
| SfUGT40-06R | 5′ -ACTGATTCTTGTAGTCTCGTCCA |

#### *S12.6.2 Result*

We could confirm the R strain specificity of *SfCYP340L10*, *SfCYP6AE8*, *UGT33-17* and the C strain specificity of *UGT40-06* (Fig. S21). The other genes *SfCYP6AE87* and *SfCXE15* were detected by PCR in both strains suggesting that they were missing in the assemblies in one of the other strains.

## S13. Digestion genes

Proteases can be classified into five main classes based on their catalytic mechanisms; proteases that have an activated cysteine residue (cysteine proteases), an aspartate (aspartate proteases), a metal ion (metalloproteases), a threonine (threonine proteases), and proteases with an active serine (serine proteases)[173]. Over one third of all known proteolytic enzymes are serine proteases [174]. Digestive proteases are one of the most abundant and essential protease enzymes necessary for metabolism of insects. In lepidopteran insects serine proteases carry out about 95 % protein digestion [175]. Serine proteases are produced by the midgut epithelial cells and secreted into lumen. Proteases carry out hydrolysis of peptide bond in proteins, generating peptides and then to amino acids. Insects need amino acids as essential

building blocks for the insect's structural and functional components. These components are involved in development and physiology [176,177].

Digestive serine proteases are of trypsin and chymotrypsin type having same mechanistic class but different substrate specificity. Trypsins are specific for hydrolysis of peptide bonds adjacent to basic amino acids arginine or lysine, while chymotrypsins are specific for aromatic or bulky non-polar amino acid such as tryptophan, phenylalanine, or tyrosine. The active center of serine proteases composed of His57, Asp102, and Ser195, which are responsible for the acyl transfer mechanism of catalysis. The plant protease inhibitors are induced in response to herbivory and inhibit insect's serine proteases. To cope with the protease inhibitors, it seems that the insects have acquired large multigene families of serine protease [178-182]. Thus, our goal is understanding characteristics, evolution and how serine protease gene family is expanding in different insects, in response to feeding on different host plants.

### S13.1 Methods

The serine proteases from *S. frugiperda* (corn and rice strain) were identified and annotated by using previously annotated serine proteases from *H. armigera*. Genomic and transcript databases of *S. frugiperda* genome consortium FAW-IPC (Fall Armyworm International Public Consortium) were used. Blastn and tBlastn searches using *Helicoverpa armigera* serine proteases [183] against the genomic database of *S. frugiperda* were performed. Individual contigs containing protease genes were screened manually. The collected contigs containing serine protease genes were assembled in Sequencher 4.7 (Gene Codes Corporation, Ann Arbor, MI, USA). Each assembly representing a single gene was arbitrarily named and the assembly work was continued until no more contigs were left. The 5'- or 3'-UTR sequences were assembled with flanking genomic contigs and extended as long as possible in order to identify neighboring genes. For genes without cDNA support from transcript database of FAW-IPC, transcript databases in MPI-CE private library was used.

### S13.2 Number, phylogeny, between strain comparison

According to our survey of *S. frugiperda* genomic databases, there are 86 and 113 digestive serine proteases in Corn and Rice strains, respectively. Protease sequences are highly conserved at their catalytic residues, H57, D102, S195 and N-terminal signature sequence. All the digestive serine proteases belong to S1 family. Phylogenetic analysis inferred using the neighbor-joining method revealed eleven sub-groups (Trypsin; Chymotrypsin 1, 2, 3 4; Chymotrypsin like proteases; Diverged serine proteases 1, 2, 3, 4; and azurocidine) for this gene family (Fig. S22). Most of the genes in the sub-groups also follow the conserved intron-exon structure. The numbers of proteases are expanding rapidly by gene duplication and divergence. Majority of the genes are present in clusters, most likes formed by lineage specific gene duplication. The largest gene clusters of serine proteases are chymotrypsin type 1having 9 genes on scaffold_448 in corn strain and 7 genes on scaffold SFRU_RICE_002652 in Rice strain. All the subfamilies of serine proteases have true orthologs in both the strains (Fig. S22). Further comparison of digestive proteases of *Spodoptera frugiperda* with other lepidopteran species can be found in Kuwar *et al* , in prep.

## S14. Immunity genes

The invertebrate immune response has been extensively studied in insects such as the insect model, *Drosophila melanogaster*, and also more recently in several lepidopteran, i. e. *Bombyx mori* [184], *Manduca sexta* [185,186] and *Galleria mellonella* [187] as well as in the hymenoptera, *Apis mellifera* [188]. The most integrated understanding of this physiological function comes from studies performed on Drosophila. Indeed, biochemical, molecular biology and genetics approaches have led to the characterization of the molecular mechanisms involved in (i)

pathogen recognition and extra-cellular signaling, (ii) signal transduction through intra-cellular signaling pathways, and (iii) pathogen elimination through the production of effectors molecules and cell activation (for review see [189,190])

### S14.1 Methods

In order to annotate immune-related genes, transcripts already identified in our reference transcriptome TR2012b [16] were used. In absence of such transcripts, DNA sequences from *Danaus plexippus*, *Spodoptera* sp, *Bombyx mori* or *Manduca sexta* were used as query and blastn were performed. Exon/Intron junctions were manually corrected whenever necessary. Deduced protein sequences of the annotated genes were checked using blastp on nr database at NCBI.

### S14.2 Results

A total of 163 immune genes were found in the genome of *S. frugiperda* (Table S18). However, 216 members of OGS2.2 were annotated since 56 of the immune genes were encoded on more than 2 scaffolds. 164 OGS2.2 genes were supported by the presence of one or more transcripts in TR2012b (Supplementary Excel Table5, Immunity tab, Panel B).

All members of the major signaling pathways, Toll, imd, Jak/STAT and JNK, are present in *Spodoptera frugiperda* genome but Grass, Dif and Udp3. Grass is a serine protease which belongs to a large family of CLIP domain containing proteases and is involved in the activation of spätzle processing enzyme which then cleaves pro-spätzle to generate spätzle, the natural ligand of Toll receptor. In Sf_TR2012b, we identified 16 such proteases while 15 and 37 were found in the genomes of *B. mori* and *D. melanogaster*, respectively. Therefore, even though Grass might be one of them, we were not able to identify it with certainty. The second one is the Dorsal-related immunity factor, Dif, and the third missing component is the cytokine Upd3, an activator of the JAK/STAT pathway. To our knowledge, these two genes were characterized only in *Diptera*.

A comparison of immune-related genes found in *S. frugiperda* genome with those present in the genomes of *B. mori*, *D. melanogaster*, *A. gambiae* and *A. mellifera* is shown in Table S19. It appears that *S. frugiperda* has a comparable number of immune genes with the other insects with the exception of *A. mellifera* which has a reduced immune repertoire as previously reported [188]. On the other hand, lepidopteran, *S. frugiperda* and *B. mori*, have a higher number of effector genes likely due to the presence of lepidopteran-specific genes encoding antimicrobial peptides such as gloverins, lebocin and moricins.

Finally, a selection of families of genes encoding proteins involved in the recognition of pathogen associated molecular pattern (GNBP for Gram negative binding proteins and PGRP for Peptidoglycan recognition proteins) or immune effectors such as antimicrobial peptides (Cecropins, Attacins and Lysozymes) was used to analyzed putative differences between corn and rice *Spodoptera* variants (Supplementary Excel Table5, Immunity tab, Panel B). All searched genes were present in both variants. In addition, in some cases, *i.e.* GNBP1, GNBP2, the rice variant genome was very useful in the establishment of the complete sequence of the genes which were fractionated on at least two scaffolds in the corn variant. On the contrary, corn variant allowed the annotation of the full sequences of the two phenoloxidases whose sequences in the rice variant spanned on 2 scaffolds (Fig. S24).

## S15. RNA interference genes

RNA interference (RNAi) and related RNA silencing phenomena use short antisense guide RNA molecules to repress the expression of target genes. RNA silencing is mediated by the effector complex RNA induced silencing complex (RISC) and RNA induced transcriptional silencing (RITS), functioning in post transcriptional and translational silencing respectively

[191]. PIWI-Argonaute genes are part of the RNAi pathway and involved in producing double-stranded RNA (dsRNA) and in degradation of messenger RNA (mRNA). Argonaute proteins, comprising the Ago and Piwi subfamilies, are the only proteins common to these complexes. In Lepidoptera, the presence of RNAi was also reported [192]. The key genes involved in RNAi are evolutionarily conserved and play a major role for host defense against viruses [193]. In subsequent years it was shown that RNAi in Lepidoptera is not as straight-forward and effective as it has been shown for a number of non-Lepidopteran insects [194]. Besides its role in viral defense, RNAi also plays a major role in different biological pathways like epigenetic regulation and heterochromatin formation [195,196].

### S15.1 Methods

As query for Ago1, Ago2a, Ago2b, Dcr1, Dcr2 Aubergine/PIWI, Paha and Loquacious we used available genome information from *Bombyx mori*, *Manduca sexta*, *Spodoptera litura* and DNA sequences obtained in our laboratory from *Helicoverpa armigera*. We searched in the SfruDB by tblastn and annotated the corresponding transcripts in WebApollo. We corrected the exon/intron structure based on homology. Most of the genes were found to be spread over more than one scaffold.

### S15.2 Results

In the *Spodoptera frugiperda* genome we identified genes belonging to the RNase III family of ribonucleases, so called Dicer 1 (Dcr1 involved in miRNA pathway) and Dicer 2 (Dcr2 involved in viral RNAi or long dsRNA processing) that cleaves long dsRNA precursors into products around 21 – 23 nucleotides long. We were also able to identify Argonaute genes, namely Argonaute1 (Ago1), Argonaute2a (Ago2a) and Argonaute2b (Ago2b). AGO3 was not found during the analysis of TR2012b, our reference transcriptome [16]. However, two predicted genes, GSSPFT00018695001 and GSSPFT00031765001, were found to group together in a phylogenetic tree (Fig. S25) with AGO3 from *Bombyx mori* and *Danaus plexippus*.
These genes encode the RNaseH family which are key components of RISC or the microRNA ribonucleoprotein complex (miRNP), which causes mRNA-cleavage or transcriptional silencing [197]. The genes of Aubergine/PIWI, Pasha and Loquacious were also found in the *Spodoptera frugiperda* genome. The coding sequence from a dsRNA binding protein called R2D2 was missing in the *S. frugiperda* genome database and transcript database.

## S16. Homeodomain (HD) genes

The Homeodomain (HD) is a DNA binding domain that is conserved across phyla of metazoa [198]. Proteins with that domain are regulatory Transcription Factors (TF) that are often involved in crucial steps of development and cell differentiation. The best known HD proteins are the homeotic (Hox) proteins that specify the segmental identity along the antero-posterior axis of metameric animals. The HD is typically composed of 60 amino-acids forming three alpha helices arranging themselves in a globular domain that can contact the major groove of DNA in a sequence specific manner. Paralogous HD proteins within a single organism share a lot of analogous amino-acid positions. But interestingly, orthologous proteins between organisms share even more amino-acid conservation. This feature makes it an easy set of benchmark proteins to assess the completeness of a genome assembly. Here, we present a complete manual curation of full length HD proteins in *Spodoptera frugiperda* (*Sf*) genomes, based on 108 *Drosophila melanogaster* (*Dm*) HD sequences and *Bombyx mori* (*Bm*) annotations. Having both the C strain and the R strain genome assemblies allowed the replication of the annotation and thus confirmation of full length sequences in not all, but most cases. We confirmed previously described Lepidoptera-specific particularities and

propose some corrections in *Bm* annotation. No notable difference was detected between *Sf* strains, or between *Sf* and *Bm* except for the Special Homeobox (Shx) family.

## S16.1 Methods

### *S16.1.1 Identification of S. frugiperda HD proteins*

HD peptide sequences for *Drosophila melanogaster* were downloaded from HomeoDB[2] (http://homeodb.zoo.ox.ac.uk/) [199,200]. For each HD sequence, we searched by BLASTP, the nr database restricted to *Bombyx mori*. Usually, we could retrieve a *Bm* protein sequence based on >80% identity of their HD, but most of the cases the percentage of identity was closer to 95%. In cases we could not retrieve a *Bm* target with this level of identity, we extended our search to all Lepidoptera in nr. In all cases, we could find Lepidoptera orthologs that were not documented in *Bm*. We then used the Full Length *Bm* (or alternative Lepidoptera - often *Amyelois transitella*) sequence to perform a tBLASTn against the *Spodoptera frugiperda* Corn (*SfC*) strain and Rice strain (*SfR*) genome assemblies. Based on this alignment, gene models were retrieved and manually annotated in WebApollo on SfruDB (http://www6.inra.fr/lepidodb/SfruDB).

Finally, *Dm* HD sequences were used for a direct tblastn against *SfC* genome to retrieve all instances of matches, in case a diverged instance of HD with no full length homology with *Dm* or *Bm* exist in *Sf*. All matches for all HD were summarized into a single list of all putative HDs in *Sf* and compared to the manual annotation. This allowed to retrieve HD of the *Shx* family, specific to Lepidoptera (see Results).

### *S16.1.2 Manual curation*

Manual curation of gene models has been performed based on three lines of evidence, in order of priority.

1/ RNA evidence: If the gene is expressed, the RNAseq tracks on the JBrowse interface of WebApollo help curate the exon intron junctions of the gene. If there is a clear evidence for alternative transcripts, they are annotated, but otherwise, no particular effort was made to search for different isoforms. If an EST is present (track TR2012-b from [16]), we used it to confirm the exon-intron structures and most of the time the length of the 5'- and 3' UTRs.

2/ homology. If there is evidence for an exon homologous and contiguous to the rest of the protein when compared to *Bm*, this homology is used to create an exon, even in absence of RNA evidence.

3/ prediction. If only conserved domains homology is present, and no RNA support, we trusted the automatic annotation of genomes to support the gene models.

Finally, this manual curation was revised based on clustal alignments [201], [143] of the retrieved *Bm*, *SfC* and *SfR* full length amino acid sequences, using *Bm* as the best support for the accuracy of protein sequences and under the hypothesis that both *Sf* strains should contain the same protein sequences, as for this time of divergence, only a few amino-acid substitutions are expected. In clear, we wanted both strains to have the same peptide sequence based on the gene model and as close as possible to *Bm*. In particular, unless supported by strong RNA evidence, we are being conservative and do not allow extra unsupported protein sequence in *Sf*. We can provide upon request a Fasta file that contains the Full Length Protein sequence for all *Dm* HD proteins, grouped with all retrieved *Bm* (or alternate Lepidoptera) HD proteins, and all orthologous HD proteins annotated in *SfC* and *SfR* genomes. When *Sf* duplications were observed, we checked by blastn whether the 2 scaffolds that contained them were allelic, and we retained only one representative sequence of both alleles.

### *S16.1.3 Annotation*

To name the *Sf* genes, we referred to the classification used in HomeoDB[2], keeping the Drosophila naming, unless not meaningful (such as CG11617 for example). In which case, we

kept the family name. To make sure the genes were belonging to the right families, we used all aligned HD sequences from *Dm*, *Bm*, *SfC* and *SfR* to reconstruct a phylogenetic tree, using PhyML [76]. The tree obtained (Fig. S26) allowed us to assign each *Sf* gene to the right family and use this information for the naming of the genes in the annotation process. A summary of this annotation can be found in Supplementary Excel Table5.

## S16.2 Results

We provide a complete set of HD in *Spodoptera frugiperda* based on *Drosophila melanogaster* complete list [199,200] and the benchmark *Bombyx mori* sequences retrieved in nr. First and foremost, this annotation work made us refine the lepidopteran HD annotations and propose some corrections for *Bm* naming. We would like to extend this work by providing with our annotation a resource for re-annotation and naming of newly sequenced as well as pioneer Lepidoptera genomes. In some cases (Supplementary Excel Table5, HD tab) we could not retrieve a *Bm* HD homolog, that was present in other Lepidoptera. The most parsimonious explanation would posit that these genes were not identified in the genome assemblies, however that should warrant further PCR-based confirmation.
Based on the phylogenetic tree (Fig. S26), we identified most of orthologous gene groups between *D. melanogaster*, *B. mori*, and two strains of *S. frugiperda*.
Paralog of HD are expected to be frequent [198]. However, we could find many instances where paralogs are observed only in Drosophila but not from Lepidoptera (Table S20).
On the contrary, some duplications occurred in Lepidoptera but not in Drosophila (Table S21).

**Cers family**. Ceramide synthase is a special class of HD proteins. They are transmembrane receptors with a HD in the sequence that is mostly not useful for their catalytic function [202]. The only Drosophila member of this family is *schlank*, also known as the *longevity assurance gene 1* (*Lag1*). However, homology for *Lag1* HD could be found for 2 *Bm* proteins in nr (*Cers5-like* and *Cer6-like*). In *Sf*, we could retrieve HD homologies for 4 different *Cers*. *Cers1* proteins were identified in both strains. We could not determine, in the phylogenetic analysis (Fig. S27), if it was a direct ortholog of *Cers5-like* or *Cers6-like* in Bombyx. Similarly for *Cers3*. We identified a third *Cers* (*Cers4/5*), resembling *Cers3* and *Bm_Cers5-like*. However, in *SfC*, this protein was only partial and lacking the HD. We verified by blastn that they did not represent cases of allelism compared to *Cers3*, but the scaffolds bearing those genes are dissimilar, strongly suggesting that *Cers3* and *Cers4/5* represent a *Spodoptera*-specific duplication. Finally, we identified in both strains a protein carrying a truncated version of the Cers HD, we called it *Cers2*.

**ZF family**. *Zfh1* and *Zfh2* are both present in Drosophila and Lepidoptera. But *zfh2* has 4 HD in Lepidoptera while only 3 in Drosophila.

**PRD family**. This family has 3 members in Drosophila : *prd*, *gsb* and *gsbn*. In Lepidoptera, no ortholog for *prd* itself has been detected. Since their name indicate a function which we don't know if it has been conserved in Lepidoptera, we named the 2 other paralogs of this family: *gsb1* and *gsb2* instead of *gsb* and *gsbn*. Similarly to Drosophila, they are arranged in cluster within the *Sf* genome. Homology with HD only retrieved one *Bombyx* Gsb protein instead of 2. This Bombyx gsb-like protein is most similar to *gsb2*. When we searched again nr with the full-length *gsb1* protein, we retrieved a partial protein in *Bombyx*, lacking the HD probably due to genome annotation error. Other Lepidoptera also have 2 *gsb* proteins.

**Tgif family**. There are 2 members of the Tgif family in Drosophila: *achi* and *vis*. We could also detect 2 members in *Bm* and *Sf*. However their duplication events seem all to be independent. We propose that *Tgif2* is the ortholog of *Dm-achi*, *Dm-vis* and *Bm-vis* and *Tgif1* is ortholog to *Bm-achi*, these 2 members representing a Lepidoptera specific divergence.

**The Irx family**. This famous cluster of 3 members in Drosophila contains *ara*, *caup* and *mirr*. There are only 2 members detected in Lepidoptera, but the pattern of duplication suggested by the phylogenetic tree seems to be different in *Bombyx* and in *Spodoptera*, with the *ara/caup* duplication present in *Dm* and *Bm*, but a duplication in *Spodoptera*, closely resembling *mirr* not present or lost in *Bm* (Fig. S28).

**Hox3 family**: In Drosophila, this family comprises *bicoid* (*bcd*), *zercknüllt* (*zen*) and *zercknüllt-2* (*zen2*). We could retrieve homologs for *zen* in *Bm* and *Sf*. But not for *bcd* and *zen2*, suggesting that this duplication never occurred in Lepidoptera. But closer inspection of the HOXL family phylogeny shows that *Dm-zen* (in yellow on Fig. S29) and Lepidoptera *zen* (in green on Fig. S29) are not on the same clade, with *bcd* (in red on Fig. S29) being an outgroup. This is in agreement with previously published phylogeny of Lepidoptera HOXL family [203; 88]. The Hox3 naming of this family comes from their conserved position within the Hox cluster between the 2 homeotic genes *proboscipedia* (*pb* - *Hox2*) and *Deformed* (*Dfd* - *Hox4*). Based on this we propose to rename the Lep *zen* homologs in *Hox3*, because they are also located in a similar position within the Hox cluster.

**The Shx family**: Finally, we could retrieve a family of HD in Lepidoptera, that is not present in Drosophila, and that are called *Shx* for Special Homeobox. They belong to the HOXL family, based on the phylogeny but also based on their location within the Hox cluster. This *Shx* family has been identified first in *Bombyx* and recently compared across different Lepidoptera genome, with 4 major classes emerging (A, B, C and D) and *Bombyx* having a huge expansion of 12x the ShxA family and no member of the ShxD family [204; 203]. The homology and the colinearity of this family was really difficult to assess in *Spodoptera*. In particular, there were some partial sequences and allelic homologies present. But based on scaffold localizations (Fig. S29), phylogenetic tree of Irx proteins, (Fig. S28) and the phylogenetic tree of all HOXL members in Lepidoptera (Fig. S26), we could retrieve 3 *S. frugiperda* members of the ShxA subfamily -one of them (ShxA3) is unusual since it seems to contain 2 HD-, 2 members of the ShXB subfamily and no member of the C or D subfamilies. Surprisingly, we found one Shx member that is unique to *Spodoptera*. Based on its position within the Hox cluster and on the phylogenetic tree, we propose that it is a new subfamily, named E, making it yet another variation of this Lepidoptera specific HD proteins family.

We did not detect differences in gene content between SfC and SfR HD families. One exception is the gene *bsh* that is not present in SfC. This is most likely due to sequencing assembly. Indeed, when we align genomic Illumina reads of SfC on this gene we find a high coverage, indicating that indeed, The SfC strains contains this gene. The same thing is true for *ShxA2* and *ShxA3* that are not annotated in the SfC assembly but can still be confirmed present in this strain based on coverage of the SfR counterpart.

## S17. Centromere protein genes

The holocentric structure of Lepidopteran chromosomes ([205] for review) prompted us to look for genes encoding kinetochore components as putative markers to uncover centromeres location.

### S17.1 Methods

The so called constitutive centromere-associated network (CCAN) proteins have been identified at first in humans. Homologues of these proteins can be identified in yeasts however only few of them (only CENP-A and CENP-C homogues) have been identified in *D. melanogaster* and *C. elegans* ( [206]). For that reason, we were obliged to use mainly human proteins sequences as queries in homology search. Performing BLASTP against the S. frugiperda proteome OGS was more sensitive than TBLASTN against the whole genome assembly to detect homologues. *S. frugiperda* protein sequences identified as kinetochore component candidates were also blasted against NCBI in order to confirm their relationship with centromeric proteins. When Drosophila proteins homologs existed, they were used as queries in homology search against the whole genome assembly using TBLASTN. Presence of functional domains characterizing the protein queries was checked in the *S. frugiperda* centromeric candidates as well as existence of transcripts validating the gene predictions and conservation of the genes in other Lepidoptera like *Danaus plexippus* or *Bombyx mori*. In order to identify a putative CENP-A homologue, the histone fold domain was taken as query in BLAST search. All histone fold domain containing protein predictions were analyzed one by one in order to identify putative candidates with an N-terminal extension and amino-acid variation in loop1.

### S17.2 Results

A ubiquitous centromeric marker is the histone H3-like protein CENP-A [206]. No homolog of CENP-A could be found in *S. frugiperda* genome nor in ESTs [207]. Homologs of proteins known to interact with CENP-A like CENP-C and CENP-N, were absent as well. The histone H3 variant CENP-A is specifically deposited at the centromere by a conserved chaperone, called HJURP or Smc3, in vertebrate and fungi. Homologs of this protein have not been found in Drosophila, *C. elegans* or plant, nor in *Spodoptera*. A functional homolog of HJURP has been identified in Drosophila called CAL1, which could not be identified in *Spodoptera* genome, either. KLN-2, specifically required for CENP-A recruitment was not found. In absence of candidate gene for CENP-A, we searched for other genes encoding proteins involved in centromeric chromatin organization (Supplementary Excel Table5, Kinetochore Tab). Identification of genes containing the histone fold domain led to discovery of homologs of CENP-S and CENP-X, but of absence of CENP-T and CENP-W. CENP-S, X, T, W, are four histone fold containing proteins, CENP-S and X are able to interact as dimers, as well as CENP-T and W, and tetramer formation of CENP-T-W-S-X is essential for functional kinetochore formation in vertebrate cells [208]. CENP-S-X are conserved kinetochore localized proteins but have also been identified as Fanconia Anemia M associated proteins binding to DNA damage sites. CENP-A is located at the centromere but also at sites of DNA breakage [209] as well. Homologs of CENP-L, M I, J in addition to Ndc80, Spc25 could be identified as candidates for inner and outer kinetochore proteins, respectively. Absence or presence of kinetochore gene candidates has been corroborated by their similar representation in *Danaus plexippus* and the silkworm genomes.

## S18. Circadian rhythm genes

Circadian clocks are endogenous mechanisms involved in important animal physiological and behavioral processes such as mating and feeding and growth process such as cell-division [210,211]. Key genes involved in circadian clock are well characterized in number of model organisms and present a high level of sequence conservation among organisms. Homologs of most of the known Drosophila clock genes are found in Lepidoptera [212,213]. Clock genes are involved in a core negative transcriptional feedback loop and a second, interlocking feedback loop (reviewed e.g. in [214]).

### S18.1 Methods

In the GenBank database, DBT, TIM, CRY1, CRY2, CLK and PER proteins were first identified in the closely related species *Spodoptera exigua*. As for PDP1 and CYC no sequence was available from *S. exigua*, PDP1 was first identified in *Drosophila melanogaster* and CYC in *Danaus plexippus*. For *vri*, the DNA sequence obtained in our laboratory was used.

DBT, TIM, CRY1 and CRY2 homologs in *Spodoptera frugiperda* were searched in the SfruDB by tblastn using *S. exigua* total or partial CDS as query. In WebApollo, we annotated the corresponding transcript, if present, and corrected the exon/intron structure based on homology. We further corrected the gene structure, including 5' and 3' UTRs, based on RNAseq and TR2012b evidence of RNA.

For CLK, PER, CYC, VRI and PDP1, homologs were blasted in an RNAseq assembly from larval midguts of both strains (now available on SfruDB Web Apollo: name of string), using tblastn. The obtained cDNA sequences were blasted in SfruDB using blastn. In WebApollo, we annotated the corresponding transcripts and corrected the exon/intron structure and UTRs, based on RNAseq and TR2012b evidence. We carefully named the alleles and parts of all genes, if present. As a final check, we retrieved the created protein sequences and performed a blastp against insects on the NCBI blast server to confirm homology in other lepidopteran insects.

### S18.2 Results

All the critical clock genes –clock (clk), cycle (cyc), period (per), timeless (tim) and cryptochrome-type1 (cry1) – were found. As in the monarch butterfly genome (Danaus plexippus), in the S. frugiperda genome we found a type-2 vertebrate-like cryptochrome (cry2), which does not exist in D. melanogaster. We identified an ortholog encoding for Doubletime (dbt), which is involved in the posttranslational modifications of PER and TIM. We also identified major regulators of clk transcription: genes encoding orthologs of vrille (vri) and PAR domain protein 1 (PDP1). In per and cyc we found alternative splicing, i.e. in per exons 6 and 28 are present in ~ half of the RNA-seq reads mapped to the rice variant of the genome, exon 23 in ~ 70% and exon 22 in ~ 90% of the RNA-seq reads, while in cyc two alternative first exons exist. We annotated 4 different variants in per and 2 different variants in cyc. Comparing the two strains, in all genes we found SNPs, but only in clk, cyc and per we found two, two and one non-synonymous SNPs, respectively. In addition, in the corn strain we could not find exon 1 of clk and exon 6 and 13 of per, which were all present in the rice strain, but this is likely due to the fragmented genome of the corn strain. Whether any of these non-synonymous SNPs or exons are involved in the allochronic differentiation of the two strains remains to be determined.

## S19. Autophagy-related genes

Autophagy encompasses all catabolic mechanisms resulting in delivery of cellular components to the lysosomes for degradation. There are three main subtypes usually distinguished based on how cargo reaches the lysosome : microautophagy involved in the degradation of small portions of cytoplasm by invagination of the lysosomal membrane [215]; chaperone-mediated autophagy using specific proteins, including the protein Hsc70 (Heat shock cognate protein of 70 kDa), which target the misfolded proteins and transport them to the lysosome for degradation [216]; and macroautophagy, often referred to as autophagy, where damaged proteins and organelles are sequestered by a membrane called phagophore which elongates and closes to form a characteristic double-membrane structure called the autophagosome. This vesicle subsequently fuses with lysosomes in which cellular material is degraded and recycled [217].

Autophagy is generally activated under conditions of stress, including nutrient deprivation, hypoxia and infection. It allows the recycling of materials and cell survival but is also a process of type II programmed cell death (PCD II) widely described during metamorphosis in holometabolous insects and embryonic development in mammals [218-221].
This biological process and factors that regulate this pathway are highly conserved among eukaryotes and have extensively studied in yeast in which at least 37 autophagy-related (Atg) proteins were identified [222,223]. Among them, half are essential for autophagy itself and most are conserved in mammals and insects. The core Atg proteins that are involved in the formation of the autophagosome can be divided into four subgroups: (1) Atg1 and their regulators; (2) the Vps34 complex; (3) the Atg9-dependent vesicular complex; and (4) the ubiquitin-like proteins Atg12 and Atg8 and their conjugation systems.

### S19.1 *Atg1* and their regulators

The serine/threonine kinase Atg1 complex (consisting of Atg1, Atg13, Atg17, Atg29 and Atg31 in yeast) initiates the formation of the autophagosomal membrane. In *Spodoptera frugiperda*, ATG1 and ATG13 genes have been identified. As for flies and yeast, only one copy of ATG1 has been found, whereas humans have two closely related homologs (Unc-51-like kinase: ULK1 and ULK2) that are functionally redundant in starvation-induced autophagy [224]. Atg17, Atg29 and Atg31 are present only in *S. cerevisiae* and closely related species, no homologs of these genes were found in mammals neither in insects, including *S. frugiperda*. By contrast, homologs of FIP200 (also known as RB1CC1) and Atg101 [225], two other subunits of the Atg1 complex are widely conserved in eukaryotes but not in *S.cerevisiae*. Although the function of FIP200 is speculated to be similar to that of Atg17, there is no apparent sequence similarity [226]. In *S. frugiperda*, two alleles of RB1CC1 and Atg101 were found. Therefore, the composition of the Atg1 complex in *S. frugiperda*, consisting of Atg1, Atg13, RB1CC1 and ATG101, is different from *S. cerevisiae* and conserved with the other eukaryotes.

### S19.2 the *Vps34* complex

The phosphatidylinositol 3-kinase (PI3K) Vps34 complex (consisting of Vps34, Vps15, Atg6 and Atg14 in yeast) mediates nucleation of the pre-autophagosomal membrane. In *S. frugiperda*, we have identified all members of this complex: the catalytic subunit of the complex, Vps34 (known as class 3 PI3K, PI3KC3, in mammals), the regulatory subunit of the complex, Vps15 (known as ird1 in *D. melanogaster* and PIK3R4 in mammals; two alleles) and Atg6 (Beclin-1 in mammals; two alleles) and Atg14. Mammalian Vps34 complexes contain additional proteins including UVRAG (UV radiation Resistance-Associated Gene protein), Bif-1 (also known as endophilin B1), Rubicon and Ambra1 (Activating Molecule in Beclin-1-Regulated Autophagy 1) [227], one of them, UVRAG, was found in *S. frugiperda*.

### S19.3 The Atg9-dependent vesicular complex and the ubiquitin-like proteins Atg12 and Atg8 and their conjugation systems

The Atg9-dependent vesicular complex and two ubiquitin-like proteins conjugation systems are involved in the elongation of the autophagosomal double membrane. The first Atg5-Atg12-Atg16 conjugation system proceeds through the action of the E1-like enzyme Atg7 and the E2-like enzyme Atg10 [223]. The second system conjugates the ubiquitin-like protein Atg8 (LC3 in mammals) to the lipid phosphatidylethanolamine (PE) to form Atg8-II through the actions of Atg3, Atg4 and Atg7. Atg8-II is then incorporated into the autophagosomal membrane and is used as a marker to quantify autophagosome formation [217]. All members involved in the elongation step have been identified from the SfruDB: the only autophagic transmembrane protein Atg9, Atg5, Atg12, Atg16, Atg7 (2 alleles), Atg10, Atg3 (2 alleles), Atg4 and Atg8. Additional genes including Atg2, Atg18 (human WIPI1 and WIPI2) and

sequestrome-1 (SQSTM1, also known as p62) were also identified. Whereas yeast has a single ATG8 gene, many other eukaryotes contain several genes. Atg8 proteins can be divided, by sequence similarities, into three subfamilies: microtubule-associated protein 1 light chain 3 (MAP1LC3 or LC3), γ-aminobutyric acid receptor-associated protein (GABARAP) and Golgi-associated ATPase enhancer of 16 kDa (GATE-16) [228]. Although genes from all three subfamilies are found in vertebrates, some invertebrate lineages have lost the genes from one or two subfamilies. In *S. frugiperda*, only one copy of ATG8 gene was identified, as for the other lepidopteran species [229]. Note that a much longer *ATG*8-like gene, containing an internal duplication of the ubiquitin-related domain, was found. Multiple alignments analysis of Atg8 ORFs from *S. frugiperda*, *B. mori*, *G. mellonella*, *H. armigera*, *P. xuthus*, and *D. plexippus* (Fig. S30) revealed that Atg8 proteins were highly conserved among Lepidoptera. Amino acid sequence of *S. frugiperda* Atg8 share 99 to 100% similarity with the others lepidopteran proteins and 99% similarity with homologs of *D. melanogaster* (Table S22). *S. frugiperda* Atg8 protein, like the other lepidopteran proteins, shares more sequence similarity with GABARAP subfamily (94% similarity with human GABARAP) than with LC3 subfamily (60% similarity with human LC3), it contains a typical glycine residue at position 18, an ubiquitin-related domain and an essential glycine residue at the C-terminus [229,230].

In both mammals and insects, autophagy is best studied for its role in nutrient homeostasis. In nutrient-rich conditions, class I PI3K signaling activates, via the serine/threonine kinase Akt/PKB, a protein kinase called TOR (target of rapamycin) which inhibits autophagy at the level of the Atg1 complex. In nutrient-poor conditions, TOR is inactivated and the repression of autophagy is relieved [223,231]. This nutrient responsive signaling cascade is highly conserved from yeast to insect and humans. In *S. frugiperda*, we have identified all members of the PI3K/Akt signaling. Two alleles coding for the catalytic subunit of the class I PI3K (PI3KC1) and one gene encoding Akt were identified. The protein sequence of these two members is well conserved: 87% sequence similarity with the PI3KC1 from B.mori and 60-68% with the PI3KC1 from the other insect orders; 94% sequence similarity with the Akt from B. mori, 78-80% and 73-74% with the Akt proteins from Diptera and Hymenoptera, respectively, 68-76% with the human proteins (Table S22). In addition to sequence conservation, we also found that the different domains of the PI3K (p85BD, RBD, C2, PIK, kinase domain; [232]) are conserved in the sequence of *S. frugiperda* and in all Lepidoptera PI3K proteins. In *S. frugiperda* Akt protein, the three PM, kinase and HM domains and the two phosphorylation sites S473 and T308 [233] are present. Whereas most eukaryotes have a single TOR gene, we have found two genes encoding TOR in *S. frugiperda*. In *B. mori*, two paralogous TOR genes with high sequence similarity, BmTOR1 and BmTOR2, have been also identified by Zhou and colleagues [234]. The genomic analysis revealed that BmTOR1 is the ortholog, while BmTOR2 is then derived after a duplication event. The two BmTOR genes have similar expression patterns and are transcriptionally regulated by starvation and injection of 20-hydroxyecdysone. Amino acid sequences of both copies of *S. frugiperda* TOR proteins have 79% sequence similarity and 62% sequence identity (Table S22). They share 91% and 94% sequence similarity with BmTOR1 and BmTOR2 genes, respectively, 67-75% with the TOR protein sequences of Diptera and 71-78% with those of Hymenoptera (Table S22). The four highly conserved domains of TOR, especially the binding domain of rapamycin (FRB) [234], are found in the *S. frugiperda* TOR sequences. The phosphorylation site in S2448 position of the H. sapiens TOR is found in one of the two copies of *S. frugiperda* sequences, suggesting different functions for the two TOR present in this organism.

Additional genes of the upstream regulatory pathways were also identified: the small GTPase Rheb (effector of Akt), Raptor and LST8 (associated proteins to the TOR complex).

Finally, some genes encoding proteins involved in selective autophagy-related processes were annotated: Peroxin 3 and Peroxin 14 involved in pexophagy (selective aotophagic degradation of peroxisomes); Arp2 and Arp3 involved in the cytoplasm to vacuole targeting (Cvt) pathway.
To conclude, 17 ATG genes and 4 core machinery genes were identified in *S. frugiperda* genome (Supplementary Excel Table5, Autophagy Tab). Moreover, not only the core autophagic machinery seems to be conserved, but also the upstream regulatory pathways, including the PI3K/Akt/TOR signaling pathway. The study of autophagy and its regulation in *S.frugiperda* could thus potentially be performed with the same tools (antibodies and inhibitors) as those used in other cellular systems. Even identified genes were supported by transcriptomic analysis in the reference Sf_TR2012b transcriptome [16], they need to be validated in silico by reverse-transcription PCR and functionally validated by RNAi studies. In lepidopteran cells, we recently validated the TOR pathway involved in autophagy [235].

## S20. Apoptosis-related genes

Caspase-mediated apoptotic cell death is the most studied form of programmed cell death, involved in many important regulatory mechanisms, including growth development, tissue homeostasis or immunological response. This process permits the elimination of unnecessary, damaged or infected cells. Apoptosis is activated by two canonical signaling pathways, that is the intrinsic pathway involving mitochondrial events and the extrinsic pathway, triggered by binding of extracellular ligands (FasL, TRAIL, TNF) to death receptors. In both apoptotic modes, activation of cysteinyl asparte-specific proteases, called caspases, is a crucial step and results in mitochondrial membrane permeabilization, chromatin condensation, nuclear fragmentation, cytoskeletal rearrangement and formation of apoptotic bodies, thereby leading ultimately to the destruction of the cell [236].
Apoptosis has been well studied using model organisms, including mammals and Diptera. However, the molecular mechanism of apoptosis is still poorly understood in Lepidoptera. A recent survey of apoptotis-related genes present in the silkworm genome concluded to the existence of the apoptotic pathways typically described in mammals [237].

Annotation of the *S. frugiperda* genome revealed a number of genes known to play key roles in extrinsic and intrinsic apoptosic pathways (Supplementary Excel Table5, Apoptosis Tab). Some of them were previously cloned. Among the main mediator of apoptosis, we have annotated five caspase-related genes, including two initiators caspases : the mammalian caspase 9 ortholog, SfDRONC [238] and the caspase-8 homolog Sf-DREDD and three effectors caspases: Sf-caspase-1 [239], Sf-caspase-3 and sf-caspase-4, although this latter is predicted to be catalytically inactive. We also identified a number of genes involved in the regulation of caspases, such as two members of the IAP (inhibitor of apoptosis) family, Sf-IAP [240] and Sf-IAP-2, and two others BIR domains proteins, Sf-survivin-1 and Sf-surviving-2.

### S20.1 Extrinsic pathway

In mammals, the death receptors involved in the extrinsic pathway belong to the tumor necrosis factor receptor (TNFR) gene family. Upon binding of their cognate ligands, death receptors aggregate and the adaptors proteins such as FADD (Fas-associated death domain) or TRADD (TNF-alpha associated death domain) and the initiator procaspases 8 are recruited. These successive events result in the formation of multiprotein death-inducing signaling complex (DISC) that activates the procaspases-8 and -10. At this step, the mode of function of caspase-8 diverges depending on the type of cells. In type I cells, caspase-8 is able to cleave and activates the effector procaspases-3 and -7, while in type II cells, caspase-8 cannot

activate caspases, but instead cleaves the pro-apoptotic Bcl-2 family member Bid, that results in the activation of the mitochondrial pathway.
No gene related to TNFR death receptors genes or wengen, the Drosophila ortholog, was found in the *S. frugiperda* genome. However, we identified other genes encoding proteins involved in this pathway, such as two members of the TNF ligand family (TNFSF5 and TNFSF13), and the gene encoding for the adaptor protein FADD. As mentioned above, the caspase-8 homolog DREDD is also present in *S. frugiperda*.

### S20.2 Intrinsic pathway

The intrinsic pathway is triggered by various signals and leads to the release of apoptosis-inducing factors, including cytochrome c, apoptosis-inducing factor (AIF), Endo-G and Smac/Diablo by mitochondria into the cytosol. Once released, cytochrome c binds to apoptotic proteinase-activating factor-1 (Apaf-1) to form the apoptosome. The procaspase-9 is recruited by this complex, thereby activating the enzyme, which in turn activates several effector caspases. Apoptosis requires direct activation of Bax and BAK at the mitochondria by a member the Bcl-2 homology domain-3 (BH3)-only family of proteins including Bid, Bim and PUMA.
We found several homologs of proteins involved in this pathway, notably proteins involved in the apoptosome formation, including Apaf-1, cyt c and Sf-Dronc. We also identified the IAP antagonists, Smac/Diablo and Sf-IBM1 (IAP-binding motif 1), a Drosophila Reaper ortholog, and the mitochondrial apoptogenic factors, AIF and EndoG. As for *B. mori*, we found only one member of the Bcl-2 family proteins, Sf-Buffy.
According to this analysis, we can conclude that the overall apoptotic machinery, including extrinsic and intrinsic apoptotic pathways, is present in the *S. frugiperda* genome. Moreover, we did not found noticeable difference in the panel of apoptosis-related genes in *S. frugiperda* genome with those of *B. mori*. Thus the apoptotic machinery seems to be highly conserved in Lepidoptera.

## S21. Heat Shock proteins

HSPs are a superfamily that has been widely studied in a wide range of organisms and that are expressed in response to a wide range of stressful environmental conditions and are generally viewed as a protective cellular mechanism. In addition to act as molecular chaperons, promoting correct refolding and preventing aggregation of denatured proteins in response to various stress factors, Hsps also play important role in diverse physiological and biological processes, including embryogenesis, diapause, and morphogenesis. These proteins are usually assigned to several families based on their molecular weights.

### S21.1 *Hsp* gene content of the corn strain

Following the Kampiga et al.'s guidelines for the nomenclature of HSP (2009) [241], we identified a total of 44 HSP-related unigenes with full length open reading frames (ORFs) in the *Spodoptera frugiperda* transcriptome (Supplementary Excel Table5, HSP Tab).
The majority of the HSP-related unigenes were predicted to encode members of the HSP70 superfamily (11 genes) and of the HSPB family (22 genes), also known as the small heat shock protein (sHSP) family. The HSP70 superfamily is divided in two families: HSPA (HSP70) family including for example the strictly stress-inducible HSP70 and the constitutive HSC70 (heat shock cognate proteins) and the HSPH (HSP110) family which includes in our S. frugiperda transcriptome, one HSP105 and one HSP97. Hsp70s function for facilitating the assembly of multimeric protein complexes and as molecular chaperons for facilitating intracellular folding of proteins, for secretion and transport, which generally interact with DNAjs/Hsp40s [242]. Similar sHSP diversity was reported in other insect genome or transcriptome as for example in *Bombyx mori* (16 HSPB) [243,244] and in *Rhyacionia leptotubula*

(17 HSPB) [245]. The HSPB family represents the proteins with low molecular weights of 12–43 kDa depending on the variable N- and C- terminal extensions, which contains a conserved alpha-crystallin domain. Functionally, these proteins act as molecular chaperones by binding partially denatured proteins [246].
The other HSP types among the HSP-related unigenes found in *S. frugiperda* transcriptome were members of the HSPC (HSP90) family (4 genes), of the Chaperonins family (one HSP10 and one HSP60), and of the DNAj (HSP40) family (5 genes). Hsp90 proteins are highly conserved molecular chaperones contributing to the folding, maintenance of structural integrity and proper regulation of a subset of cytosolic protein [247]. Although Hsp10 in insects has not been functionally defined in detail, it is well admitted that this protein, as in vertebrates, is an essential component of the protein folding apparatus, which co-chaperones with Hsp60 for protein folding as well as the assembly and disassembly of protein complexes [248,249]. Hsp40, homologues of bacterial DnaJ proteins also named DnaJ, are important for protein translation, folding, unfolding, translocation, and degradation, primarily by stimulating the ATPase activity of Hsp70s [250]. Hsp105/110 family is a divergent subgroup of the Hsp70 family. In insects, the role of Hsp105/110 has not been clearly defined whereas in mammals, the proteins of this family exist as complexes associated with Hsp70 (a constitutive form of Hsp70) and function to suppress the aggregation of denatured proteins in cells under severe stress, in which the cellular ATP level decreases markedly [251].
Finally, we also found several proteins known to interact with HSPs or allow regulation of their expression: two Heat shock Transcription Factors (HSF1 and 2) which are involved in the control of transcription of HSP genes [252], a Heat shock factor binding protein 1 (HSBP1) that binds to heat shock factor and help to control its transcriptional activities [253], a Hsp70-Hsp90 Organizing Protein (HOP) a co-chaperones which regulate and assist mainly HSPs [254], a C terminus of HSC70-Interacting Protein (CHIP) which binds to and inhibits the ATPase activity of the chaperone proteins HSC70 and HSP70 and blocks the forward reaction of the HSC70-HSP70 substrate-binding cycle [255], an activator of 90 kDa heat shock protein ATPase (AHSA), a cochaperone that stimulates HSP90 ATPase activity [256] and a mitochondrial import receptor subunit TOM70 also known as translocase of outer membrane 70 kDa subunit that accelerates the import of all mitochondrial precursor proteins [257].

### S21.2 Comparison with rice strain

In rice variant of the *Spodoptera frugiperda* transcriptome, only 65 HSP-related unigenes with full length open reading frames (ORFs) were found. The comparison with the corn variant revealed the absence of one HSPB (i.e. HSPB17) and two HSP70 members (i.e. HSP70-1 and 7). A clustalW analyses between the proteins of the two variants showed a relatively good conservation in amino acid sequences. The punctual modifications were reported (Supplementary Excel Table5, HSP Tab).

## S22. Oxydative stress related genes

All aerobic organisms possess enzymatic and non-enzymatic antioxidant systems to scavenge reactive oxygen species (ROS) generated as by-products of aerobic metabolism. Phytophagous insects are also exposed to ROS from pro-oxidant allelochemicals produced by the host-plant in response to herbivory [258,259]. In this context, antioxidant system of *Spodoptera frugiperda* is of particular interest.

### S22.1 Methods

Protein sequences of antioxidant of *D. melanogaster*, *A. gambiae, A. mellifera*, and *B. mori* were extracted from GenBank (http://www.ncbi.nlm.nih.gov/genbank/) and Flybase (http://flybase.org/) using both keyword searches and protein queries versus translated DNA database (tblastn). Candidate antioxidant genes from S. frugiperda were searched using the

tblastn program against the genome assembly (E-value cut-off = 1e-05), which include gene automatic annotation from the Genoscope. The identification of a putative ortholog was based both on protein sequence similarity and the presence of conserved domains predicted using Interpro (http://www.ebi.ac.uk/interpro/) [260]. The presence of signal peptide in the amino acid sequence was performed using SignalP (http://www.cbs.dtu.dk/services/SignalP/)[28]. Multiple amino acid sequences alignment were performed using Clustal Omega (http://www.ebi.ac.uk/Tools/msa/clustalo/) [201] or MUSCLE (http://phylemon.bioinfo.cipf.es) [261]. Phylogenetic analyses were performed using maximum-likelihood (ML) inference with the PhyML program [76].

### S22.2 Results

Thirty seven genes coding enzymes belonging to eight antioxidant enzymes families have been identified in the *S. frugiperda* genome (Supplementary Excel Table5, Oxydative stress Tab and Table S23). All of these genes code major components of the antioxidant system. Most of the antioxidant genes such as superoxide dismutases (Sod), catalase (Cat), glutathione peroxidases (Gtpx), glutaredoxin (Grx), thioredoxin peroxidases (Tpx), thioredoxin reductases (Trxr) and methionine sulphoxide reductases (Msr) are highly conserved and their number is almost the same as in other insects (*D. melanogaster*, *A. gambiae, A. mellifera*, and *B. mori*).

Superoxide dismutases (Sod) are ubiquitous metalloenzymes which catalyze the dismutation of the superoxide anion (O2•-) to hydrogen peroxide (H2O2). H2O2 is then converted to H2O by catalase and peroxidases. Eukaryotic Sod exists in two forms that differ in their metal cofactor and cellular localization: a cytoplasmic or extracellular Cu/Zn Sod and a nuclear-encoded mitochondrial Mn Sod. As other eukaryotes, *S. frugiperda* possess one gene coding MnSOD. We have identified 5 genes coding members of Cu/Zn Sod family (Table S23, Fig. S31), as in *A. gambiae*, *D. melanogaster* and *B. mori*. This family includes the canonical cytoplasmic SOD (Sod1), the copper chaperone for superoxide (Ccs), sodesque (Sodq), related to Sod (Rsod) and the extracellular Sod (Sod3). Sod3 of *S. frugiperda* has 200 amino acids and a putative signal peptide of 43 amino acids (SignalP prediction) [28].

Catalase catalyzes the breakdown of hydrogen peroxide into water and molecular oxygen. Three genes encoding catalase are present in the *S. frugiperda* genome SfCat1, SfCat2 and SfCat3. SfCat1 encodes a protein of 507 amino acids; no signal peptide was predicted suggesting a cytosolic localization as in other eukaryotes (Fig. S32). A second gene, SfCat2 encodes a protein of 523 amino acids that have a signal peptide of 15 residues. The presence of this signal peptide suggests that SfCat2 might be a putative secreted protein. Interestingly, catalase activity was detected in the foregut and the midgut of *Spodoptera littoralis* [259]. Both SfCat1 and SfCat2 contain the conserved catalytic residues H73 and N147, the heme-binding residues S112, V114, F151, F159, M297, M336, R340, Y344 and the NADPH-binding residues H192, R201, I276 and E281. A third gene, SfCat3 codes a shorter protein of 408 amino acids but SfCat3 lacks the catalytic residue H73 and two of the eight heme-binding residues, suggesting it is probably not active.

Peroxidases catalyze the reduction of hydrogen peroxide to water and oxidize various substrates. Peroxidases include heme-containing peroxidase (Hpx) and non heme-containing peroxidases.

Hpx has four classes, peroxinectin, peroxidasin, dual oxidase and double-peroxidase. Ten Hpx-coding genes are present in the *S. frugiperda* genome (Table S23). This gene family is significantly amplified in the mosquito *A. gambiae* (18 members) and in the silkworm *B. mori* (17 members) [262,263]. Seven *S. frugiperda* Hpx-coding genes (SfHpx1, SfHpx3, SfHpx5, SfHpx6, SfHpx7, SfHpx8, SfHpx16) show similarity with peroxinectin-coding genes from *B. mori*. Peroxidasin is a class of extracellular Hpx that combine multiple domains: leucine rich

repeats, immunoglobulin domains, a heme-binding peroxidase domain and a von Willebrand factor domain [264]. One peroxidasin-coding gene is found in the *S. frugiperda* genome (SfPxd), the primary transcript lacks the signal peptide for extracellular secretion and the von Willebrand factor domain. Dual oxidase (Duox) is another class of Hpx that contain a heme-binding peroxidase and a NADPH-oxidase domain. Duox play a role in the protection of insect gut against microbial invasion via the regulated production of ROS [265]. As in other insects (*D. melanogaster*, *A. gambiae*, *A. mellifera*, and *B. mori*), one gene encoding Duox is present in *S. frugiperda* (SfDuox) [266]. Double peroxidase is coded by one gene in *S. frugiperda* (SfDblox), the primary translation product of SfDblox contains two peroxidase domains.
The non heme-containing peroxidases include peroxiredoxin (Prx) and glutathione peroxidases (Gpx). Prx, also known as thioredoxin peroxidases, are a ubiquitous family of cysteine-based peroxidases regulating cellular peroxide levels. After the reduction of peroxide, the inactive oxidized form of Prx uses thioredoxin as an electron donor to regenerate the reduced active form. Three classes of Prx have been characterized: 1-Cys, typical 2-Cys, and atypical 2-Cys [267]. Four Prx homologs have been identified in the *S. frugiperda* genome. Sequence analysis of the primary transcripts with Peroxiscan tool reveals that SfPrx1, SfPrx2 and SfPrx3 are members of the typical 2-Cys subfamily and SfPrx4 is included in the 1-Cys subfamily.
Gpx catalyzes the reduction of hydroperoxides by reduced glutathione [268]. Surprisingly, only one Gpx-coding gene (SfGtpx) has been found in the *S. frugiperda* genome. Three Gpx homologs are also present in B. mori [262]. Two Gpx homologs are present in D. melanogaster genome, but at least one of them (Gtpx-1) uses thioredoxin instead of glutathione as electron donor. Three Gpx homologs are present in the *A. gambiae* genome and two of them are also likely to use thioredoxin as substrate [263]. These data suggest that SfGtpx could also use reduced thioredoxin.

Thioredoxins and glutaredoxins are oxidoreductase proteins that participate to maintain intracellular redox homeostasis. Oxidized Trx are regenerated by thioredoxin reductase (TrxR) through NADPH and oxidized Grx are regenerated by reduced glutathione [269]. In most organisms, oxidized glutathione is then reduced by glutathione reductase. However, in insect the oxidized glutathione is reduced by TrxR [270]. As in *D. melanogaster*, *A. gambiae* and *B. mori*, the *S. frugiperda* genome contains three genes encoding thioredoxins (SfTrx1, SfTrx2, SfTrx3) three genes encoding glutaredoxins (SfGrx1, SfGrx2, SfGrx3) and one gene coding thioredoxin reductase (SfTrxR). SfGrx3 contains a N-terminal thioredoxin domain and a C-terminal glutaredoxin domain. Three genes encoding Trx-related proteins (SfTrx-like 1, SfTrx-like 2, SfTrx-like 3) and two genes encoding Grx-related proteins (SfGrx-like 1, SfGrx-like 2) have been also identified in *S. frugiperda* genome as in other insect genomes (Table S23).

Methionine sulfoxide reductases (Msr) catalyze the reduction of methionine sulfoxide (the oxidative alteration of methionine) to methionine in oxidatively damaged proteins. There are two types of Msr, A and B, specific to the S- and R-diastereomers of methionine sulfoxide, respectively [271]. A single gene for each of these enzymes has been found in the *S. frugiperda* genome (Table S23).

## S23. Neuropeptides

Moulting, reproduction, motility, feeding, development, behaviour, metabolism, immune system, sex attraction, practically all the physiological and behavioural processes in an

insect's life are controlled by neuropeptides, small peptides synthesized by neurons. Neuropeptides comprise the largest class of extracellular signalling molecules that are involved in communication between insect cells [272] and so far, more than 30 different neuropeptides have been identified in insects [273,274].

### S23.1 Methods

Two complementary strategies were developed for the annotation of the whole set of neuropeptides present at the genome of *S. frugiperda*. In a first approach, a complete list of insect neuropeptides names was obtained from related literature [273-275]. Then, those names were searched in the OGS (Official Gene Set) automatically annotated in the *S. frugiperda* genome.

After manual curation of the obtained genes, a final group of 44 neuropeptides genes were detected. In a second approach, a complete list of known neuropeptides described in insects was obtained from the NeuroPep database (http://isyslab.info/NeuroPep/).

These, added to the 44 genes that were previously annotated, made a sum of 55 neuropeptide genes. Finally, each of the predicted peptide was confirmed in a one-by-one manner by blastp against Uniprot database (http://www.uniprot.org/) and their closest homolog in other insect species was reported. In addition, expression of each of the neuropeptide was reported in the main selected samples (early L2 larva and late L6 larva; head (antennas and palps) and fat body) by analysis of the presence of RNAseq reads mapping on each neuropeptide gene.

### S23.2 Results

Fifty-five genes encoding for potential neuropeptides were identified in the genome of S. frugiperda. The identified genes grouped in 29 different families, being the Diuretic Hormone, the Insulin-like peptide and the Ubiquin-like Protein the most abundant. Eight neuropeptide genes were simultaneously found in more than one scaffold, representing potential duplications. The annotated genes and their expression level is reported in Table S24

## SI Tables

### Table S1 Statistics for different sequencing technologies performed for *Spodoptera frugiperda* genomes.

| Species | Sequencing technology | Library preparation | Number of pairs | Number of bp | Coverage | Accession Number |
|---|---|---|---|---|---|---|
| Rice strain | Illumina HiSeq2000 | Paired-end reads (2x100bp) | 348,116,122 | 52,695,844,704 | 105.4X | |
| | Illumina HiSeq2000 | Paired-end reads (2x150bp) | 174,489,552 | 70,319,456,644 | 140.6X | |
| Corn strain | Illumina HiSeq2000 | Overlapping paired-end reads (2x100bp) | 332,360,409 | 66,213,918,249 | 132.4X | |
| | Illumina HiSeq2000 | Mate-pairs 3Kb (2x50bp) | 186,876,322 | 18,771,433,532 | 37.5X | |
| | Illumina HiSeq2000 | Mate-pairs 6Kb (2x100bp) | 155,886,269 | 30,399,430,322 | 60.8X | |
| | Illumina HiSeq2000 | Mate-pairs 7Kb (2x100bp) | 161,717,635 | 31,532,059,527 | 63.1X | |
| | Illumina HiSeq2000 | Mate-pairs 8Kb (2x100bp) | 166,360,793 | 32,514,734,407 | 65.0X | |

## Table S2 Statistics for *Spodoptera frugiperda* genome assemblies

| | Corn strain | | Rice strain |
|---|---|---|---|
| Assembly software | AllPaths-LG 43241 V3.0 | Corrected assembly V3.1 | Platanus V1.0 |
| # of scaffolds | 48,272 | 41,577 | 29,127 |
| Cumulative size (bp) | 526,022,508 | 437,873,293 | 371,020,023 |
| Scaffold N50 bp (L50) | 39,593 (2,682) | 52,781 (1,616) | 28 526 (3,761) |
| Scaffold N90 bp (L90) | 3,867 (22,307) | 3,545 (18,788) | 6,422 (13,881) |
| Number of N's | 13,625,586 (2.59%) | 11,379,916 (2.60 %) | 130,481 (0.04%) |
| GC percent | 35.92% | 36% | 36% |
| Accession Number | | PRJEB13110 | PRJEB13834 |

## Table S3 Complete statistics of mitochondrial and ribosomal DNA sequence assembly

| | | Target sequence | Kmer size | Number of PE reads | coverage | Contig size |
|---|---|---|---|---|---|---|
| Corn strain | mt genome | *Helicoverpa armigera* mt genome | 21 | 30,957 | 410X | 15,411 bp |
| | rDNA | *Papilio Xuthus* rDNA | 25 | 6,140 | 149X | 2,293 bp 557 bp and 4515 bp |
| | | Spades contigs of round1 | 25 | 4,253 | 103X | 7,817 bp |
| Rice strain | mt genome | *Spodoptera frugiperda* (Corn strain) mt genome | 21 | 10,962 | 221X | 15,431 bp |

## Table S4 Statistics for *Spodoptera frugiperda* genomes predictions.

| | Corn strain | | Rice strain |
|---|---|---|---|
| | OGS1.0 | OGS2.2 | OGS2.3 |
| # of predicted genes | 24,447 | 21,700 | 26,329 |
| # of unspliced genes | 6,315 | 5,407 | 2,069 |
| Average number of exon per gene | 4.57 | 4.62 | 5.23 |
| Median size of gene (kb) | 1.9 | 1.9 | 3.2 |
| Average coding size (kb) | 1.01 | 1.03 | 1.02 |
| Median size of introns (bp) | 406 | 408 | 442 |
| Coding base coverage (Mb) | 24.7 | 22.47 | 27.13 |

**Table S5 List of manually curated genes families, number of curated genes models in corn and rice strain**

| Annotation group | Number of curated gene models in corn strain OGS2.2 | Number of curated gene models in rice strain OGS2.3 | C strain 2016 03 21 | R strain 2016 03 21 |
|---|---|---|---|---|
| ABC transporters | 46 | 0 | 47 | 0 |
| Chemosensory | 577 | 465 | 579 | 466 |
| Circadian signaling | 28 | 7 | 27 | 13 |
| Developmental | 178 | 2 | 131 | 3 |
| Epigenetic | 23 | 0 | 26 | 1 |
| Esterases | 134 | 66 | 134 | 66 |
| Fat body metabolism | 8 | 0 | 9 | |
| GST | 57 | 49 | 57 | 49 |
| Hox | 33 | 0 | 219 | 160 |
| Immunity | 300 | 4 | 327 | 40 |
| Kinetochore | 14 | 3 | 14 | 10 |
| Midgut | 5 | 0 | 5 | 0 |
| miRNA | 8 | 0 | 8 | 0 |
| Osiris | 0 | 0 | 38 | 9 |
| Oxid. stress, hypoxia, autophagy, cell death, HSP | 235 | 67 | 247 | 70 |
| P450 | 141 | 175 | 148 | 176 |
| PIWI/ARGONAUTE | 18 | 0 | 18 | 0 |
| Serine proteases | 108 | 26 | 84 | 108 |
| Sex determining genes | 19 | 0 | 19 | 0 |
| UGT | 76 | 0 | 84 | 0 |
| Virus interaction | 3 | 0 | 5 | 0 |
| Neuropeptides | | | 49 | 0 |
| Miscellaneous | 45 | 1 | 55 | 2 |

### Table S6 Assessment of quality of genome assemblies by mapping of BAC ends sequences.

The end sequences of 32166 BAC have been mapped onto the corn strain genome. The number of BACs whose both ends mapped in the right orientation on a single scaffold, and distant by 50 to 200 kb, is shown. The average BACs length is 125 kb, longer than the N50 of the scaffolds, which explains that only a fraction of them mapped. However, reducing of heterozygosity in V3.1, increased the N50 of the scaffolds and the number of BACs end properly paired.

| | Corn strain | |
|---|---|---|
| Assembly software | AllPaths-LG 43241 V3.0 | Corrected assembly V3.1 |
| # properly paired | 2,262 | 4,045 |

## Table S7 BUSCO assessment of the completeness of genome assemblies

by mapping of Benchmarking Sets of Universal Single-copy orthologs (BUSCO, 2,675 for arthropoda species, http://busco.ezlab.org/). *Missing* means that the core protein was not found in the assembly, *Single copy* that the complete protein (its size is higher than average size of all the proteins in the BUSCO set minus 2 standard deviation), *fragmented* (its size is below the average size of all the proteins in the BUSCO set minus 2 standard deviation), or duplicated (more than one complete copy is found in the genome).

| | Corn strain | | Rice strain |
|---|---|---|---|
| | V3.0 | V3.1 | V1.0 |
| Missing | 363 | 336 | 220 |
| Single copy | 1,246 | 1,586 | 1,973 |
| Fragmented | 476 | 457 | 384 |
| Duplicated | 590 | 296 | 98 |

## Table S8 BUSCO assessment of the quality and completeness of automatic gene annotation

BUSCO, 2,675 genes for arthropoda species (http://busco.ezlab.org/), have been compared to the genome annotation at the protein level. *Missing* means that the core protein was not found in the gene predictions, *Single copy* that the complete protein (its size is higher than average size of all the proteins in the BUSCO set minus two standard deviation), *fragmented* (its size is below the average size of all the proteins in the BUSCO set minus two standard deviation), or duplicated (more than one complete copy is found in the genome).

| | Annotation | |
|---|---|---|
| | Corn strain (v2.0) | Rice strain (v2.0) |
| Missing | 306 | 287 |
| Single copy | 1522 | 1784 |
| Fragmented | 412 | 391 |
| Duplicated | 435 | 213 |

## Table S9 Genome coverage of different classes of transposable elements in the two strains

The TE classification follows Wicker's rules [276]. TIR (Terminal inverted repeats), LTR (Long terminal repeats)

| | | CORN strain | | RICE strain | |
|---|---|---|---|---|---|
| | | bp | % | bp | % |
| LTR retrotransposon | Copia | 0 | 0,00 | 0 | 0 |
| | Gypsy | 80950 | 0,02 | 65072 | 0,02 |
| | DIRS | 36929 | 0,01 | 9604 | 0,00 |
| | BEL | 209506 | 0,05 | 176029 | 0,05 |
| Non-LTR retrotransposon | LINE | 8542263 | 1,95 | 6374064 | 1,72 |
| | SINE | 54799928 | 12,52 | 48005818 | 12,94 |
| Putative_retrotransposon | Putative_RT | 304272 | 0,07 | 254472 | 0,07 |
| DNA | DNA | 1147985 | 0,26 | 894464 | 0,24 |
| Helitron | Helitron | 236489 | 0,05 | 149457 | 0,04 |
| Crypton | Crypton | 0 | 0,00 | 0 | 0,00 |
| TIR | TIR | 5637592 | 1,29 | 4285958 | 1,16 |
| Confused | Confused | 7398076 | 1,69 | 6563499 | 1,77 |
| Unclassified | Unclassified | 42206559 | 9,64 | 35406596 | 9,54 |
| Hostgene | Hostgene | 6572207 | 1,50 | 5516594 | 1,49 |
| Tandem repeats | Tandem repeats | 1026964 | 0,23 | 725488 | 0,20 |
| | Total | 128199720 | 29,28 | 108427115 | 29,22 |

## Table S10 Protein datasets used for orthology assessment

| Species | #proteins | Version | reference |
|---|---|---|---|
| *Bombyx mori* | 22,163 | SGP : GeneSet A, B and C (http://sgp.dna.affrc.go.jp/ComprehensiveGeneSet/) | [277] |
| *Danaus plexippus* | 16,254 | MonarchBase : 2.0 http://monarchbase.umassmed.edu/geneset.html) | [213] |
| *Drosophila melanogaster* | 30,385 | Flybase r6.03 (ftp://ftp.flybase.net/releases/FB2014_06/dmel_r6.03/fasta/dmel-all-translation-r6.03.fasta.gz) | [278] |
| *Heliconius melpomene* | 12,829 | ButterflyBase : v1.1 (http://www.butterflygenome.org/sites/default/files/Hmel1-1_Release_20120601.tgz) | [88] |
| *Manduca sexta* | 27,397 | ManducaBase : OGS2_20140407 (ftp://ftp.bioinformatics.ksu.edu/pub/Manduca/OGS2/OGS2_20140407.fa) | |
| *Spodoptera frugiperda* corn strain | 21,778 | SfruDB v2.2 | |
| *Spodoptera frugiperda* rice strain | 26,352 | SfruDB v2.2 | |

**Table S11 Number of proteins in different classes of orthologous groups.**
Here are reported numbers used to obtain Fig. S3.

| Species | ORPHAN | SF only | SF+ MSEX only | MOTH ONLY | LEPS ONLY | CORE SINGLE | CORE MULTI |
|---|---|---|---|---|---|---|---|
| *Spodoptera frugiperda* corn strain | 4422 (20.3%) | 1748 (8.0%) | 172 (0.7%) | 238 (1.1%) | 2913 (13.4%) | 745 (3.4%) | 6812 (31.3%) |
| *Spodoptera frugiperda* rice strain | 6080 (23.1%) | 3565 (13.5%) | 210 (0.8%) | 238 (0.9%) | 2603 (9.9%) | 745 (2.8%) | 6113 (23.2%) |
| *Manduca sexta* | 2481 (9.%) | NA | 263 (1.0%) | 411 (1.5%) | 4759 (17.4%)%) | 745 (2.7%) | 11605 (42.4%) |
| *Bombyx mori* | 6186 (27.9%) | NA | NA | 232 (1.0%) | 2566 (11.6%) | 745 (3.4%) | 5889 (26.6%) |
| *Danaus plexippus* | 2534 (15.6%) | NA | NA | NA | 2475 (125.2%) | 745 (4.6%) | 5559 (34.2%) |
| *Heliconius melpomene* | 1477 (11.5%) | NA | NA | NA | 2401 (18.7%) | 745 (5.8%) | 5341 (41.6%) |
| *Drosophila melanogaster* | 9385 (30.9%) | NA | NA | NA | NA | 745 (2.4%) | 15667 (51.6%) |

**Table S12 The distance between two individuals with homozygous non-variants (00), heterozygous variants (01), and homozygous variants (00).**

| | | 00 | 01 | 11 |
|---|---|---|---|---|
| Transition | 00 | 0 | 1 | 2 |
| | 01 | 1 | 1 | 1 |
| | 11 | 2 | 1 | 0 |
| Transversion | 00 | 0 | 2 | 4 |
| | 01 | 2 | 2 | 2 |
| | 11 | 4 | 2 | 0 |

## Table S13 Number of chemosensory genes annotated in lepidopteran genomes

| | CSP | OBP | IR | OR | GR |
|---|---|---|---|---|---|
| *Spodoptera frugiperda* **CORN variant** | **22** | **50** | **42** | **69** | **231** |
| *Spodoptera frugiperda* **RICE variant** | **22** | **51** | **43** | **69** | **230** |
| ***Bombyx mori*** [86,109,279] **K. Mita, pers. comm.)** | 21 | 43 | 25 | 70 | 74 |
| ***Manduca sexta*** [82,100, 280] | 19 | 49 | 21 | 71 | 45 |
| ***Danaus plexippus*** [88,213,279] | 34 | 32 | 27 | 64 | 47 |
| ***Heliconius melpomene*** [82,88,113,279] | 33 | 51 | ? | 66 | 73 |
| ***Plutella xylostella*** [115] | ? | ? | ? | 95 | 69 |

## Table S14 CYP genes clan composition in various arthropods

| | Species | total | clan2 | Clanmito | clan3 | clan4 |
|---|---|---|---|---|---|---|
| | *S. frugiperda* C strain | 117 | 8 | 11 | 59 | 39 |
| | *S. frugiperda* R. strain | 136 | 8 | 11 | 61 | 55 |
| | *Manduca sexta* | 117 | 10 | 17 | 52 | 38 |
| | *Heliconius Melpomeme* | 100 | 9 | 9 | 43 | 39 |
| | *Plutella xylostella* | 85 | 10 | 13 | 26 | 36 |
| Lepidoptera | *Bombyx mori* | 81 | 7 | 10 | 32 | 32 |
| Coleoptera | *Tribolium castaneum* | 134 | 8 | 9 | 72 | 45 |
| | *Apis mellifera* | 46 | 8 | 6 | 28 | 4 |
| Hymenoptera | *Nasonia vitripennis* | 92 | 7 | 7 | 48 | 30 |
| | *Drosophila melanogaster* | 88 | 7 | 11 | 36 | 32 |
| | *Anopheles gambiae* | 105 | 10 | 9 | 40 | 46 |
| Diptera | *Aedes aegypti* | 160 | 12 | 9 | 82 | 57 |
| Hemiptera | *Acyrthosiphon pisum* | 64 | 10 | 8 | 23 | 23 |
| Crustacea | *Daphnia pulex* | 75 | 20 | 6 | 12 | 37 |

## Table S15 Detailed list of P450 genes by clan in Corn and Rice strain of *Spodoptera frugiperda*

Underlined are strain specific genes having no ortholog in the other variant.

| | Family | Subfamily | No. of genes | |
|---|---|---|---|---|
| | | | Sf-corn | Sf-rice |
| Mitochondria l | CYP49 | A | CYP49A1 | CYP49A1 |
| | CYP301 | A, B | CYP301A1, CY301B1 | CYP301A1, CY301B1 |
| | CYP302 | A | CYP302A1 | CYP302A1 |
| | CYP314 | A | CYP314A1 | CYP314A1 |
| | CYP315 | A | CYP315A1 (V1 & V2) | CYP315A1 (V1 & V2) |
| | CYP333 | A, B | CYP333A12, CYP333B3, CYP333B4 (V1 & V2) | CYP333A12, CYP333B3, CYP333B4 (V1 & V2) |
| | CYP339 | A | CYP339A1 | CYP339A1 |
| | CYP428 | A | CYP428A1 | CYP428A1 |
| CYP2 | CYP15 | C | CYP15C1 | CYP15C1 |
| | CYP18 | A, B | CYP18A1, CYP18B1 | CYP18A1, CYP18B1 |
| | CYP303 | A | CYP303A1 | CYP303A1 |
| | CYP304 | F | CYP304F16 | CYP304F16 |
| | CYP305 | B | CYP305B1 | CYP305B1 |
| | CYP306 | A | CYP306A1 | CYP306A1 |
| | CYP307 | A | CYP307A2 | CYP307A2 |
| CYP3 | CYP6 | B | 7: CYP6B38, CYP6B39(V2), CYP6B40, CYP6B41(V2),CY6B42(V2), CYP6B50, CYP6B65P | 6: CYP6B38, CYP6B39(V1), CYP6B40, CYP6B41(V1,V2),CY6B42(V1,V2),CYP6B50 |
| | | AB | 5: CYP6AB12, CYP6AB58, CYP6AB59, CYP6AB60, CYP6AB61 | 5: CYP6AB12, CYP6AB58, CYP6AB59, CYP6AB60, CYP6AB61 |
| | | AE | 11: CYP6AE43, CYP6AE44, CYP6AE49, CYP6AE68, CYP6AE69, CYP6AE70(V1,V2), CYP6AE71, CYP6AE72, CYP6AE73, CYP6AE74, CYP6AE75 | 12: CYP6AE43, CYP6AE44, CYP6AE68, CYP6AE69, CYP6AE70(V1,V2), CYP6AE71, CYP6AE72, CYP6AE73, CYP6AE74, CYP6AE75, CYP6AE86,CYP6AE87 |
| | | AN | CYP6AN4 (V1,V2,V3) | CYP6AN4 |
| | | AW | CYP6AW1 | CYP6AW1 |
| | | CT | CYP6CT1 | CYP6CT1 |

| | | | | |
|---|---|---|---|---|
| | CYP9 | A | 14: CYP9A24, CYP9A25, CYP9A26, CYP9A27, CYP9A28, CYP9A29, CYP9A30, CYP9A31, CYP9A32, CYP9A58, CYP9A59, CYP9A60, CYP9A75, CYP9A76 | 15: CYP9A24, CYP9A25, CYP9A26, CYP9A27, CYP9A28, CYP9A28P, CYP9A29 (V1, V2), CYP9A30, CYP9A31, CYP9A32, CYP9A58, CYP9A59, CYP9A60, CYP9A75, CYP9A91 |
| | | G | CYP9G17 | CYP9G17 |
| | | AJ | CYP9AJ1 | CYP9AJ1 |
| | | BS | 0 | CP9BS1P |
| | CYP321 | A | 5: CYP321A7, CYP321A8, CYP321A9, CYP321A10, CYP321A15(V1,V2) | 5: CYP321A7, CYP321A8, CYP321A9, CYP321A10, CYP321A15 |
| | | B | 3: CYP321B1, CYP321B3, CYP321B4 | 3: CYP321B1, CYP321B3, CYP321B4 |
| | CYP324 | A | CYP324A16 (V1,V2), CYP324A17 (V1,V2), CYP324A18 | CYP324A16, CYP324A17 (V1,V2), CYP324A18 |
| | CYP332 | A | CYP332A1 | CYP332A1 |
| | CYP337 | B | CYP337B5 | CYP337B5 |
| | CYP338 | A | CYP338A1 | CYP338A1 (V1,V2) |
| | CYP354 | A | CYP354A14 | CYP354A14 |
| | CYP365 | A | CYP365A1 | CYP365A1 |
| | CYP3097 | A | CYP3097A1 | CYP3097A1 |
| CYP4 | CYP4 | G | 4: CYP4G74, CYP4G75, CYP4G108, CYP4G109 | 4: CYP4G74, CYP4G75, CYP4G108, CYP4G109 |
| | | L | 3: CYP4L9, CY4L12, CYP4L13 | 3: CYP4L9, CY4L12, CYP4L13 |
| | | M | 4: CYP4M14, CYP4M15,CYP4M17, CYP4M18 | 4: CYP4M14, CYP4M15,CYP4M17, CYP4M18 |
| | | S | 2: CYP4S8, CYP4S9 | 2: CYP4S8, CYP4S9 |
| | | AU | 3: CYP4AU1 (V1, V2) , CYP4AU2 (V1, V2) , CYP4AU2P | 2: CYP4AU1 , CYP4AU2 |
| | | CG | 2: CYP4CG16, CYP4CG18 | 2: CYP4CG16 (V1, V2), CYP4CG18 |
| | CYP340 | G | / | CYP340G2 |
| | | K | CYP340K14 | CYP340K14 |

| | | | | |
|---|---|---|---|---|
| | | L | 9: CYP340L1, CYP340L4, CYP340L9P, CYP340L11, CYP340L16, CYP340L18P, CYP340L19, CYP340L20 (V1, V2), CYP340L21 | 15: CYP340L1, CYP340L4, CYP340L5 (V1, V2), CYP340L6, CYP340L7, CYP340L8, CYP340L9P, CYP340L10, CYP340L11, CYP340L12 (V1,V2), CYP340L13 (V1,V2), CYP340L14, CYP340L15, CYP340L16, CYP340L17P |
| | | Q | / | CYP340Q4 |
| | | AA | / | 3: CYP340AA1 (V1,V2), CYP340AA2P, CYP340AA3P |
| | | AB | / | CYP340AB1 |
| | | AD | 1:CYP340AD3 | 2: CYP340AD3, CYP340AD4 |
| | | AH | / | CYP340AH |
| | | AX | / | CYP340AX1 |
| | CYP341 | A | CYP341A11 | CYP341A11 |
| | | B | 4: CYP341B15, CYP341B16 (V1, V2), CYP341B17, CYP341B18, CYP341B21 | 7: CYP341B15, CYP341B16 , CYP341B17 (V1, V2), CYP341B18, CYP341B21, CYP341B22, CYP341B23 |
| | CYP366 | A | CYP366A1 | CYP366A1 |
| | CYP367 | A | CYP367A12 | CYP367A12 |
| | | B | CYP367B11 | CYP367B11 |
| | CYP421 | B | CYP421B1 | CYP421B1 |

## Table S16 Comparison of GST gene number

| GST | *S.frugiperda* | *T.castaneum* | *D.melanogaster* | *A.gambiae* | *A.mellifera* | *B. mori* |
|---|---|---|---|---|---|---|
| Delta | 4 | 3 | 16 | 11 | 17 | 5 |
| Epsilon | 21 | 19 | 1 | 14 | 8 | 8 |
| Omega | 3 | 3 | 2 | 4 | 1 | 4 |
| Sigma | 8 | 7 | 6 | 1 | 1 | 2 |
| Theta | 1 | 1 | 2 | 4 | 2 | 1 |
| Zeta | 2 | 1 | 0 | 2 | 1 | 2 |
| Microsomal | 5 | 5 | 2 | 3 | 3 | 0 |
| Unkonwn | 1 | 2 | 3 | 1 | 2 | 1 |
| **Total** | **45** | **41** | **32** | **40** | **35** | **23** |

## Table S17 Comparison of the CCE repertoires of *B.mori* and *S. frugiperda*.

JHE: juvenile hormone esterase; ACHE: acetylcholinesterase ; GLI: gliotactin ; NLG: neuroligin ; NRT: neurotactin.

| Clade | *Spodoptera frugiperda* | *Bombyx mori* | |
|---|---|---|---|
| CCE001 | 20 | 7 | Includes CXE4/14, CXE28. CXE7 & CXE29 absent |
| CCE002 | 2 | 2 | |
| CCE003 | 1 | 1 | |
| CCE004 | 1 | 1 | |
| CCE005 | 1 | 1 | Includes CXE27 |
| CCE006 | 16 | 14 | Includes CXE6, CXE12 |
| CCE007 | 1 | 1 | Includes CXE17 |
| CCE008 | 1 | 1 | Includes CXE20 |
| CCE009 | 0 | 1 | |
| CCE010 | 1 | 1 | Includes CXE1 |
| CCE011 | 2 | 2 | Includes CXE8, CXE18 |
| CCE012 | 2 | 1 | |
| CCE013 | 1 | 1 | Includes CXE25 |
| CCE014 | 1 | 2 | |
| CCE015 | 2 | 1 | Includes CXE9, CXE22 |
| CCE016 | 13 | 4 | Includes CXE3, CXE10, CXE21 |
| CCE017 | 2 | 2 | Includes CXE23, CXE26 |
| CCE018 | 3 | 2 | Includes CXE11 |
| CCE019 | 1 | 1 | |
| CCE020 | 2 | 4 | JHE. Includes CXE2 and CXE15 |
| CCE021 | 3 | 3 | |
| CCE022 | 1 | 1 | |
| CCE023 | 1 | 1 | |
| CCE024 | 3 | 3 | Includes CXE5, CXE16, CXE24 |
| CCE025 | 1 | 1 | |
| CCE026 | 1 | 1 | Includes CXE13 |
| CCE027 | 2 | 2 | ACHE |
| CCE028 | 1 | 1 | GLI |
| CCE029 | 1 | 1 | Includes CXE19 |
| CCE030 | 6 | 6 | NLG |
| CCE031 | 1 | 1 | Includes CXE30 |
| CCE032 | 1 | 1 | NRT |
| CCE033 | 1 | 0 | |
| Total | 96 | 72 | |

## Table S18 Number of genes involved in immunity

found by genoscope automatic annotation (OGS 2.2) and supported by the presence of transcripts (TR2012b). Number of genes split on 2 or more scaffolds is indicated.

| Gene Family | Genes | OGS2.2 | TR2012b | Full | 2 or more scaffolds |
|---|---|---|---|---|---|
| **Recognition** | 51 | 69 | 48 | 23 | 28 |
| **Intracellular signaling** | 58 | 90 | 66 | 40 | 18 |
| **Effectors** | 54 | 57 | 50 | 44 | 10 |
| **Total** | **163** | **216** | **164** | **107** | **56** |

## Table S19 Immunity genes

Gene counts for subsets of gene families involved in insect immunity. Bombyx mori, Drosophila melanogaster and Anopheles gambiae, and Apis mellifera counts based on [184,188,281] respectively and newer analyses.

| Gene family | *S. frugiperda* | *B. mori* | *D. melanogaster* | *A. gambiae* | *A. mellifera* |
|---|---|---|---|---|---|
| *Recognition* | 45 | 70 | 97 | 86 | 42 |
| PGRP-S | 6 | 6 | 7 | 3 | 3 |
| PGRP-L | 4 | 6 | 6 | 4 | 1 |
| ◎GRP | 4 | 4 | 3 | 6 | 2 |
| Hemolin | 1 | 1 | 0 | 0 | 0 |
| Scavenger receptor A | - | 4 | 5 | 5 | 3 |
| Scavenger receptor B | 3 | 13 | 12 | 15 | 9 |
| Scavenger receptor C | 1 | 1 | 4 | 1 | 1 |
| C-type lectin | 8 | 21 | 35 | 22 | 10 |
| Hemocytin | 1 | 1 | 1 | 0 | 1 |
| Galectin | 8 | 4 | 5 | 8 | 2 |
| TEP | 2 | 3 | 6 | 15 | 4 |
| Nimrod A | 2 | 4 | 10 | 4 | 4 |
| Draper | - | 1 | 1 | 1 | 1 |
| Eater | 2 | 0 | 1 | 1 | 0 |
| Dscam | 3 | 1 | 1 | 1 | 1 |
| *Signaling* | | | | | |
| Toll pathway | 24 | 27 | 25 | 27 | 19 |
| Spätzle | 3 | 3 | 6 | 6 | 2 |
| Toll | 12 | 14 | 9 | 11 | 5 |
| MyD88 | 2 | 1 | 1 | 1 | 1 |
| Tollip | 1 | 2 | 1 | 2 | 1 |
| Tube | 1 | 1 | 1 | 1 | 1 |
| Pellino | 1 | 1 | 1 | 1 | 1 |
| Pelle | 1 | 1 | 1 | 1 | 1 |
| TRAF2 | n.f. | 1 | 1 | 1 | 1 |
| ECSIT | 1 | 1 | 1 | 1 | 1 |
| Cactus | 1 | 1 | 1 | 1 | 3 |
| Dif/Dorsal | n.f./1 | 1 | 2 | 1 | 2 |
| Imd pathway | 9 | 10 | 10 | 10 | 11 |
| IMD | 1 | 1 | 1 | 1 | 1 |
| Dredd | n.d. | 1 | 1 | 1 | 1 |
| TAK1 | 1 | 1 | 1 | 1 | 1 |
| FADD | 1 | 1 | 1 | 1 | 1 |
| Tab2 | 1 | 1 | 1 | 1 | 1 |
| IAP2 | 1 | 1 | 1 | 1 | 1 |
| IKK◎ | 1 | 1 | 1 | 1 | 1 |
| IKK◎ | 1 | 1 | 1 | 1 | 1 |
| Ubc13 | 1 | 1 | 1 | 1 | 1 |

| | | | | | |
|---|---|---|---|---|---|
| Relish | 1 | 1 | 1 | 1 | 2 |
| JNK pathway | 6 | 4 | 4 | 4 | 4 |
| Hem | 1 | 1 | 1 | 1 | 1 |
| JNK | 1 | 1 | 1 | 1 | 1 |
| Fos | 3 | 1 | 1 | 1 | 1 |
| Jun | 1 | 1 | 1 | 1 | 1 |
| JAK/STAT pathway | 5 | 4 | 6 | 6 | 5 |
| Upd3 | n.f. | 0 | 1 | 0 | 0 |
| PIAS | 1 | 1 | 1 | 1 | 1 |
| SOCS | 1 | 1 | 1 | 1 | 1 |
| Domeless | 1 | 1 | 1 | 1 | 1 |
| Hopscotch | 1 | 0 | 1 | 1 | 1 |
| STAT | 1 | 1 | 1 | 2 | 1 |
| Effectors | 50 | 37 | 44 | 32 | 9 |
| PPO | 2 | 2 | 3 | 9 | 1 |
| POI | 2 | 1 | 2 | 1 | 0 |
| Lysozyme | 3 | 1 | 8 | 4 | 0 |
| Lysozyme-like protein | 2 | 3 | 3 | 4 | 2 |
| Cecropin | 6 | 13 | 4 | 4 | 0 |
| Attacin | 5 | 2 | 4 | 1 | 0 |
| Defensin | 6 | 1 | 1 | 4 | 2 |
| Gloverin | 2 | 4 | 0 | 0 | 0 |
| Moricin & Moricin-like protein | 10 | 9 | 0 | 0 | 0 |
| Lebocin | 3 | 1 | 0 | 0 | 0 |
| Other Amp*** | 9 | 0 | 19 | 5 | 4 |
| **Total** | **139** | **152** | **186** | **165** | **90** |

## Table S20 HD genes with paralogs in Drosophila not in Lepidoptera

| *Drosophila* | *Bombyx* | *Spodoptera* |
|---|---|---|
| nub/pdm2 | / | pdm2 |
| lbe/lbl | LBX1-like | lbx |
| BH1/BH2 | B-H1-like | Barhl |
| E5/ems | empty spiracles-like | emx |
| eyg/toe | Pax3A-like | Pax4 |
| ey/toy | Pax-6-like | Pax6 |
| vsx1/vsx2 | visual system homeobox2 | Vsx |
| unc4/OdsH | unc-4 | unc-4 |
| CG32105/CG4328 | LIM homeobox 1-beta | Lmx |

## Table S21 HD genes with paralogs in Lepidoptera but not in Drosophila

| *Drosophila* | *Bombyx* | *Spodoptera* |
|---|---|---|
| apterous (ap) | apterous/apterous A | apA/apB |
| aristaless (al) | aristaless-like/aristaless-like | al-1/al-2 |
| homothorax (hth) | homothorax/PKNOX2 | hth/PKNOX |

## Table S22 Sequence homology of Atg8, PI3K, Akt and TOR proteins from different organisms.

The degree of similarity of *Spodoptera frugiperda* proteins with other organisms was determined with the NCBI Blast software (http://blast.ncbi.nlm.nih.gov/) using standard conditions of the software. Identifiants (ID) of Atg8, PI3K, Akt and TOR proteins from *Bombyx mori*, *Galleria mellonella*, *Helicoverpa armigera*, *Papilio xuthus*, *Danaus plexippus*, *Drosophila melanogaster*, *Homo sapiens*, *Aedes aegypti*, *Nasonia vitripennis*, *Apis florea* are indicated.

| Protein | Organism | ID | % similarity |
|---|---|---|---|
| **Atg8** | *S. frugiperda* | GSSPFG00035793001.1-RA | 100 |
| | *B. mori* | 114052412 | 99 |
| | *G. mellonella* | 400073886 | 100 |
| | *H. armigera* | 389604114 | 100 |
| | *P. xuthus* | 389608575 | 100 |
| | *D. plexippus* | 357624756 | 100 |
| | *D. melanogaster (Atg8a)* | 7291184 | 99 |
| | *H. sapiens (GABARAP)* | 13899219 | 94 |
| | *H. sapiens (MAPLC3)* | 14210522 | 60 |
| | *S.frugiperda* | GSSPFT00020142001 | 100 |
| **PI3K** | *B.mori* | 512886038 | 87 |
| | *A.aegypti* | 157132832 | 65 |
| | *D.melanogaster* | 21356197 | 60 |
| | *N.vitripennis* | 156541823 | 67 |
| | *A.florea* | 380018616 | 68 |
| | *H.sapiens* | 67477424 | 58 |
| | *S.frugiperda* | GSSPFT00005567001 | 100 |
| **Akt** | *B.mori* | 163962993 | 94 |
| | *A.aegypti* | 30725240 | 80 |
| | *D.melanogaster* | 24647358 | 78 |
| | *N.vitripennis* | 156537289 | 74 |
| | *A.florea* | 380015932 | 73 |
| | *H.sapiens (Akt1)* | 62241015 | 76 |
| | *H.sapiens (Akt2)* | 111309392 | 68 |
| | *H.sapiens (Akt3)* | 5804886 | 76 |
| | *S.frugiperda (TOR1)* | GSSPFT00031300001 (TOR1 part1) | 100 |
| **TOR 1** | *S.frugiperda (TOR1)* | GSSPFT00027097001 (TOR1 part2) | 100 |
| | *S.frugiperda (TOR2)* | GSSPFT00027094001 (TOR2) | 79 |
| | *B.mori (BmTOR1)* | 284517116 | 91 |
| | *B.mori (BmTOR2)* | 284517118 | 78 |
| | *A.aegypti* | 40888981 | 70 |
| | *D.melanogaster* | 17864562 | 67 |
| | *N.vitripennis* | 345489192 | 71 |
| | *A.florea* | 380015740 | 72 |
| | *H.sapiens* | 4826730 | 66 |
| | *S.frugiperda (TOR1)* | GSSPFT00031300001 (TOR 1 part1) | 79 |

| | | | |
|---|---|---|---|
| **TOR 2** | *S.frugiperda (TOR1)* | GSSPFT00027097001 (TOR 1 part2) | 79 |
| | *S.frugiperda (TOR2)* | GSSPFT00027094001 (TOR 2) | 100 |
| | *B.mori (BmTOR1)* | 284517116 | 76 |
| | *B.mori (BmTOR2)* | 284517118 | 94 |
| | *A.aegypti* | 40888981 | 75 |
| | *D.melanogaster* | 17864562 | 72 |
| | *N.vitripennis* | 345489192 | 77 |
| | *A.florea* | 380015740 | 78 |
| | *H.sapiens* | 4826730 | 71 |

## Table S23 Major components of enzymatic antioxidant system of *Spodoptera frugiperda*, *Drosophila melanogaster*, *Anopheles gambiae* and *Bombyx mori*.

| Gene name | Symbol | *Sopdoptera frugiperda* | *Drosophila melanogaster* | *Anopheles gambiae* | *Bombyx mori* |
|---|---|---|---|---|---|
| Superoxide dismutase (Cu/Zn) | *SfSod1* | GSSPFG00013592001 | CG11793 | XP_311594 | BAD69805 |
| Superoxide dismutase 2 (Mn) | *SfSod2* | GSSPFT00028152001 | CG8905 | AAR90328 | NP_001037299 |
| Superoxide dismutase 3 | *SfSod3* | GSSPFT00024862001 | CG9027 | AAS17758 | BGIBMGA005489 |
| Copper chaperone for superoxide dismutase | *SfCcs* | GSSPFT00022465001 | CG17753 | XP_308747 | BGIBMGA001698 |
| Related to SOD | *SfRsod* | GSSPFT00027089001 | CG31028 | EAA00894 | BGIBMGA002311 |
| Sodesque | *SfSodq* | GSSPFG00022962001 | CG5948 | EAA04552 | BGIBMGA002798 |
| Catalase | *SfCat1* | GSSPFT00030477001 | CG6871 | XP_314995 | NP_001036912 |
| | *SfCat2* | GSSPFT00000106001 | | | |
| | *SfCat3* | GSSPFT00024144001 | | | |
| Heme-containing peroxidase | *SfHpx1* | GSSPFG00031018001 | XP_311448 | CG3477 | BGIBMGA006520 |
| | *SfHpx3* | GSSPFG00015708001 | XP_313514 | CG6879 | BGIBMGA005680 |
| | *SfHpx5* | GSSPFG00032295001 | XP_311106 | CG5873 | BGIBMGA014559 |
| | *SfHpx6* | GSSPFG00006524001 | XP_309656 | CG6969 | BGIBMGA013482 |
| | *SfHpx7* | GSSPFG00018725001 | XP_309590 | | BGIBMGA012737 |
| | *SfHpx8* | GSSPFG00015208001 | XP_309592 | CG7660 | BGIBMGA013640 |
| | *SfHpx16* | GSSPFG00025186001 | XP_309429 | | BGIBMGA006519 |
| | *SfPxd* | GSSPFT00023252001 | XP_308561 | CG12002 | BGIBMGA000553 |
| | *SfDuox* | GSSPFT00001640001 | XP_319115 | CG3131 | BGIBMGA005478 |
| | *SfDblox* | GSSPFG00002871001 | XP_317106 | CG10211 | BGIBMGA007042 |
| Peroxiredoxin 1 | *SfPrx1* | GSSPFT00030261001 (part1) GSSPFG00035440001 (part2) | XP_308081 | CG1633 | NP 001037083 |
| Peroxiredoxin 2 | *SfPrx2* | GSSPFG00011444001 | XP_308336 | CG1274 | BGIBMGA002406 |
| Peroxiredoxin 3 | *SfPrx3* | GSSPFG00021219001 | XP_565975 | CG5826 | NP 001040464 |
| Peroxiredoxin 4 | *SfPrx4* | GSSPFT00009048001 (part1) GSSPFT00011952001 (part2) | XP_320690 | CG12405 | |
| Glutathione peroxidase | *SfGtpx* | GSSPFT28057001 | XP_313166 | CG12013 | NP 001040104 |
| Thioredoxin 1 | *SfTrx-1* | GSSPFG00012450001 (part1) GSSPFG00035225001 (part2) | EAA04498 | CG8993 | ABM_92269 |
| Thioredoxin 2 | *SfTrx-2* | GSSPFT00032690001 (partial) | EAA14495 | CG31884 | NP 001040283 |
| Thioredoxin 3 | *SfTrx-3* | GSSPFT00018815001 | EAA09650 | CG3719 | BGIBMGA008199 |
| Thioredoxin reductase | *SfTrxR* | GSSPFT00002751001 (part1) GSSPFT00023968001 (part2) | CAD30858 | CG2151 | BGIBMGA002818 |
| Thioredoxin-like 1 | *SfTrx-like 1* | GSSPFT00032039001 | EAA11972 | CG5495 | NP_001040348 |
| Thioredoxin-like 2 | *SfTrx-like 2* | GSSPFT00000559001 | XP_320264 | CG14221 | BGIBMGA006070 |
| Thioredoxin-like 3 | *SfTrx-like 3* | GSSPFT00029301001 | XP_316887 | CG9911 | BGIBMGA006941 |
| Glutaredoxin 1 | *SfGrx1* | GSSPFT00017667001 | XP_309539 | CG6852 | NP_001040246 |
| Glutaredoxin 2 | *SfGrx2* | GSSPFT00028944001 | XP_312440 | CG14407 | BGIBMGA008525 |
| Glutaredoxin 3 | *Grx3* | GSSPFG00017176001 | EAA07378 | CG6523 | BGIBMGA006401 |
| Glutaredoxin-like | *SfGrx-like* | GSSPFG00017105001 | EAA06446 | CG31559 | BGIBGA013430 |
| Methionine sulfoxide reductase A | *SfMsrA* | GSSPFT00009150001 | XP_320164 | CG7266 | ABF_51258 |
| Methionine sulfoxide | *SfMsrB* | GSSPFT00006354001 | XP_003436334 | CG6584 | BGIBMGA007514 |

reductase B

### Table S24 Neuropeptides genes in the Corn strain genome and their expression level

*according to the maximum number of reads per position for each gene:
No Expression: - (no reads detected)
Low Expression: + (1-10 reads)
Medium Expression: ++ (10-100 reads)
High Expression: +++ (100-1000 reads)

| Neuropeptide | Copies | Scaffold CORN | First Hit<br>Organism | Acc. Number | E-value | Expression Level*<br>Head | Fat Body | Early L2 Larva | Late L6 Larva |
|---|---|---|---|---|---|---|---|---|---|
| Adipokinetic Hormone 1 | 2 | 18146 | *H.armigera* | AGH25544.1 | 9E-31 | - | + | - | - |
| Adipokinetic Hormone 1 | | 21318 | *M.separata* | ALX27200.1 | 6E-32 | + | - | + | - |
| Adipokinetic Hormone 2 | 1 | s336 | *H.armigera* | AGH25545.1 | 7E-39 | - | - | + | - |
| Allatostatin C | 2 | 1794 | *S.frugiperda* | Q868F8.1 | 3E-84 | + | + | + | + |
| | | 1873 | *S.frugiperda* | Q868F8.1 | 3E-84 | + | + | + | - |
| Allatotropin | 2 | 1236 | *H.armigera* | AAT92286.1 | 6E-152 | + | + | + | + |
| | | s365 | *H.armigera* | AAT92286.1 | 1E-157 | + | + | + | + |
| Bursicon α Subunit | 1 | 10137 | *H.armigera* | AHM02472.1 | 4E-98 | - | + | + | - |
| Bursicon β Subunit | 1 | 10137 | *H.armigera* | AHM02473.1 | 2E-79 | - | + | + | - |
| CAPA | 1 | 235 | *H.armigera* | AGH25549.1 | 1E-76 | - | + | + | + |
| CCHamide 1 | 1 | 2664 | *B.mori* | ALM30310.1 | 1E-57 | + | - | + | - |
| CCHamide 2 | 1 | 8059 | *H.armigera* | AGH25550.1 | 4E-54 | + | - | - | - |
| Corazonin | 1 | s998 | *H.armigera* | AGH25551.1 | 4E-62 | + | + | + | - |
| Diuretic Hormone 31 - pseudogene | 1 | 24411 | *A. Trasitella* | P82372.1 | 6E-11 | - | + | ++ | + |
| Diuretic Hormone 34 | 1 | 473 | *H.armigera* | AGH25555.1 | 3E-47 | + | ++ | ++ | + |
| Diuretic Hormone 41 | 1 | 473 | *H.armigera* | AGH25554.1 | 1E-29 | ++ | ++ | ++ | + |
| Diuretic Hormone 45 | 1 | 473 | *O.brumata* | KOB78000.1 | 5E-31 | + | + | ++ | + |
| Eclosion Hormone | 1 | 12966 | *H.armigera* | AAV69026.1 | 4E-35 | + | - | + | - |
| FMRFamide | 1 | 10702 | *H.armigera* | AGH25556.1 | 1E-93 | - | - | + | - |
| Glycoprotein Hormone α 2 | 1 | 1138 | *P.machaon* | XP_014362533.1 | 2E-74 | - | - | + | + |
| Glycoprotein Hormone β 5 | 1 | s967 | *B.mori* | CAR95348.2 | 1E-66 | + | - | + | + |
| Insulin-like peptide 2 | 1 | 16636 | *S.exigua* | AIA56827.1 | 7E-10 | ++ | ++ | + | + |
| Insulin-like peptide 2 | 1 | 1399 | *S.exigua* | AIA56827.1 | 2E-11 | + | + | + | + |

| | | | | | | | | | |
|---|---|---|---|---|---|---|---|---|---|
| Insulin-like polypeptide 1 | 1 | s111 | *S.littoralis* | AEE43936.1 | 4E-50 | - | - | + | - |
| Insulin-like polypeptide 2 | 1 | s1169 | *S.littoralis* | AEE43937.1 | 7E-65 | - | - | + | - |
| Insulin-like polypeptide A | 2 | 170 | *H.armigera* | AGH25572.1 | 6E-35 | - | - | - | - |
| | | 23585 | *H.armigera* | AGH25572.1 | 4E-28 | - | - | - | - |
| Insulin-like polypeptide D | 1 | s719 | *H.armigera* | AGH25575.1 | 2E-69 | + | - | ++ | + |
| Ion Transport Peptide - CCH-like | 2 | 1847 | *D.plexippus* | EHJ71841.1 | 2E-65 | + | - | + | + |
| | | s668 | *D.plexippus* | EHJ71841.1 | 6E-65 | + | + | + | - |
| Long Neuropeptide F | 1 | 567 | *D.plexippus* | XP_013142257.1 | 3E-70 | + | + | + | + |
| Myosuppressin - pseudogene | 1 | 9060 | *S.littoralis* | CAO86065.1 | 2E-09 | + | + | + | + |
| Neuroparsin | 1 | s428 | *H.armigera* | AGH25563.1 | 1E-50 | - | + | - | - |
| Neuropeptide Y/F2 | 2 | 2020 | *H.virescens* | AEE01344.1 | 1E-57 | - | - | - | - |
| | | 12868 | *H.virescens* | AEE01344.1 | 1E-57 | - | + | + | + |
| Orcokinin | 1 | 15368 | *A.transitella* | XP_013186838.1 | 7E-91 | + | - | ++ | ++ |
| PBAN-DH | 2 | 1995 | *S.litura* | AAK84160.1 | 7E-132 | + | + | + | + |
| | | 713 | *S.litura* | AJT60314.1 | 3E-132 | + | + | + | + |
| Proctolin | 1 | 109 | *P.machaon* | XP_014369719.1 | 3E-108 | + | + | + | + |
| Prothoraticostatic/Myoinhibitory Peptide | 2 | 2755 | *H.armigera* | AGH25567.1 | 3E-139 | + | + | ++ | + |
| | | 6192 | *A.transitella* | AGH25567.1 | 9E-92 | - | + | ++ | + |
| Prothoraticostatic/Myoinhibitory Peptide - pseudogene | 1 | 3620 | *H.armigera* | XP_013195980.1 | 1E-123 | + | + | ++ | + |
| Short Neuropeptide F | 1 | 5344 | *H.armigera* | AGH25568.1 | 3E-57 | - | - | ++ | + |
| SIFamide | 1 | 12670 | *H.armigera* | AGH25569.1 | 8E-34 | - | - | - | - |
| Sulfakinin | 1 | 102 | *H.armigera* | AGH25570.1 | 2E-30 | - | - | + | - |
| Tachykinin | 1 | 9301 | *H.armigera* | AGH25571.1 | 2E-133 | - | - | - | - |
| Ubiquitin-like Protein 3 | 1 | 3162 | *P.xuthus* | KPJ02072.1 | 5E-75 | + | + | + | + |
| Ubiquitin-like Protein 4A | 1 | 9308 | *A.transitella* | XP_013201077.1 | 2E-61 | ++ | ++ | ++ | ++ |
| Ubiquitin-like Protein 5 | 1 | 1184 | *D.plexippus* | EHJ73714.1 | 1E-44 | ++ | ++ | + | + |
| Ubiquitin-like Protein 7 | 1 | 816 | *B.mori* | XP_004929615.1 | 6E-179 | ++ | ++ | ++ | ++ |

| | | | | | | | | | |
|---|---|---|---|---|---|---|---|---|---|
| Ecdysis Triggering Hormone | 2 | 241 | *M.sexta* | AAD45613.1 | 1E-36 | + | + | ++ | + |
| | | 254 | *M.sexta* | AAD45613.1 | 1E-36 | + | + | + | + |
| Leucokinin | 1 | 11641 | *H.armigera* | AGH25561.1 | 0E+00 | - | - | ++ | + |
| Neuropeptide-like Precursor 1 | 1 | s1116 | *P.xuthus* | XP_013179800.1 | 3E-173 | - | - | + | + |
| Neuropeptide-like Precursor 4 | 1 | s609 | *D.plexippus* | EHJ74726.1 | 2E-17 | ++ | + | ++ | ++ |

## SI Figures

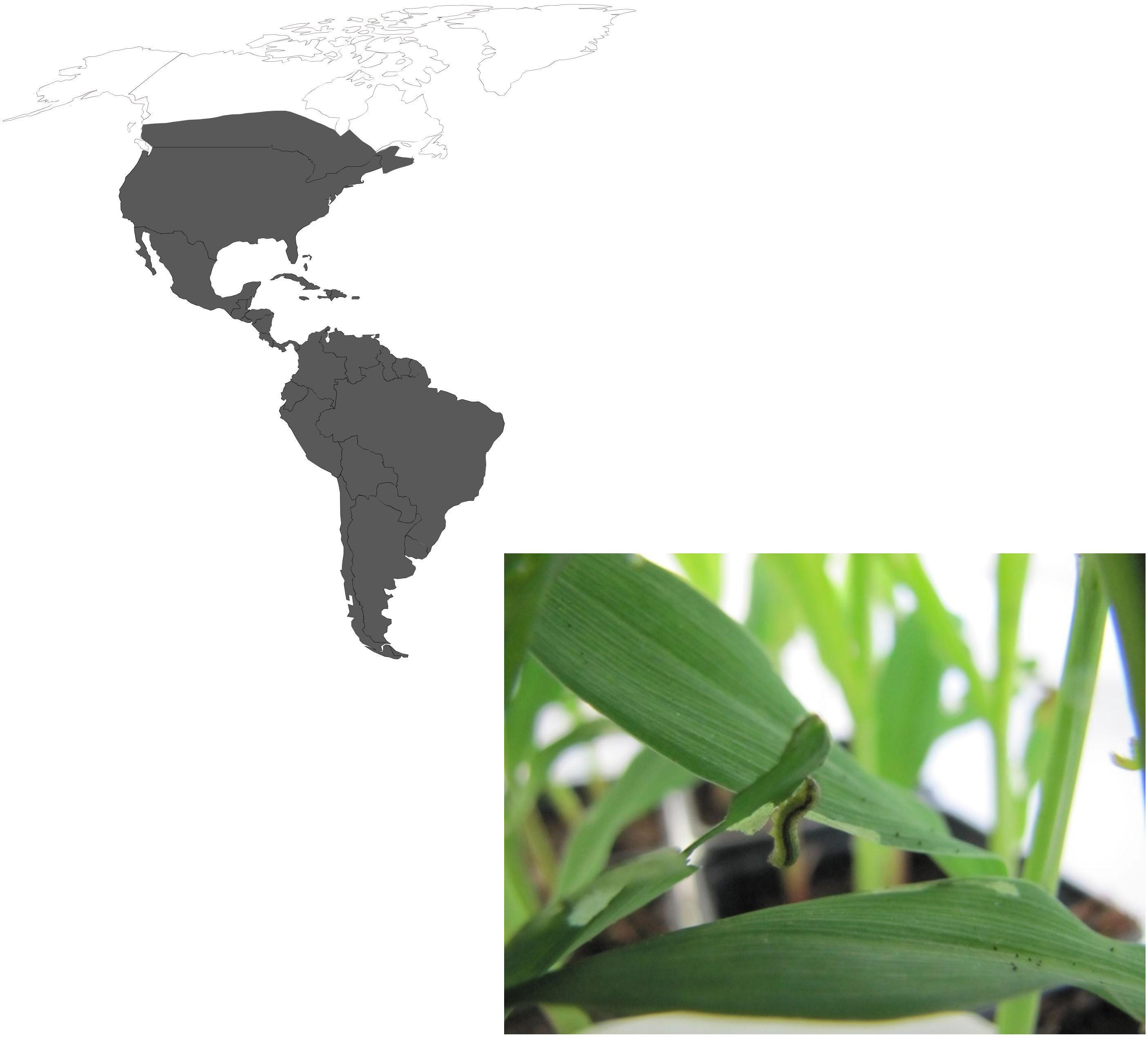

**Figure S1 Distribution area of *Spodoptera frugiperda* and picture of a caterpillar on corn**

The two corn and rice strains leave in sympatry on the American continent. The map was created using the software Microsoft Powerpoint 10
https://www.microsoft.com/
using a map template available at http://www.freeworldmaps.net/powerpoint/
(http://www.freeworldmaps.net/about.html for copyright).
Picture showing *S. frugiperda* at larval stage causing damages on corn.

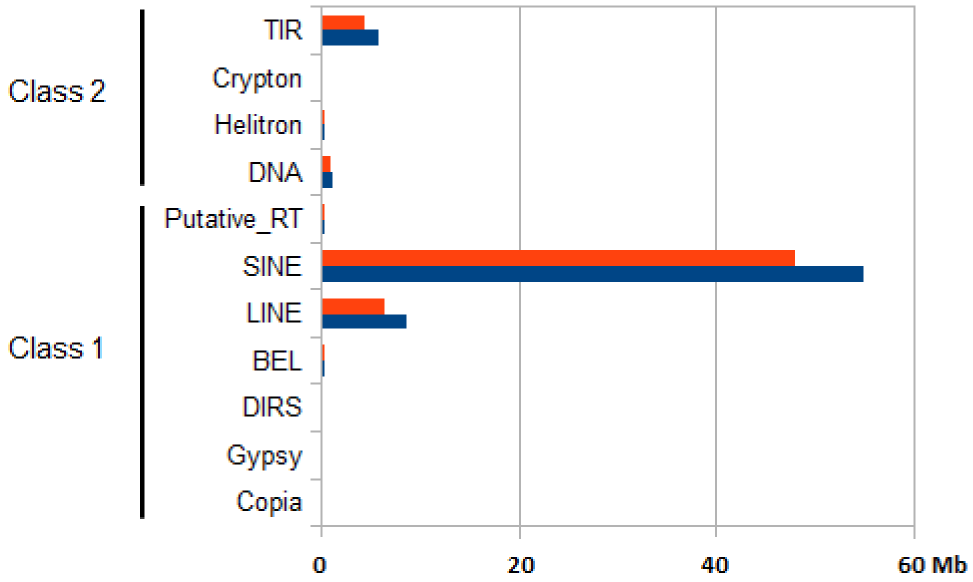


**Figure S2 Comparison of TE content of the two *Spodoptera frugiperda* corn and rice strains**

The TE genome coverage is shown, in corn strain (blue) or in rice strain (red).

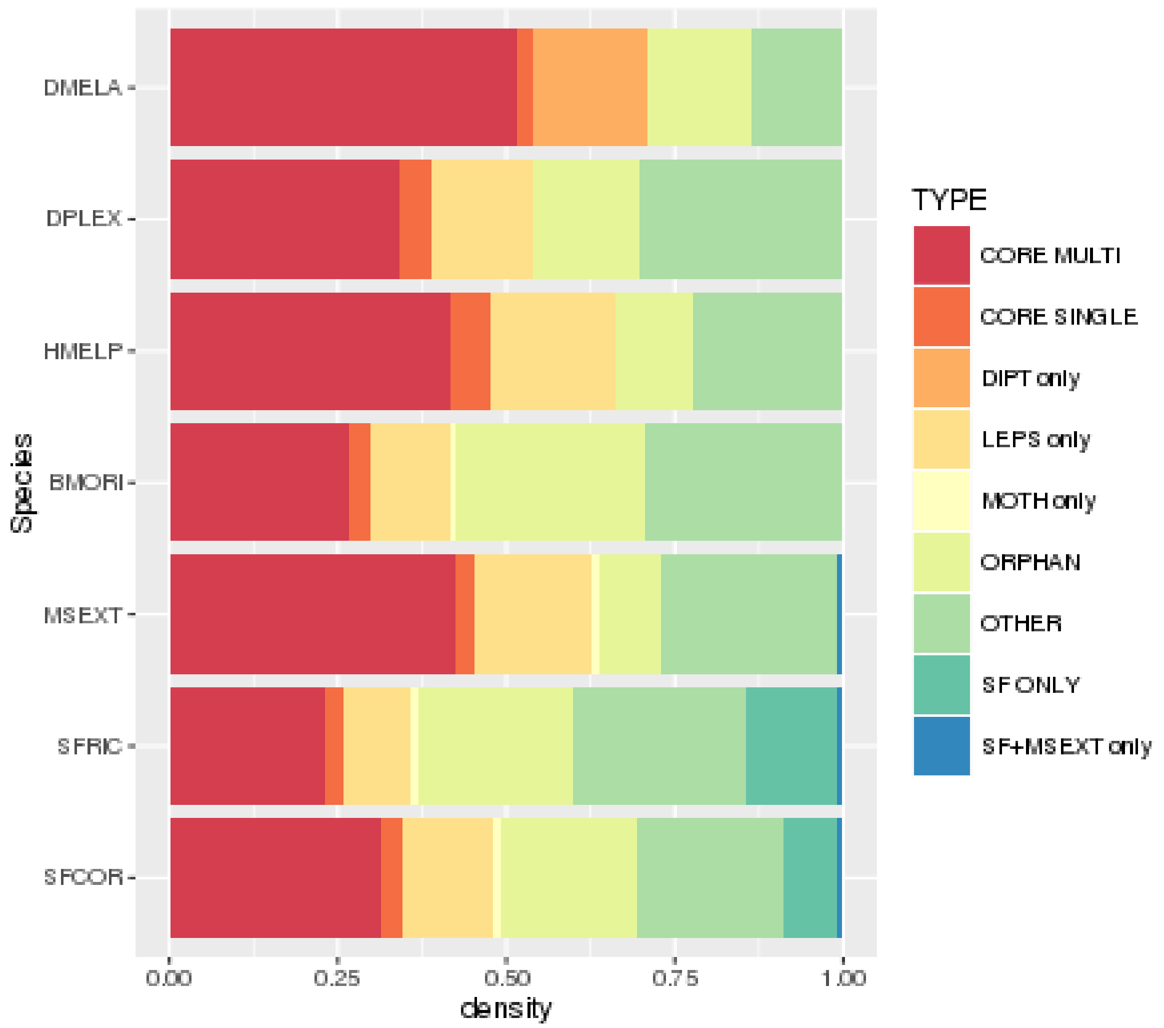


**Figure S3 Number of proteins in different classes of orthologous groups.**
Histogram representation. The plot shows the number of proteins for 6 lepidopteran species that have been uniquely found in the species genome (ORPHAN), only in *Spodoptera frugiperda* variant (SF only), only in the *Spodoptera frugiperda* variants and *Manduca sexta* (SF + MSEX), only in moths (*Spodoptera frugiperda*, *Manduca sexta* and *Bombyx mori*) or only in lepidopteran species (LEPS only). DMELA = *Drosophila melanogaster* DPLEX= *Danaus plexippus*, HMELP= *Heliconius melpomene* BMORI = *Bombyx mori* MSEXT= *Manduca sexta* SFRIC = *Spodoptera frugiperda* rice strain SFCOR = *Spodoptera frugiperda* corn strain

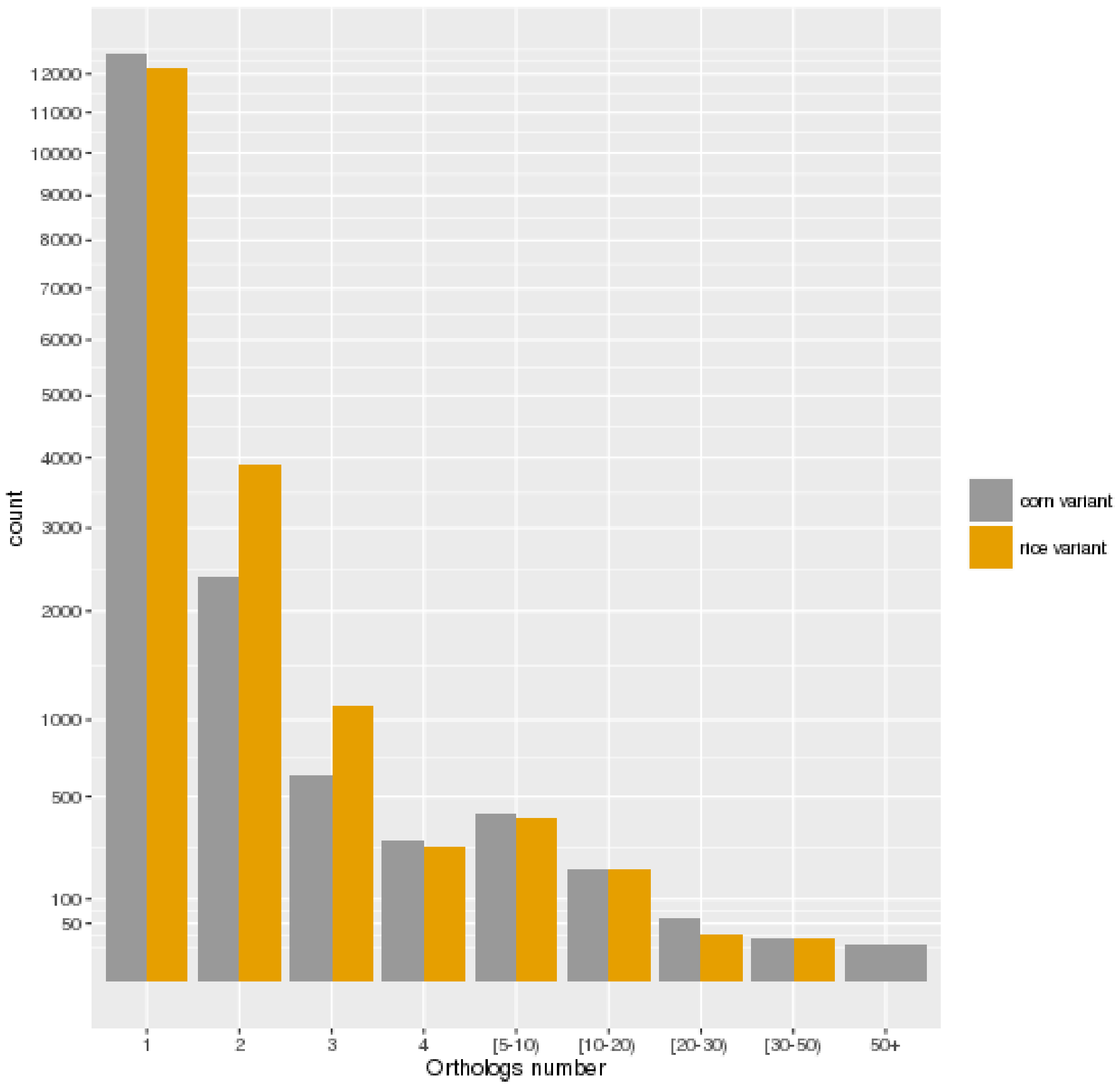


**Figure S4 Number of genes with one or more orthologs for each strain.**

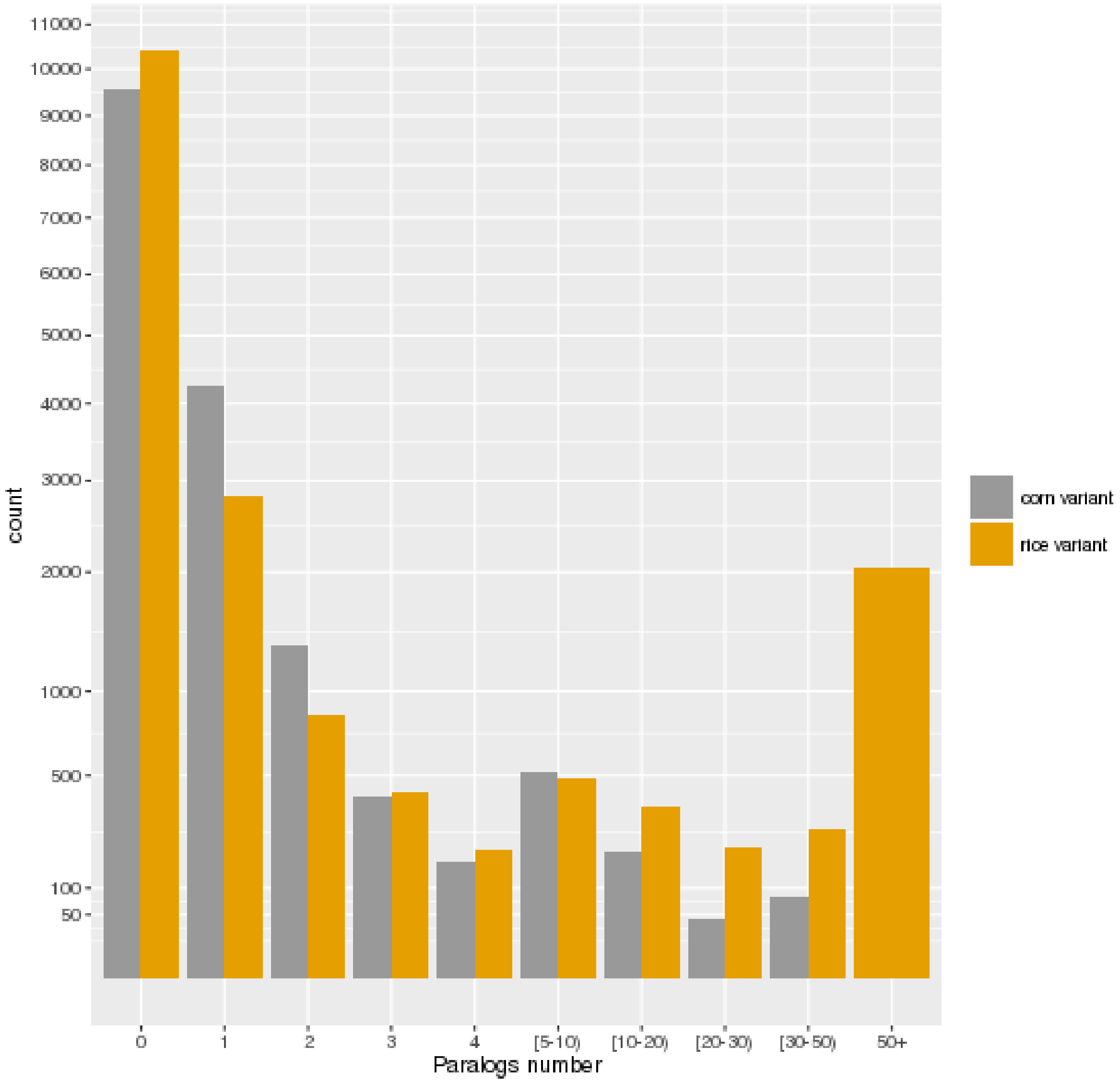


**Figure S5 Number of genes having no or more paralogs in each strain.**

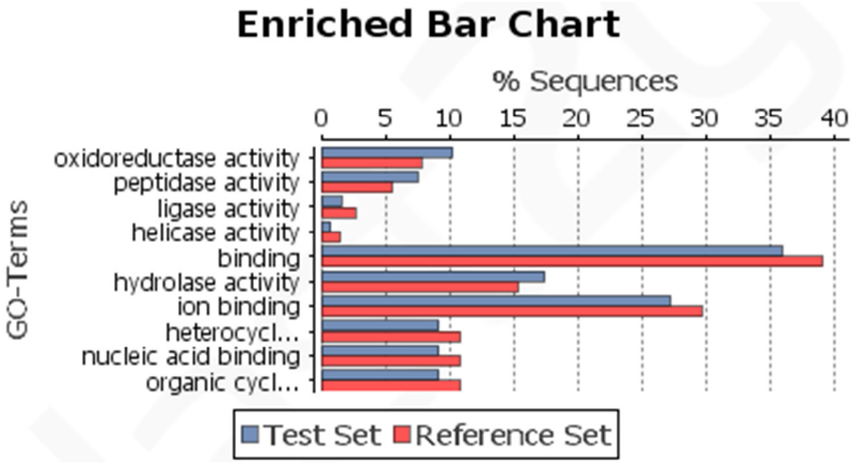


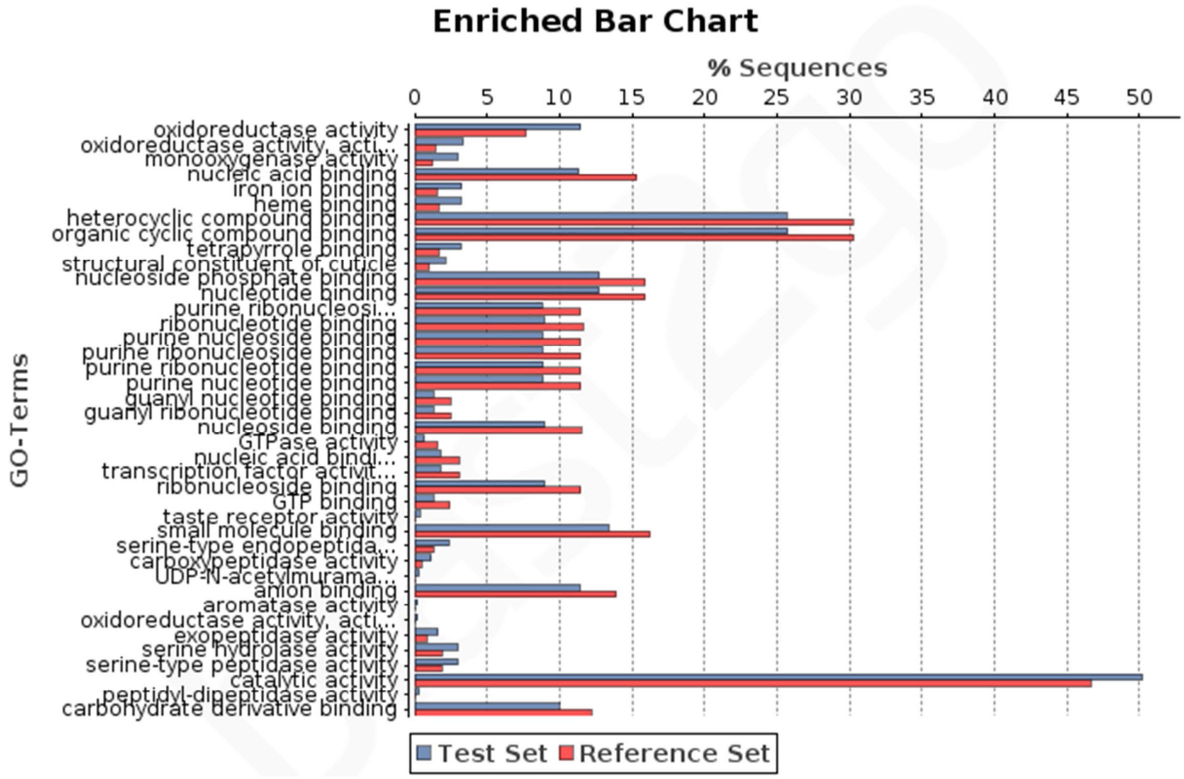


**Figure S6 GO enrichment of genes spanning rearrangements.**
Top panel in corn strain, Bottom panel in rice strain.

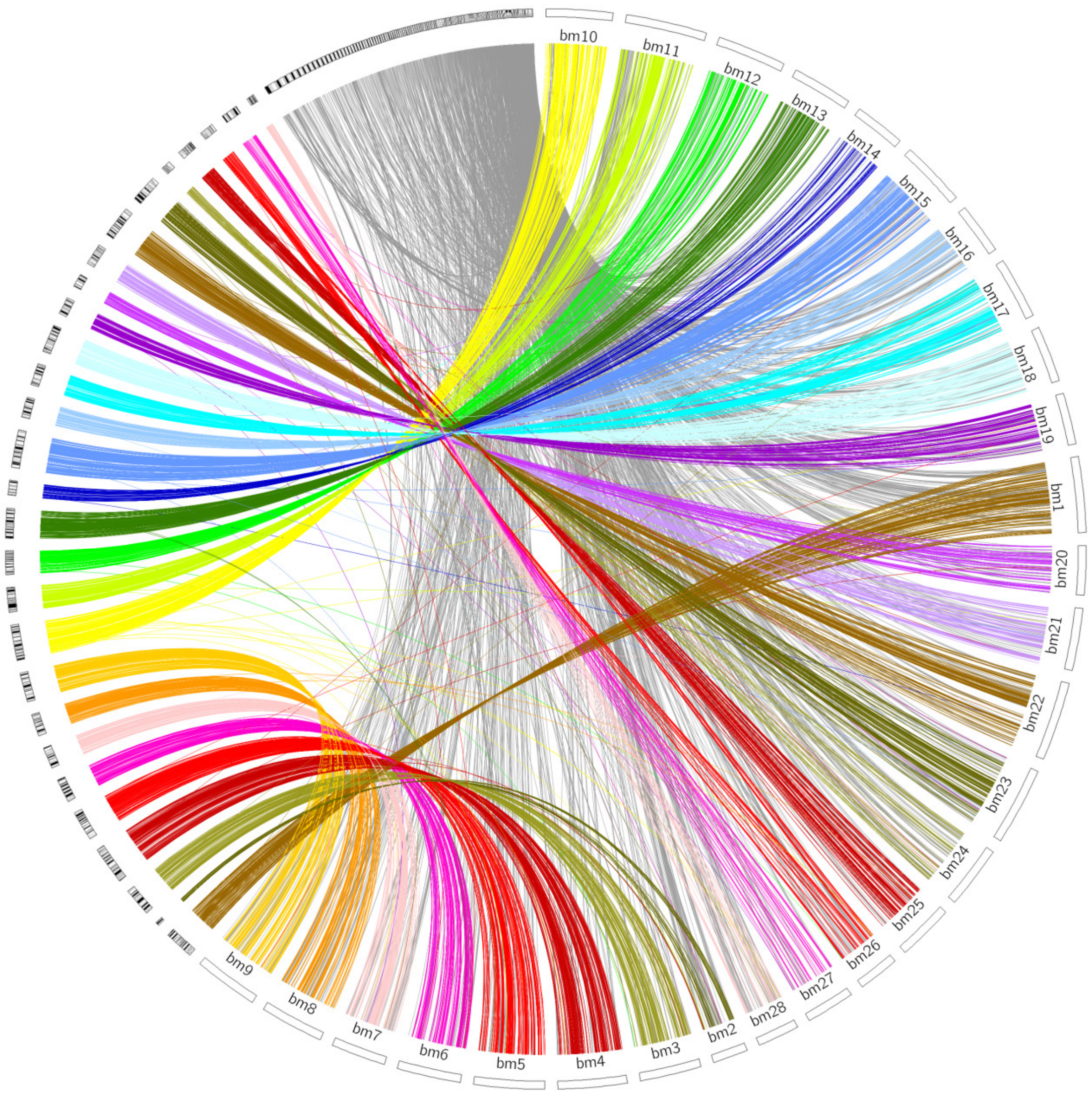


**Figure S7 Synteny with *Bombyx mori* chromosomes.**

Pseudoscaffolds resulting from the reference guided assembly and containing at least two orthologs in the same order and orientation on *Bombyx* chromosomes were anchored (coloured links). The ones that contained only one ortholog gene with *Bombyx* are shown with links in grey. The results of the reference guided assembly and the correspondence with *Bombyx* chromosomes are available on a browser at the following address, by clicking on the "synteny" button:
http://bipaa.genouest.org/is/lepidodb/spodoptera_frugiperda/

m

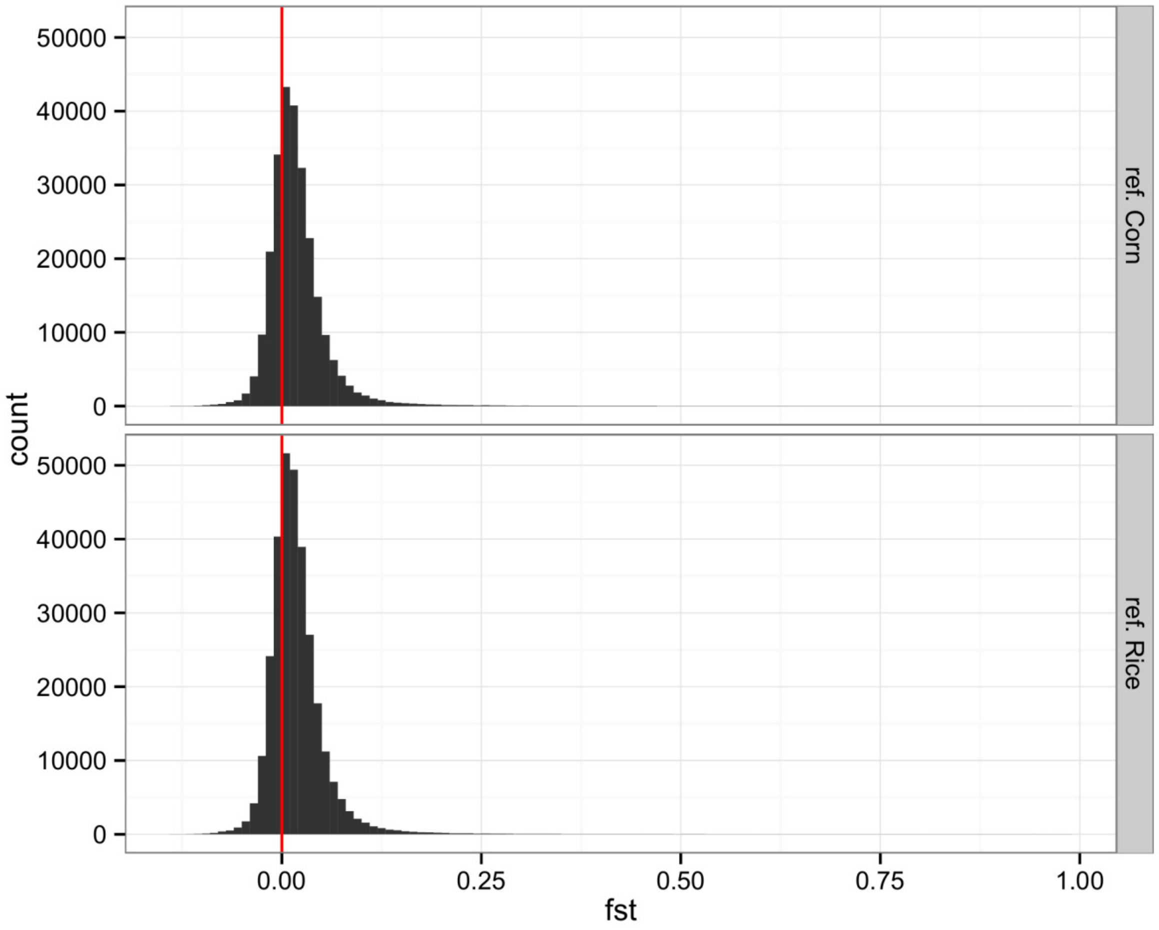


**Figure S8 The genomic differentiation between strains.**

The histogram shows the distribution of Fst calculated from 1kb windows using either corn or rice reference genomes. The vertical red line indicates when Fst equals to zero, an expectation that there is no genetic differentiation between corn and rice strains.

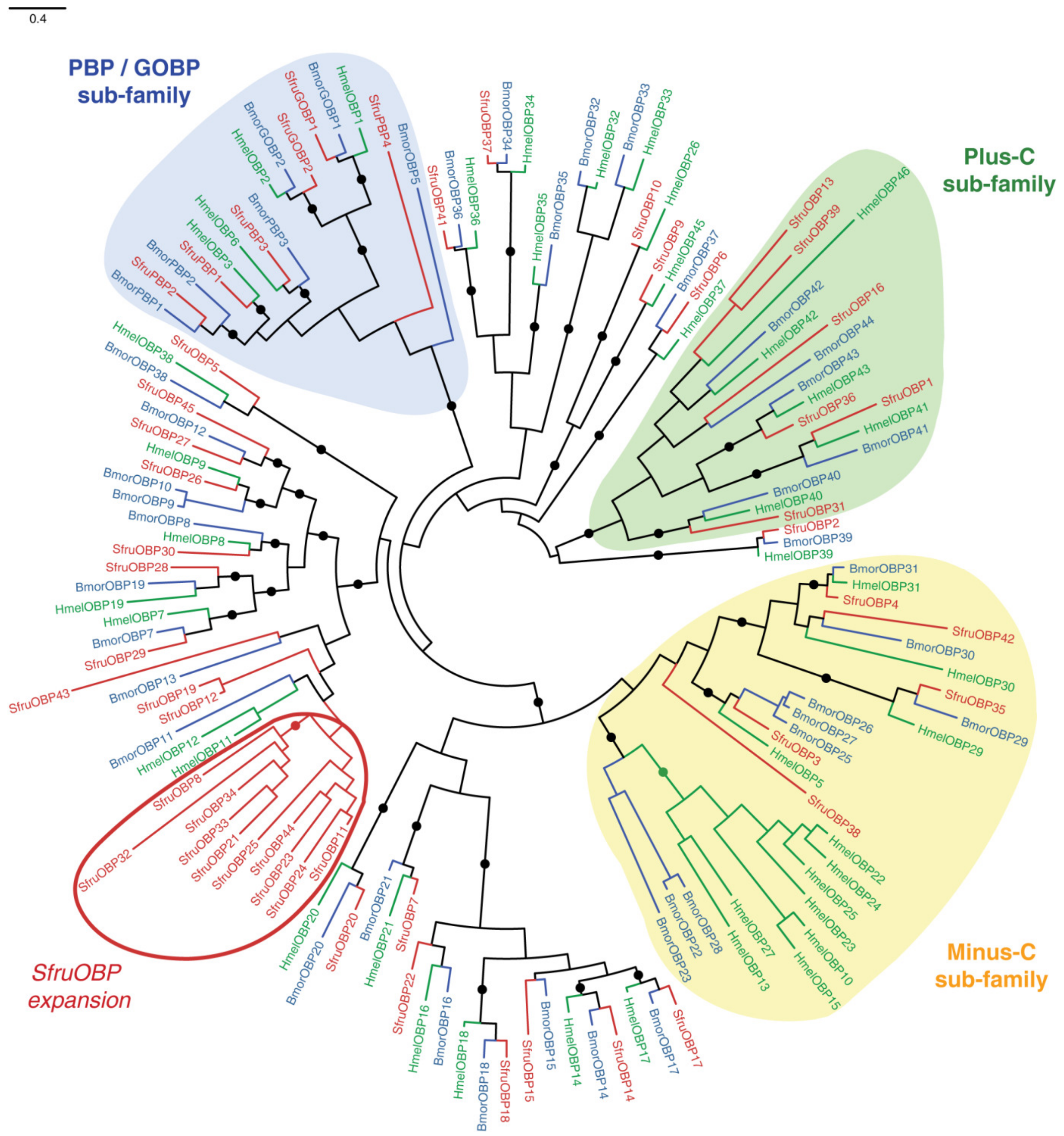


**Figure S9 Unrooted maximum-likelihood phylogeny of lepidopteran OBPs.** The amino-acid dataset included OBP repertoires from *S. frugiperda* (Noctuoidea, red), *B. mori* (Bombycoidea, blue) and *H. melpomene* (Papilionoidea, green). Circles indicate basal nodes supported by the approximate likelihood ratio-test (aLRT >0.9).

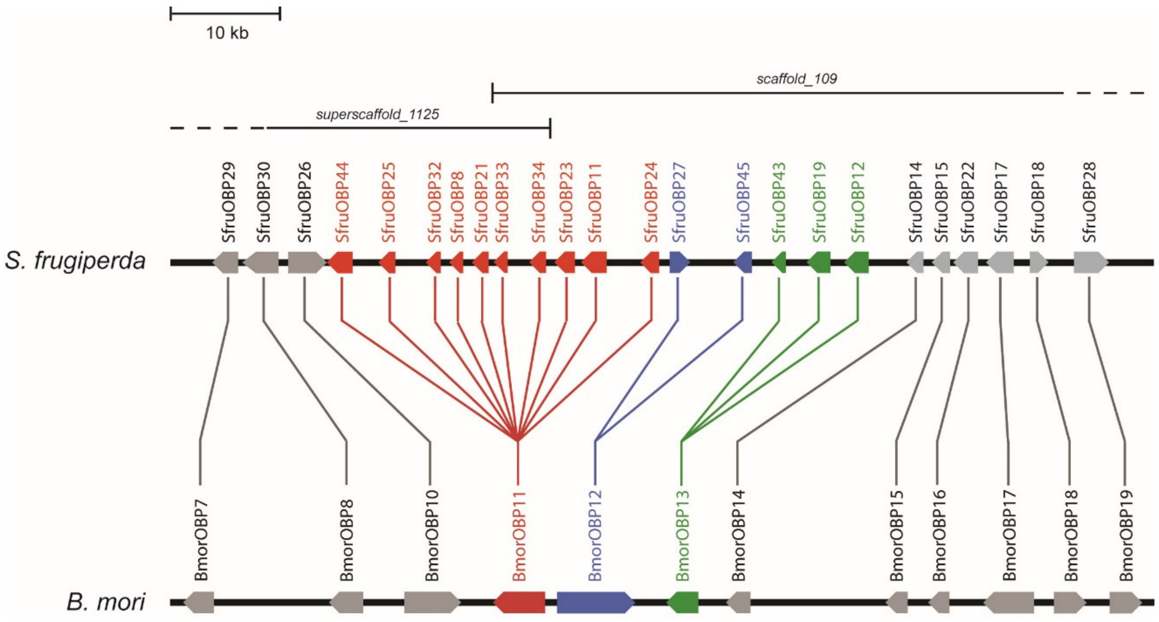


**Figure S10 Comparison of synteny among clusters of OBP genes in *S. frugiperda* and *B. mori*.**

Position and orientation (arrows) of genes within the scaffolds are indicated.

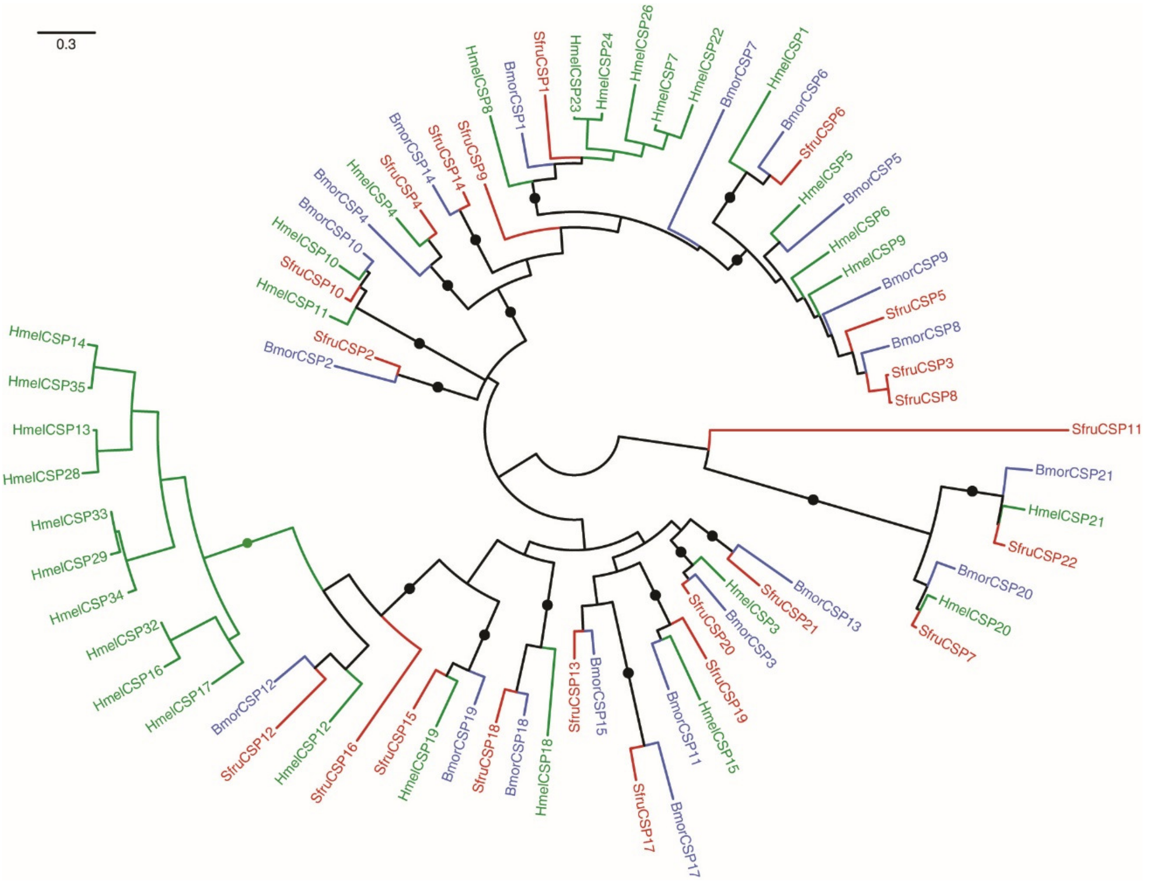


**Figure S11 Unrooted maximum-likelihood phylogeny of lepidopteran CSPs.**
The amino-acid dataset included CSP repertoires from *S. frugiperda* (Noctuoidea, red), *B. mori* (Bombycoidea, blue) and H. melpomene (Papilionoidea, green). Circles indicate basal nodes supported by the approximate likelihood ratio-test (aLRT >0.9).

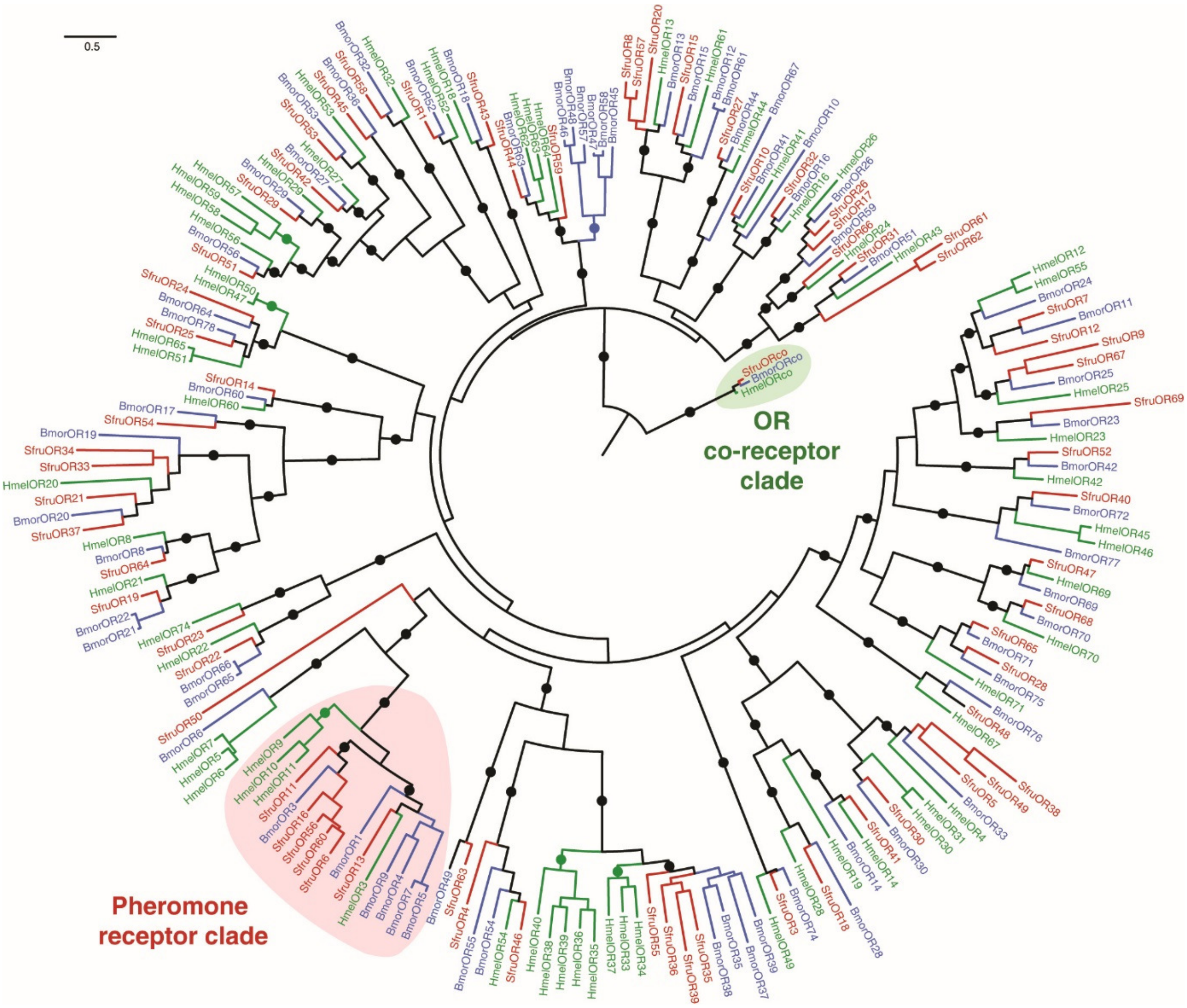


**Figure S12 Maximum-likelihood phylogeny of the lepidopteran ORs.**

The amino-acid dataset included OR repertoires from *S. frugiperda* (Noctuoidea, red), *B. mori* (Bombycoidea, blue) and *H. melpomene* (Papilionoidea, green). The tree was rooted using the OR co-receptor clade as the out-group. Circles indicate basal nodes supported by the approximate likelihood ratio-test (aLRT >0.9).

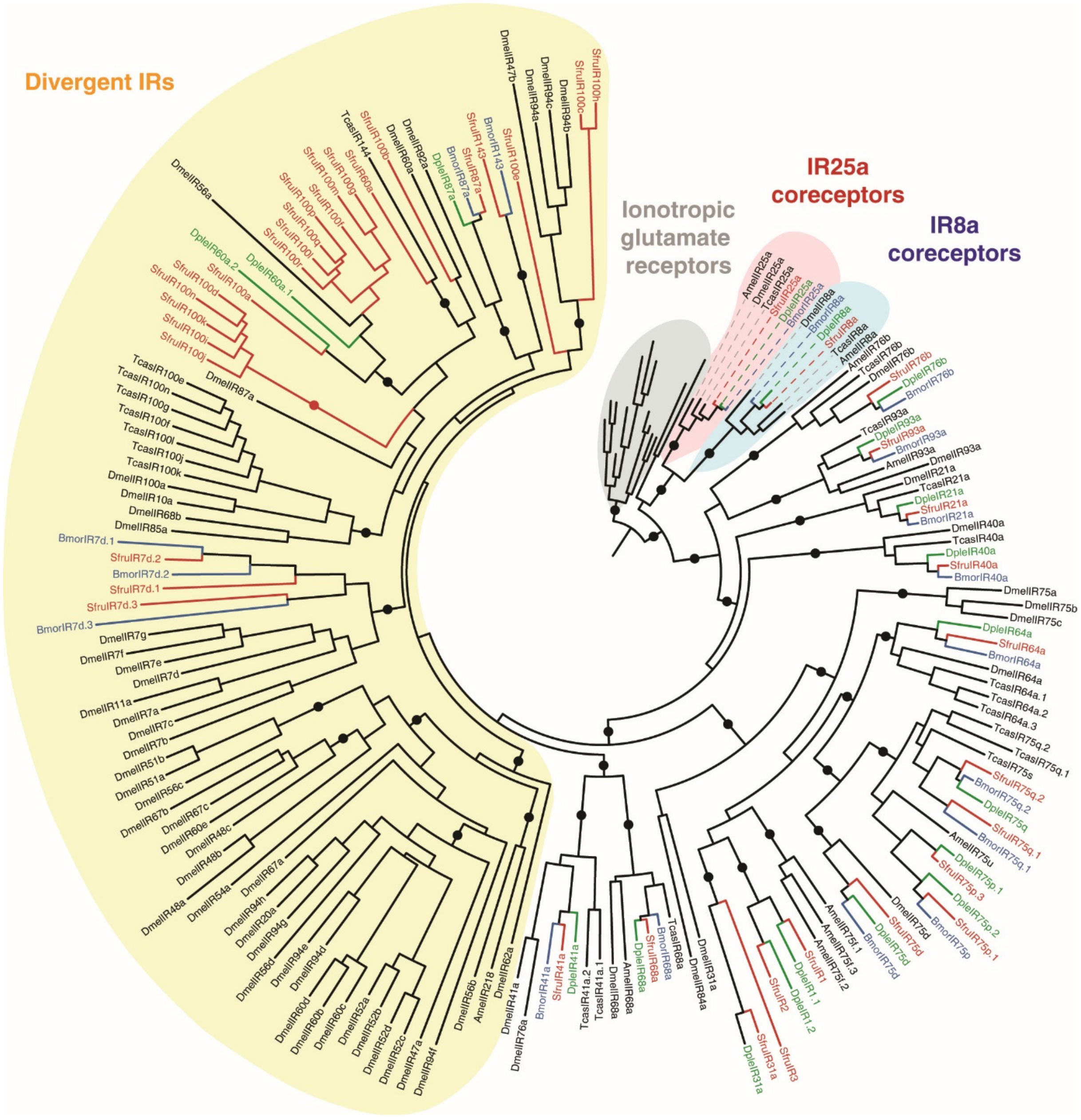


## Figure S13 Maximum-likelihood phylogeny of insect IRs.

The amino-acid dataset included IR repertoires from the Lepidoptera *S. frugiperda* (Noctuoidea, red), *B. mori* (Bombycoidea, blue) and *D. plexippus* (Papilionoidea, green), plus IR repertoires from *D. melanogaster*, *T. castaneum* and *A. mellifera*. The tree was rooted using the *D. melanogaster* ionotropic glutamate receptor clade as the out-group. Circles indicate basal nodes supported by the approximate likelihood ratio-test (aLRT >0.9).

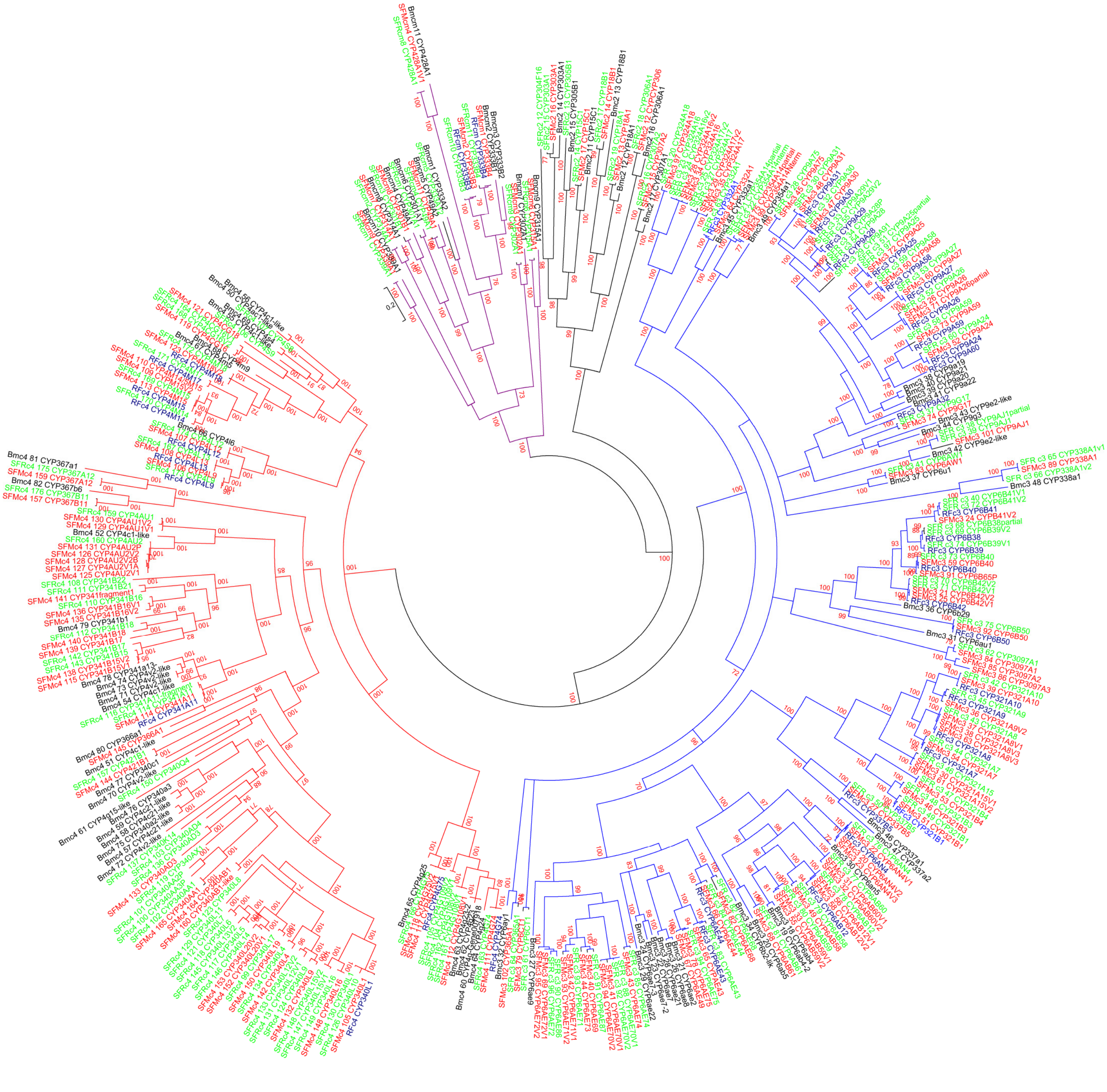

**Figure S14 Phylogenetic analysis of CYP.**

390 CYP peptidic sequences with sequence length of 282 amino-acids were used in tree inference using Bayesian method (detailed in supplementary Note S12.1.1). In black, sequences from *Bombyx mori* ; in green, from *S. frugiperda*, Rice strain, in red from *S. frugiperda* Corn strain (this study), in blue, P450 genes described before this study[119] belonging to *S. frugiperda*, Corn strain.

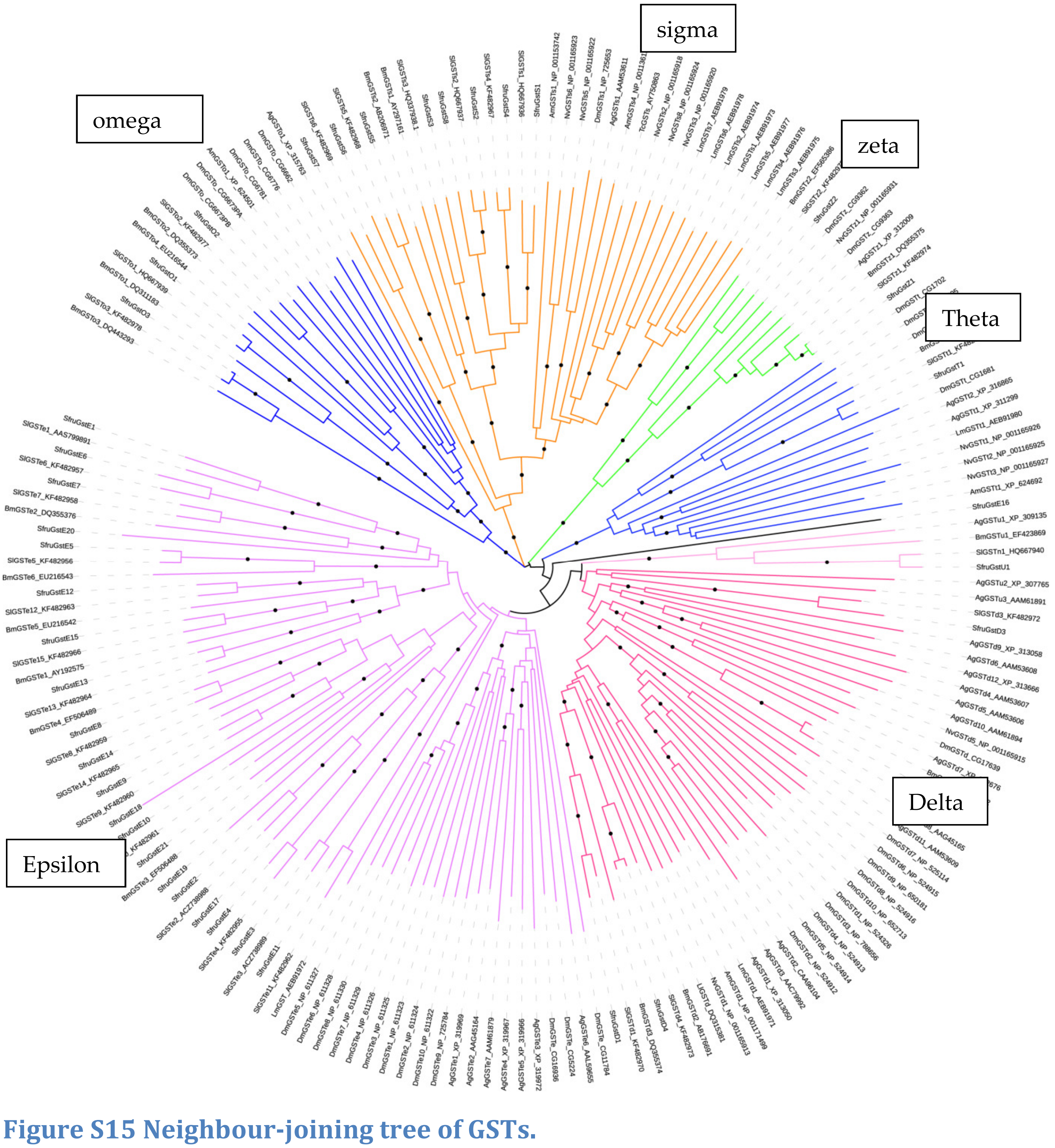


**Figure S15 Neighbour-joining tree of GSTs.**

*S. frugiperda* (sfru), *B. mori* (Bm), *S. litura* (Sl), *D. melanogaster* (Dm), *A. gambiae* (Ag), *A. mellifera* (Am), *N. vitripennis* (Nv), *L. migratoria* (Lm) and *T. castaneum* (Tc) GSTs. Node support was assessed by carrying out a bootstrap analysis with 1000 replicates.

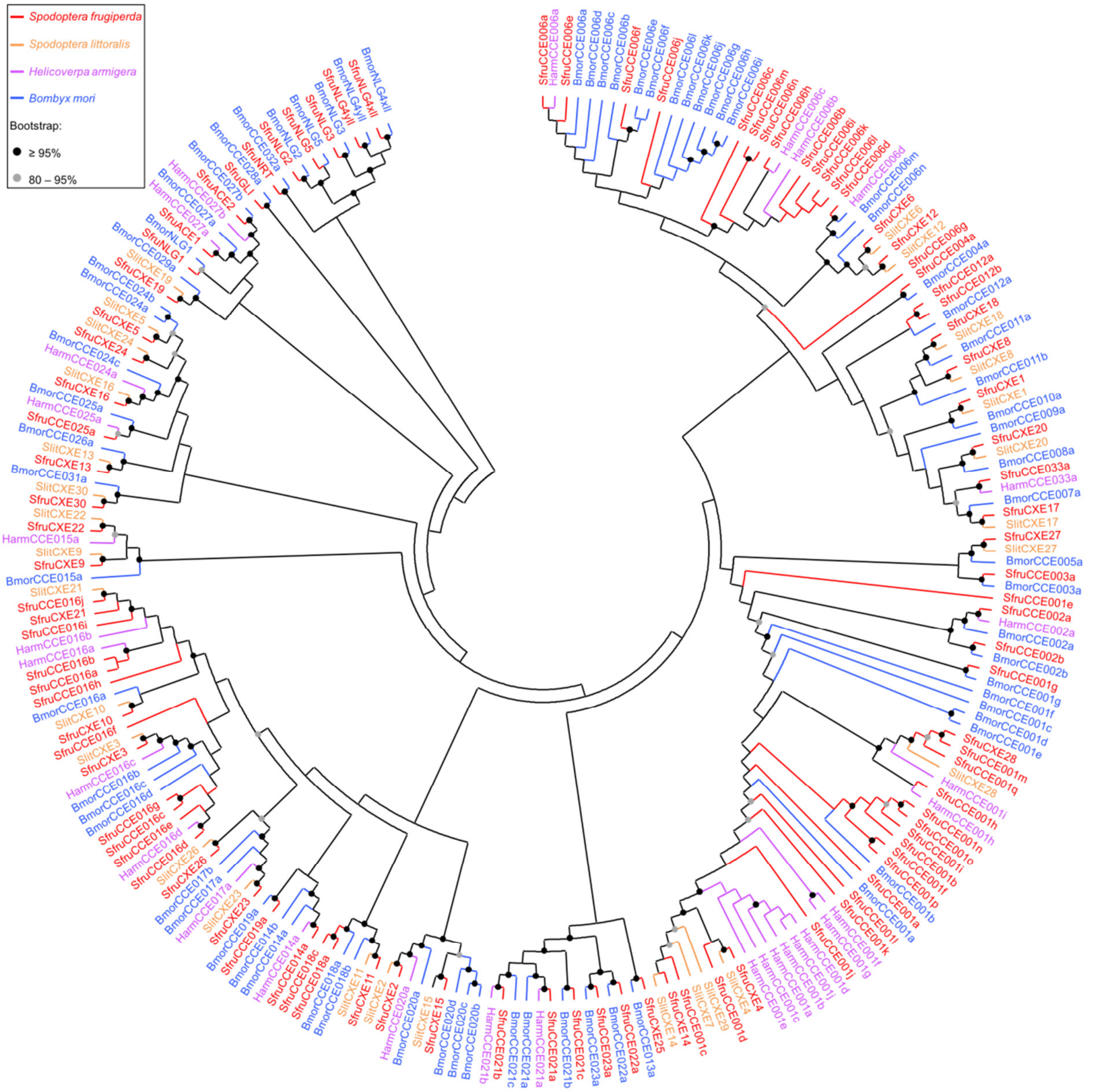


**Figure S16 Phylogeny of lepidopteran esterases.**

*S. frugiperda* CCEs are represented in red.

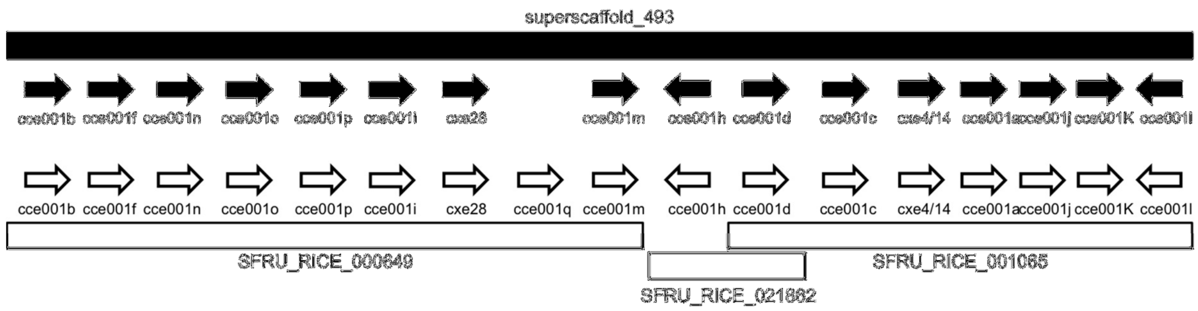


**Figure S17 Comparison between the two *S. frugiperda* strains of the genomic organization of CCE genes from the clade 001 cluster**

Scaffolds and genes are indicated in black for the corn strain and in white for the rice strain.

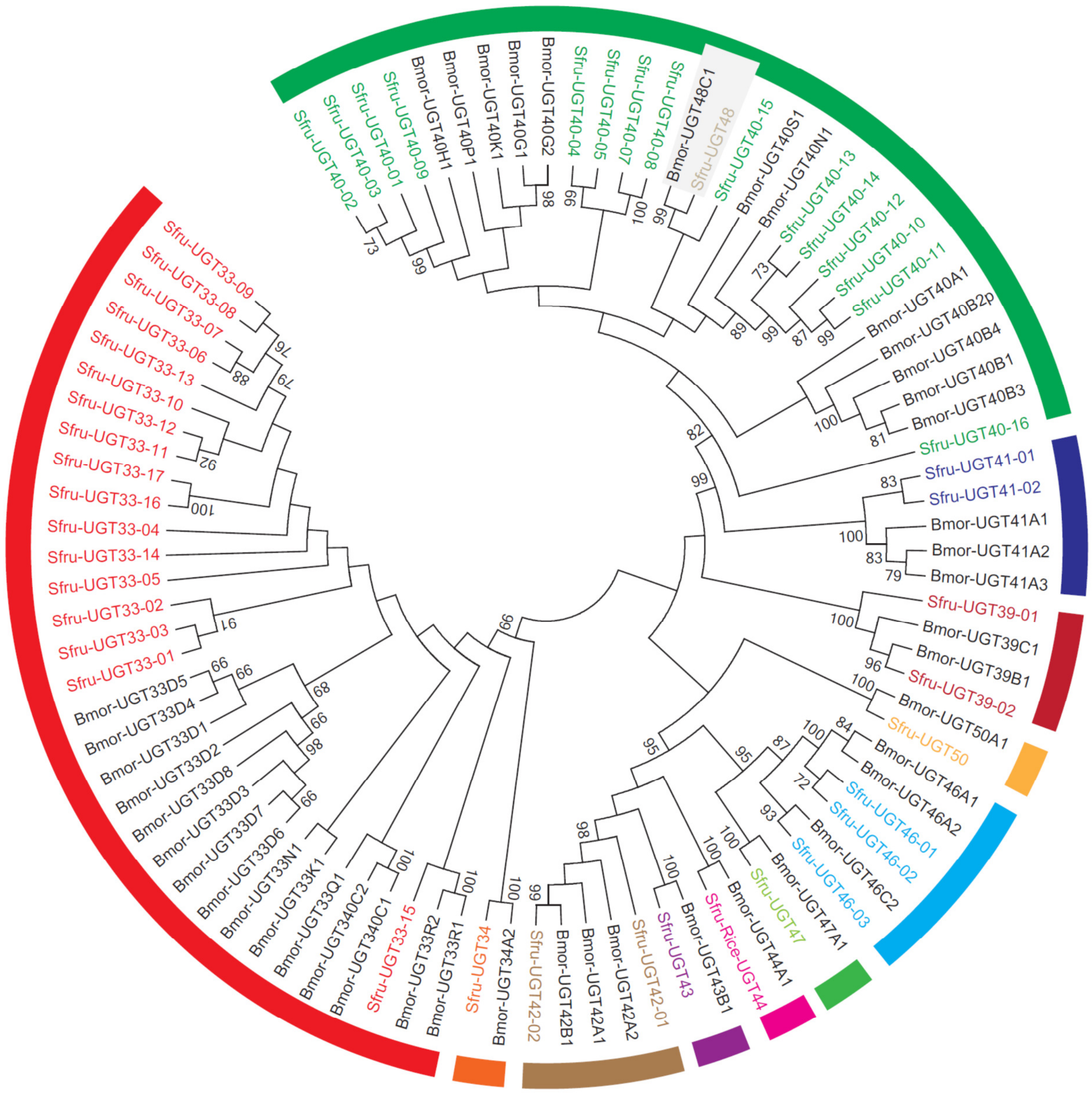


**Figure S18 A consensus Maximum-likelihood tree of the deduced amino acid sequences of UGTs from Spodoptera frugiperda and Bombyx mori.**
UGTs belonging to same gene family are depicted in same color in *S. frugiperda*.

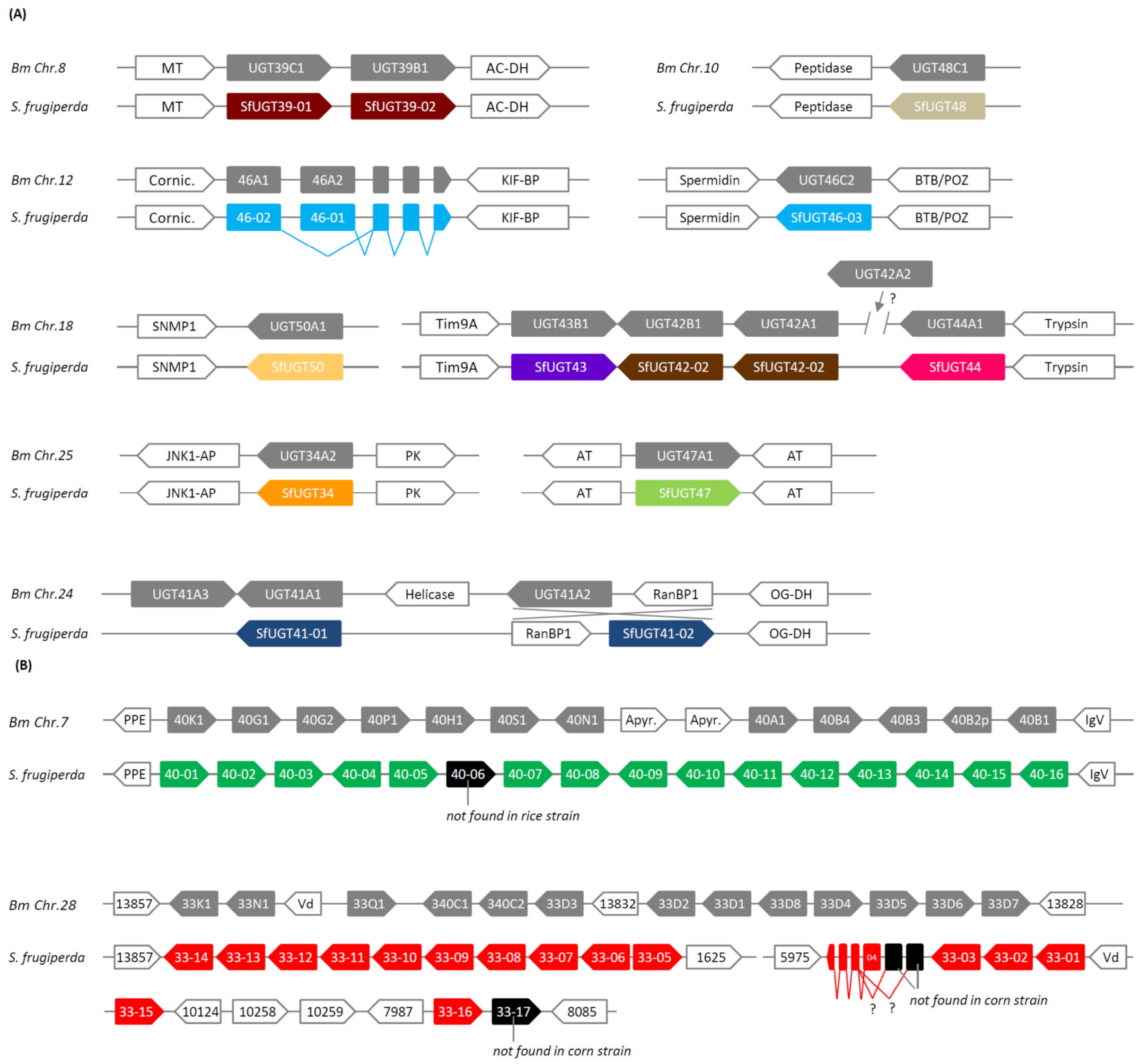


## Figure S19 Genomic position and orientation of the Spodoptera frugiperda and Bombyx mori UGTs.

(A) Inter-specific conservation of the UGT microsynteny. (B) Lineage-specific gene expansions in the UGT40 and UGT33 families.

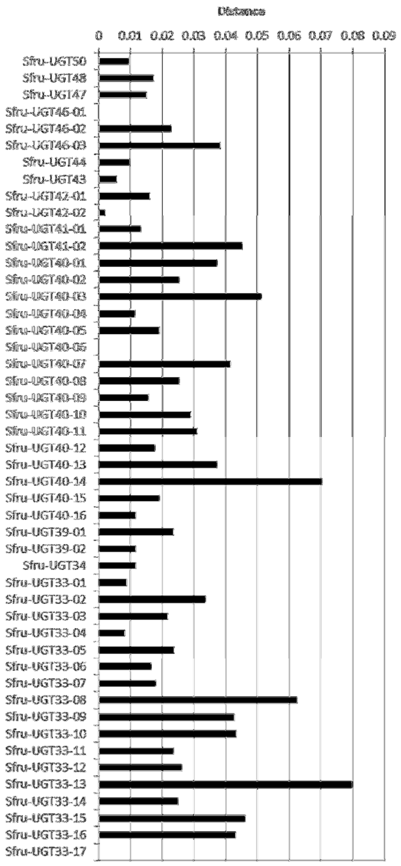


## Figure S20 Comparison of UGT amino acid sequences between the rice and corn strains.

The number of amino acid substitutions per site from between sequences are shown from 94 sequences. The level of substitution was estimated based on the JTT distance matrix using the MEGA5 software. All ambiguous positions were removed for each sequence pair.

**1**- 500 bp DNA ladder
**2**- C CYP340L10 (528bp)
**3**- R CYP340L10 (528bp)
**4**- C CYP6AE86 (1413bp)
**5**- R CYP6AE86 (1413bp)
**6**- C CYP6AE87 (967 bp)
**7**- R CYP6AE87 (967 bp)
**8**- 500 bp DNA ladder
**9**- C CXE15 (486 bp)
**10**- R CXE15 (486 bp)
**11**- C GST8 (150 bp, positive control)
**12**- R GST8 (150 bp, positive control)
**13**- 50 bp DNA ladder
14- 1 kb ladder
15- C UGT33-17 (1564 bp)
16- R UGT33-17 (1564 bp)
17- negative control
18- C UGT40-06 (788 bp)
19-R UGT40-06 (788 bp)
20-negative control
21- C UGT40-06 (416 bp)
22-R UGT40-06 (416 bp)
23- negative control
24- 1 kb ladder

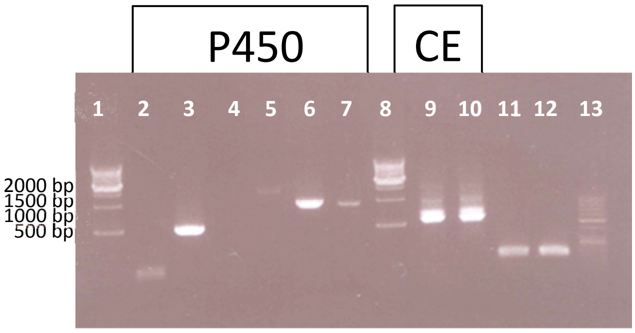


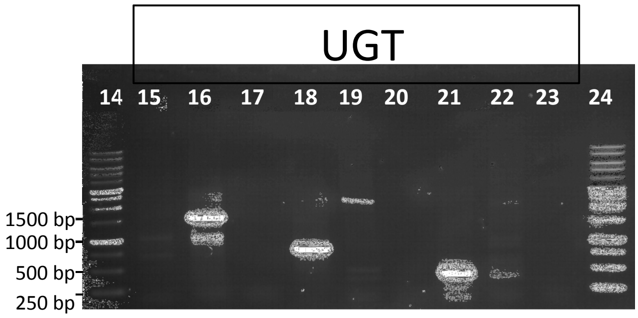


## Figure S21 Experimental validation of variation in detoxification gene repertoire between C and R strain.

Specific primers were designed for PCR amplification of the detoxification genes listed above (Supplementary Note S12.6). These genes were chosen because they had been found by annotators in only one of the two C or the R genome assemblies (except GST8, a positive control). A specific amplification band was found in lanes 3 and 5 from amplification of R strain genomic DNA as template, which confirmed that genes *CYP340L10* and *CYP6AE86* are specific of the R strain. An amplification band was found for *UGT33-17* in rice strain (lane 16) but not in corn strain (lane 15). An amplification band was found with two primer pairs for *UGT40-06* in corn strain (lanes 18,21) but not in rice strain (lanes 19,22) showing that *UGT33-17* and *UGT40-06* are respectively specific of rice or corn strain.

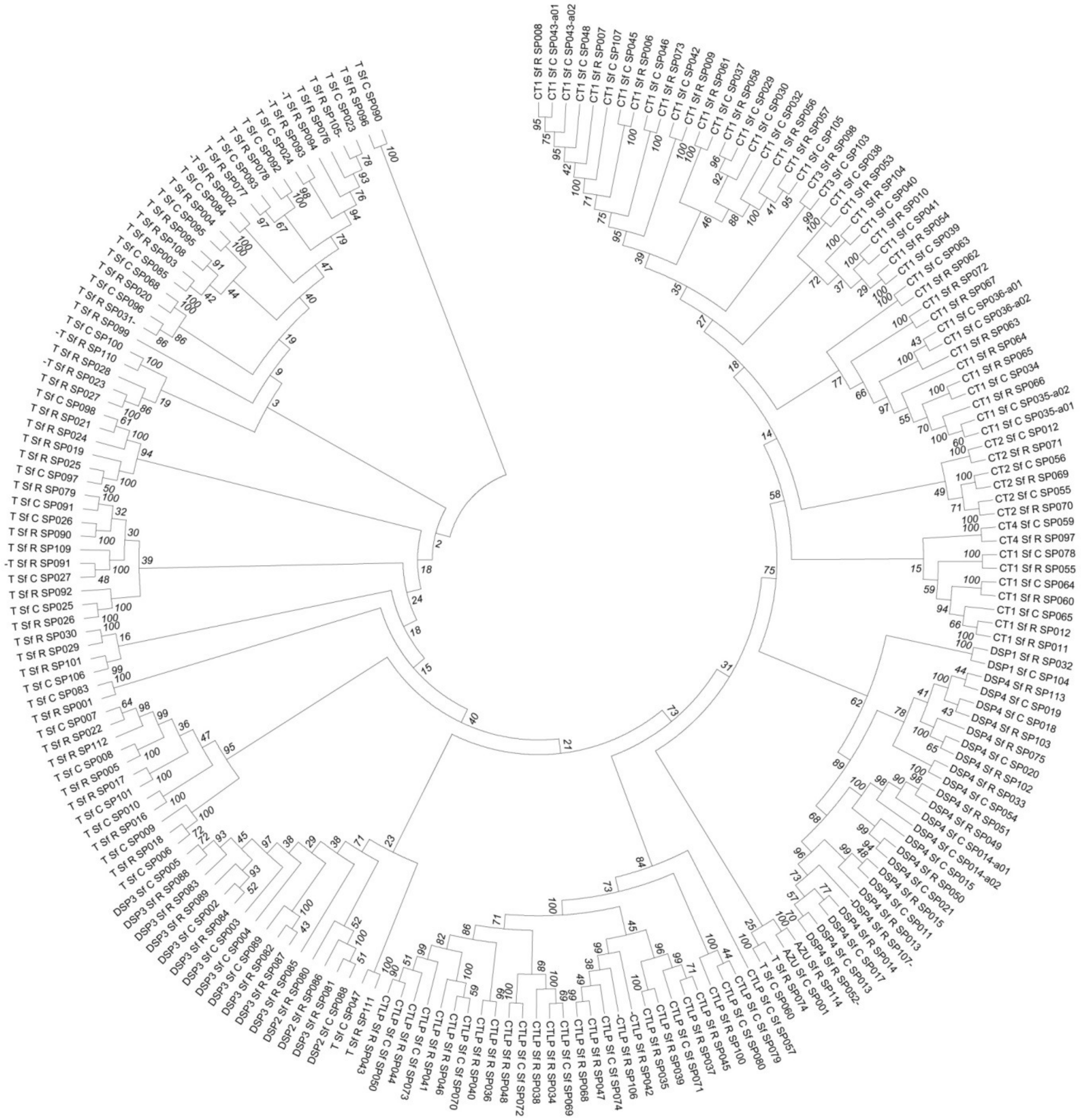

**Figure S22 Molecular phylogenetic analysis of lepidopteran Serine Proteases.**

The evolutionary history was inferred by using the Maximum Likelihood method based on the JTT matrix-based model [282]. The bootstrap consensus tree inferred from 100 replicates [283] is taken to represent the evolutionary history of the taxa analyzed [283]. Branches corresponding to partitions reproduced in less than 50% bootstrap replicates are collapsed. Initial tree(s) for the heuristic search were obtained automatically by applying the Maximum Parsimony method. The analysis involved 199 amino acid sequences. All positions with less than 95% site coverage were eliminated. That is, fewer than 5% alignment gaps, missing data, and ambiguous bases were allowed at any position. There were a total of 194 positions in the final dataset. Evolutionary analyses were conducted in MEGA7 [284]. Sequence name "-" at prefix-

missing start, “-“ at suffix missing end, “-” at prefix and suffix missing start and end. The sequences were indicated with “C” and “R” before the sequence number for the corn stain and rice strain respectively.

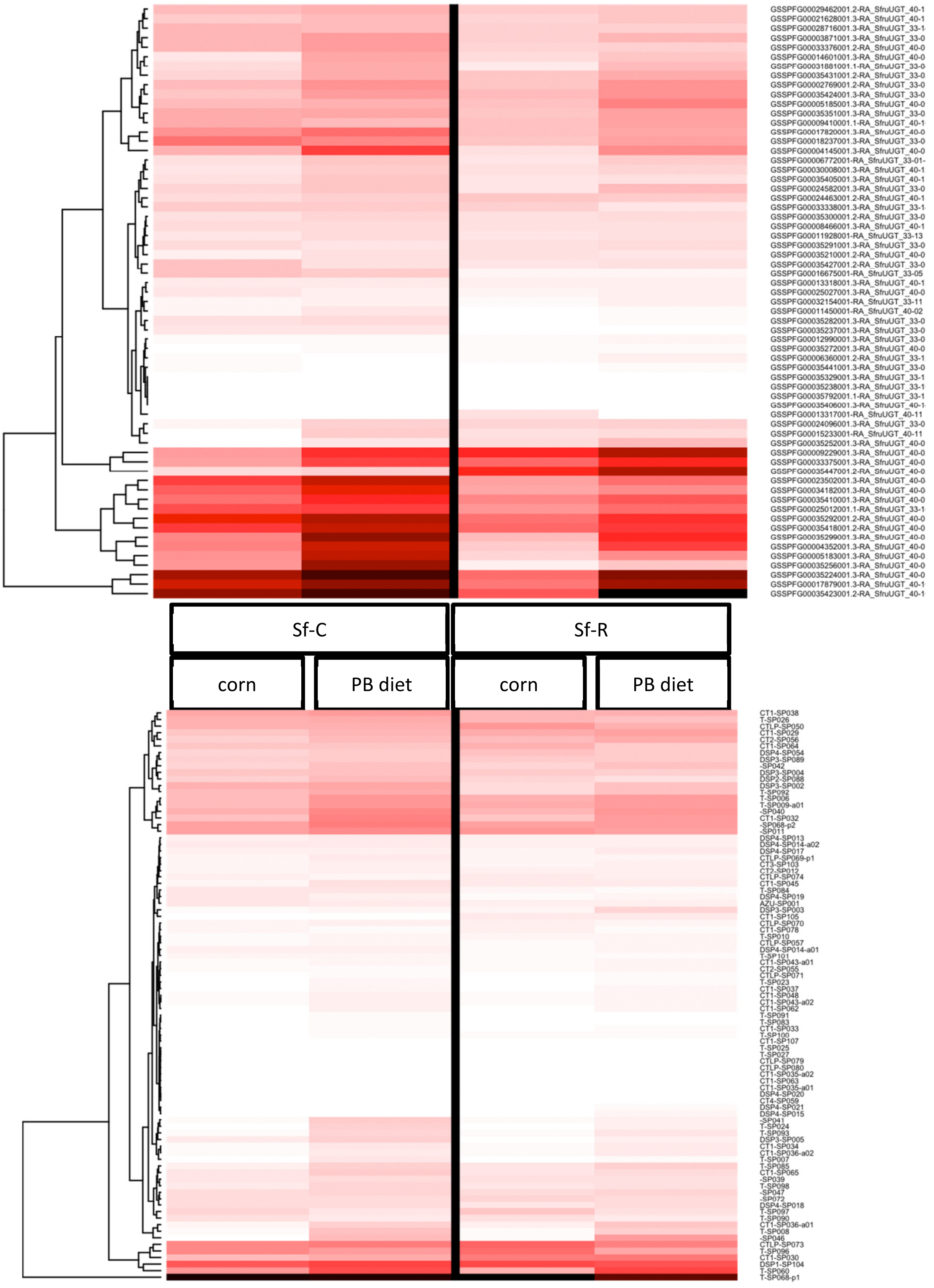


## Figure S23 Expression of UGT and serine proteases genes in the two strains reared on different diets

Heatmaps of transcription levels, expressed as normalized read counts (tpm: transcripts per millions) of the UGT (Top) and serine proteases (Bottom) families in the midgut of corn strain or rice stain larvae fed either on corn leaves or Pinto bean based artificial diet (PB diet). RNAseq data have been retrieved from Roy et al. 2015 [285] and realigned against the sf-C OGS reference using kallisto [286] Genes have been grouped (hierarchical clustering, left) based on similar expression profiles.

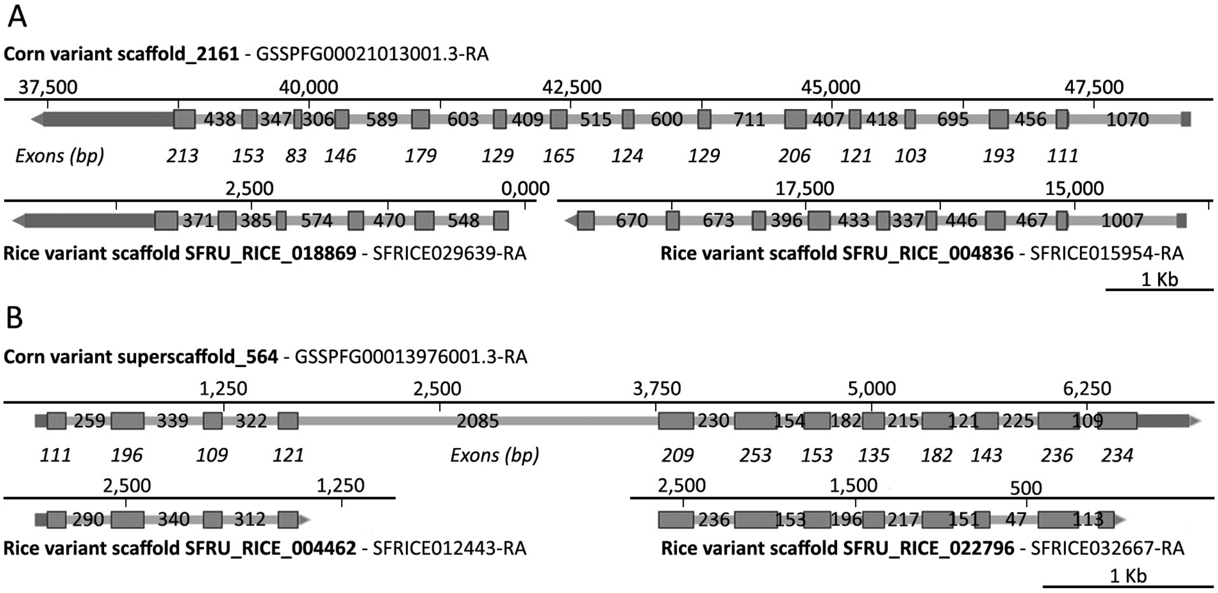


**Figure S24 Phenoloxydase genes manual annotation in corn and rice strains genomes**

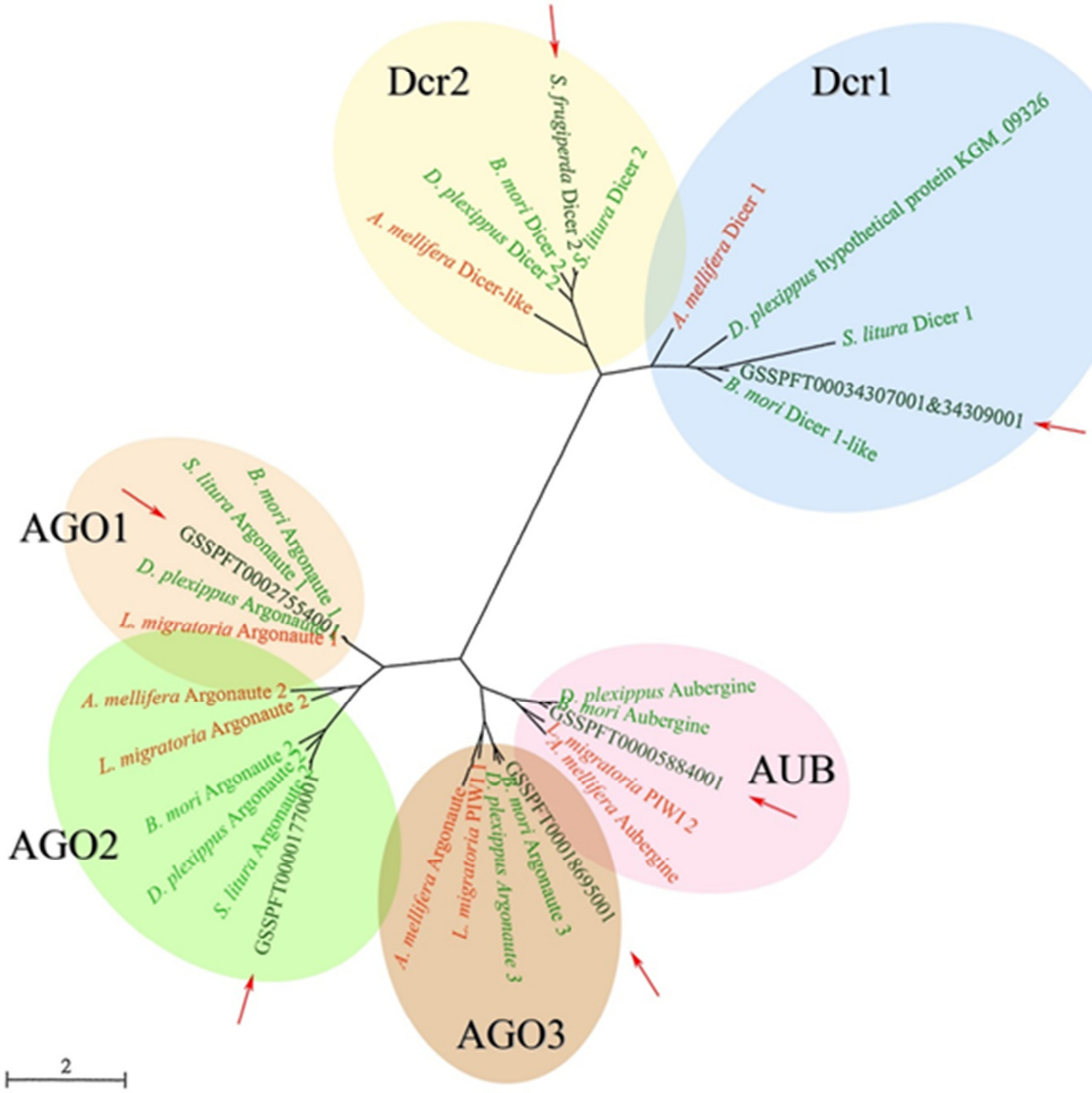


**Figure S25 Phylogenetic tree of AGO1, AGO2, AGO3, AUB, DCR1 and DCR2**

Tree scale: 0.1

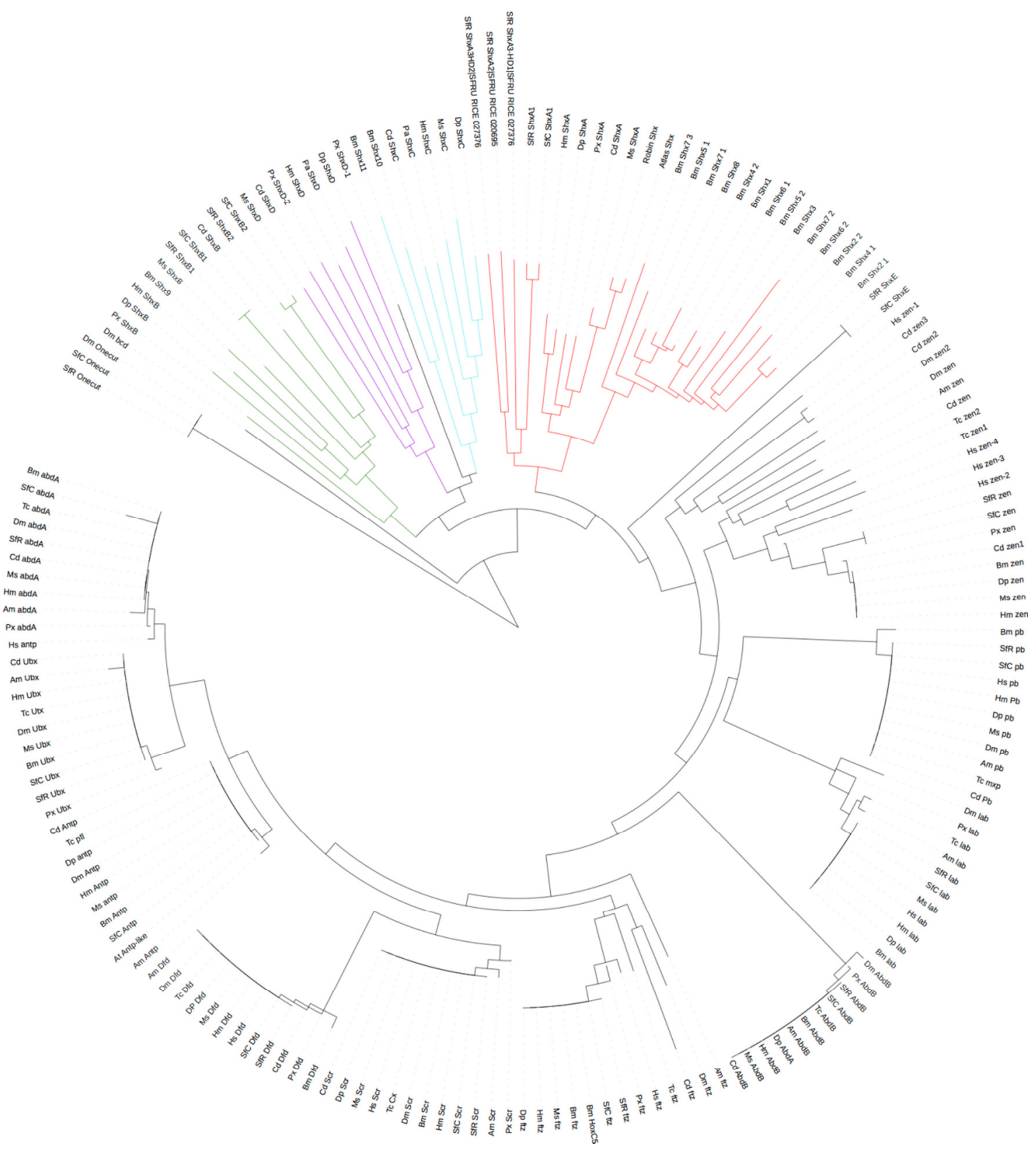

**Figure S26 Neighbour-joining tree of HoxL family homeodomains (HD) in sequenced Lepidoptera.**

The Drosophila and Spodoptera Onecut HD is used as an outgroup. Special homeobox (Shx) A, B, C and D clades are coloured in red, green, blue and purple respectively. The Lepidoptera HD have been retrieved from [203]. Species names are as follows : Dm - *Drosophila melanogaster*; SfC and SfR - *Spodoptera frugiperda* C and R strains; Bm - *Bombyx mori*; Ms - *Manduca sexta*; Cd - *Callimorpha dominula*; Hm - *Heliconius melpomene*; Dp - *Danaus plexxipus*; Px - *Plutella xylostella*; Pa - *Pararge aegeria*; Tc - *Tribolium castaneum*; Am - *Apis mellifera*; Hs - *Hepialus sylvina*; At - *Amyelois transitella*.

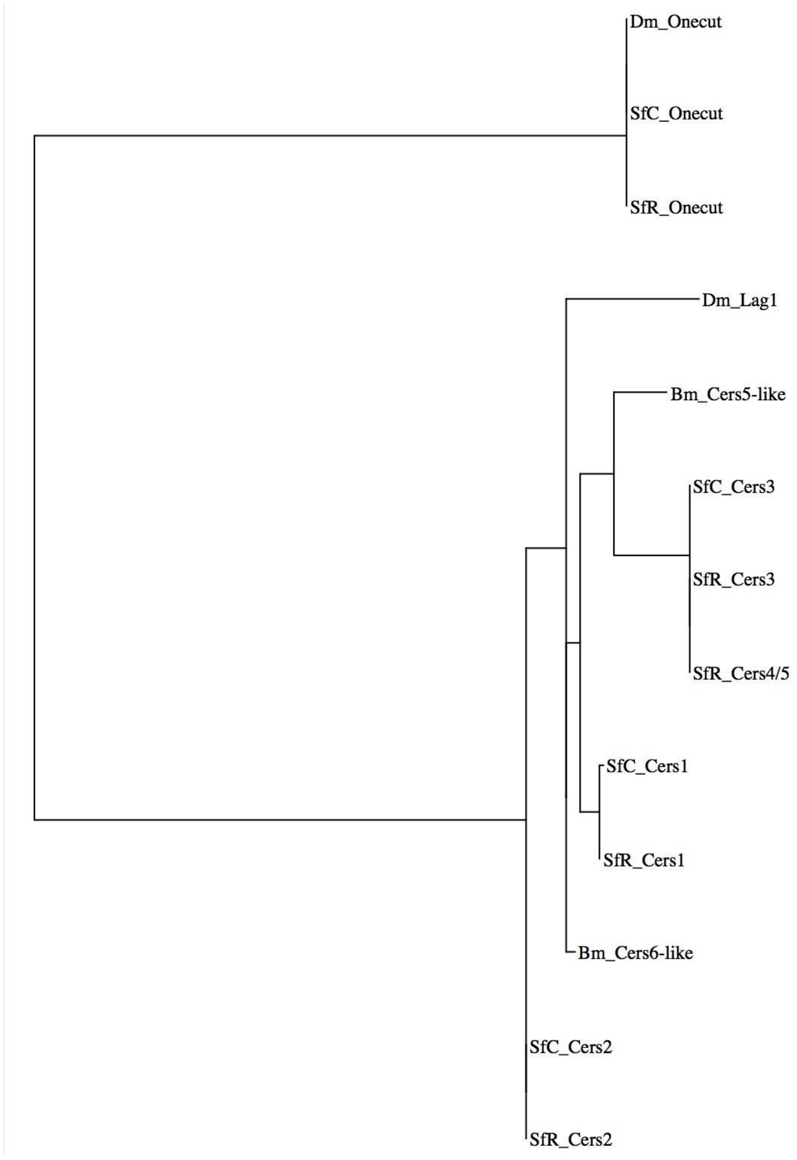


## Figure S27 Phylogenetic tree of Cers family

Neighbour-joining tree based on the alignment of HD domains for the Drosophila (Dm), Bombyx (Bm) and Spodoptera (Sf) orthologs. The Onecut HD is used as an outgroup.

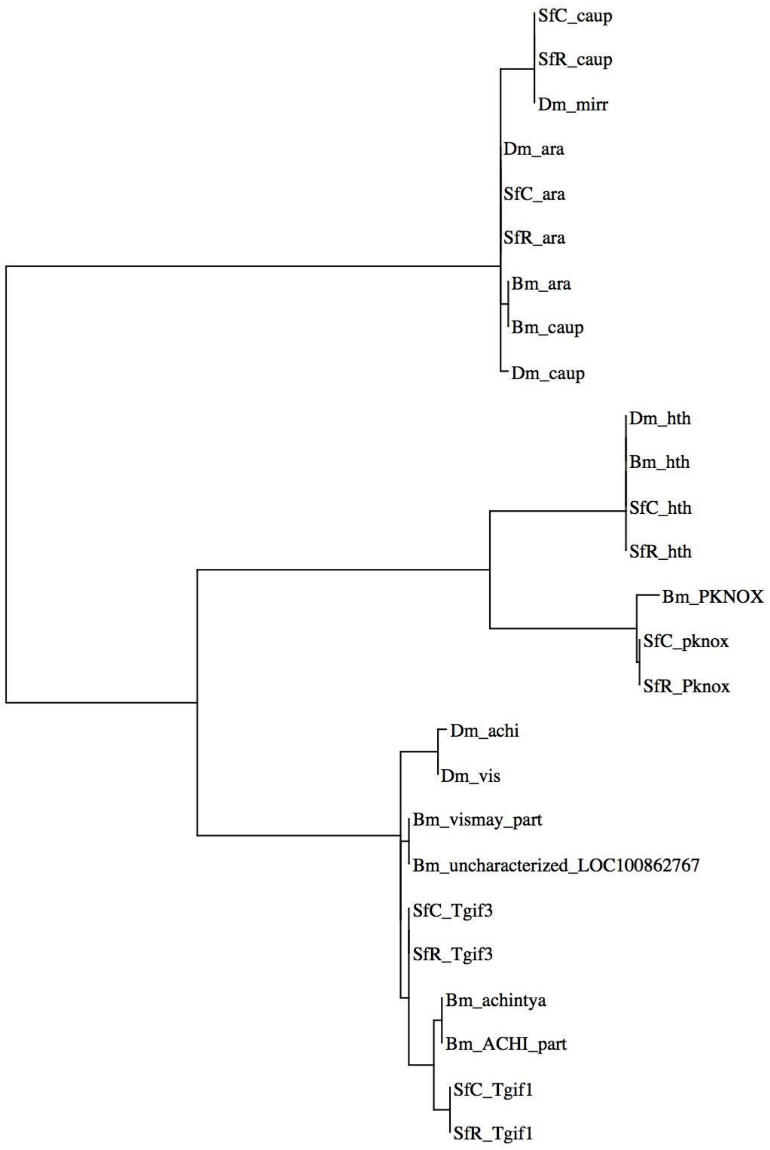


**Figure S28 Phylogenetic tree of the Irx family and Tgif family HD proteins.**
Neighbour-joining tree based on the alignment of HD domains for the Drosophila (Dm), Bombyx (Bm) and Spodoptera (Sf) orthologs. The Pknox family orthologs (hth and pknox) is used as an outgroup.

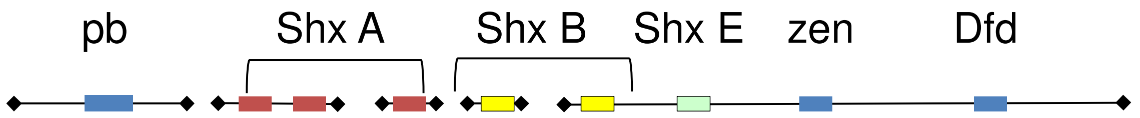


**Figure S29 The *S. frugiperda Hox3* genes cluster.**
The black line corresponds to contiguous scaffolds retrieved from aligning both SfC and SfR scaffolds. Line breaks indicate gaps in the assembly.

```
Atg8

S. frugiperda         --MKFQYKEEHSFEKRKTEGEKIRRKYPDRVPVIVEKAP-KARLGDLDKKKYLVPSDLTV   57
B. mori               --MKFQYKEEHSFEKRKAEGEKIRRKYPDRVPVIVEKAP-KARLGDLDKKKYLVPSDLTV   57
G. mellonella         --MKFQYKEEHSFEKRKTEGEKIRRKYPDRVPVIVEKAP-KARLGDLDKKKYLVPSDLTV   57
H. armigera           --MKFQYKEEHSFEKRKTEGEKIRRKYPDRVPVIVEKAP-KARLGDLDKKKYLVPSDLTV   57
P. xuthus             --MKFQYKEEHSFEKRKTEGEKIRRKYPDRVPVIVEKAP-KARLGDLDKKKYLVPSDLTV   57
D. plexippus          --MKFQYKEEHSFEKRKTEGEKIRRKYPDRVPVVVEKAP-KARLGNLDKKKYLVPSDLTV   57
D. melanogaster       --MKFQYKEEHAFEKRRAEGDKIRRKYPDRVPVIVEKAP-KARIGDLDKKKYLVPSDLTV   57
H. sapiens[GABARAP]   --MKFQYKEDHPFEYRKKEGEKIRKKYPDRVPVIVEKAP-KARVPDLDKRKYLVPSDLTV   57
H. sapiens[MAPLC3]    MPSDRPFKQRRSFADRCKEVQQIRDQHPSKIPVIIERYKGEKQLPVLDKTKFLVPDHVNM   60
                         .  :*: : *  *  * ::** ::*.::**::*:   : ::  *** *:***..:.:

S. frugiperda         GQFYFLIRKRIHLRPEDALFFFVNNV-IPPTSATMGSLYQEHHDEDFFLYIAFSDENVYG   116
B. mori               GQFYFLIRKRIHLRPEDALFFFVNNV-IPPTSATMGSLYQEHHDEDFFLYIAFSDENVYG   116
G. mellonella         GQFYFLIRKRIHLRPEDALFFFVNNV-IPPTSATMGSLYQEHHDEDFFLYIAFSDENVYG   116
H. armigera           GQFYFLIRKRIHLRPEDALFFFVNNV-IPPTSATMGSLYQEHHDEDFFLYIAFSDENVYG   116
P. xuthus             GQFYFLIRKRIHLRPEDALFFFVNNV-IPPTSATMGSLYQEHHDEDFFLYIAFSDENVYG   116
D. plexippus          GQFYFLIRKRIHLRPEDALFFFVNNV-IPPTSATMGSLYQEHHDEDFFLYIAFSDENVYG   116
D. melanogaster       GQFYFLIRKRIHLRPEDALFFFVNNV-IPPTSATMGSLYQEHHEEDYFLYIAYSDENVYG   116
H. sapiens[GABARAP]   GQFYFLIRKRIHLRPEDALFFFVNNT-IPPTSATMGQLYEDNHEEDYFLYVAYSDESVYG   116
H. sapiens[MAPLC3]    SELVKIIRRRLQLNPTQAFFLLVNQHSMVSVSTPIADIYEQEKDEDGFLYMVYASQETFG   120
                      .::  :**:*::*.* :*:*::**:  :  .*: :..:*::.::** ***:.::.:..:*

S. frugiperda         YY---   118
B. mori               N----   117
G. mellonella         SM---   118
H. armigera           Y----   117
P. xuthus             Y----   117
D. plexippus          Y----   117
D. melanogaster       MAKIN   121
H. sapiens[GABARAP]   K----   117
```

H. sapiens[MAPLC3] F---- 121

**Figure S30 Multiple alignments of Atg8 protein sequences in insects.** *Spodoptera frugiperda* (SFruDB:GSSPFG00035793001.1-RA), *Bombyx mori* (gi:114052412), *Galleria mellonella* (gi:400073886), *Helicoverpa armigera* (gi:389604114), *Papilio xuthus* (gi:389608575), *Danaus plexippus* (gi:357624756), Drosophila melanogaster (gi:7291184), *Homo sapiens* [GABARAP] (gi:13899219) and *Homo sapiens* [MAPLC3] (gi:14210522). Conserved glycine at position 18 (GABARAP subfamily), valine at position 20 (LC3 subfamily) and C-terminal glycine are highlighted in black, the ubiquitin-related domain is in gray.

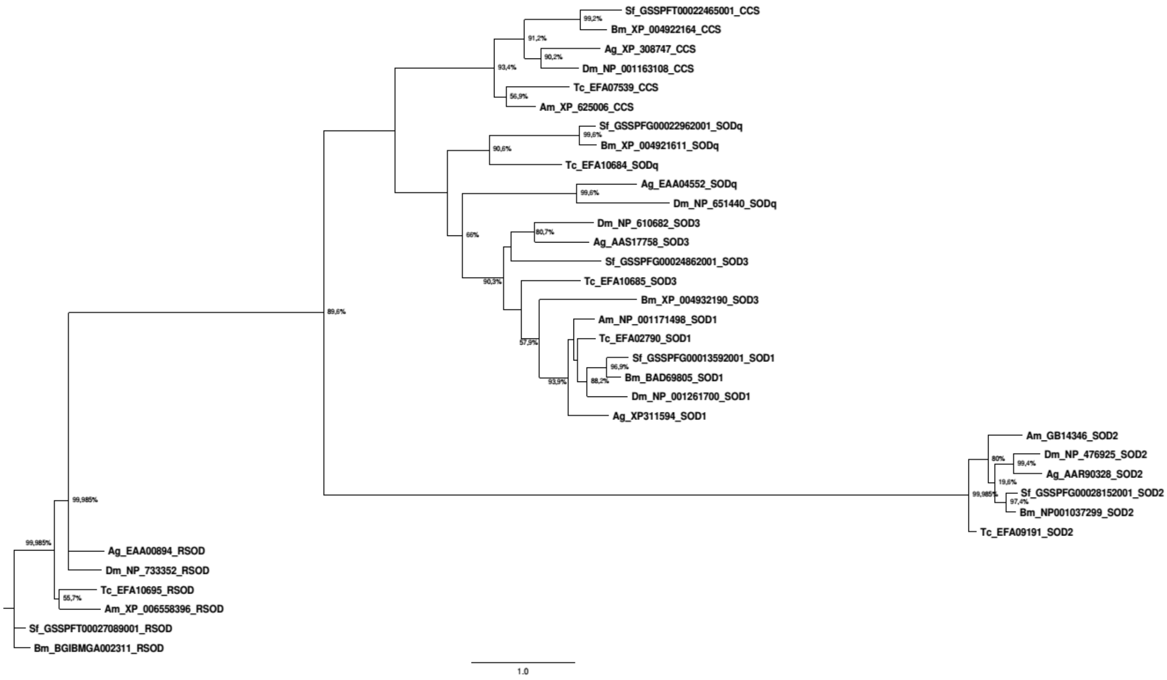


## Figure S31 Maximum likelihood tree of insect superoxide dismutase (SOD), based on the LG+G+I model.

Numbers on the nodes represent bootstrap support (1000 bootstrap) supporting the branch, only values ≥50% are shown. Each entry has a species name (Am, for *Apis mellifera*; Ag, for *Anopheles gambiae*; Bm, for *Bombyx mori*; Dm, for *Drosophila melanogaster*; Sf, for *Spodoptera frugiperda*; Tc, for *Tribolium castaneum*), accession number and protein name.

```
SfCat1                     ---------------MASRDPASDQLVNYKKNLKDSPGYITTKAGAPVGVKTAVQTVGKN 45
SfCat2                     MLRLLFLVAMAVVTAKVQDDPAANQIVIFKEKSKGPIATMTTAAGAPIEQKEATVTLNER 60
SfCat3                     ------------------------------------------------------------
Dm_CG6871                  ---------------MAGRDAASNQLIDYKNSQTVSPGAITTGNGAPIGIKDASQTVGPR 45
Ag_XP_314995               -----------------MSRNPAENQLNAYRDAQKD-KVTATMSHGAPVGTKTASETAGPR 43
Aa_XP_001663600            -----------------MSRNPAENQLNLFKESQKD-KSVATTGNGAPLGTKTATATVGER 43
Am_NP_001171540            --------------MTEIKRNPSADQLIDYKKNLKPDCPIFLTGSGTPISK-KASLTVGPN 46
Bm_NP_001036912            ---------------MASRDPATDQLINYKKTLKDSPGFITTKSGAPVGIKTAIQTVGKN 45


SfCat1                     GPTLLQDVNFLDEISAFDRERIPERVVHAKGAGAFGYFEVTHDITKYCAAKILESVGKTT 105
SfCat2                     ---LIFNEYFMDTMTHLVRERIPERLVHAKAGGAFGYFEVTHDITDICKAKLFSKVGKKT 117
SfCat3                     ------------------------------------------------------------
Dm_CG6871                  GPILLQDVNFLDEMSHFDRERIPERVVHAKGAGAFGYFEVTHDITQYCAAKIFDKVKKRT 105
Ag_XP_314995               GPVLLQDVHLIDELAHFDRERIPERVVHAKGAGAFGYFEVTHDITQYCAAKLFEKVGKKT 103
Aa_XP_001663600            GPVVLQDVHFLDEMSHFDRERIPERVVHAKGAGAFGYFEVTHDITQYCAAKVFEKVGKKT 103
Am_NP_001171540            GPILLQDYVFLDELSHFNRERIPERVVHAKGAGAFGYFEVTHDITKYSKAKVFSSIGKRT 106
Bm_NP_001036912            GPALLQDVNFLDEMSSFDRERIPERVVHAKGAGAFGYFEVTHDITKYSAAKVFESIGKRT 105


SfCat1                     PMAVRFSTVGGESGSADTVRDPRGFAVKFYTDDGNWDLVGNNTPIFFIRDASLFPSFIHT 165
SfCat2                     PIAARFSPVVVERGGIDTSRDARGFALKFYTEDGNFDIVGFNTPMYVYKDPLLFPTFVRA 177
SfCat3                     ------------------------MSIKFYTKEGNLDILCLSIPVYLYRDPMFFLNLVHA 36
Dm_CG6871                  PLAVRFSTVGGESGSADTARDPRGFAVKFYTEDGVWDLVGNNTPVFFIRDPILFPSFIHT 165
Ag_XP_314995               PLAVRFSTVGGESGSADTVRDPRGFAVKFYTDDGVWDMVGNNTPIFFIRDPVLFPSFIHT 163
Aa_XP_001663600            PLAVRFSTVGGESGSADTARDPRGFAVKFYTDDGVWDLVGNNTPIFFIRDPILFPSFIHT 163
Am_NP_001171540            PIAVRFSTVGGESGSADTVRDPRGFAVKFYTEEGVWDLVGNNTPIFFIKDPIYFPSFIHT 166
Bm_NP_001036912            PIAVRFSTVGGESGSADTVRDPRGFAVKFYTDDGVWDLVGNNTPIFFIRDPTLFPSFIHT 165
                                                   :::****.:*  *::  . *::. :*   * .::::

SfCat1                     QKRNPATHLKDPDMFWDFITLRPETTHQVLYLFGDRGIPDGYRHMNGYGSHTFKMVNAQG 225
SfCat2                     QKRNPATNLLDPNMLWDFLTLRPESLHMFLLVFGDRGIPDGYRHMPGFGIHTFQVVNKHG 237
SfCat3                     FKRNPQTQMFDFTAQWDLMTLRPVINHNLFWTFADYGIPDGYRRMDAFPIHTYELSNKHG 96
Dm_CG6871                  QKRNPQTHLKDPDMFWDFLTLRPESAHQVCILFSDRGTPDGYCHMNGYGSHTFKLINAKG 225
Ag_XP_314995               QKRNPATHLKDPDMFWDFISLRPETTHQTMFLFSDRGTPDGYRFMNGYGSHTYKLVNADG 223
Aa_XP_001663600            QKRNPATHLKDADMFWDFISLRPESTHQVMFLFADRGIPDGYRFMNGYGSHTFKLINAQG 223
Am_NP_001171540            QKRNPVTHLKDADMFWDFLSLRPESTHQVMFLFSDRGIPDGYRHMNGYGSHTFSLVNAKD 226
Bm_NP_001036912            QKRNPATHLKDPDMFWDLLTLRPETIHQLLYLFGDRGIPDGYRHMNGYGSHTFKLVNSQG 225
                            **** *.: *    **:::***   *     *.* * ****  * .:  **:.: * .

SfCat1                     VAHWVKFHYKTNQGIKNLPVEKAAELASSDPDYSIRDLYNAIAKGEFPSWTMYIQVMTMA 285
SfCat2                     DSHFIRFHFRPDAGIKNLRSEEARKLAGTDPDYATRDLYRAIGEGHYPSWTASIQVLSED 297
SfCat3                     ETHYVRFNFRTEQGIATLTTAQAAAIQATDPDYFNRDLYNAIDAGNFPAWRLELDVMTPH 156
Dm_CG6871                  EPIYAKFHFKTDQGIKNLDVKTADQLASTDPDYSIRDLYNRIKTCKFPSWTMYIQVMTYE 285
Ag_XP_314995               KPVYCKFHFKTDQGIKNLDPARANELTATDPDYSIRDLYNAIAKKDFPSWTLKVQVMTFE 283
Aa_XP_001663600            KPVYCKFHFKSNQGIKNLEARRADELAGSDPDYSIRDLYNAIAKGECPSWNLKIQVMTFE 283
Am_NP_001171540            EIVYCKFHYKTDQGIKNLPVDKAGELSASNPDYAIQDLYDAIAKNQYPTWTFYIQVMTPT 286
Bm_NP_001036912            VGYWVKFHYKTNQGIKNLSVDKAGELASTDPDYSIRDLYNSIAKGDYPSWTFYIQVMTMA 285
                              : :*.:: : ** .*    *  : .::***  :***  *   . *:*   ::*::

SfCat1                     QAESCKFNPFDMTKIWPHSEYPLIPVGKMVLNRNPKNYFAEVEQIAFSPANMVPGIEPSP 345
SfCat2                     DVKEADFDVFDVTRVLPLDKYPLRPLGRFVLNKNPVNYFAEIEQLAYSPANLVPGILGGP 357
SfCat3                     DIQKLDYDPFDVTRLWKNGTFFTVPVGRLVLNKNVENQFRDVEQGAFNPGHLVPGIPGPV 216
Dm_CG6871                  QAKKFKYNPFDVTKVWSQKEYPLIPVGKMVLDRNPKNYFAEVEQIAFSPAHLVPGVEPSP 345
Ag_XP_314995               QAEKVPYNPFDLTKVWPQNEFPLIPVGRMVLDRNPSNYFAEVEQAAFAPSHLVPGIEPSP 343
Aa_XP_001663600            QAEQHSFNPFDVTKVWPQNEFPLIPVGRMVLDRNPSNYFAEVEQIAFAPSHLVPGIEASP 343
Am_NP_001171540            QAKSFKWNPFDLTKVWPHDEYPLIPVGKLVLNRNPENYFADIEQIAFDPAHMVPGIGASP 346
Bm_NP_001036912            QAESCKFNPFDLTKIWPHAEYPLIPVGKLVLDRNPKNYFAEVEQIAFSPSNLVPGIEPSP 345
                           : :.  :: **:*::     :   *:*::**::*  * * ::** *: *..:***:



SfCat1                     DKMLQGRLFSYSDTHRHRLGANFLQIPVNCPFR-VSVSNYQRDGPQNI-NNQEGCPNYFP 403
SfCat2                     DKVFEARRLAYRDAQYYRLGSNFFNIPVNCPLQ-NRAFPYNRDGVPPVKDNQKDIPNYYP 416
SfCat3                     DFLFRGRRAFYRDTQNYRLGRNHNNILVNMPLY---EKTYVRDGRPPTHFNMKNAPNYYP 273
```

```
Dm_CG6871                 DKMLHGRLFSYSDTHRHRLGPNYLQIPVNCPYK-VKIENFQRDGAMNVTDNQDGAPNYFP 404
Ag_XP_314995              DKMLQARLFAYADTHRHRVGANYLMLPVNCPYR-VATRNFQRDGPMNCTDNQGGAPNYFP 402
Aa_XP_001663600           DKMLQGRLFSYADTHRHRLGANYLQLPVNCPYR-VSMKNYQRDGPMNVTDNQGGAPNYYP 402
Am_NP_001171540           DKMLQGRMFTYGDAHRHRLGPNNLQLPVNCPFKEISVINYQRDGQATI-NNQNGAPNYFP 405
Bm_NP_001036912           DKMLQGRLFAYSDTHRHRLGANYLQIPVNCPYK-VAVSNYQRDGPQAI-HNQDDCPNYFP 403
                          * ::..*   * *:: :*:* *   : ** *        : ***      *    ***:*

SfCat1                    NSFSGPQECPRAQRLQP-RYNVSGDVDRYDSGQTEDNFSQATILYKQV-LDDAQKQRAVD 461
SfCat2                    NSFHGPVPYKEKDRVELIE----------VHQDQPDNFEQARELYINE-MEPEERQRLVE 465
SfCat3                    NSFNGPVPYVDERRPKKKLQVLE---------NNAIDLEPLWYFYNFILEDEAHRQRFID 324
Dm_CG6871                 NSFNGPQECPRARALSS-CCPVTGDVYRYSSGDTEDNFGQVTDFWVHV-LDKCAKKRLVQ 462
Ag_XP_314995              NSFSGPQTCPRAHKLQNTPLKLSGDVNRYETGD-EDNFSQATVFYRRV-LDDAGRQRLIN 460
Aa_XP_001663600           NSFGGPEPCGFAHKLQNSKFNVSGDVNRFESGETEDNFAQPGIFYRRV-LDEAARERMIT 461
Am_NP_001171540           NSFGGPRECPAV---APPTYFVSGDVGRYDVDPKEDNFGQVTLFWRNV-LDDKEKSRLVN 461
Bm_NP_001036912           NSFSGPQECPRAQRLQP-RYNVGGDVDRYDSGQTEDNFSQATALYKQV-FDDAAKQRAIA 461
                          *** **                              ::     ::     :   :.* :

SfCat1                    NIVGNLKDAAGFIQERAIKIFTQVHPDLGSKIAAGLAPFKKYHA---NL----------- 507
SfCat2                    NILYSLGPATKFLQDRAVKMFGRIHSDLSDRIYQGLQANRTKNPYEIDLGFFGT------ 519
SfCat3                    NIVMSLVPVTPPVVQRAIKLLHLVDQDLGNRVRVGYQIALAQAMAEQAAAQATPPPMTPL 384
Dm_CG6871                 NIAGHLSNASQFLQERAVKNFTQVHADFGRMLTEELNLAKSSKF---------------- 506
Ag_XP_314995              NIVGHLKDASPFLQERAVKNFAMVDADFGRHLSEGLKLRRTANL---------------- 514
Aa_XP_001663600           NMVNHMSAASPFIQERAVQNFSQVDADFGRRLTEGLKLRRSAKM---------------- 515
Am_NP_001171540           NLVQNLSNASMFIVERAVKNFTQVDADLGLRLTEGLRNKGVLIN---RYGKTAR------ 512
Bm_NP_001036912           NIVDHLKDAAAFIQERAIKIFSQVHPELGNKVAAGLAPYKKYHA---NL----------- 507
                          *:   :  .:  : :**:: :  :. ::.  :

SfCat1                    ------------------------
SfCat2                    -----------HSYF--------- 523
SfCat3                    RNVPTAEAPPEHDYPKSIYKLSIH 408
Dm_CG6871                 ------------------------
Ag_XP_314995              ------------------------
Aa_XP_001663600           ------------------------
Am_NP_001171540           -----------L------------ 513
Bm_NP_001036912           ------------------------
```

## Figure S32 Amino acid sequences alignment of insect catalases.

Sequence comparison was done using Clustal Omega (1.2.1). Accession numbers for *S. frugiperda* catalases are GSSPFT00030477001 (SfCat1), GSSPFT00000106001 (SfCat2) and GSSPFT00024144001 (SfCat3). Sequences of the selected catalases from other insects were retrieved from GeneBank and from FlyBase for *Drosophila melanogaster*: *D. melanogaster* (Dm) CG6871, *Anopheles gambiae* (Ag) XP_314995, *Aedes aegypti* (XP_001663600), *Apis mellifera* (NP_001171540), *Bombyx mori* (NP_001036912). Fully conserved residues are marked by an asterisk (*), a colon (:) indicates conservation between groups of strongly similar properties and a period (.) indicates conservation between groups of weakly similar properties. Catalytic residues are shown in red (H73 and N147), heme binding residues (S112, V114, F151, F159, M297, M336, R340, Y344) are shown in green and NADPH-binding residues (H192, R201, I276 and E281) are shown in blue.

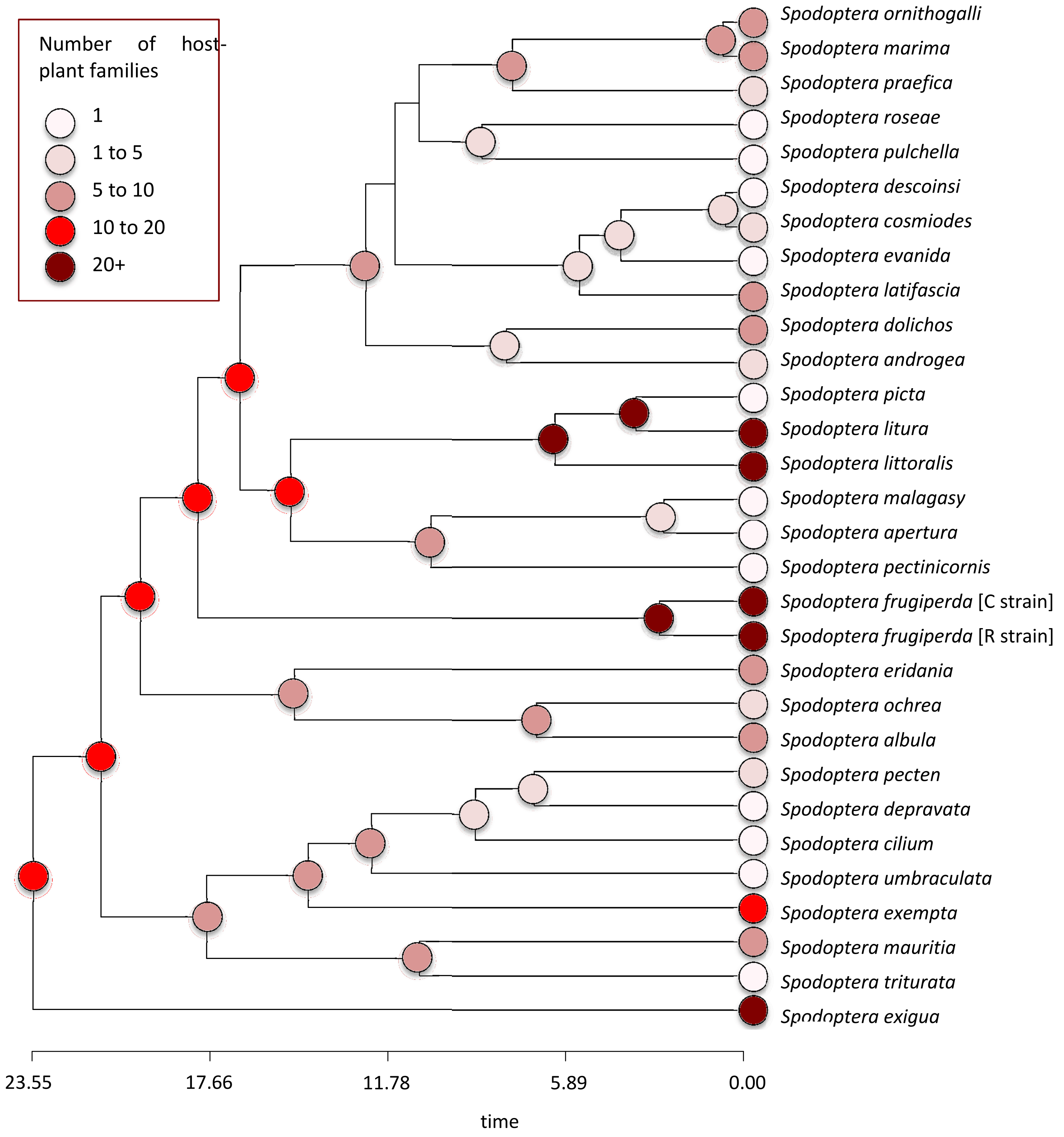


**Figure S33 Evolution of host-plant range in the genus *Spodoptera*.**
Maximum likelihood ancestral state estimation of the number of host-plant families, expressed as a continuous trait. Branch lengths are proportional to time

## SI References


1 Poitout, S. & Bues, R. [Linolenic acid requirements of lepidoptera Noctuidae Quadrifinae Plusiinae: Chrysodeixis chalcites Esp., Autographa gamma L.' Macdunnoughia confusa Stph., Trichoplusia ni Hbn. reared on artificial diets]. *Ann Nutr Aliment* **28**, 173-187 (1974).

2 Nagoshi, R. N. & Meagher, R. Fall armyworm FR sequences map to sex chromosomes and their distribution in the wild indicate limitations in interstrain mating. *Insect Mol Biol* **12**, 453-458 (2003).

3 d'Alencon, E. *et al.* A genomic BAC library and a new BAC-GFP vector to study the holocentric pest Spodoptera frugiperda. *Insect Biochem Mol Biol* **34**, 331-341, doi:10.1016/j.ibmb.2003.12.004 (2004).

4 Gnerre, S. *et al.* High-quality draft assemblies of mammalian genomes from massively parallel sequence data. *Proc Natl Acad Sci U S A* **108**, 1513-1518, doi:1017351108 [pii] 10.1073/pnas.1017351108 (2011).

5 Nguyen, V. H. & Lavenier, D. PLAST: parallel local alignment search tool for database comparison. *BMC Bioinformatics* **10**, 329 (2009).

6 Harris, R. S. *Improved pairwise alignment of genomic DNA.* , The Pennsylvania State University, (2007).

7 Kent, W. J., Baertsch, R., Hinrichs, A., Miller, W. & Haussler, D. Evolution's cauldron: duplication, deletion, and rearrangement in the mouse and human genomes. *Proc Natl Acad Sci U S A* **100**, 11484-11489 (2003).

8 Schmieder, R. & Edwards, R. Quality control and preprocessing of metagenomic datasets. *Bioinformatics* **27**, 863-864, doi:btr026 [pii] 10.1093/bioinformatics/btr026 (2011).

9 Luo, R. *et al.* SOAPdenovo2: an empirically improved memory-efficient short-read de novo assembler. *Gigascience* **1**, 18, doi:2047-217X-1-18 [pii] 10.1186/2047-217X-1-18 (2012).

10 Kajitani, R. *et al.* Efficient de novo assembly of highly heterozygous genomes from whole-genome shotgun short reads. *Genome Res* **24**, 1384-1395, doi:gr.170720.113 [pii] 10.1101/gr.170720.113 (2014).

11 Bankevich, A. *et al.* SPAdes: a new genome assembly algorithm and its applications to single-cell sequencing. *J Comput Biol* **19**, 455-477, doi:10.1089/cmb.2012.0021 (2012).

12 Howe, K. L., Chothia, T. & Durbin, R. GAZE: a generic framework for the integration of gene-prediction data by dynamic programming. *Genome Res* **12**, 1418-1427 (2002).

13 Bairoch, A. *et al.* The Universal Protein Resource (UniProt). *Nucleic Acids Res* **33**, D154-159 (2005).

14 Kent, W. J. BLAT---The BLAST-Like Alignment Tool. *Genome Research* **12**, 656-664, doi:10.1101/gr.229202 (2002).

15 Birney, E., Clamp, M. & Durbin, R. GeneWise and Genomewise. *Genome Res* **14**, 988-995, doi:10.1101/gr.1865504 (2004).

16 Legeai, F. *et al.* Establishment and analysis of a reference transcriptome for Spodoptera frugiperda. *BMC genomics* **15**, 704, doi:10.1186/1471-2164-15-704 (2014).

17 Mott, R. EST_GENOME: a program to align spliced DNA sequences to unspliced genomic DNA. *Comput Appl Biosci* **13**, 477-478 (1997).

18 Korf, I. Gene finding in novel genomes. *BMC Bioinformatics* **5**, 59 (2004).

19 Slater, G. S. & Birney, E. Automated generation of heuristics for biological sequence comparison. *BMC Bioinformatics* **6**, 31 (2005).

20 Keller, O., Kollmar, M., Stanke, M. & Waack, S. A novel hybrid gene prediction method employing protein multiple sequence alignments. *Bioinformatics* **27**, 757-763, doi:btr010 [pii]
10.1093/bioinformatics/btr010 (2011).

21 Bolger, A. M., Lohse, M. & Usadel, B. Trimmomatic: a flexible trimmer for Illumina sequence data. *Bioinformatics* **30**, 2114-2120, doi:btu170 [pii]
10.1093/bioinformatics/btu170 (2014).

22 Grabherr, M. G. *et al.* Full-length transcriptome assembly from RNA-Seq data without a reference genome. *Nat Biotechnol* **29**, 644-652, doi:nbt.1883 [pii]
10.1038/nbt.1883 (2011).

23 Li, B. & Dewey, C. N. RSEM: accurate transcript quantification from RNA-Seq data with or without a reference genome. *BMC Bioinformatics* **12**, 323, doi:1471-2105-12-323 [pii]
10.1186/1471-2105-12-323 (2011).

24 Campbell, M. S., Holt, C., Moore, B. & Yandell, M. Genome Annotation and Curation Using MAKER and MAKER-P. *Curr Protoc Bioinformatics* **48**, 4 11 11-14 11 39, doi:10.1002/0471250953.bi0411s48 (2014).

25 Lomsadze, A., Ter-Hovhannisyan, V., Chernoff, Y. O. & Borodovsky, M. Gene identification in novel eukaryotic genomes by self-training algorithm. *Nucleic Acids Res* **33**, 6494-6506 (2005).

26 Camacho, C. *et al.* BLAST+: architecture and applications. *BMC Bioinformatics* **10**, 421 (2009).

27 Jones, P. *et al.* InterProScan 5: genome-scale protein function classification. *Bioinformatics* **30**, 1236-1240, doi:btu031 [pii]
10.1093/bioinformatics/btu031 (2014).

28 Petersen, T. N., Brunak, S., von Heijne, G. & Nielsen, H. SignalP 4.0: discriminating signal peptides from transmembrane regions. *Nat Methods* **8**, 785-786, doi:10.1038/nmeth.1701 (2011).

29 Krogh, A., Larsson, B., von Heijne, G. & Sonnhammer, E. L. Predicting transmembrane protein topology with a hidden Markov model: application to complete genomes. *J Mol Biol* **305**, 567-580 (2001).

30 Conesa, A. *et al.* Blast2GO: a universal tool for annotation, visualization and analysis in functional genomics research. *Bioinformatics* **21**, 3674-3676 (2005).

31 Lee, E. *et al.* Web Apollo: a web-based genomic annotation editing platform. *Genome Biol* **14**, R93, doi:gb-2013-14-8-r93 [pii]
10.1186/gb-2013-14-8-r93 (2013).

32 Li, H. & Durbin, R. Fast and accurate long-read alignment with Burrows-Wheeler transform. *Bioinformatics* **26**, 589-595, doi:btp698 [pii]
10.1093/bioinformatics/btp698 (2010).

33 Simao, F. A., Waterhouse, R. M., Ioannidis, P., Kriventseva, E. V. & Zdobnov, E. M. BUSCO: assessing genome assembly and annotation completeness with single-copy orthologs. *Bioinformatics* **31**, 3210-3212, doi:btv351 [pii]
10.1093/bioinformatics/btv351 (2015).

34 Flutre, T., Duprat, E., Feuillet, C. & Quesneville, H. Considering transposable element diversification in de novo annotation approaches. *PLoS One* **6**, e16526, doi:10.1371/journal.pone.0016526 (2011).

35 Quesneville, H. *et al.* Combined evidence annotation of transposable elements in genome sequences. *PLoS Comput Biol* **1**, 166-175 (2005).
36 Goecks, J., Nekrutenko, A. & Taylor, J. Galaxy: a comprehensive approach for supporting accessible, reproducible, and transparent computational research in the life sciences. *Genome Biol* **11**, R86, doi:gb-2010-11-8-r86 [pii]
10.1186/gb-2010-11-8-r86 (2010).
37 Blankenberg, D. *et al.* Galaxy: a web-based genome analysis tool for experimentalists. *Curr Protoc Mol Biol* **Chapter 19**, Unit 19 10 11-21, doi:10.1002/0471142727.mb1910s89 (2010).
38 Giardine, B. *et al.* Galaxy: a platform for interactive large-scale genome analysis. *Genome research* **15**, 1451-1455, doi:10.1101/gr.4086505 (2005).
39 Krzywinski, M. *et al.* Circos: an information aesthetic for comparative genomics. *Genome Res* **19**, 1639-1645 (2009).
40 Fischer, S. *et al.* Using OrthoMCL to assign proteins to OrthoMCL-DB groups or to cluster proteomes into new ortholog groups. *Curr Protoc Bioinformatics* **Chapter 6**, Unit 6 12 11-19, doi:10.1002/0471250953.bi0612s35 (2011).
41 Remm, M., Storm, C. E. & Sonnhammer, E. L. Automatic clustering of orthologs and in-paralogs from pairwise species comparisons. *J Mol Biol* **314**, 1041-1052 (2001).
42 Ostlund, G. *et al.* InParanoid 7: new algorithms and tools for eukaryotic orthology analysis. *Nucleic Acids Res* **38**, D196-203, doi:gkp931 [pii]
10.1093/nar/gkp931 (2010).
43 Loytynoja, A. & Goldman, N. Phylogeny-aware gap placement prevents errors in sequence alignment and evolutionary analysis. *Science* **320**, 1632-1635, doi:320/5883/1632 [pii]
10.1126/science.1158395 (2008).
44 Landan, G. & Graur, D. Heads or tails: a simple reliability check for multiple sequence alignments. *Mol Biol Evol* **24**, 1380-1383 (2007).
45 Castresana, J. Selection of conserved blocks from multiple alignments for their use in phylogenetic analysis. *Mol Biol Evol* **17**, 540-552 (2000).
46 Yang, Z., Nielsen, R., Goldman, N. & Pedersen, A. M. Codon-substitution models for heterogeneous selection pressure at amino acid sites. *Genetics* **155**, 431-449 (2000).
47 Yang, Z. PAML 4: phylogenetic analysis by maximum likelihood. *Mol Biol Evol* **24**, 1586-1591 (2007).
48 Langmead, B. & Salzberg, S. L. Fast gapped-read alignment with Bowtie 2. *Nat Methods* **9**, 357-359, doi:nmeth.1923 [pii]
10.1038/nmeth.1923 (2012).
49 Li, H. *et al.* The Sequence Alignment/Map format and SAMtools. *Bioinformatics (Oxford, England)* **25**, 2078-2079, doi:10.1093/bioinformatics/btp352 (2009).
50 Quinlan, A. R. & Hall, I. M. BEDTools: a flexible suite of utilities for comparing genomic features. *Bioinformatics* **26**, 841-842, doi:btq033 [pii]
10.1093/bioinformatics/btq033 (2010).
51 McKenna, A. *et al.* The Genome Analysis Toolkit: a MapReduce framework for analyzing next-generation DNA sequencing data. *Genome Res* **20**, 1297-1303, doi:gr.107524.110 [pii]
10.1101/gr.107524.110 (2010).
52 Danecek, P. *et al.* The variant call format and VCFtools. *Bioinformatics* **27**, 2156-2158, doi:btr330 [pii]

10.1093/bioinformatics/btr330 (2011).
53 Suetsugu, Y. *et al.* Large scale full-length cDNA sequencing reveals a unique genomic landscape in a lepidopteran model insect, Bombyx mori. *G3 (Bethesda, Md.)* **3**, 1481-1492, doi:10.1534/g3.113.006239 (2013).
54 Lemaitre, C., Tannier, E., Gautier, C. & Sagot, M. F. Precise detection of rearrangement breakpoints in mammalian chromosomes. *BMC Bioinformatics* **9**, 286 (2008).
55 Levy, H. C., Garcia-Maruniak, A. & Maruniak, J. E. Strain identification of *Spodoptera frugiperda* (Lepidoptera: Noctuidae) insects and cell line: PCR-RFLP of cytochrome oxidase C subunit I gene. *Florida Entomol.* **85**, 186-190. (2002).
56 Lu, Y. J., Kochert, G. D., Isenhour, D. J. & Adang, M. J. Molecular characterization of a strain-specific repeated DNA sequence in the fall armyworm Spodoptera frugiperda (Lepidoptera: Noctuidae). *Insect Mol Biol* **3**, 123-130. (1994).
57 Jiang, H., Lei, R., Ding, S. W. & Zhu, S. Skewer: a fast and accurate adapter trimmer for next-generation sequencing paired-end reads. *BMC Bioinformatics* **15**, 182, doi:1471-2105-15-182 [pii]
10.1186/1471-2105-15-182 (2014).
58 Sickle: A sliding-window, adaptive, quality-based trimming tool for FastQ files v. Version 1.33 (2011).
59 Plotree, D. & Plotgram, D. PHYLIP-phylogeny inference package *cladistics* **5163-166** (1989).
60 Edgar, R. C. MUSCLE: multiple sequence alignment with high accuracy and high throughput. *Nucleic Acids Res* **32**, 1792-1797 (2004).
61 Tamura, K. *et al.* MEGA5: molecular evolutionary genetics analysis using maximum likelihood, evolutionary distance, and maximum parsimony methods. *Mol Biol Evol* **28**, 2731-2739, doi:msr121 [pii]
10.1093/molbev/msr121 (2011).
62 Weir, B. S. & Cockerham, C. C. Estimating F-Statistics for the analysis of Population structure. *Evolution* **38**, 1358-1370 (1984).
63 Guo, S. & Kim, J. Molecular evolution of Drosophila odorant receptor genes. *Mol Biol Evol* **24**, 1198-1207, doi:10.1093/molbev/msm038 (2007).
64 Gardiner, A., Barker, D., Butlin, R. K., Jordan, W. C. & Ritchie, M. G. Drosophila chemoreceptor gene evolution: selection, specialization and genome size. *Mol Ecol* **17**, 1648-1657, doi:10.1111/j.1365-294X.2008.03713.x (2008).
65 McBride, C. S. Rapid evolution of smell and taste receptor genes during host specialization in Drosophila sechellia. *Proc Natl Acad Sci U S A* **104**, 4996-5001, doi:10.1073/pnas.0608424104 (2007).
66 McBride, C. S., Arguello, J. R. & O'Meara, B. C. Five Drosophila genomes reveal nonneutral evolution and the signature of host specialization in the chemoreceptor superfamily. *Genetics* **177**, 1395-1416, doi:10.1534/genetics.107.078683 (2007).
67 Smadja, C., Shi, P., Butlin, R. K. & Robertson, H. M. Large gene family expansions and adaptive evolution for odorant and gustatory receptors in the pea aphid, Acyrthosiphon pisum. *Mol Biol Evol* **26**, 2073-2086, doi:10.1093/molbev/msp116 (2009).
68 Smadja, C. M. *et al.* Large-scale candidate gene scan reveals the role of chemoreceptor genes in host plant specialization and speciation in the pea aphid. *Evolution; international journal of organic evolution* **66**, 2723-2738, doi:10.1111/j.1558-5646.2012.01612.x (2012).

69 Sanchez-Gracia, A., Vieira, F. G. & Rozas, J. Molecular evolution of the major chemosensory gene families in insects. *Heredity (Edinb)* **103**, 208-216, doi:10.1038/hdy.2009.55 (2009).

70 Legeai, F. *et al.* An Expressed Sequence Tag collection from the male antennae of the Noctuid moth Spodoptera littoralis: a resource for olfactory and pheromone detection research. *BMC Genomics* **12**, 86.

71 Jacquin-Joly, E. *et al.* Candidate chemosensory genes in female antennae of the noctuid moth Spodoptera littoralis. *International journal of biological sciences* **8**, 1036-1050, doi:10.7150/ijbs.4469 (2012).

72 Poivet, E. *et al.* A comparison of the olfactory gene repertoires of adults and larvae in the noctuid moth Spodoptera littoralis. *PloS one* **8**, e60263, doi:10.1371/journal.pone.0060263 (2013).

73 Finn, R. D., Clements, J. & Eddy, S. R. HMMER web server: interactive sequence similarity searching. *Nucleic Acids Res* **39**, W29-37, doi:10.1093/nar/gkr367 (2011).

74 Keller, O., Odronitz, F., Stanke, M., Kollmar, M. & Waack, S. Scipio: using protein sequences to determine the precise exon/intron structures of genes and their orthologs in closely related species. *BMC Bioinformatics* **9**, 278, doi:10.1186/1471-2105-9-278 (2008).

75 Katoh, K. & Standley, D. M. MAFFT multiple sequence alignment software version 7: improvements in performance and usability. *Mol Biol Evol* **30**, 772-780, doi:10.1093/molbev/mst010 (2013).

76 Guindon, S. *et al.* New algorithms and methods to estimate maximum-likelihood phylogenies: assessing the performance of PhyML 3.0. *Syst Biol* **59**, 307-321, doi:10.1093/sysbio/syq010 (2010).

77 Abascal, F., Zardoya, R. & Posada, D. ProtTest: selection of best-fit models of protein evolution. *Bioinformatics* **21**, 2104-2105 (2005).

78 Anisimova, M. & Gascuel, O. Approximate likelihood-ratio test for branches: A fast, accurate, and powerful alternative. *Syst Biol* **55**, 539-552 (2006).

79 Pelosi, P., Zhou, J. J., Ban, L. P. & Calvello, M. Soluble proteins in insect chemical communication. *Cell Mol Life Sci* **63**, 1658-1676, doi:10.1007/s00018-005-5607-0 (2006).

80 Leal, W. S. Odorant reception in insects: roles of receptors, binding proteins, and degrading enzymes. *Annu Rev Entomol* **58**, 373-391, doi:10.1146/annurev-ento-120811-153635 (2013).

81 Leal, W. S., Nikonova, L. & Peng, G. Disulfide structure of the pheromone binding protein from the silkworm moth, Bombyx mori. *FEBS Lett* **464**, 85-90 (1999).

82 Vogt, R. G., Grosse-Wilde, E. & Zhou, J. J. The Lepidoptera Odorant Binding Protein gene family: Gene gain and loss within the GOBP/PBP complex of moths and butterflies. *Insect Biochem Mol Biol* **62**, 142-153, doi:S0965-1748(15)00052-1 [pii]
10.1016/j.ibmb.2015.03.003 (2015).

83 Jacquin-Joly, E., Vogt, R. G., Francois, M. C. & Nagnan-Le Meillour, P. Functional and expression pattern analysis of chemosensory proteins expressed in antennae and pheromonal gland of Mamestra brassicae. *Chemical senses* **26**, 833-844 (2001).

84 Briand, L. *et al.* Characterization of a chemosensory protein (ASP3c) from honeybee (Apis mellifera L.) as a brood pheromone carrier. *Eur J Biochem* **269**, 4586-4596 (2002).

85 Heliconius, T., Consortium, G. & Information, S. Butterfly genome reveals promiscuous exchange of mimicry adaptations among species. *Nature* **487**, 94-98, doi:10.1038/nature11041 (2012).

86 Gong, D. P., Zhang, H. J., Zhao, P., Xia, Q. Y. & Xiang, Z. H. The odorant binding protein gene family from the genome of silkworm, Bombyx mori. *BMC Genomics* **10**, 332, doi:10.1186/1471-2164-10-332 (2009).
87 Vieira, F. G. & Rozas, J. Comparative genomics of the odorant-binding and chemosensory protein gene families across the Arthropoda: origin and evolutionary history of the chemosensory system. *Genome Biol Evol* **3**, 476-490, doi:10.1093/gbe/evr033 (2011).
88 Consortium, T. H. G. Butterfly genome reveals promiscuous exchange of mimicry adaptations among species. *Nature* **487**, 94-98, doi:10.1038/nature11041 (2012).
89 Gong, D. P. *et al.* Identification and expression pattern of the chemosensory protein gene family in the silkworm, Bombyx mori. *Insect Biochem Mol Biol* **37**, 266-277, doi:10.1016/j.ibmb.2006.11.012 (2007).
90 Silbering, A. F. *et al.* Complementary function and integrated wiring of the evolutionarily distinct Drosophila olfactory subsystems. *J Neurosci* **31**, 13357-13375, doi:10.1523/JNEUROSCI.2360-11.2011 (2011).
91 Benton, R., Vannice, K. S., Gomez-Diaz, C. & Vosshall, L. B. Variant ionotropic glutamate receptors as chemosensory receptors in Drosophila. *Cell* **136**, 149-162, doi:10.1016/j.cell.2008.12.001 (2009).
92 Benton, R., Sachse, S., Michnick, S. W. & Vosshall, L. B. Atypical membrane topology and heteromeric function of Drosophila odorant receptors in vivo. *PLoS Biol* **4**, e20, doi:10.1371/journal.pbio.0040020 (2006).
93 Larsson, M. C. *et al.* Or83b encodes a broadly expressed odorant receptor essential for Drosophila olfaction. *Neuron* **43**, 703-714, doi:10.1016/j.neuron.2004.08.019 (2004).
94 Vosshall, L. B. & Hansson, B. S. A unified nomenclature system for the insect olfactory coreceptor. *Chemical senses* **36**, 497-498, doi:10.1093/chemse/bjr022 (2011).
95 Abuin, L. *et al.* Functional architecture of olfactory ionotropic glutamate receptors. *Neuron* **69**, 44-60, doi:10.1016/j.neuron.2010.11.042 (2011).
96 Krieger, J. *et al.* Genes encoding candidate pheromone receptors in a moth (Heliothis virescens). *Proc Natl Acad Sci U S A* **101**, 11845-11850, doi:10.1073/pnas.0403052101 (2004).
97 de Fouchier, A., Montagné, N., Mirabeau, O. & Jacquin-Joly, E. *current views on the function and evolution of olfactory receptors in Lepidoptera*. Vol. 2 385 – 408 (2014).
98 Isono, K. & Morita, H. Molecular and cellular designs of insect taste receptor system. *Frontiers in cellular neuroscience* **4**, 20, doi:10.3389/fncel.2010.00020 (2010).
99 Jones, W. D., Cayirlioglu, P., Kadow, I. G. & Vosshall, L. B. Two chemosensory receptors together mediate carbon dioxide detection in Drosophila. *Nature* **445**, 86-90 (2007).
100 Koenig, C. *et al.* A reference gene set for chemosensory receptor genes of Manduca sexta. *Insect Biochem Mol Biol* **66**, 51-63, doi:S0965-1748(15)30045-X [pii] 10.1016/j.ibmb.2015.09.007 (2015).
101 Montagne, N. *et al.* Functional characterization of a sex pheromone receptor in the pest moth Spodoptera littoralis by heterologous expression in Drosophila. *The European journal of neuroscience* **36**, 2588-2596, doi:10.1111/j.1460-9568.2012.08183.x (2012).
102 Liu, C., Liu, Y., Walker, W. B., Dong, S. & Wang, G. Identification and functional characterization of sex pheromone receptors in beet armyworm Spodoptera exigua (Hubner). *Insect Biochem Mol Biol* **43**, 747-754, doi:S0965-1748(13)00103-3 [pii]

10.1016/j.ibmb.2013.05.009 (2013).
103 Tanaka, K. *et al.* Highly selective tuning of a silkworm olfactory receptor to a key mulberry leaf volatile. *Curr Biol* **19**, 881-890, doi:10.1016/j.cub.2009.04.035 (2009).
104 Zhang, J. *et al.* An odorant receptor from the common cutworm (Spodoptera litura) exclusively tuned to the important plant volatile cis-3-hexenyl acetate. *Insect Mol Biol* **22**, 424-432, doi:10.1111/imb.12033 (2013).
105 Jordan, M. D. *et al.* Odorant receptors from the light brown apple moth (Epiphyas postvittana) recognize important volatile compounds produced by plants. *Chemical senses* **34**, 383-394, doi:10.1093/chemse/bjp010 (2009).
106 Liu, C. *et al.* Narrow tuning of an odorant receptor to plant volatiles in Spodoptera exigua (Hubner). *Insect Mol Biol* **23**, 487-496, doi:10.1111/imb.12096 (2014).
107 Olivier, V., Monsempes, C., Francois, M. C., Poivet, E. & Jacquin-Joly, E. Candidate chemosensory ionotropic receptors in a Lepidoptera. *Insect Mol Biol* **20**, 189-199, doi:10.1111/j.1365-2583.2010.01057.x (2011).
108 Koh, T. W. *et al.* The Drosophila IR20a clade of ionotropic receptors are candidate taste and pheromone receptors. *Neuron* **83**, 850-865, doi:S0896-6273(14)00623-0 [pii]
10.1016/j.neuron.2014.07.012 (2014).
109 Croset, V. *et al.* Ancient protostome origin of chemosensory ionotropic glutamate receptors and the evolution of insect taste and olfaction. *PLoS Genet* **6**, e1001064, doi:10.1371/journal.pgen.1001064 (2010).
110 Robertson, H. M. & Kent, L. B. Evolution of the gene lineage encoding the carbon dioxide receptor in insects. *J Insect Sci* **9**, 19 (2009).
111 Kent, L. B. & Robertson, H. M. Evolution of the sugar receptors in insects. *BMC Evol Biol* **9**, 41 (2009).
112 Wanner, K. W. & Robertson, H. M. The gustatory receptor family in the silkworm moth *Bombyx mori* is characterized by a large expansion of a single lineage of putative bitter receptors. *Insect Mol Biol* **17**, 621-629, doi:10.1111/j.1365-2583.2008.00836.x (2008).
113 Briscoe, A. D. *et al.* Female behaviour drives expression and evolution of gustatory receptors in butterflies. *PLoS Genet* **9**, e1003620, doi:10.1371/journal.pgen.1003620 (2013).
114 Xu, W., Papanicolaou, A., Zhang, H. J. & Anderson, A. Expansion of a bitter taste receptor family in a polyphagous insect herbivore. *Sci Rep* **6**, 23666, doi:srep23666 [pii]
10.1038/srep23666 (2016).
115 Engsontia, P., Sangket, U., Chotigeat, W. & Satasook, C. Molecular evolution of the odorant and gustatory receptor genes in lepidopteran insects: implications for their adaptation and speciation. *J Mol Evol* **79**, 21-39, doi:10.1007/s00239-014-9633-0 (2014).
116 Werck-Reichhart, D. & Feyereisen, R. Cytochromes P450: a success story. *Genome Biol* **1**, REVIEWS3003, doi:10.1186/gb-2000-1-6-reviews3003 (2000).
117 Heidel-Fischer, H. M. & Vogel, H. Molecular mechanisms of insect adaptation to plant secondary compounds. *Current Opinion in Insect Science* 8-14 (2015).
118 Ai, J. *et al.* Genome-wide analysis of cytochrome P450 monooxygenase genes in the silkworm, Bombyx mori. *Gene* **480**, 42-50, doi:10.1016/j.gene.2011.03.002 (2011).
119 Giraudo, M. *et al.* Cytochrome P450s from the fall armyworm (*Spodoptera frugiperda*): responses to plant allelochemicals and pesticides. *Insect Mol Biol* **12140**, doi:10.1111/imb.12140 (2014).

120 Nelson, D. R. Cytochrome P450 nomenclature. *Methods Mol Biol* **107**, 15-24, doi:10.1385/0-89603-519-0:15 (1998).
121 Ronquist, F. *et al.* MrBayes 3.2: efficient Bayesian phylogenetic inference and model choice across a large model space. *Syst Biol* **61**, 539-542, doi:10.1093/sysbio/sys029 (2012).
122 Darriba, D., Taboada, G. L., Doallo, R. & Posada, D. ProtTest 3: fast selection of best-fit models of protein evolution. *Bioinformatics* **27**, 1164-1165, doi:10.1093/bioinformatics/btr088 (2011).
123 Tamura, K., Stecher, G., Peterson, D., Filipski, A. & Kumar, S. MEGA6: Molecular Evolutionary Genetics Analysis version 6.0. *Mol Biol Evol* **30**, 2725-2729, doi:10.1093/molbev/mst197 (2013).
124 Yu, L. *et al.* Characterization and expression of the cytochrome P450 gene family in diamondback moth, *Plutella xylostella* (L.). *Sci Rep* **5**, 8952, doi:srep08952 [pii]
10.1038/srep08952 (2015).
125 Chauhan, R., Jones, R., Wilkinson, P., Pauchet, Y. & Ffrench-Constant, R. H. Cytochrome P450-encoding genes from the *Heliconius* genome as candidates for cyanogenesis. *Insect Mol Biol* **22**, 532-540, doi:10.1111/imb.12042 (2013).
126 Hung, C. F., Berenbaum, M. R. & Schuler, M. A. Isolation and characterization of CYP6B4, a furanocoumarin-inducible cytochrome P450 from a polyphagous caterpillar (Lepidoptera:papilionidae). *Insect Biochem Mol Biol* **27**, 377-385 (1997).
127 Li, X., Baudry, J., Berenbaum, M. R. & Schuler, M. A. Structural and functional divergence of insect CYP6B proteins: From specialist to generalist cytochrome P450. *Proc Natl Acad Sci U S A* **101**, 2939-2944 (2004).
128 Kumar, P., Pandit, S. S., Steppuhn, A. & Baldwin, I. T. Natural history-driven, plant-mediated RNAi-based study reveals CYP6B46's role in a nicotine-mediated antipredator herbivore defense. *Proc Natl Acad Sci U S A* **111**, 1245-1252, doi:1314848111 [pii]
10.1073/pnas.1314848111 (2014).
129 Tao, X. Y., Xue, X. Y., Huang, Y. P., Chen, X. Y. & Mao, Y. B. Gossypol-enhanced P450 gene pool contributes to cotton bollworm tolerance to a pyrethroid insecticide. *Mol Ecol* **21**, 4371-4385, doi:10.1111/j.1365-294X.2012.05548.x (2012).
130 Wang, Y. H. *et al.* Changes in the activity and the expression of detoxification enzymes in silkworms (*Bombyx mori*) after phoxim feeding. *Pestic Biochem Physiol* **105**, 13-17, doi:S0048-3575(12)00164-2 [pii]
10.1016/j.pestbp.2012.11.001 (2013).
131 Wang, R. L., Staehelin, C., Xia, Q. Q., Su, Y. J. & Zeng, R. S. Identification and Characterization of CYP9A40 from the Tobacco Cutworm Moth (Spodoptera litura), a Cytochrome P450 Gene Induced by Plant Allelochemicals and Insecticides. *International journal of molecular sciences* **16**, 22606-22620, doi:ijms160922606 [pii]
10.3390/ijms160922606 (2015).
132 Maibeche-Coisne, M., Jacquin-Joly, E., Francois, M. C. & Nagnan-Le Meillour, P. cDNA cloning of biotransformation enzymes belonging to the cytochrome P450 family in the antennae of the noctuid moth Mamestra brassicae. *Insect Mol Biol* **11**, 273-281, doi:335 [pii] (2002).
133 Qiu, Y. *et al.* An insect-specific P450 oxidative decarbonylase for cuticular hydrocarbon biosynthesis. *Proc Natl Acad Sci U S A* **109**, 14858-14863, doi:1208650109 [pii]

10.1073/pnas.1208650109 (2012).
134 Ono, H., Ozaki, K. & Yoshikawa, H. Identification of cytochrome P450 and glutathione-S-transferase genes preferentially expressed in chemosensory organs of the swallowtail butterfly, Papilio xuthus L. *Insect biochemistry and molecular biology* **35**, 837-846, doi:10.1016/j.ibmb.2005.03.013 (2005).
135 Rong, Y. *et al.* CYP341B14: a cytochrome P450 involved in the specific epoxidation of pheromone precursors in the fall webworm Hyphantria cunea. *Insect Biochem Mol Biol* **54**, 122-128, doi:S0965-1748(14)00159-3 [pii]
10.1016/j.ibmb.2014.09.009 (2014).
136 Legeai, F. *et al.* An Expressed Sequence Tag collection from the male antennae of the Noctuid moth Spodoptera littoralis: a resource for olfactory and pheromone detection research. *BMC Genomics* **12**, 86, doi:10.1186/1471-2164-12-86 (2011).
137 Jacquin-Joly, E. *et al.* Candidate chemosensory Genes In Female Antennae Of The Noctuid Moth *Spodoptera littoralis*. *Int J Biol Sci* **8**, 1036 (2012).
138 Poivet, E. *et al.* A comparison of the olfactory gene repertoires of adults and larvae in the noctuid moth Spodoptera littoralis. *PLoS One* **8**, e60263, doi:10.1371/journal.pone.0060263 (2013).
139 Giardine, B. *et al.* Galaxy: a platform for interactive large-scale genome analysis. *Genome Res* **15**, 1451-1455, doi:10.1101/gr.4086505 (2005).
140 Oakeshott, J. G., Claudianos, C., Campbell, P. M., Newcomb, R. D. & Russell, R. J. *Biochemical genetics and genomics of insect esterases*. Vol. 5 309-381 (Elsevier, 2005).
141 Teese, M. G. *et al.* Gene identification and proteomic analysis of the esterases of the cotton bollworm, *Helicoverpa armigera*. *Insect Biochem Mol Biol* **40**, 1-16, doi:10.1016/j.ibmb.2009.12.002 (2010).
142 Durand, N., Chertemps, T. & Maibeche-Coisne, M. Antennal carboxylesterases in a moth, structural and functional diversity. *Commun Integr Biol* **5**, 284-286, doi:10.4161/cib.19701
2012CIB0015 [pii] (2012).
143 Sievers, F. *et al.* Fast, scalable generation of high-quality protein multiple sequence alignments using Clustal Omega. *Mol Syst Biol* **7**, 539, doi:msb201175 [pii]
10.1038/msb.2011.75 (2011).
144 Durand, N. *et al.* A diversity of putative carboxylesterases are expressed in the antennae of the noctuid moth Spodoptera littoralis. *Insect Mol Biol* **19**, 87-97, doi:IMB939 [pii]
10.1111/j.1365-2583.2009.00939.x (2010).
145 Yu, Q. Y., Lu, C., Li, W. L., Xiang, Z. H. & Zhang, Z. Annotation and expression of carboxylesterases in the silkworm, Bombyx mori. *BMC Genomics* **10**, 553, doi:1471-2164-10-553 [pii]
10.1186/1471-2164-10-553 (2009).
146 Durand, N. *et al.* Degradation of pheromone and plant volatile components by a same odorant-degrading enzyme in the cotton leafworm, Spodoptera littoralis. *PLoS One* **6**, e29147, doi:10.1371/journal.pone.0029147
PONE-D-11-22004 [pii] (2011).
147 Bock, K. W. Vertebrate UDP-glucuronosyltransferases: functional and evolutionary aspects. *Biochemical Pharmacology* **66**, 691-696, doi:http://dx.doi.org/10.1016/S0006-2952(03)00296-X (2003).

148 Morello, A. & Repetto, Y. UDP-glucosyltransferase activity of housefly microsomal fraction. *Biochemical Journal* **177**, 809-812 (1979).

149 Real, M. D., Ferré, J. & Chapa, F. J. UDP-glucosyltransferase activity toward exogenous substrates in *Drosophila melanogaster*. *Analytical Biochemistry* **194**, 349-352, doi:Doi: 10.1016/0003-2697(91)90239-p (1991).

150 Ahmad, S. A. & Hopkins, T. L. Phenol β-glucosyltransferase and β-glucosidase activities in the tobacco hornworm larva *Manduca sexta* (L.): Properties and tissue localization. *Archives of Insect Biochemistry and Physiology* **21**, 207-224, doi:10.1002/arch.940210305 (1992).

151 Luque, T., Okano, K. & O'Reilly, D. R. Characterization of a novel silkworm (*Bombyx mori*) phenol UDP-glucosyltransferase. *European Journal of Biochemistry* **269**, 819-825 (2002).

152 Ahmad, S. A. & Hopkins, T. L. β-Glucosylation of plant phenolics by phenol β-glucosyltransferase in larval tissues of the tobacco hornworm, *Manduca sexta* (L.). *Insect Biochemistry and Molecular Biology* **23**, 581-589, doi:Doi: 10.1016/0965-1748(93)90031-m (1993).

153 Ahn, S.-J. *et al.* Metabolic detoxification of capsaicin by UDP-glycosyltransferase in three Helicoverpa species. *Archives of Insect Biochemistry and Physiology* **78**, 104-118, doi:10.1002/arch.20444 (2011).

154 Daimon, T. *et al.* The silkworm Green b locus encodes a quercetin 5-O-glucosyltransferase that produces green cocoons with UV-shielding properties. *Proceedings of the National Academy of Sciences of the United States of America* **107**, 11471-11476, doi:10.1073/pnas.1000479107 (2010).

155 Kojima, W., Fujii, T., Suwa, M., Miyazawa, M. & Ishikawa, Y. Physiological adaptation of the Asian corn borer Ostrinia furnacalis to chemical defenses of its host plant, maize. *Journal of Insect Physiology* **56**, 1349-1355, doi:http://dx.doi.org/10.1016/j.jinsphys.2010.04.021 (2010).

156 Lee, S.-W., Ohta, K., Tashiro, S. & Shono, T. Metabolic resistance mechanisms of the housefly (Musca domestica) resistant to pyraclofos. *Pesticide Biochemistry and Physiology* **85**, 76-83, doi:http://dx.doi.org/10.1016/j.pestbp.2005.10.005 (2006).

157 Sasai, H. *et al.* Species-specific glucosylation of DIMBOA in larvae of the rice armyworm. *Biosci. Biotechnol. Biochem.* **73**, 1333-1338, doi:10.1271/bbb.80903 (2009).

158 Wang, Q., Hasan, G. & Pikielny, C. W. Preferential expression of biotransformation enzymes in the olfactory organs of *Drosophila melanogaster*, the antennae. *Journal of Biological Chemistry* **274**, 10309-10315, doi:10.1074/jbc.274.15.10309 (1999).

159 Younus, F. *et al.* Identification of candidate odorant degrading gene/enzyme systems in the antennal transcriptome of Drosophila melanogaster. *Insect Biochemistry and Molecular Biology* **53**, 30-43, doi:http://dx.doi.org/10.1016/j.ibmb.2014.07.003 (2014).

160 Bozzolan, F. *et al.* Antennal uridine diphosphate (UDP)-glycosyltransferases in a pest insect: diversity and putative function in odorant and xenobiotics clearance. *Insect Molecular Biology* **23**, 539-549, doi:10.1111/imb.12100 (2014).

161 Svoboda, J. & Weirich, G. Sterol metabolism in the tobacco hornworm, *Manduca sexta* - A review. *Lipids* **30**, 263-267, doi:10.1007/bf02537831 (1995).

162 Ahmad, S. A., Hopkins, T. L. & Kramer, K. J. Tyrosine β-glucosyltransferase in the tobacco hornworm, *Manduca sexta* (L): Properties, tissue localization, and developmental profile. *Insect Biochemistry and Molecular Biology* **26**, 49-57 (1996).

163 Hopkins, T. L. & Kramer, K. J. Insect cuticle sclerotization. *Annual Review of Entomology* **37**, 273-302 (1992).
164 Wiesen, B., Krug, E., Fiedler, K., Wray, V. & Proksch, P. Sequestration of host-plant-derived flavonoids by lycaenid butterfly *Polyommatus icarus*. *Journal of Chemical Ecology* **20**, 2523-2538, doi:10.1007/bf02036189 (1994).
165 Jensen, N. B. *et al.* Convergent evolution in biosynthesis of cyanogenic defence compounds in plants and insects. *Nat Commun* **2**, 273, doi:http://www.nature.com/ncomms/journal/v2/n4/suppinfo/ncomms1271_S1.html (2011).
166 Maag, D. *et al.* 3-β-d-Glucopyranosyl-6-methoxy-2-benzoxazolinone (MBOA-N-Glc) is an insect detoxification product of maize 1,4-benzoxazin-3-ones. *Phytochemistry* **102**, 97-105, doi:http://dx.doi.org/10.1016/j.phytochem.2014.03.018 (2014).
167 Wouters, F. C. *et al.* Reglucosylation of the Benzoxazinoid DIMBOA with Inversion of Stereochemical Configuration is a Detoxification Strategy in Lepidopteran Herbivores. *Angewandte Chemie International Edition* **53**, 11320-11324, doi:10.1002/anie.201406643 (2014).
168 Ahn, S. J., Vogel, H. & Heckel, D. G. Comparative analysis of the UDP-glycosyltransferase multigene family in insects. *Insect Biochem Mol Biol* **42**, 133-147, doi:S0965-1748(11)00202-5 [pii]
10.1016/j.ibmb.2011.11.006 (2012).
169 Linton, K. J. Structure and function of ABC transporters. *Physiology (Bethesda)* **22**, 122-130, doi:10.1152/physiol.00046.2006 (2007).
170 Labbe, R., Caveney, S. & Donly, C. Genetic analysis of the xenobiotic resistance-associated ABC gene subfamilies of the Lepidoptera. *Insect Mol Biol* **20**, 243-256, doi:10.1111/j.1365-2583.2010.01064.x (2011).
171 Liu, S. *et al.* Genome-wide identification and characterization of ATP-binding cassette transporters in the silkworm, Bombyx mori. *BMC Genomics* **12**, 491, doi:10.1186/1471-2164-12-491 (2011).
172 Xie, X. *et al.* Genome-wide analysis of the ATP-binding cassette (ABC) transporter gene family in the silkworm, Bombyx mori. *Mol Biol Rep* **39**, 7281-7291, doi:10.1007/s11033-012-1558-3 (2012).
173 Yousef, G. M., Kopolovic, A. D., Elliott, M. B. & Diamandis, E. P. Genomic overview of serine proteases. *Biochem Bioph Res Co* **305**, 28-36, doi:Doi 10.1016/S0006-291x(03)00638-7 (2003).
174 Cera, E. D. Serine proteases. *IUBMB Life* **61**, 510-515, doi:10.1002/iub.186. (2009).
175 Srinivasan, A., Giri, A. P. & Gupta, V. S. Structural and functional diversities in Lepidopteran serine proteases. *Cellular & Molecular Biology Letters* **11**, 132-154, doi:10.2478/s11658-006-0012-8 (2006).
176 Dunse, K. M. *et al.* Molecular basis for the resistance of an insect chymotrypsin to a potato type II proteinase inhibitor. *Proceedings of the National Academy of Sciences of the United States of America* **107**, 15016-15021, doi:Doi 10.1073/Pnas.1009327107 (2010).
177 Dunse, K. M. *et al.* Coexpression of potato type I and II proteinase inhibitors gives cotton plants protection against insect damage in the field. *Proc Natl Acad Sci U S A* **107**, 15011-15015, doi:1009241107 [pii]
10.1073/pnas.1009241107 (2010).
178 Gatehouse, L. N., Shannon, A. L., Burgess, E. P. & Christeller, J. T. Characterization of major midgut proteinase cDNAs from Helicoverpa armigera larvae and changes in

gene expression in response to four proteinase inhibitors in the diet. *Insect Biochem Mol Biol* **27**, 929-944, doi:S096517489700074X [pii] (1997).
179 Hegedus, D. *et al.* Midgut proteases from Mamestra configurata (Lepidoptera: Noctuidae) larvae: characterization, cDNA cloning, and expressed sequence tag analysis. *Arch Insect Biochem Physiol* **53**, 30-47, doi:10.1002/arch.10084 (2003).
180 Mazumdar-Leighton, S., Babu, C. R. & Bennett, J. Identification of novel serine proteinase gene transcripts in the midguts of two tropical insect pests, Scirpophaga incertulas (Wk.) and Helicoverpa armigera (Hb.). *Insect Biochem Mol Biol* **30**, 57-68, doi:S0965174899000971 [pii] (2000).
181 Ross, J., Jiang, H., Kanost, M. R. & Wang, Y. Serine proteases and their homologs in the Drosophila melanogaster genome: an initial analysis of sequence conservation and phylogenetic relationships. *Gene* **304**, 117-131, doi:Doi 10.1016/S0378-1119(02)01187-3 (2003).
182 Zhao, P. *et al.* Genome-wide identification and expression analysis of serine proteases and homologs in the silkworm *Bombyx mori*. *Bmc Genomics* **11**, doi:Artn 405
Doi 10.1186/1471-2164-11-405 (2010).
183 Altschul, S. F. *et al.* Gapped BLAST and PSI-BLAST: a new generation of protein database search programs. *Nucleic Acids Res* **25**, 3389-3402 (1997).
184 Tanaka, H. *et al.* A genome-wide analysis of genes and gene families involved in innate immunity of Bombyx mori. *Insect biochemistry and molecular biology* **38**, 1087-1110, doi:10.1016/j.ibmb.2008.09.001 (2008).
185 Cao, X. *et al.* The immune signaling pathways of Manduca sexta. *Insect Biochem Mol Biol* **62**, 64-74, doi:S0965-1748(15)00063-6 [pii]
10.1016/j.ibmb.2015.03.006 (2015).
186 He, Y. *et al.* A genome-wide analysis of antimicrobial effector genes and their transcription patterns in Manduca sexta. *Insect Biochem Mol Biol* **62**, 23-37, doi:S0965-1748(15)00022-3 [pii]
10.1016/j.ibmb.2015.01.015 (2015).
187 Vogel, H., Altincicek, B., Glockner, G. & Vilcinskas, A. A comprehensive transcriptome and immune-gene repertoire of the lepidopteran model host Galleria mellonella. *BMC Genomics* **12**, 308, doi:1471-2164-12-308 [pii]
10.1186/1471-2164-12-308 (2011).
188 Evans, J. D. *et al.* Immune pathways and defence mechanisms in honey bees Apis mellifera. *Insect Mol Biol* **15**, 645-656 (2006).
189 Ferrandon, D., Imler, J. L., Hetru, C. & Hoffmann, J. A. The Drosophila systemic immune response: sensing and signalling during bacterial and fungal infections. *Nat Rev Immunol* **7**, 862-874 (2007).
190 Lemaitre, B. & Hoffmann, J. The host defense of Drosophila melanogaster. *Annu Rev Immunol* **25**, 697-743 (2007).
191 Parker, J. S., Roe, S. M. & Barford, D. Structural insights into mRNA recognition from a PIWI domain-siRNA guide complex. *Nature* **434**, 663-666, doi:10.1038/nature03462 (2005).
192 Bettencourt, R., Terenius, O. & Faye, I. Hemolin gene silencing by ds-RNA injected into Cecropia pupae is lethal to next generation embryos. *Insect molecular biology* **11**, 267-271 (2002).
193 Wilkins, C. *et al.* RNA interference is an antiviral defence mechanism in Caenorhabditis elegans. *Nature* **436**, 1044-1047, doi:10.1038/nature03957 (2005).

194 Terenius, O. *et al.* RNA interference in Lepidoptera: an overview of successful and unsuccessful studies and implications for experimental design. *J Insect Physiol* **57**, 231-245.

195 Matzke, M. A. & Birchler, J. A. RNAi-mediated pathways in the nucleus. *Nat Rev Genet* **6**, 24-35 (2005).

196 Verdel, A. *et al.* RNAi-mediated targeting of heterochromatin by the RITS complex. *Science* **303**, 672-676, doi:10.1126/science.1093686 (2004).

197 Okamura, K., Ishizuka, A., Siomi, H. & Siomi, M. C. Distinct roles for Argonaute proteins in small RNA-directed RNA cleavage pathways. *Genes Dev* **18**, 1655-1666, doi:10.1101/gad.1210204 (2004).

198 Burglin, T. R. & Affolter, M. Homeodomain proteins: an update. *Chromosoma*, doi:10.1007/s00412-015-0543-8
10.1007/s00412-015-0543-8 [pii] (2015).

199 Zhong, Y. F., Butts, T. & Holland, P. W. HomeoDB: a database of homeobox gene diversity. *Evol Dev* **10**, 516-518 (2008).

200 Zhong, Y. F. & Holland, P. W. HomeoDB2: functional expansion of a comparative homeobox gene database for evolutionary developmental biology. *Evol Dev* **13**, 567-568, doi:10.1111/j.1525-142X.2011.00513.x (2011).

201 Li, W. *et al.* The EMBL-EBI bioinformatics web and programmatic tools framework. *Nucleic Acids Res* **43**, W580-584, doi:10.1093/nar/gkv279 (2015).

202 Mesika, A., Ben-Dor, S., Laviad, E. L. & Futerman, A. H. A new functional motif in Hox domain-containing ceramide synthases: identification of a novel region flanking the Hox and TLC domains essential for activity. *J Biol Chem* **282**, 27366-27373 (2007).

203 Ferguson, L. *et al.* Ancient expansion of the hox cluster in lepidoptera generated four homeobox genes implicated in extra-embryonic tissue formation. *PLoS Genet* **10**, e1004698, doi:10.1371/journal.pgen.1004698
PGENETICS-D-14-01212 [pii] (2014).

204 Chai, C. L. *et al.* A genomewide survey of homeobox genes and identification of novel structure of the Hox cluster in the silkworm, *Bombyx mori*. *Insect Biochem Mol Biol* **38**, 1111-1120 (2008).

205 Melters, D. P., Paliulis, L. V., Korf, I. F. & Chan, S. W. Holocentric chromosomes: convergent evolution, meiotic adaptations, and genomic analysis. *Chromosome Res* **20**, 579-593, doi:10.1007/s10577-012-9292-1 (2012).

206 Perpelescu, M. & Fukagawa, T. The ABCs of CENPs. *Chromosoma* **120**, 425-446, doi:10.1007/s00412-011-0330-0 (2011).

207 d'Alencon, E. *et al.* Characterization of a CENP-B homolog in the holocentric Lepidoptera Spodoptera frugiperda. *Gene* **485**, 91-101, doi:S0378-1119(11)00265-4 [pii] 10.1016/j.gene.2011.06.007 (2011).

208 Fukagawa, T. Formation of a centromere-specific chromatin structure. *Epigenetics* **7**, 672-675 (2012).

209 Zeitlin, S. G. *et al.* Double-strand DNA breaks recruit the centromeric histone CENP-A. *Proceedings of the National Academy of Sciences of the United States of America* **106**, 15762-15767, doi:10.1073/pnas.0908233106 (2009).

210 Dunlap, J. C. Molecular bases for circadian clocks. *Cell* **96**, 271-290 (1999).

211 Wijnen, H. & Young, M. W. Interplay of circadian clocks and metabolic rhythms. *Annu Rev Genet* **40**, 409-448, doi:10.1146/annurev.genet.40.110405.090603 (2006).

212 Sandrelli, F., Costa, R., Kyriacou, C. P. & Rosato, E. Comparative analysis of circadian clock genes in insects. *Insect Mol Biol* **17**, 447-463, doi:10.1111/j.1365-2583.2008.00832.x (2008).
213 Zhan, S., Merlin, C., Boore, J. L. & Reppert, S. M. The monarch butterfly genome yields insights into long-distance migration. *Cell* **147**, 1171-1185, doi:S0092-8674(11)01268-2 [pii]
10.1016/j.cell.2011.09.052 (2011).
214 Hardin, P. E. The circadian timekeeping system of Drosophila. *Curr Biol* **15**, R714-722, doi:10.1016/j.cub.2005.08.019 (2005).
215 Li, W. W., Li, J. & Bao, J. K. Microautophagy: lesser-known self-eating. *Cell Mol Life Sci* **69**, 1125-1136, doi:10.1007/s00018-011-0865-5 (2012).
216 Kaushik, S. & Cuervo, A. M. Chaperones in autophagy. *Pharmacological research : the official journal of the Italian Pharmacological Society* **66**, 484-493, doi:10.1016/j.phrs.2012.10.002 (2012).
217 Mizushima, N., Yoshimori, T. & Levine, B. Methods in mammalian autophagy research. *Cell* **140**, 313-326, doi:10.1016/j.cell.2010.01.028 (2010).
218 Facey, C. O. & Lockshin, R. A. The execution phase of autophagy associated PCD during insect metamorphosis. *Apoptosis : an international journal on programmed cell death* **15**, 639-652, doi:10.1007/s10495-010-0499-3 (2010).
219 Mizushima, N. & Levine, B. Autophagy in mammalian development and differentiation. *Nat Cell Biol* **12**, 823-830, doi:10.1038/ncb0910-823 (2010).
220 Tracy, K. & Baehrecke, E. H. The role of autophagy in Drosophila metamorphosis. *Current topics in developmental biology* **103**, 101-125, doi:10.1016/B978-0-12-385979-2.00004-6 (2013).
221 Romanelli, D., Casati, B., Franzetti, E. & Tettamanti, G. A molecular view of autophagy in Lepidoptera. *BioMed research international* **2014**, 902315, doi:10.1155/2014/902315 (2014).
222 Reggiori, F. & Klionsky, D. J. Autophagic processes in yeast: mechanism, machinery and regulation. *Genetics* **194**, 341-361, doi:10.1534/genetics.112.149013 (2013).
223 Mizushima, N. & Komatsu, M. Autophagy: renovation of cells and tissues. *Cell* **147**, 728-741, doi:10.1016/j.cell.2011.10.026 (2011).
224 Lee, J. Y., Chiu, Y. H., Asara, J. & Cantley, L. C. Inhibition of PI3K binding to activators by serine phosphorylation of PI3K regulatory subunit p85alpha Src homology-2 domains. *Proc Natl Acad Sci U S A* **108**, 14157-14162, doi:10.1073/pnas.1107747108 (2011).
225 Hosokawa, N. *et al.* Atg101, a novel mammalian autophagy protein interacting with Atg13. *Autophagy* **5**, 973-979 (2009).
226 Hara, T. *et al.* FIP200, a ULK-interacting protein, is required for autophagosome formation in mammalian cells. *J Cell Biol* **181**, 497-510, doi:10.1083/jcb.200712064 (2008).
227 Simonsen, A. & Tooze, S. A. Coordination of membrane events during autophagy by multiple class III PI3-kinase complexes. *J Cell Biol* **186**, 773-782, doi:10.1083/jcb.200907014 (2009).
228 Shpilka, T., Weidberg, H., Pietrokovski, S. & Elazar, Z. Atg8: an autophagy-related ubiquitin-like protein family. *Genome Biol* **12**, 226, doi:10.1186/gb-2011-12-7-226 (2011).
229 Gai, Z. *et al.* Characterization of Atg8 in lepidopteran insect cells. *Arch Insect Biochem Physiol* **84**, 57-77, doi:10.1002/arch.21114 (2013).

230 Hu, C., Zhang, X., Teng, Y. B., Hu, H. X. & Li, W. F. Structure of autophagy-related protein Atg8 from the silkworm Bombyx mori. *Acta crystallographica. Section F, Structural biology and crystallization communications* **66**, 787-790, doi:10.1107/S1744309110018464 (2010).
231 Pattingre, S., Espert, L., Biard-Piechaczyk, M. & Codogno, P. Regulation of macroautophagy by mTOR and Beclin 1 complexes. *Biochimie* **90**, 313-323, doi:10.1016/j.biochi.2007.08.014 (2008).
232 Vanhaesebroeck, B., Guillermet-Guibert, J., Graupera, M. & Bilanges, B. The emerging mechanisms of isoform-specific PI3K signalling. *Nat Rev Mol Cell Biol* **11**, 329-341, doi:10.1038/nrm2882 (2010).
233 Hanada, M., Feng, J. & Hemmings, B. A. Structure, regulation and function of PKB/AKT--a major therapeutic target. *Biochim Biophys Acta* **1697**, 3-16, doi:10.1016/j.bbapap.2003.11.009 (2004).
234 Zhou, S. *et al.* Two Tor genes in the silkworm Bombyx mori. *Insect Mol Biol* **19**, 727-735, doi:10.1111/j.1365-2583.2010.01026.x (2010).
235 Salasc, F. *et al.* Role of the phosphatidylinositol-3-kinase/Akt/target of rapamycin pathway during ambidensovirus infection of insect cells. *J Gen Virol* **97**, 233-245, doi:10.1099/jgv.0.000327 (2016).
236 Taylor, R. C., Cullen, S. P. & Martin, S. J. Apoptosis: controlled demolition at the cellular level. *Nat Rev Mol Cell Biol* **9**, 231-241, doi:10.1038/nrm2312 (2008).
237 Zhang, J. Y. *et al.* The genomic underpinnings of apoptosis in the silkworm, Bombyx mori. *BMC Genomics* **11**, 611, doi:10.1186/1471-2164-11-611 (2010).
238 Huang, N., Civciristov, S., Hawkins, C. J. & Clem, R. J. SfDronc, an initiator caspase involved in apoptosis in the fall armyworm Spodoptera frugiperda. *Insect Biochem Mol Biol* **43**, 444-454, doi:10.1016/j.ibmb.2013.02.005 (2013).
239 Ahmad, M. *et al.* Spodoptera frugiperda caspase-1, a novel insect death protease that cleaves the nuclear immunophilin FKBP46, is the target of the baculovirus antiapoptotic protein p35. *J Biol Chem* **272**, 1421-1424 (1997).
240 Huang, Q. *et al.* Evolutionary conservation of apoptosis mechanisms: lepidopteran and baculoviral inhibitor of apoptosis proteins are inhibitors of mammalian caspase-9. *Proc Natl Acad Sci U S A* **97**, 1427-1432 (2000).
241 Kampinga, H. H. *et al.* Guidelines for the nomenclature of the human heat shock proteins. *Cell stress & chaperones* **14**, 105-111, doi:10.1007/s12192-008-0068-7 (2009).
242 Mayer, M. P. & Bukau, B. Hsp70 chaperones: cellular functions and molecular mechanism. *Cell Mol Life Sci* **62**, 670-684, doi:10.1007/s00018-004-4464-6 (2005).
243 Manjunatha, H. B., Rajesh, R. K. & Aparna, H. S. Silkworm thermal biology: a review of heat shock response, heat shock proteins and heat acclimation in the domesticated silkworm, Bombyx mori. *J Insect Sci* **10**, 204, doi:10.1673/031.010.20401 (2010).
244 Li, Q. R. *et al.* Analysis of midgut gene expression profiles from different silkworm varieties after exposure to high temperature. *Gene* **549**, 85-96, doi:10.1016/j.gene.2014.07.050 (2014).
245 Zhu, J. Y., Li, Y. H., Yang, S. & Li, Q. W. De novo assembly and characterization of the global transcriptome for Rhyacionia leptotubula using Illumina paired-end sequencing. *PLoS One* **8**, e81096, doi:10.1371/journal.pone.0081096 (2013).
246 Laskowska, E., Matuszewska, E. & Kuczynska-Wisnik, D. Small heat shock proteins and protein-misfolding diseases. *Current pharmaceutical biotechnology* **11**, 146-157 (2010).

247 Haslbeck, V., Kaiser, C. J. & Richter, K. Hsp90 in non-mammalian metazoan model systems. *Biochim Biophys Acta* **1823**, 712-721, doi:10.1016/j.bbamcr.2011.09.004 (2012).

248 Li, Z. & Srivastava, P. Heat-shock proteins. *Current protocols in immunology / edited by John E. Coligan ... [et al.]* **Appendix 1**, Appendix 1T, doi:10.1002/0471142735.ima01ts58 (2004).

249 King, A. M. & MacRae, T. H. Insect heat shock proteins during stress and diapause. *Annu Rev Entomol* **60**, 59-75, doi:10.1146/annurev-ento-011613-162107 (2015).

250 Qiu, X. B., Shao, Y. M., Miao, S. & Wang, L. The diversity of the DnaJ/Hsp40 family, the crucial partners for Hsp70 chaperones. *Cell Mol Life Sci* **63**, 2560-2570, doi:10.1007/s00018-006-6192-6 (2006).

251 Yamagishi, N., Ishihara, K., Saito, Y. & Hatayama, T. Hsp105 but not Hsp70 family proteins suppress the aggregation of heat-denatured protein in the presence of ADP. *FEBS Lett* **555**, 390-396 (2003).

252 Jedlicka, P., Mortin, M. A. & Wu, C. Multiple functions of Drosophila heat shock transcription factor in vivo. *Embo J* **16**, 2452-2462, doi:10.1093/emboj/16.9.2452 (1997).

253 Satyal, S. H., Chen, D., Fox, S. G., Kramer, J. M. & Morimoto, R. I. Negative regulation of the heat shock transcriptional response by HSBP1. *Genes Dev* **12**, 1962-1974 (1998).

254 Odunuga, O. O., Longshaw, V. M. & Blatch, G. L. Hop: more than an Hsp70/Hsp90 adaptor protein. *Bioessays* **26**, 1058-1068, doi:10.1002/bies.20107 (2004).

255 Ballinger, C. A. *et al.* Identification of CHIP, a novel tetratricopeptide repeat-containing protein that interacts with heat shock proteins and negatively regulates chaperone functions. *Mol Cell Biol* **19**, 4535-4545 (1999).

256 Panaretou, B. *et al.* Activation of the ATPase activity of hsp90 by the stress-regulated cochaperone aha1. *Mol Cell* **10**, 1307-1318 (2002).

257 Brix, J. *et al.* The mitochondrial import receptor Tom70: identification of a 25 kDa core domain with a specific binding site for preproteins. *J Mol Biol* **303**, 479-488, doi:10.1006/jmbi.2000.4120 (2000).

258 Mittapalli, O., Neal, J. J. & Shukle, R. H. Antioxidant defense response in a galling insect. *Proc Natl Acad Sci U S A* **104**, 1889-1894, doi:10.1073/pnas.0604722104 (2007).

259 Krishnan, N. & Sehnal, F. Compartmentalization of oxidative stress and antioxidant defense in the larval gut of Spodoptera littoralis. *Arch Insect Biochem Physiol* **63**, 1-10, doi:10.1002/arch.20135 (2006).

260 Mitchell, A. *et al.* The InterPro protein families database: the classification resource after 15 years. *Nucleic Acids Res* **43**, D213-221, doi:10.1093/nar/gku1243 (2015).

261 Sanchez, R. *et al.* Phylemon 2.0: a suite of web-tools for molecular evolution, phylogenetics, phylogenomics and hypotheses testing. *Nucleic Acids Res* **39**, W470-474, doi:10.1093/nar/gkr408 (2011).

262 Shi, G. Q., Yu, Q. Y. & Zhang, Z. Annotation and evolution of the antioxidant genes in the silkworm, Bombyx mori. *Arch Insect Biochem Physiol* **79**, 87-103, doi:10.1002/arch.21014 (2012).

263 Waterhouse, R. M. *et al.* Evolutionary dynamics of immune-related genes and pathways in disease-vector mosquitoes. *Science* **316**, 1738-1743, doi:10.1126/science.1139862 (2007).

264 Soudi, M., Zamocky, M., Jakopitsch, C., Furtmuller, P. G. & Obinger, C. Molecular evolution, structure, and function of peroxidasins. *Chemistry & biodiversity* **9**, 1776-1793, doi:10.1002/cbdv.201100438 (2012).

265 Ha, E. M. *et al.* Coordination of multiple dual oxidase-regulatory pathways in responses to commensal and infectious microbes in drosophila gut. *Nat Immunol* **10**, 949-957, doi:10.1038/ni.1765 (2009).
266 Kawahara, T., Quinn, M. T. & Lambeth, J. D. Molecular evolution of the reactive oxygen-generating NADPH oxidase (Nox/Duox) family of enzymes. *BMC Evol Biol* **7**, 109, doi:10.1186/1471-2148-7-109 (2007).
267 Perkins, A., Nelson, K. J., Parsonage, D., Poole, L. B. & Karplus, P. A. Peroxiredoxins: guardians against oxidative stress and modulators of peroxide signaling. *Trends Biochem Sci* **40**, 435-445, doi:10.1016/j.tibs.2015.05.001 (2015).
268 Corona, M. & Robinson, G. E. Genes of the antioxidant system of the honey bee: annotation and phylogeny. *Insect Mol Biol* **15**, 687-701, doi:10.1111/j.1365-2583.2006.00695.x (2006).
269 Yao, P. *et al.* Glutaredoxin 1, glutaredoxin 2, thioredoxin 1, and thioredoxin peroxidase 3 play important roles in antioxidant defense in Apis cerana cerana. *Free radical biology & medicine* **68**, 335-346, doi:10.1016/j.freeradbiomed.2013.12.020 (2014).
270 Kanzok, S. M. *et al.* Substitution of the thioredoxin system for glutathione reductase in Drosophila melanogaster. *Science* **291**, 643-646, doi:10.1126/science.291.5504.643 (2001).
271 Moskovitz, J. Methionine sulfoxide reductases: ubiquitous enzymes involved in antioxidant defense, protein regulation, and prevention of aging-associated diseases. *Biochim Biophys Acta* **1703**, 213-219, doi:10.1016/j.bbapap.2004.09.003 (2005).
272 Caers, J. *et al.* More than two decades of research on insect neuropeptide GPCRs: an overview. *Front Endocrinol (Lausanne)* **3**, 151, doi:10.3389/fendo.2012.00151 (2012).
273 Gäde, G. & Goldsworthy, G. J. Insect peptide hormones: a selective review of their physiology and potential application for pest control. *Pest Management Science* **59**, 1063-1075, doi:10.1002/ps.755 (2003).
274 Scherkenbeck, J. & Zdobinsky, T. Insect neuropeptides: Structures, chemical modifications and potential for insect control. *Bioorganic & Medicinal Chemistry* **17**, 4071-4084, doi:http://dx.doi.org/10.1016/j.bmc.2008.12.061 (2009).
275 Egekwu, N. *et al.* Comparison of synganglion neuropeptides, neuropeptide receptors and neurotransmitter receptors and their gene expression in response to feeding in Ixodes scapularis (Ixodidae) vs. Ornithodoros turicata (Argasidae). *Insect Molecular Biology* **25**, 72-92, doi:10.1111/imb.12202 (2016).
276 Wicker, T. *et al.* A unified classification system for eukaryotic transposable elements. *Nat Rev Genet* **8**, 973-982 (2007).
277 Suetsugu, Y. *et al.* Large scale full-length cDNA sequencing reveals a unique genomic landscape in a lepidopteran model insect, Bombyx mori. *G3 (Bethesda)* **3**, 1481-1492, doi:g3.113.006239 [pii]
10.1534/g3.113.006239 (2013).
278 dos Santos, G. *et al.* FlyBase: introduction of the Drosophila melanogaster Release 6 reference genome assembly and large-scale migration of genome annotations. *Nucleic Acids Res* **43**, D690-697, doi:gku1099 [pii]
10.1093/nar/gku1099 (2015).
279 van Schooten, B., Jiggins, C. D., Briscoe, A. D. & Papa, R. Genome-wide analysis of ionotropic receptors provides insight into their evolution in *Heliconius* butterflies. *BMC Genomics* **17**, 254, doi:10.1186/s12864-016-2572-y
10.1186/s12864-016-2572-y [pii] (2016).

280 Kanost, M. R. *et al.* Multifaceted biological insights from a draft genome sequence of the tobacco hornworm moth, *Manduca sexta*. *Insect Biochem Mol Biol* **76**, 118-147, doi:10.1016/j.ibmb.2016.07.005 (2016).

281 Christophides, G. K. *et al.* Immunity-related genes and gene families in Anopheles gambiae. *Science* **298**, 159-165 (2002).

282 Jones, D. T., Taylor, W. R. & Thornton, J. M. The rapid generation of mutation data matrices from protein sequences. *Comput Appl Biosci* **8**, 275-282 (1992).

283 Felsenstein, J. Confidence limits on phylogenies: An approach using the bootstrap. *Evolution* **39** 783-791 (1985).

284 Kumar, S., Stecher, G. & Tamura, K. MEGA7: Molecular Evolutionary Genetics Analysis Version 7.0 for Bigger Datasets. *Mol Biol Evol* **33**, 1870-1874, doi:10.1093/molbev/msw054 (2016).

285 Roy, A. *et al.* Diet dependent metabolic responses in three generalist insect herbivores Spodoptera spp. *Insect Biochem Mol Biol* **71**, 91-105, doi:10.1016/j.ibmb.2016.02.006 (2016).

286 Bray, N. L., Pimentel, H., Melsted, P. & Pachter, L. Near-optimal probabilistic RNA-seq quantification. *Nat Biotechnol* **34**, 525-527, doi:10.1038/nbt.3519 (2016).
